# Supplementary material for: Modular synthesis of clickable peptides via late-stage maleimidation on C(7)-H tryptophan
Source: Nat Commun. 2023 Jul 5;14:3973. doi: 10.1038/s41467-023-39703-y (PMC10322970; doi:10.1038/s41467-023-39703-y)
Supplement: Supplementary file 1 — Supplementary information [file 41467_2023_39703_MOESM1_ESM.pdf]

Supplementary Information

Modular Synthesis of Clickable Peptides via Late-stage Maleimidation on C(7)-H Tryptophan

Table of Contents

1. General Information……………………………………………………………......S2

2. Structure of C7-modification/cyclization Tryptophan-peptides……………………S2

3. General Procedure for Removal of Directing and Protecting Groups…………….S3

4. Experiment Section……………………………………………………………….…S4

A. General procedure for the synthesis of dipeptides and tripeptides

B. General procedure for the subtrates which modification with maleimides of N-substituted alkanoyl chlorides

C. General procedure of tetrapeptides, pentapeptides and hexapeptide

D. General procedure for Rh-catalyzed Maleimidylation of Trp containing amino acids, dipeptides, tripeptides, tetrapeptides and pentapeptides

E. General procedure of Rh-catalyzed cyclization of peptides

F. Studies on potential racemization

G. X-ray Data of **9a**

H. Removal of the directing group

I. Synthesis of cyclic peptide **10a**

J. Gram-scale reactions

K. Reaction of maleimide-modified substrate **3a** with Boc-Cys-OMe.

L. Synthesis of probe **10b**

M. Synthesis of Peptide Drug Conjugation **RGD-GFLG-DOX**

N. Synthesis of **RGD-GFLG**

O. General Procedures for Cell Culture, Staining Experiments, and Cytotoxicity Assays

P. Affinity determination using SPR

Q. Structural characterization of amino acids, peptides, cyclic peptides and others

R. **^{1}H NMR** and **^{13}C NMR** of products

1. General Information

All the reagents are obtained from commercial sources without further purification unless indicated. The water used in the laboratory comes from the Milli-Q reference system. Thin-layer chromatography (TLC) and silica gel for column chromatography comes from Qingdao Marine chemical plant (200-300 mesh). The peptide substrates and stapled peptide precursors were synthesized by traditional methods including liquid phase synthesis of peptides and solid synthesis of peptides. The spectra of absorption and fluorescence were analyzed using Molecular Devices SpectraMax M5. ^{1}H NMR spectra were obtained on AVANCE Ⅲ 500 (500 MHz), WNMR-I 400MHZ and AVANCE Ⅲ HD 600 instrument (600 MHz). ^{13}C NMR spectra were obtained on AVANCE Ⅲ 500 (126 MHz), WNMR-I 400MHZ (101 MHz) and AVANCE Ⅲ HD 600 instrument (151 MHz). ^{1}H NMR spectrum multiplicities as following: s (singlet), br (broad), d (doublet), t (triplet), q (quadruplet), m (multiplet). Cell imaging was performed using Leica TCS SP8. Reactions were detected by thin layer chromatography (TLC) under 254 nm or 365 nm with portable UV lamp and 2% ninhydrin stains in ethanol. High resolution mass spectrum (HRMS) with Agilent 6530 QTOF mass spectrometer.

2. Structure of C7-modification/cyclization Tryptophan-peptides

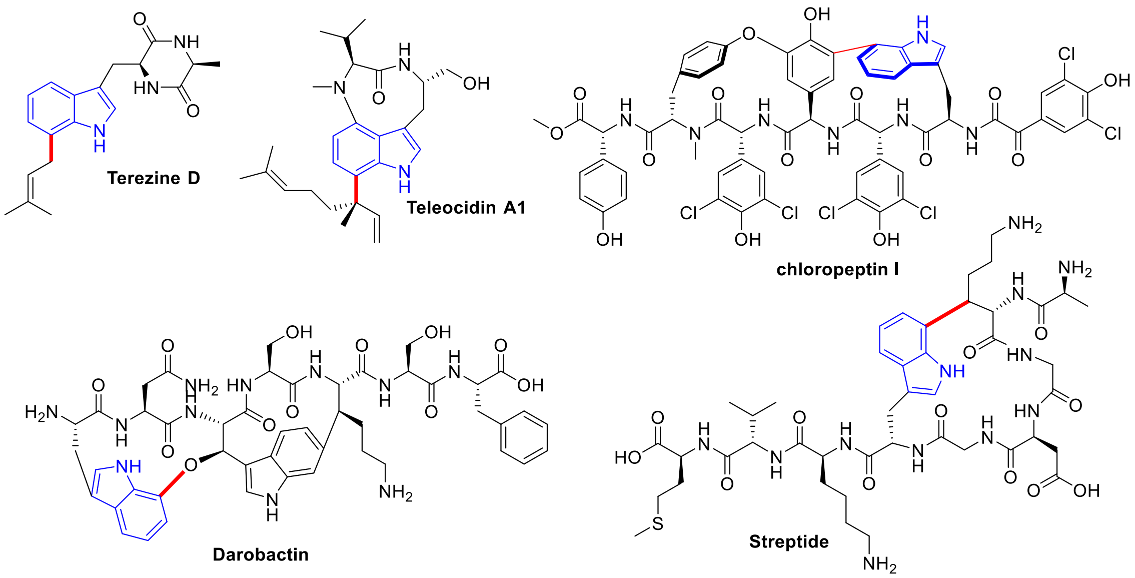

Supplementary Figure 1. Bioactive C7-modification/cyclization Tryptophan-peptide Derivatives and Natural Products

3. General Procedure for Removal of Directing and Protecting Groups

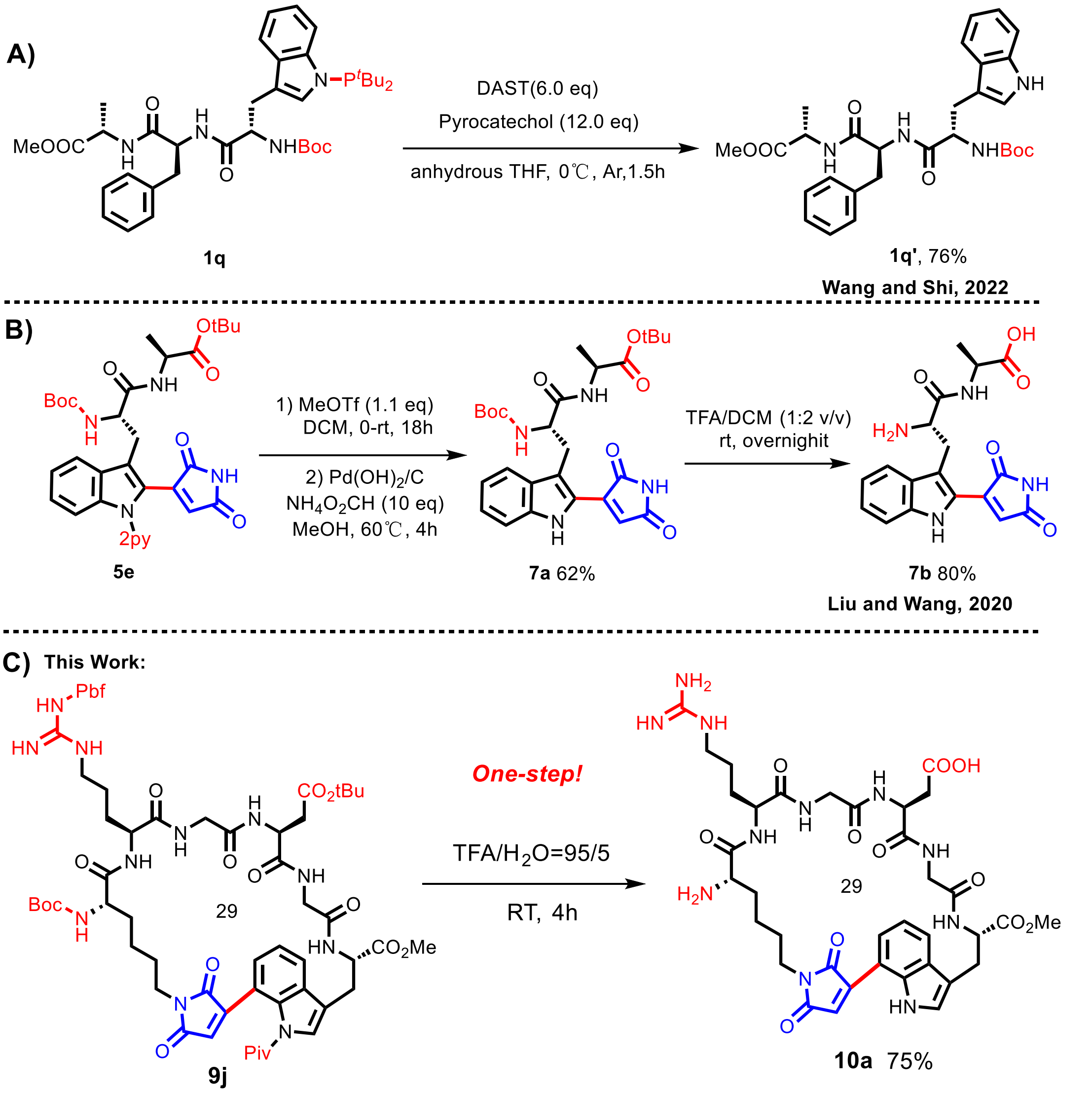

Supplementary Figure 2. General Procedure for Removal of Directing and Protecting Groups in Functionalized Peptides

3. Experimental Section

A. General procedure for the synthesis of dipeptides and tripeptides

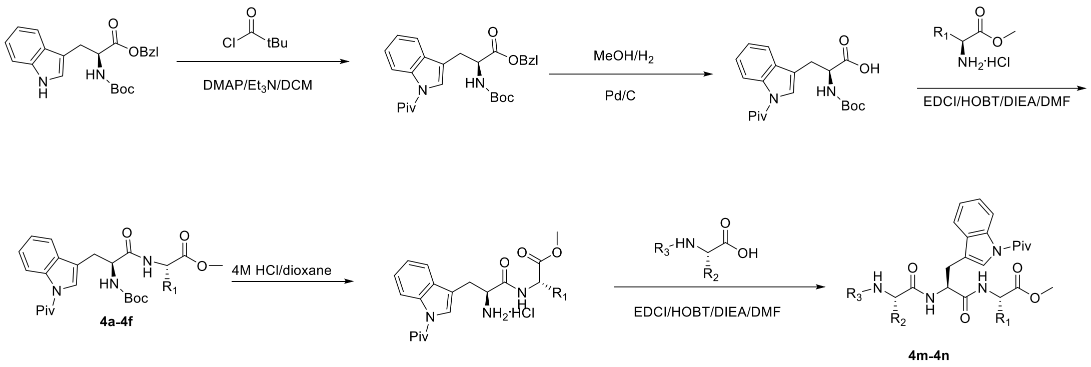

Supplementary Figure 3a. Preparation of dipeptides and tripeptides through solution-phase peptide synthesis.

Pivaloyl chloride (15.0 mmol) was added in portions at 0°C to a stirred solution of Boc-Trp-OBzl (3940mg, 10.0 mmol), DMAP (0.1 eq), Et_3N (20.0 mmol) in dry DCM (50 mL). The reaction was then allowed to warm to room temperature and stirred overnight. The mixed solution was washed with 1M dilute hydrochloric acid and saturated brine, and then dried with anhydrous sodium sulfate. After removal of the solvent, residue was purified on silica gel column chromatography to afford the corresponding acylation product Boc-Trp(Piv)-OBzl. Dissolve Boc-Trp(Piv)-OBzl (4.3 g, 9 mmol) in 50 mL methanol, then add 10% palladium/carbon (210 mg) in a nitrogen atmosphere, replace with hydrogen 3 times, and then stir at room temperature 12 hours. After completion, the reaction mixture was filtered and the filter cake was washed 3 times with methanol. The organic layers were combined and concentrated in vacuo, and residue was purified on silica gel column chromatography to afford the corresponding product Boc-Trp(Piv)-OH. Boc-Trp(Piv)-OH (388 mg, 1 mmol), EDCI (290 mg, 1.5 mmol), HOBT (202 mg, 1.5 mmol) and H-AA_1-OMe·HCl (1 mmol) were dissolved in 10mL DMF, then DIEA (390 mg, 3 mmol) was added, stirred in room temperature overnight. Upon completion, 30 mL EtOAc and 30 mL H_2O were added, the organic layer was separated and washed with 30 mL 1M HCl, 30 mL saturated sodium bicarbonate, 30 mL saturated sodium chloride and dried with anhydrous sodium sulfate, filtered, concentrated in vacuum to get dipeptides Boc-Trp(Piv)-AA_1-OMe. Next, the Boc-Trp(Piv)-AA_1-OMe (1 mmol) was dissolved in 10 mL 4M HCl/dioxane for 30 min, then concentrated in vacuum, diluting with ice ether, a lot of solid form, dried in vacuum to get H-Trp(Piv)-AA_1-OMe·HCl for the next step. R_3-AA_2-OH (1 mmol), EDCI (290 mg, 1.5 mmol), HOBT (202 mg, 1.5 mmol) and H-Trp(Piv)-AA_1-OMe·HCl (1 mmol) were dissolved in 10 mL DMF, then DIEA (390 mg, 3 mmol) was added, stirred in room temperature overnight. Upon completion, 30 mL EtOAc and 30 mL H_2O were added, the organic layer was separated and washed with 30 mL 1M HCl, 30 mL saturated sodium bicarbonate, 30 mL saturated sodium chloride and dried with anhydrous sodium sulfate, filtered, concentrated in vacuum to get tripeptides R_3-AA_2-Trp(Piv)-AA_1-OMe without further purified for the next step.

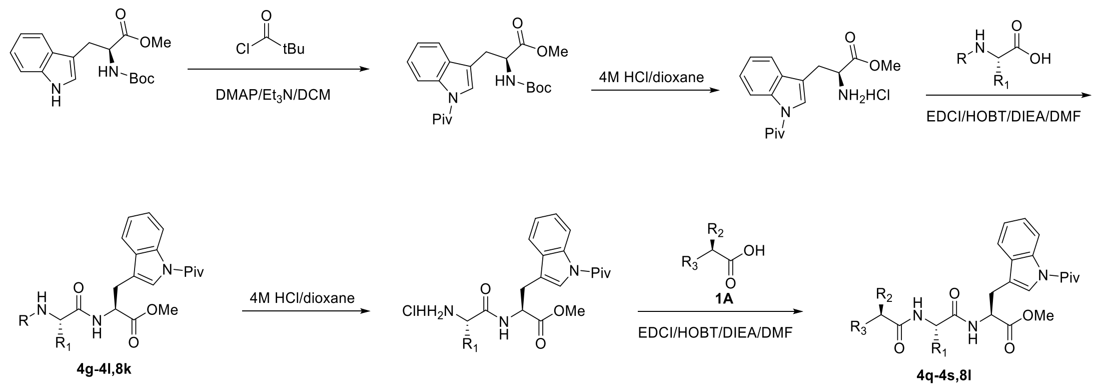

Supplementary Figure 3b. Preparation of dipeptides and tripeptides through solution-phase peptide synthesis.

Pivaloyl chloride (15.0 mmol) was added in portions at 0°C to a stirred solution of Boc-Trp-OMe (3180mg, 10.0 mmol), DMAP (0.1 eq), Et_3N (20.0 mmol) in dry DCM (50 mL). The reaction was then allowed to warm to room temperature and stirred overnight. The mixed solution was washed with 1M HCl and saturated brine, and then dried with anhydrous sodium sulfate. After removal of the solvent, residue was purified on silica gel column chromatography (ethyl acetate: petroleum ether= 1:7; R_f =0.2) to afford the corresponding acylation product Boc-Trp(Piv)-OMe. Next, the Boc-Trp(Piv)-OMe (9 mmol) was dissolved in 90 mL 4M HCl/dioxane for 30 min, then concentrated in vacuum, diluting with ice ether, a lot of solid form, dried in vacuum to get H-Trp(Piv)-OMe·HCl for the next step. H-Trp(Piv)-OMe·HCl (338 mg, 1 mmol), R-AA_1-OH (1 mmol), EDCI (290 mg, 1.5 mmol) and HOBT (202 mg, 1.5 mmol) were dissolved in 10 mL DMF, then DIEA (390 mg, 3 mmol) was added, stirred in room temperature overnight. Upon completion, 30 mL EtOAc and 30 mL H_2O were added, the organic layer was separated and washed with 30 mL 1M HCl, 30 mL saturated sodium bicarbonate, 30 mL saturated sodium chloride and dried with anhydrous sodium sulfate, filtered, concentrated in vacuum to get dipeptides R-AA_1-Trp(Piv)-OMe without further purified for the next step. The Boc-AA_1-Trp(Piv)-OMe (1 mmol) was dissolved in 10 mL 4M HCl/dioxane for 30 min, then concentrated in vacuum, diluting with ice ether, a lot of solid form, dried in vacuum to get H-AA_1-Trp(Piv)-OMe.HCl for the next step. **1A** (1 mmol), EDCI (290 mg, 1.5 mmol), HOBT (202 mg, 1.5 mmol) and H-AA_1-Trp(Piv)-OMe·HCl (1 mmol) were dissolved in 10 mL DMF, then DIEA (390 mg, 3 mmol) was added, stirred in room temperature overnight. Upon completion, 30 mL EtOAc and 30 mL H_2O were added, the organic layer was separated and washed with 30 mL 1M HCl, 30 mL saturated sodium bicarbonate, 30 mL saturated sodium chloride and dried with anhydrous sodium sulfate, filtered, concentrated in vacuum to get tripeptides **4q-4s** or **8l** without further purified for the next step.

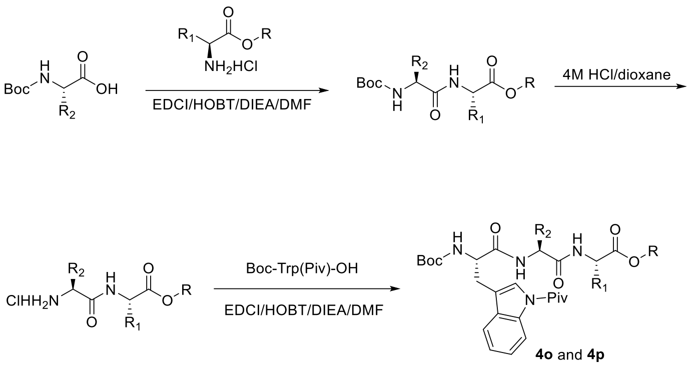

Supplementary Figure 3c. Preparation of tripeptides through solution-phase peptide synthesis.

Boc-AA_2-OH (1 mmol), EDCI (290 mg, 1.5 mmol), HOBT (202 mg, 1.5 mmol) and H-AA_1-OMe.HCl (1 mmol) were dissolved in 10mL DMF, then DIEA (390 mg, 3 mmol) was added, stirred in room temperature overnight. Upon completion, 30 mL EtOAc and 30 mL H_2O were added, the organic layer was separated and washed with 30 mL 1M HCl, 30 mL saturated sodium bicarbonate, 30 mL saturated sodium chloride and dried with anhydrous sodium sulfate, filtered, concentrated in vacuum to get dipeptides Boc-AA_2-AA_1-OMe. Next, the Boc-AA_2-AA_1-OMe (1 mmol) was dissolved in 10 mL 4M HCl/dioxane for 30 min, then concentrated in vacuum, diluting with ice ether, a lot of solid form, dried in vacuum to get H-AA_2-AA_1-OMe·HCl for the next step. Boc-Trp(Piv)-OH (388 mg, 1 mmol), EDCI (290 mg, 1.5 mmol), HOBT (202 mg, 1.5 mmol) and H-AA_2-AA_1-OMe·HCl (1 mmol) were dissolved in 10 mL DMF, then DIEA (390 mg, 3 mmol) was added, stirred in room temperature overnight. Upon completion, 30 mL EtOAc and 30 mL H_2O were added, the organic layer was separated and washed with 30 mL 1M HCl, 30 mL saturated sodium bicarbonate, 30 mL saturated sodium chloride and dried with anhydrous sodium sulfate, filtered, concentrated in vacuum to get tripeptides Boc-Trp(Piv)-AA_2-AA_1-OMe without further purified for the next step.

B. General procedure for the subtrates which modification with maleimides of N-substituted alkanoyl chlorides

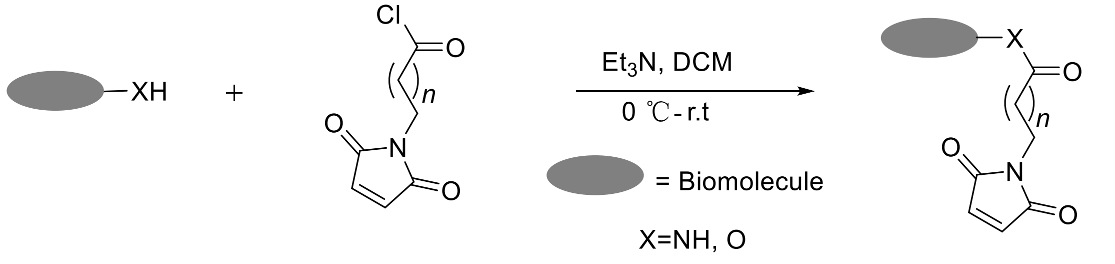

6a, 6e, 6f and **8a**

Supplementary Figure 4. Preparation of 3-maleimidopropionyl modification substrates.

Typically, the biomolecule compound (1 mmol) were dissolved in 10mL DCM, Et_3N (150 mg, 1.5 mmol) was added, then cooled to 0 °C. 3-Maleimidopropionyl chloride/11-maleimidoundecanoyl chloride (1.2 mmol) dissolved in 5mL DCM was dropwisesd to the reaction mixture, then warmed to room temperature

overnight. The reaction mixture was diluted with 10 mL EtOAc and 10 mL H_2O. The organic layer was washed with 10 mL 1M HCl, 10 mL saturated sodium bicarbonate, 10 mL saturated sodium chloride and dried with anhydrous sodium sulfate, filtered, concentrated in vacuum to get the crude product further purified by flash column to get desired products.

C. General procedure of tetrapeptides, pentapeptides and hexapeptide

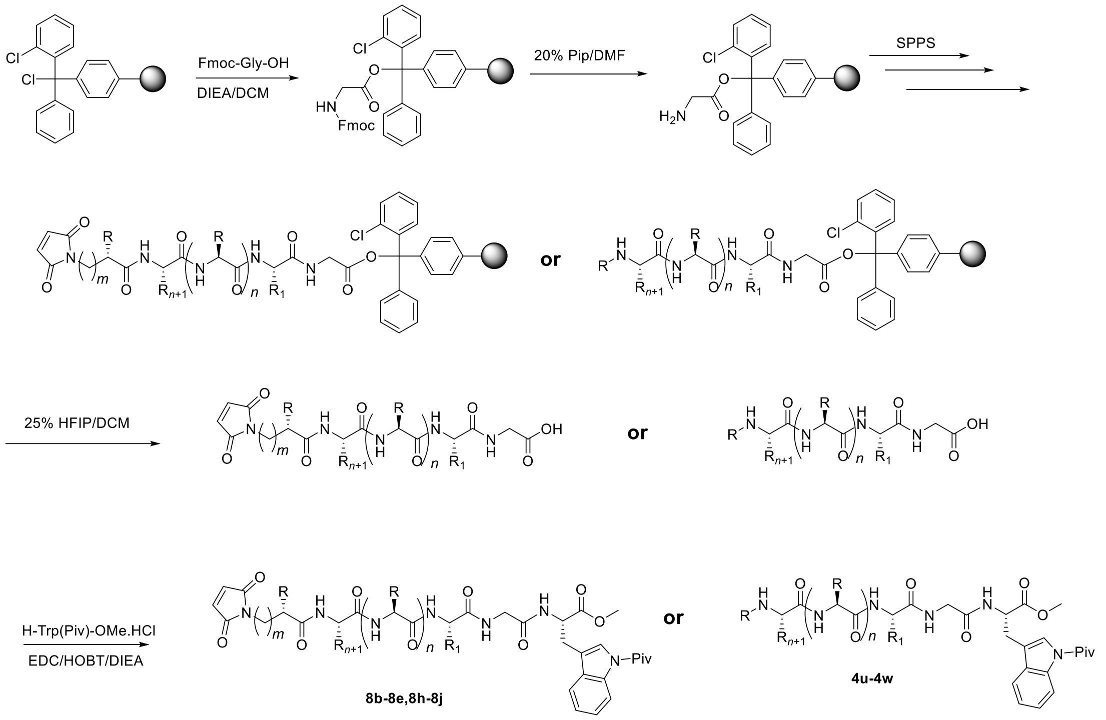

Supplementary Figure 5a. Procedure for tetrapeptides, pentapeptides and hexapeptide

The CTC Resin (300 mg, 0.3 mmol) was suspended in 5mL DCM, then Fmoc-Gly-OH (267 mg, 0.9 mmol) and DIEA (154.8 mg, 1.2 mmol) were added, reacted in the shaker, after 2 h, 300 μl MeOH was added for 10 min, then the Fmoc-Gly-CTC Resin washed with DMF for three times. Fmoc-Gly-CTC Resin deprotect the Fmoc with 20% piperidine/DMF for 30 min. After reaction, the H-Gly-CTC Resin was washed with DMF for four times. Subsequent amino acids were coupled using standard solid phase peptide synthesis (SPPS) until all the amino acid was incorporated. The peptides were removed from CTC Resin using 25% HFIP/DCM for 1 h, filtered, washed DCM for three times, combined the filtrate and concentrated in vacuum to get peptide. Finally, the linear peptide (0.2 mmol), H-Trp(Piv)-OMe·HCl (67.7 mg, 0.2 mmol), EDCI (58 mg, 0.3 mmol) and HOBT (40 mg, 0.3 mmol) were dissolved in 3 mL DMF, then DIEA (78 mg, 0.6 mmol) was added and stirred in room temperature for 12 h. Upon completion, 20 mL EtOAc and 20 mL H_2O were added, the organic layer was separated and washed with 20 mL 1M HCl, 20 mL saturated sodium bicarbonate, 20 mL saturated sodium chloride and dried with anhydrous sodium sulfate, filtered, concentrated in vacuum to get the linear peptide without purified for the next step.

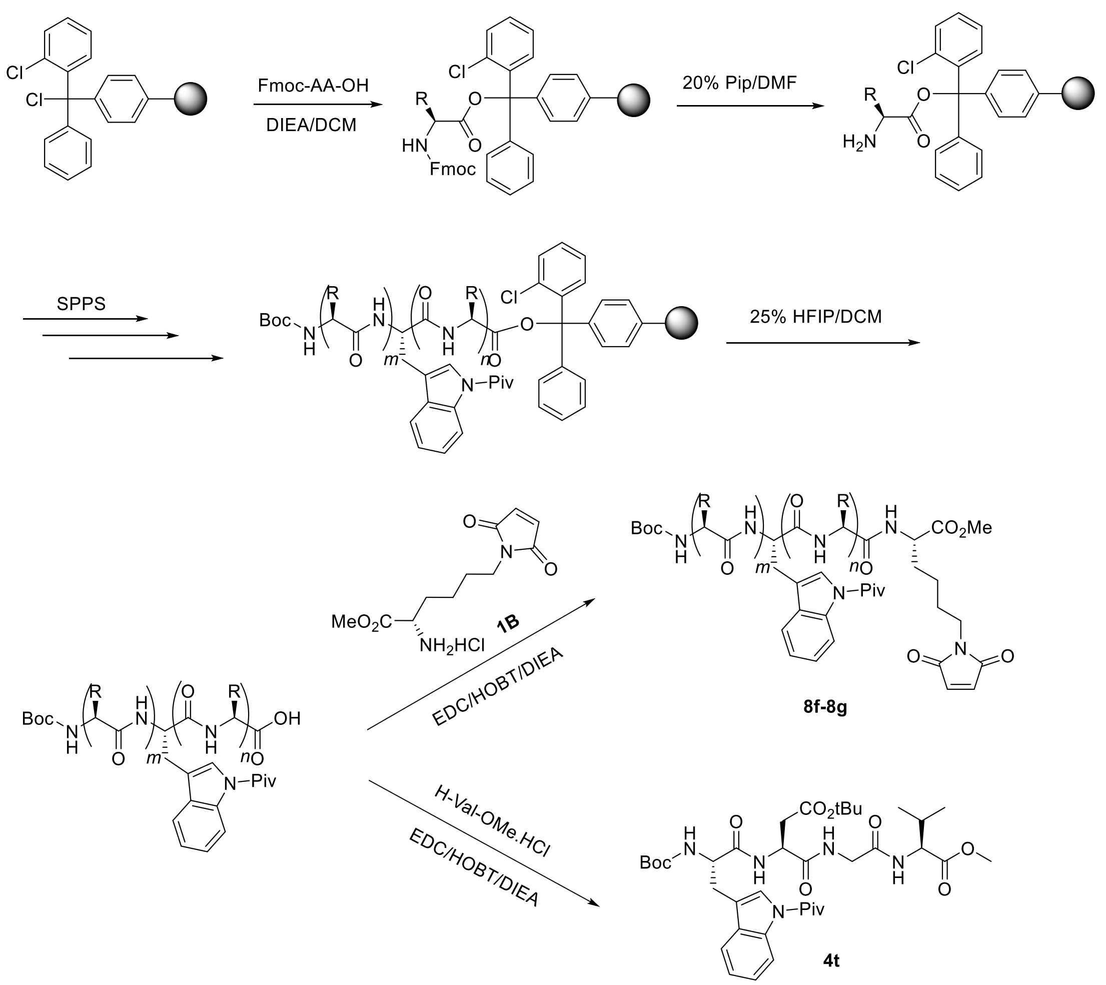

Supplementary Figure 5b. Procedure for tetrapeptides, pentapeptides and hexapeptide

The CTC Resin (300 mg, 0.3 mmol) was suspended in 5mL DCM, then Fmoc-AA-OH (0.9 mmol) and DIEA (154.8 mg, 1.2 mmol) were added and stirred for 2 h. 300 μl MeOH was added, and after 10 min, the Fmoc-AA-CTC Resin was washed with DMF for three times. The Fmoc was deprotected with 20% piperidine/DMF for 30 min. After reaction, the H-AA-CTC Resin washed with DMF for four times. Subsequent amino acids were coupled using standard solid-phase peptide synthesis (SPPS). The peptides were removed from CTC Resin using 25% HFIP/DCM for 1 h, filtered, and the resins were washed DCM for three times, combined the filtrate and concentrated in vacuum to get peptides. Finally, the linear peptides (0.2 mmol), **1B**/H-Val-OMe·HCl (0.2 mmol), EDCI (60 mg, 0.3 mmol) and HOBT (40 mg, 0.3 mmol) were dissolved in 3 mL DMF, then DIEA (78 mg, 0.6 mmol) was added, stirred in room temperature for 12 h. Upon completion, 30 mL EtOAc and 30 mL H_2O were added, the organic layer was separated and washed with 30 mL 1M HCl, 30 mL saturated sodium bicarbonate, 30 mL saturated sodium chloride and dried with anhydrous sodium sulfate, filtered, concentrated in vacuum to get the linear peptides without purified for the next step.

D. General procedure for Rh-catalyzed Maleimidylation of Trp containing amino acids, dipeptides, tripeptides, tetrapeptides and pentapeptides

Typically, the Trp containing amino acid/peptide substrate (0.2 mmol), $[RhCp^*Cl_2]_2$ (6.2 mg, 0.01 mmol)

were suspended in 1.5-2 mL DCM, then maleimide/N-substituted maleimide derivatives (0.6 mmol), $AgNTf_2$ (15.5mg, 0.04 mmol) and Ag_2O (69.5 mg, 0.3 mmol) were added. The tube was sealed and the mixture was heated to 80 °C for 6-12 h. After cooling to ambient temperature, diluted with DCM and passed through a short celite pad, the solvent was evaporated in vacuum to get the crude product further purified by flash column or PTLC.

E. General procedure of Rh-catalyzed cyclization of peptides

Typically, the linear peptide containing Trp (0.12 mmol), $[RhCp^*Cl_2]_2$ (7.4 mg, 0.012 mmol) were suspended in 10 mL DCM, then $AgNTf_2$ (18.6 mg, 0.048 mmol) and Ag_2O (41.7 mg, 0.18 mmol) were added. The tube was sealed and the mixture was heated to 80 °C for 24 h. After cooling to ambient temperature, diluted with DCM and passed through a short celite pad. The solvent was evaporated in vacuum to get the crude product further purified by flash column or PTLC.

F. Studies on potential racemization

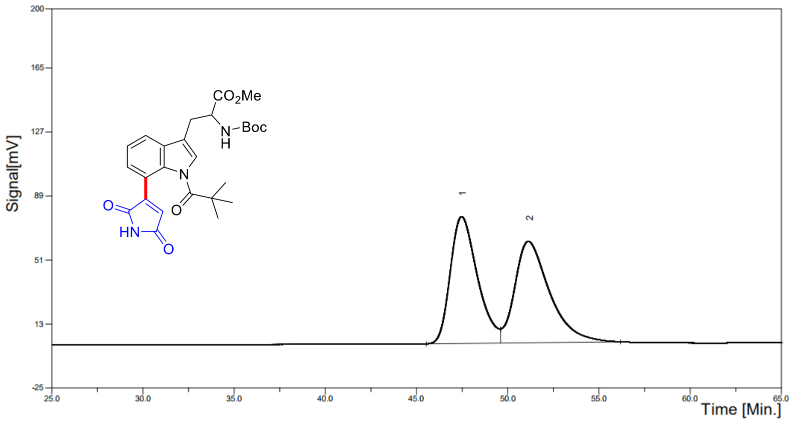

组分表

#	组分名	保留时间(min)	峰高(mV)	峰面积(mV. sec)	面积百分比(%)
1	Unknown	47.44250	75.32	7794.39	49.4225
2	Unknown	51.11167	60.20	7976.56	50.5775
合计			135.52	15770.96	100

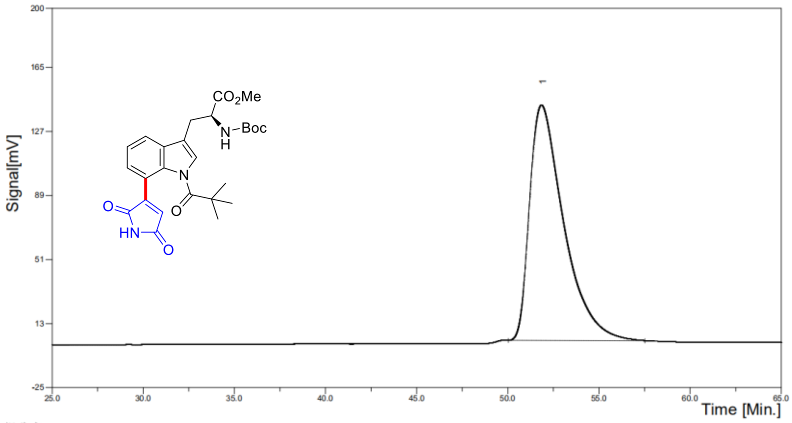

组分表

#	组分名	保留时间(min)	峰高(mV)	峰面积(mV. sec)	面积百分比(%)
1	Unknown	51.82583	139.64	18097.84	100.0000
合计			139.64	18097.84	100

Supplementary Figure 6. HPLC spectra of racemate and **3a**. Chromatographic column: Daicel Chiralpak AD-H 5μm, solvent: *n*-hexane/^{i}PrOH, wavelength: 254.

G. X-ray Data of 9a

Supplementary Table 1 Crystal data and structure refinement for 9a.

Identification code	mo_211028_YSC1021_0m
Empirical formula	$C_{34}H_{46}N_3O_7S$
Formula weight	640.80
Temperature/K	170.0
Crystal system	orthorhombic
Space group	$P2_12_12_1$
a/Å	9.3763(4)
b/Å	16.2241(7)
c/Å	23.2477(10)
α/°	90
β/°	90
γ/°	90
Volume/Å^3	3536.5(3)
Z	4
ρ_{calc}g/cm^3	1.204
μ/mm^{-1}	0.140
F(000)	1372.0
Crystal size/mm^3	0.32 × 0.07 × 0.05
Radiation	MoKα (λ = 0.71073)
2Θ range for data collection/°	4.684 to 54.236
Index ranges	-12 ≤ h ≤ 10, -20 ≤ k ≤ 20, -29 ≤ l ≤ 29
Reflections collected	35967
Independent reflections	7794 [R_{int} = 0.0584, R_{sigma} = 0.0435]
Data/restraints/parameters	7794/0/420
Goodness-of-fit on F^2	1.023
Final R indexes [I>=2σ (I)]	R_1 = 0.0528, wR_2 = 0.1264
Final R indexes [all data]	R_1 = 0.0734, wR_2 = 0.1402
Largest diff. peak/hole / e Å^{-3}	0.43/-0.51
Flack parameter	0.13(5)

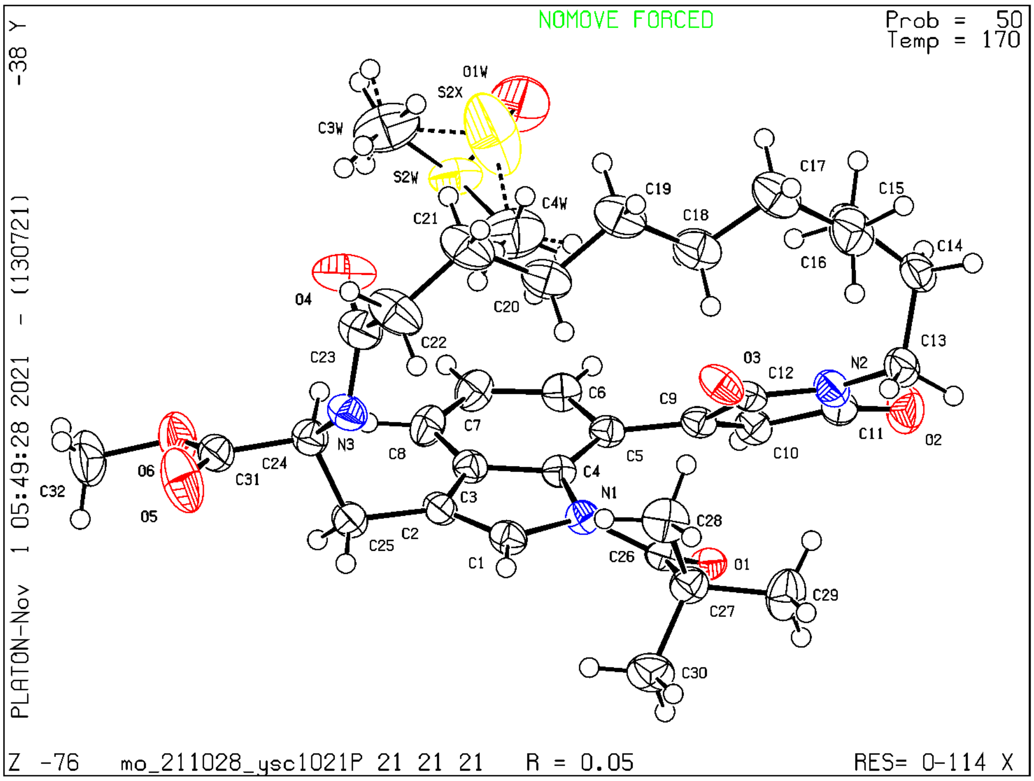

H. Removal of the directing group

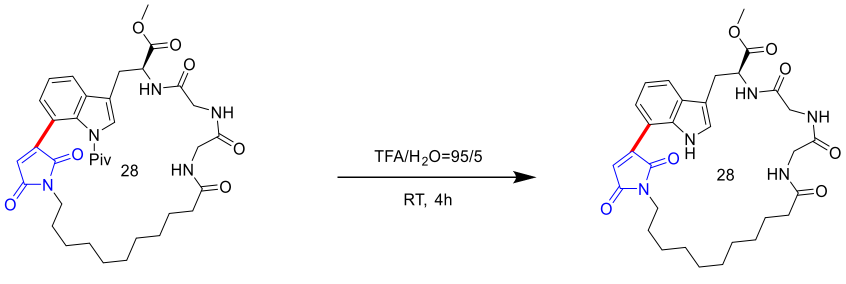

Supplementary Figure 7. Removal of the directing group

Cyclic peptide **9h** (67.7mg, 0.1 mmol) was removed the directing group using a cock tail of 2 mL TFA/H_2O=95/5 at room temperature for 4 h. Upon completion, saturated sodium bicarbonate was added, extracted with ethyl acetate, dried over anhydrous sodium sulfate, concentrated in vacuo, and further purified by flash column to afford 46 mg **9ha** as a yellow solid in 78% yield.

I. Synthesis of cyclic peptide 10a

Linear peptide **8j** (156 mg, 0.12 mmol), $[RhCp^*Cl_2]_2$ (7.4 mg, 0.012 mmol) were suspended in 10 mL DCM, then $AgNTf_2$ (18.6 mg, 0.048 mmol) and Ag_2O (41.7 mg, 0.18 mmol) were added. The tube was sealed and the mixture was heated to 80 °C for 24 h. After cooling to ambient temperature, diluted with DCM and passed through a short celite pad. The solvent was evaporated in vacuum to get the crude

product further purified by flash column (diluting with DCM/MeOH = 50:1-20:1) to afford cyclic peptide **9j** 50 mg yellow solid in 32% yield. Cyclic peptide **9j** (26 mg, 0.02 mmol) was then deprotected using a cock tail of 1 mL TFA/H_2O=95/5 at room temperature for 4 h. After diluted with ice ether, the solid formed was further purified by RP-HPLC to afford 12 mg **10a** as a yellow solid in 75% yield.

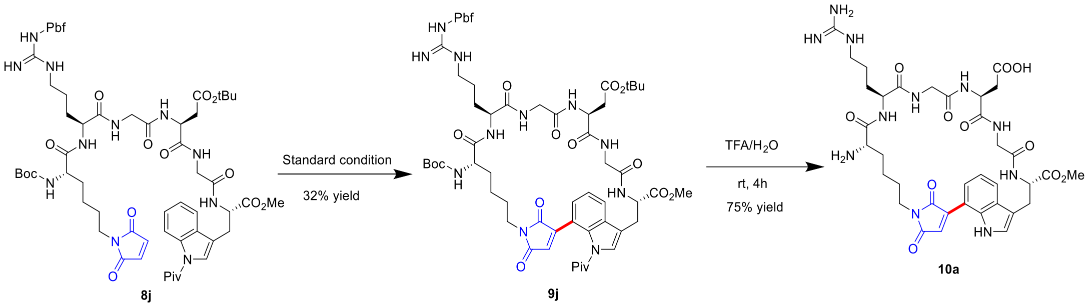

Supplementary Figure 8. Procedure for cyclic peptide **10a**

J. Gram-scale reactions

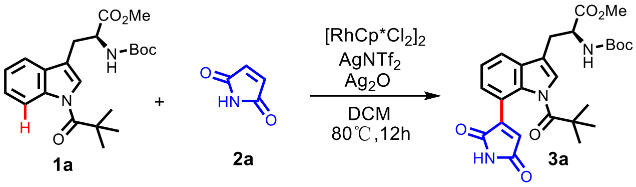

Supplementary Figure 9. Gram-scale reactions

Trp **1a** (1.21 g, 3 mmol), $[RhCp^*Cl_2]_2$ (92.7 mg, 0.15 mmol) were suspended in 20 mL DCM, then maleimide (873 mg, 9 mmol), $AgNTf_2$ (232.8 mg, 0.6 mmol) and Ag_2O (1.04 g, 4.5 mmol) were added. The tube was sealed and the mixture was heated to 80 °C for 6-12 h. After cooling to ambient temperature, diluted with DCM and passed through a short celite pad, the solvent was evaporated in vacuum to get the crude product further purified by flash column (ethyl acetate: petroleum ether= 1:3; R_f =0.2) to afford 0.89 g **3a** as a yellow solid in 60% yield.

K. Reaction of maleimide-modified substrate 3a with Boc-Cys-OMe

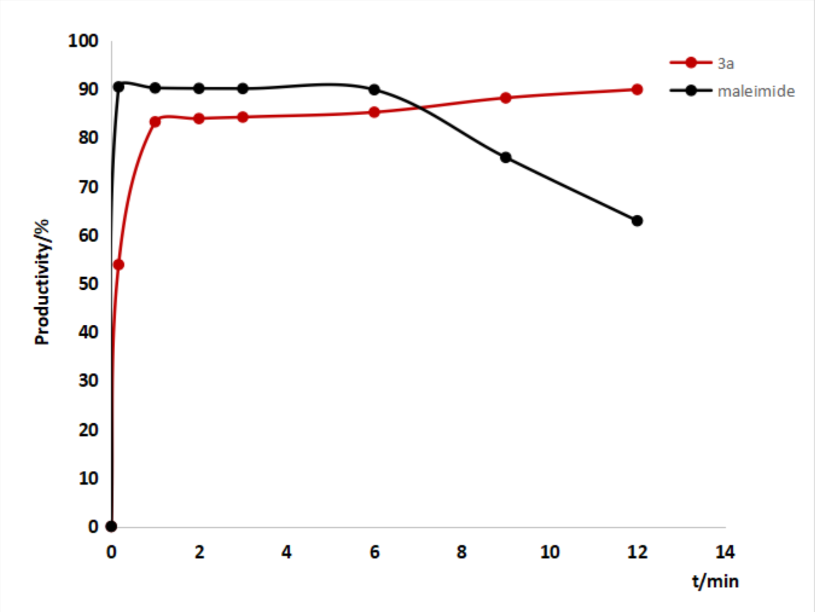

Supplementary Figure 10. Maleimide-modified substrate **3a** (red curve)/maleimide (balck curve) reaction with Boc-Cys-OMe. In alkaline solution, maleimide-modified substrate **3a**/maleimide was reacted with Boc-Cys-OMe for 0-12 min.

L. Synthesis of probe 10b

Rhodamine B derivative **P1** (20mg, 0.03 mmol) was added to 1 mL 4M HCl/dioxane for 30 min. Then the mixture was concentrated in vacuum, and ice ether was added to obtain the white solid, which dried in vacuum to get **P2**. **P2** (0.03 mmol), Boc-Cys(Trt)-OH (17mg, 0.04 mmol), EDCI (9 mg, 1.5 eq) and HOBT (6 mg, 1.5 eq) were dissolved in 1 mL DMF, then DIEA (12 mg, 0.09 mmol) was added, stirred in room temperature overnight. Upon completion, 10 mL EtOAc and 10 mL H_2O were added, and the organic layer was separated and washed with 10 mL 1M HCl, 10 mL saturated sodium bicarbonate, 10 mL saturated sodium chloride and dried with anhydrous sodium sulfate, filtered, concentrated in vacuum to get product **P3** without further purified for the next step. TFA (8 µL, 4% v/v) was added to a solution of **P3** (20 mg) and triethyl silane (4 µL, 0.05 mmol) in CH_2Cl_2 (200 µL), and the mixture was stirred for 60 minutes. The mixture was washed with saturated $NaHCO_3$ solution. Extract the mixture with CH_2Cl_2, wash the organic layers with brine. Dry the organic layers over Na_2SO_4. Evaporate the solvent under reduced pressure, and further purified by flash column to get product 10mg **10b** in 67% yeild.

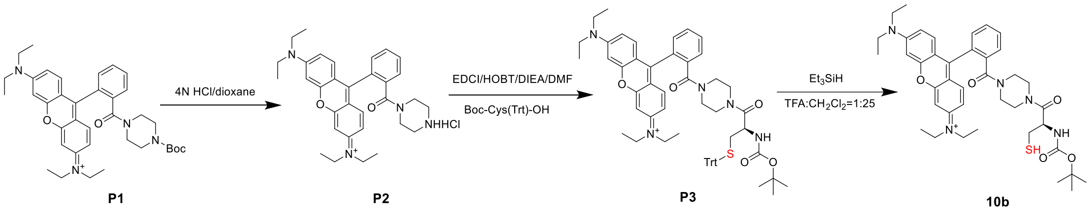

Supplementary Figure 11. Procedure for probe **10b**

M. Synthesis of Peptide Drug Conjugation RGD-GFLG-DOX

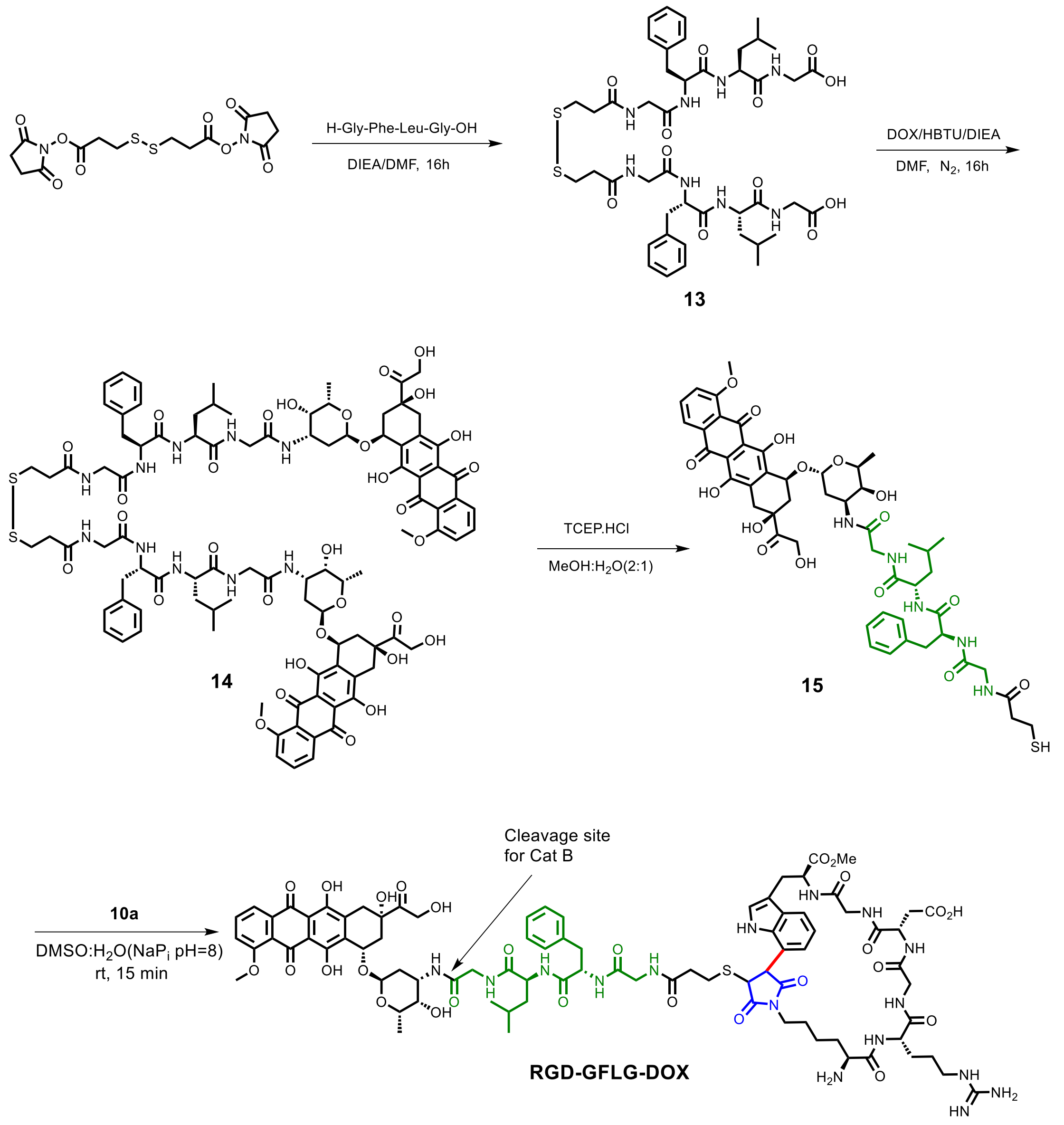

Supplementary Figure 12. Procedure for **RGD-GFLG-DOX**

As shown in **Supplementary Figure 12**, 3,3'-dithiobis(succinimidyl propionate) 130mg (0.32 mmol), H-Gly-Phe-Leu-Gly-OH 300mg (0.76 mmol) and dry DMF 2 mL were added to the flask, and the reaction mixture was stirred. Then, DIEA 133uL was added with stirring, and the solution was stirred and reacted at the room temperature for 16 hours. After the reaction was completed, 30 mL of EtOAc and 30 mL of 1M HCl were added. The organic layer was separated, washed with 30 mL of 1M HCl twice and 30 mL of saturated NaCl solution, dried over anhydrous sodium sulfate and filtered, and was concentrated in vacuo to give compound **13**, which was used in the next step without further purification. Dissolve compound **13** 228 mg (0.24 mmol) and DOX·HCl 307mg (0.53 mmol) in 2 mL of dry DMF. Add 204mg (276μL) of DIEA and stir the solution under nitrogen in the dark for 10 min. Dissolve 200 mg (0.53mmol) of HBTU in 1 mL of dry DMF and add to the solution. Stir the reaction mixture under nitrogen in the dark for 16 h. After the reaction was completed, 30 mL of 1M HCl was added, a large amount of solid was precipitated, filtered, the solid residue was washed twice with 1M HCl and four

times with diethyl ether, and dried to obtain a brown-red compound **14**, which was directly used in the next step without further purification. 100 mg (0.05 mmol) of compound **14** and 21.4 mg (0.075 mmol) of tris-(2-carboxyethyl)phosphine hydrochloride were dissolved in a MeOH:H_2O (2:1) mixture. The reaction mixture was stirred under nitrogen in the dark overnight. The methanol was completely removed and the product was extracted from the aqueous layer with ethyl acetate. The organic layer was collected and washed with saturated $NaHCO_3$ solution. Dry the organic layers over Na_2SO_4. Evaporate the solvent under reduced pressure, and further purified by flash column (DCM:MeOH=10:1, Rf=0.5) to obtain 70 mg of product **15** in a yield of 70%. Dissolve 5 mg (0.006 mmol) of **10a** in 0.4 mL of DMSO, then add 0.2 mL of H_2O (NaPi pH 8), stir to make it clear, dissolve 9.3 mg (0.009 mmol) of compound **15** in DMSO and add it to mixture reaction at room temperature. After 15 min of reaction, the obtained mixed product was further purified by RP-HPLC to obtain 7.6 mg of brown-red solid **RGD-GFLG-DOX** in a yield of 68%.

N. Synthesis of RGD-GFLG

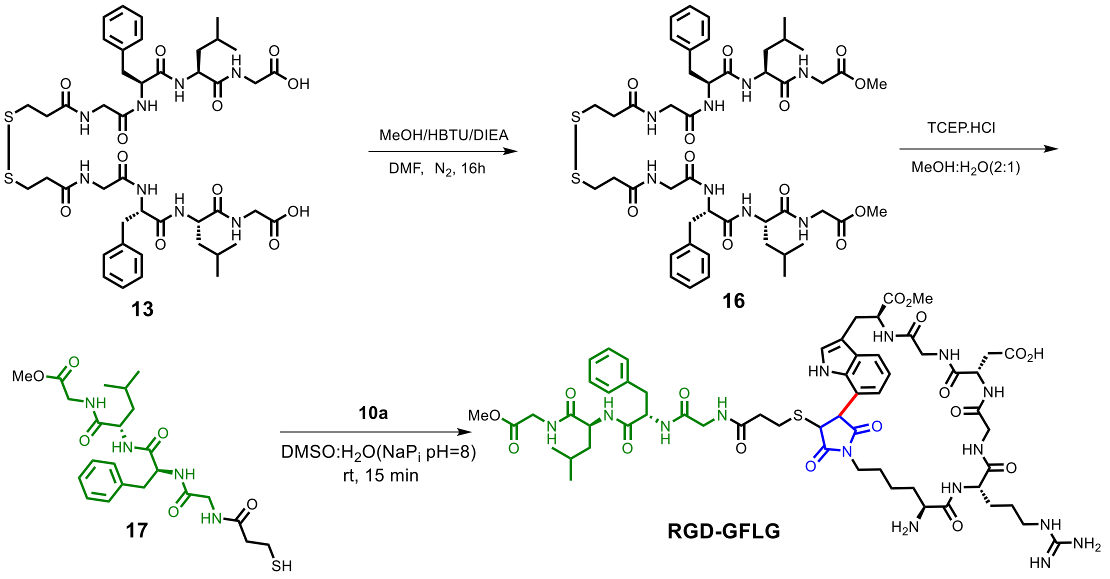

Supplementary Figure 13. Procedure for **RGD-GFLG**

As shown in **Supplementary Figure 13**, dissolve compound **13** 228 mg (0.24 mmol) and MeOH 23mg (29μL, 0.72 mmol) in 2 mL of dry DMF. Add 204mg (276μL) of DIEA and stir the solution under nitrogen in the dark for 10 min. Dissolve 200 mg (0.53mmol) of HBTU in 1 mL of dry DMF and add to the solution. Stir the reaction mixture under nitrogen in the dark for 16 h. After the reaction was completed, 30 mL of 1M HCl was added, a large amount of solid was precipitated, filtered, the solid residue was washed twice with 1M HCl and four times with diethyl ether, and dried to obtain a compound **16**, which was directly used in the next step without further purification. 50 mg (0.05 mmol) of compound **16** and 21.4 mg (0.075 mmol) of tris-(2-carboxyethyl)phosphine hydrochloride were dissolved in a MeOH:H_2O (2:1) mixture. The reaction mixture was stirred under nitrogen overnight. The methanol was completely removed and the product was extracted from the aqueous layer with ethyl acetate. The organic layer was collected and washed with saturated $NaHCO_3$ solution. The organic layers were dried over Na_2SO_4. The solvent was evaporated under reduced pressure, and the compound **17** was obtained in high

purity without further purification. Dissolve 5 mg (0.006 mmol) of **10a** in 0.4 mL of DMSO, then add 0.2 mL of H_2O (NaPi pH 8), stir to make it clear, dissolve 4.5 mg (0.009 mmol) of compound **17** in DMSO and add it to mixture reaction at room temperature. After 15 min of reaction, the obtained mixed product was further purified by RP-HPLC to obtain 6.0 mg of colorless solid **RGD-GFLG** in a yield of 76%.

O. General Procedures for Cell Culture, Staining Experiments, and Cytotoxicity Assays

Cell culture. Following cell lines were used in this study: A549 (TCH-C116), HeLa (TCH-C193), MCF-7 (TCH-C247), U87MG(TCH-C367), MIHA (CL0469), LO2 (CL0192), HUVEC (TCH-C406), And A549 (TCH-C116), HeLa (TCH-C193), MCF-7 (TCH-C247), U87MG(TCH-C367) and HUVEC (TCH-C406) originally purchased from Suzhou Starfish Biotechnology Co. LTD. MIHA (CL0469) and LO2 (CL0192) originally purchased from Hunan Fenghui Biotechnology Co. LTD.
MIHA and LO2 cells were cultured in RPMI 1640 (Hyclone) medium supplemented with 10% FBS (Titan). A549, HeLa, MCF-7, U87MG and Huvec cells were cultured in DMEM (Hyclone) medium supplemented with 10% FBS. All cells were cultured at 37 °C in an atmosphere of 5% CO_2. All media contained 100 units/mL penicillin and 100 μg/mL streptomycin.

Confocal fluorescence imaging of peptides in A549 and HeLa cells. A549 and HeLa cells were cultured in DMEM high glucose media supplemented with 10% fetal bovine serum, 1% Penstrep, 0.2% Amphotericin B. The cells were grown overnight at 37 °C incubator with 5% CO_2. A549 and HeLa cells were seeded at a density of 3×10^5 cells in 35 mm glass-bottomed dish and kept overnight prior to cell imaging studies. After 24h, the cells were washed twice with warm DMEM media, incubated at 37 °C with 5 μM peptides for 60 min, and then pretreated with 5 μM probe for 15 min. Images were taken using the Zeiss LSM 800 confocal fluorescence microscope.

Flow cytometry analysis for cell surface integrin proteins. Cells were cultured in DMEM high glucose medium supplemented with 10% fetal bovine serum, 1% Streptococcus vale, 0.2% amphotericin B. Cells are grown overnight with 5% CO2 in a 37 °C incubator. Cells are seeded in 6-well plate dishes at a density of 3×105 cells and kept overnight before cell detection. After 24 h, wash the cells twice with warm DMEM medium, incubate with 5 μM peptide for 60 min at 37 °C, and then pre-treat with a 5 μM probe for 15 min. Before measurement by flow cytometry, cells were scraped off gently and collected into a clean 2 mL centrifuge tube. Then, cells were spun down (1000 rpm, room temperature, 3 min). After discarding the supernatant, 1 mL of warm PBS was added gently to resuspend the cell pellet. Finally, cells were analyzed on a Guava (Millipore) flow cytometer equipped with a 488 nm Ar laser, and fluorescence was collected by PE channel. And the flow cytometric data was analyzed with flow analysis software Flow Jo.

Cytotoxicity was assessed by MTT assay. Various cells were cultured in DMEM medium in 96-well microplates in at 37°C under 5% CO_2 for 12h. The medium was next replaced with fresh medium containing various concentrations of cyclic peptides, **RGD-GFLG**, **RGD-GFLG-DOX** and DOX (0-40 μM). Each concentration was tested in triplicate. After 36 h, cells were washed twice with PBS buffer

and incubated with 0.5 mg/mL MTT reagent for 4 h at 37°C. 150 μL DMSO was then added to dissolve formazan. Measure the absorbance at 510 nm in a microplate reader. Cell viability (%) was calculated according to the following equation: Viability = (mean Abs. of treated wells/mean Abs. of control wells) × 100%.

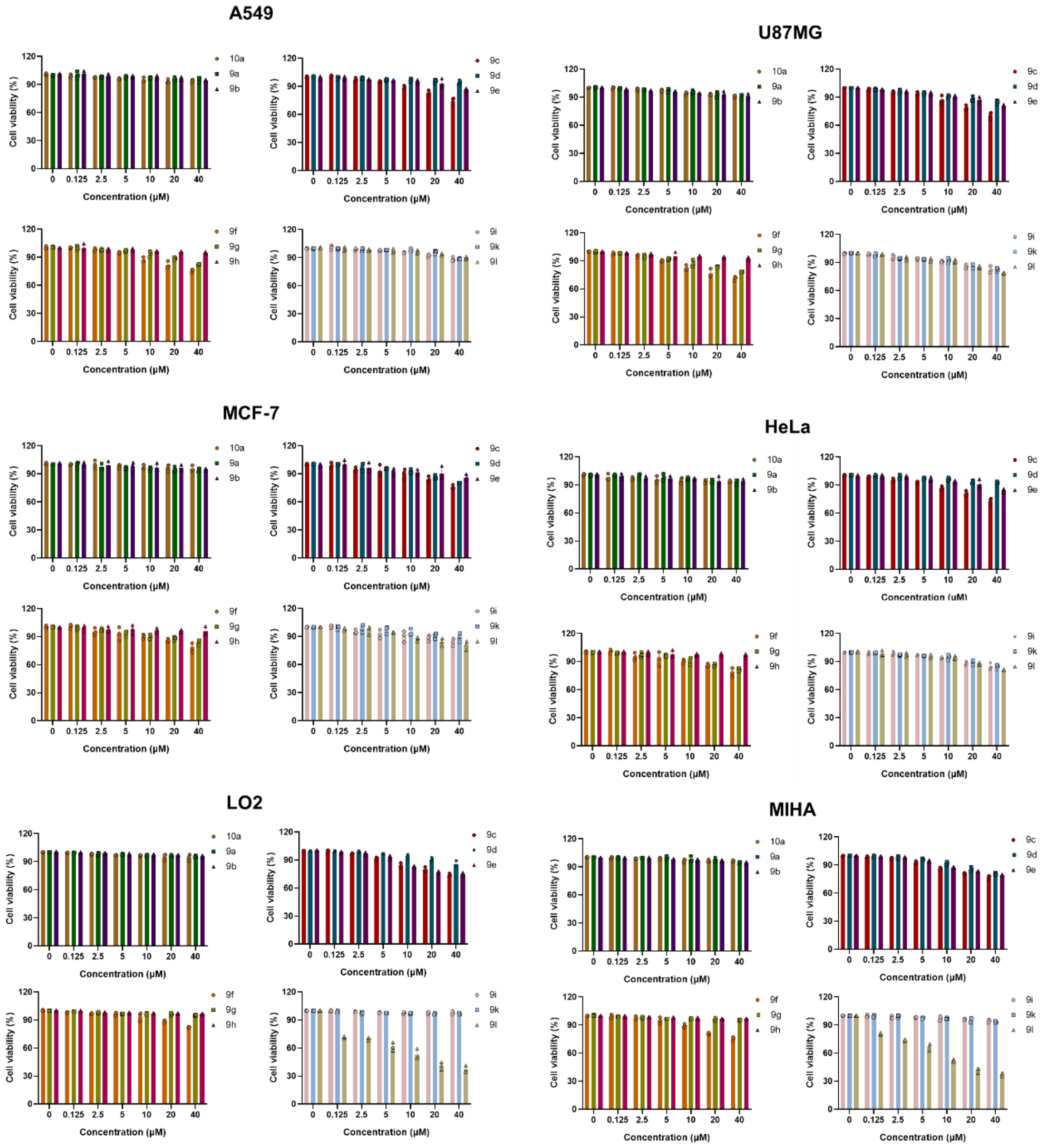

Supplementary Figure 14. Cytotoxicity of cyclic peptides on A549, U87MG, MCF-7, HeLa, LO2 and MIHA cells. Different cells were incubated with various concentrations of cyclic peptides (0-40 μM). After adding drugs, the cells were further incubated for another 36h. Data were presented as mean ± S.D. n = 3 biologically independent samples per group.

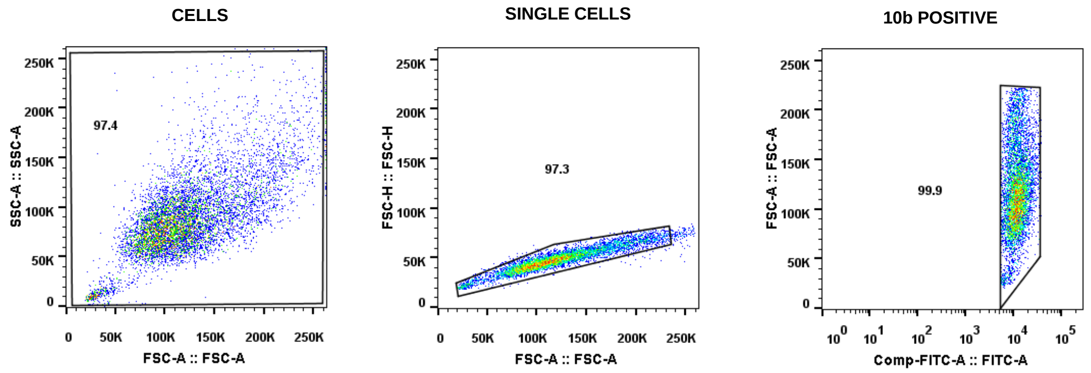

Supplementary Figure 15. Gating Strategy for **10b** positive cells. The preliminary forward scatter (FSC)side scatter (SSC) gates of the starting cell populations included 5,000 events. Debris (SSC-A VS FSC-A) and doublets (FSC-H vs FSC-A) were excluded. Boundaries between "positive" and "negative" staining were set at 5.4 × 10^4 for **10b** staining.

P. Affinity determination using SPR

The experiments were conducted on a BIAcore T200 instrument using CM5 sensor chips at 25°C, and the data were analyzed using the BIAcore T200 evaluation software (GE Healthcare) following the manufacturer's instructions. A total of 200 µl of protein (100 µg/ml) purified and adjusted to pH 4.0 as described above was immobilized on the chip surface at a flow rate of 10 µl/min (proteins were immobilized on channels 2, 3, and 4, and a new chip was used if necessary to immobilize the protein on channel 2). Channel 3 was blocked with 1 M ethanolamine (10 µl/min, 420 s), while channel 1 was used as a reference and only blocked, followed by equilibration with PBS in both channels. The peptide was diluted to a series of concentrations (100, 50, 25, 12.5, 6.25, and 3.125 µM or 200, 150, 100, 50, 25, 12.5 and 6.25 µM in PBS), and injected at a flow rate of 10 µl/min for 120 s and then dissociated for 180 s in each run. The data were collected from the sample pool and corrected by subtracting the data from the reference pool using the BIAcore T200 control software (v. 2.0, GE Healthcare). The binding and dissociation constants were obtained by globally fitting the data to a 1:1 Langmuir binding model using the BIAcore T200 evaluation software (v. 2.0, GE Healthcare). The data were exported to GraphPad software to generate the final figures.

Supplementary Table 2. Equilibrium Dissociation Constant (K_D) Values of Cyclopeptides Determined by the SPR Assay

integrin	peptide	K_D（M）	ka（1/Ms）	kd(1/s)
$\alpha v\beta_3$	10a	6.75E-7	42325.156	0.028
$\alpha_5\beta_1$	10a	8.36E-6	15322.862	0.128
$\alpha v\beta_5$	10a	6.13E-6	12523.125	0.077
$\alpha v\beta_3$	10c	6.55E-5	10251.531	0.671
$\alpha_5\beta_1$	10c	2.76E-6	25354.351	0.069
$\alpha v\beta_5$	10c	4.13E-6	23357.393	0.096

Q. Structural characterization of amino acids, peptides, cyclic peptides and others

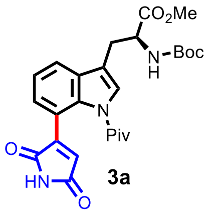

3a

According to the general procedure D, the crude residue was purified by PTLC (ethyl acetate: petroleum ether= 1:3; R_f =0.2) to yield compound **3a** (82.6mg, 83% yield). **^{1}H NMR** (500 MHz, $CDCl_3$) δ 8.13 (s, 1H), 7.61 (d, *J* = 7.5 Hz, 1H), 7.55 (s, 1H), 7.31 (t, *J* = 7.5 Hz, 1H), 7.28 (dd, *J* = 7.0, 1.5 Hz, 1H), 6.45 (d, *J* = 1.5 Hz, 1H), 5.26 (d, *J* = 8.3 Hz, 1H), 4.74 (q, *J* = 6.5 Hz, 1H), 3.71 (s, 3H), 3.25 (ddd, *J* = 52.0, 15.2, 5.9 Hz, 2H), 1.45 (s, 9H), 1.44 (s, 9H). **^{13}C NMR** (126 MHz, $CDCl_3$) δ 178.66, 172.32, 171.21, 170.77, 155.13, 150.32, 133.55, 131.54, 127.52, 124.78, 123.37, 121.98, 121.29, 117.48, 115.23, 80.21, 53.30, 52.46, 41.27, 28.39, 28.31, 27.66. **HRMS** (ESI) *m/z* calcd for $C_{26}H_{31}N_3O_7Na$ $(M + Na)^+$ 520.2054, found 520.2049.

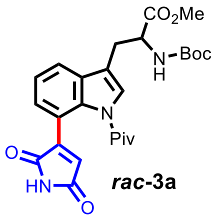

***rac*-3a**

According to the general procedure D, the crude residue was purified by PTLC (ethyl acetate: petroleum ether= 1:3; R_f =0.2) to yield compound ***rac*-3a** (80.5 mg, 81% yield). **^{1}H NMR** (500 MHz, DMSO) δ 10.98 – 10.68 (m, 1H), 7.87 (s, 1H), 7.71 (dd, *J* = 6.7, 2.4 Hz, 1H), 7.45 (d, *J* = 8.1 Hz, 1H), 7.40 – 7.23 (m, 2H), 6.72 (d, *J* = 1.5 Hz, 1H), 4.35 (ddd, *J* = 10.2, 8.1, 4.6 Hz, 1H), 3.66 (s, 3H), 3.30 – 2.90 (m, 2H), 1.41 (s, 9H), 1.33 (s, 9H). **^{13}C NMR** (126 MHz, DMSO) δ 178.89, 172.98, 172.93, 172.27, 155.84, 149.37, 133.33, 131.21, 127.48, 125.95, 123.55, 123.11, 121.20, 118.01, 116.05, 78.79, 55.33, 53.73, 52.40, 41.37, 28.57, 28.25, 26.37. **HRMS** (ESI) *m/z* calcd for $C_{26}H_{31}N_3O_7Na$ $(M + Na)^+$ 520.2054, found 520.2053.

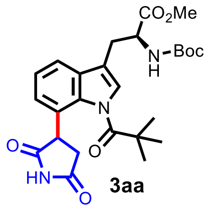

3aa

The crude residue was purified by PTLC (ethyl acetate: petroleum ether= 1:4; R_f =0.2) to yield compound **3aa**. **^{1}H NMR** (400 MHz, $CDCl_3$) δ 8.60 (s, 1H), 7.52 (s, 1H), 7.48 (d, *J* = 7.6 Hz, 1H), 7.31 (t, *J* = 7.6 Hz, 1H), 7.17 (d, *J* = 7.5 Hz, 1H), 5.19 (d, *J* = 8.2 Hz, 1H), 4.74 (q, *J* = 6.4 Hz, 1H), 4.61 (s, 1H), 3.73 (s, 3H), 3.40 (dd, *J* = 17.7, 9.6 Hz, 1H), 3.33 – 3.13 (m, 2H), 3.04 (d, *J* = 18.4 Hz, 1H), 1.53 (s, 9H), 1.45 (s, 9H). **^{13}C NMR** (101 MHz, $CDCl_3$) δ 179.06, 178.65, 176.96, 172.38, 172.35, 155.13, 132.44, 124.77, 124.15, 118.71, 115.63, 80.21, 53.48, 53.21, 52.55, 41.63, 38.42, 38.34, 28.99, 28.35, 27.65. **HRMS** (ESI) *m/z* calcd for $C_{26}H_{33}N_3O_7Na$ $(M + Na)^+$ 522.2211, found 522.2219.

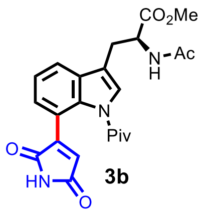

3b

According to the general procedure D, the crude residue was purified by flash column chromatography on silica gel (ethyl acetate: petroleum ether= 2:1; R_f =0.4) to yield compound **3b** (71.1 mg, 81% yield). **^{1}H NMR** (600 MHz, $CDCl_3$) δ 7.94 (s, 1H), 7.61 (dd, *J* = 7.7, 1.3 Hz, 1H), 7.54 (s, 1H), 7.33 (t, *J* = 7.6 Hz, 1H), 7.29 (d, *J* = 7.8 Hz, 1H), 6.46 (d, *J* = 1.4 Hz, 1H), 6.28 (d, *J* = 7.9 Hz, 1H), 5.05 (dt, *J* = 7.9, 5.7 Hz, 1H), 3.75 (s, 3H), 3.30 (ddd, *J* = 55.3, 15.2, 5.6 Hz, 2H), 2.01 (s, 3H), 1.46 (s, 9H). **^{13}C NMR** (151 MHz, $CDCl_3$) δ 178.73, 172.22, 171.08, 170.70, 169.99, 150.29, 133.54, 131.54, 127.62, 124.75, 123.47, 122.01, 121.22, 117.51, 115.10, 52.64, 52.14, 41.29, 28.36, 27.40, 27.11, 23.16. **HRMS** (ESI) *m/z* calcd for $C_{23}H_{25}N_3O_6Na$ $(M + Na)^+$ 462.1636, found 462.1640.

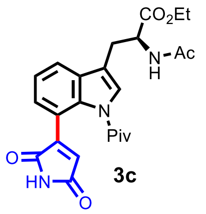

3c

According to the general procedure D, the crude residue was purified by PTLC (ethyl acetate: petroleum ether= 2:1; R_f =0.4) to yield compound **3c** (68.6mg, 78% yield). **^{1}H NMR** (600 MHz, $CDCl_3$) δ 8.15 (s, 1H), 7.62 (dd, *J* = 7.7, 1.3 Hz, 1H), 7.55 (s, 1H), 7.32 (t, *J* = 7.6 Hz, 1H), 7.28 (dd, *J* = 7.4, 1.2 Hz, 1H), 6.44 (d, *J* = 1.5 Hz, 1H), 6.37 (d, *J* = 7.9 Hz, 1H), 5.02 (dt, *J* = 8.2, 5.9 Hz, 1H), 4.29 – 4.06 (m, 2H), 3.28 (ddd, *J* = 50.3, 15.1, 5.9 Hz, 2H), 2.00 (s, 3H), 1.45 (s, 9H), 1.24 (t, *J* = 7.1 Hz, 3H). **^{13}C NMR** (151 MHz, $CDCl_3$) δ 178.76, 171.89, 171.31, 170.84, 170.14, 150.24, 133.53, 131.55, 127.59, 124.71, 123.42, 122.01, 121.24, 117.50, 115.20, 61.92, 52.15, 41.27, 28.33, 27.45, 23.12, 14.06. **HRMS** (ESI) *m/z* calcd for $C_{24}H_{27}N_3O_6Na$ $(M + Na)^+$ 476.1792, found 476.1796.

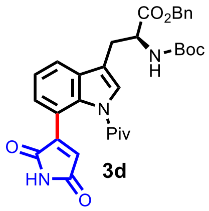

3d

According to the general procedure D, the crude residue was purified by PTLC (ethyl acetate: petroleum ether= 1:3; R_f =0.3) to yield compound **3d** (86.9 mg, 76% yield). **^{1}H NMR** (500 MHz, $CDCl_3$) δ 7.77 – 7.57 (m, 2H), 7.54 (s, 1H), 7.34 (dd, *J* = 5.2, 1.9 Hz, 3H), 7.31 – 7.13 (m, 4H), 6.47 (d, *J* = 1.6 Hz, 1H), 5.31 – 5.01 (m, 3H), 4.79 (q, *J* = 6.6 Hz, 1H), 3.28 (ddd, *J* = 55.8, 15.1, 5.9 Hz, 2H), 1.44 (s, 9H), 1.43 (s, 9H). **^{13}C NMR** (151 MHz, $CDCl_3$) δ 178.69, 171.77, 171.23, 170.75, 155.17, 150.36, 135.00, 134.96, 133.52, 131.58, 128.68, 128.53, 128.10, 127.55, 124.81, 123.41, 121.97, 121.36, 117.46, 115.19, 80.25, 67.37, 53.38, 41.26, 28.39, 28.35, 27.59. **HRMS** (ESI) *m/z* calcd for $C_{32}H_{35}N_3O_7Na$ $(M + Na)^+$ 596.2367, found 596.2368.

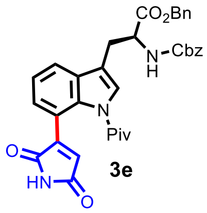

3e

According to the general procedure D, the crude residue was purified by PTLC (ethyl acetate: petroleum ether= 1:2; R_f =0.3) to yield compound **3e** (98.1 mg, 81% yield). **^{1}H NMR** (500 MHz, $CDCl_3$) δ 7.95 (d, *J* = 1.7 Hz, 1H), 7.57 (dd, *J* = 6.5, 2.6 Hz, 1H), 7.54 (s, 1H), 7.44 – 7.28 (m, 8H), 7.28 – 7.13 (m, 4H), 6.46 (d, *J* = 1.6 Hz, 1H), 5.59 (d, *J* = 8.2 Hz, 1H), 5.33 – 5.01 (m, 4H), 4.88 (dt, *J* = 8.2, 5.9 Hz, 1H), 3.62 – 2.97 (m, 2H), 1.40 (s, 9H). **^{13}C NMR** (126 MHz, $CDCl_3$) δ 178.64, 171.45, 171.14, 170.66, 155.78, 150.32, 136.06, 134.82, 133.53, 131.44, 128.70, 128.59, 128.56, 128.26, 128.13, 128.12, 127.58, 124.84, 123.44, 121.99, 121.25, 117.49, 114.91, 67.51, 67.15, 53.80, 41.24, 28.33, 27.73. **HRMS** (ESI) *m/z* calcd for $C_{35}H_{33}N_3O_7Na$ $(M + Na)^+$ 630.2211, found 630.2224.

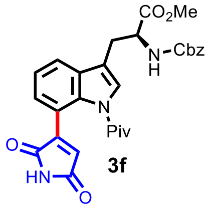

3f

According to the general procedure D, the crude residue was purified by PTLC (ethyl acetate: petroleum ether= 2:3; R_f =0.35) to yield compound **3f** (88.1 mg, 83% yield). **^{1}H NMR** (600 MHz, $CDCl_3$) δ 8.17 (s, 1H), 7.60 (dd, *J* = 6.6, 2.5 Hz, 1H), 7.57 (s, 1H), 7.34 (q, *J* = 5.1 Hz, 5H), 7.28 (q, *J* = 5.0, 4.6 Hz, 2H), 6.45 (d, *J* = 1.5 Hz, 1H), 5.63 (d, *J* = 8.2 Hz, 1H), 5.14 (q, *J* = 12.2 Hz, 2H), 4.82 (q, *J* = 6.5 Hz, 1H), 3.72 (s, 3H), 3.29 (ddd, *J* = 52.4, 15.1, 5.6 Hz, 2H), 1.43 (s, 9H). **^{13}C NMR** (151 MHz, $CDCl_3$) δ 178.70, 172.09, 171.34, 170.86, 155.82, 150.27, 136.09, 133.55, 131.42, 128.55, 128.25, 128.13, 127.60, 124.84, 123.45, 122.02, 121.23, 117.51, 115.01, 67.13, 53.72, 52.63, 41.26, 28.35, 27.74. **HRMS** (ESI) *m/z* calcd for $C_{29}H_{29}N_3O_7Na$ $(M + Na)^+$ 554.1898, found 554.1897.

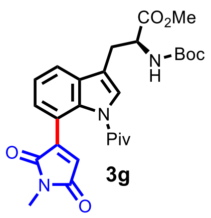

3g

According to the general procedure D, the crude residue was purified by PTLC (ethyl acetate: petroleum ether= 1:3; R_f =0.4) to yield compound **3g** (66.2 mg, 65% yield). **^{1}H NMR** (600 MHz, DMSO) δ 7.88 (s, 1H), 7.72 (dd, J = 6.8, 2.2 Hz, 1H), 7.43 (d, J = 8.1 Hz, 1H), 7.41 – 7.34 (m, 2H), 6.86 (s, 1H), 4.35 (ddd, J = 10.2, 8.1, 4.6 Hz, 1H), 3.66 (s, 3H), 3.23 – 2.99 (m, 2H), 2.90 (s, 3H), 1.40 (s, 9H), 1.32 (s, 9H). **^{13}C NMR** (151 MHz, DMSO) δ 179.03, 172.99, 171.55, 170.87, 155.84, 148.95, 135.06, 133.25, 131.28, 127.47, 126.05, 123.66, 122.30, 121.43, 117.86, 116.17, 78.78, 66.16, 53.71, 52.42, 41.31, 28.57, 28.25, 27.39, 26.34, 23.85. **HRMS** (ESI) m/z calcd for $C_{27}H_{33}N_3O_7Na$ $(M + Na)^+$ 534.2211, found 534.2201.

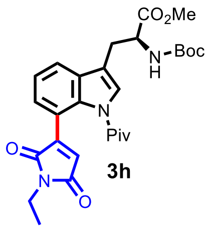

3h

According to the general procedure D, the crude residue was purified by PTLC (ethyl acetate: petroleum ether= 1:3; R_f =0.35) to yield compound **3h** (72.3 mg, 69% yield). **^{1}H NMR** (600 MHz, DMSO) δ 7.90 (s, 1H), 7.72 (dd, J = 6.6, 2.4 Hz, 1H), 7.44 (d, J = 8.1 Hz, 1H), 7.40 – 7.34 (m, 2H), 6.83 (s, 1H), 4.35 (ddd, J = 10.3, 8.1, 4.6 Hz, 1H), 3.66 (s, 3H), 3.45 (q, J = 7.2 Hz, 2H), 3.23 – 2.99 (m, 2H), 1.40 (s, 9H), 1.32 (s, 9H), 1.12 (t, J = 7.2 Hz, 3H). **^{13}C NMR** (151 MHz, DMSO) δ 178.69, 172.98, 171.41, 170.54, 155.84, 149.07, 133.26, 131.31, 127.71, 126.12, 123.72, 122.07, 121.38, 118.01, 116.28, 78.79, 53.70, 52.42, 41.30, 32.60, 28.57, 28.26, 27.38, 26.33, 14.27. **HRMS** (ESI) m/z calcd for $C_{28}H_{35}N_3O_7Na$ $(M + Na)^+$ 548.2367, found 548.2369.

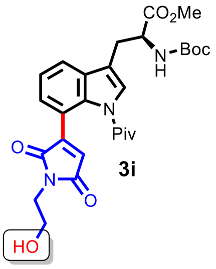

3i

According to the general procedure D, the crude residue was purified by PTLC (ethyl acetate: petroleum ether= 1:3; R_f =0.35) to yield compound **3i** (59.5 mg, 55% yield). **^{1}H NMR** (600 MHz, $CDCl_3$) δ 7.63 (d, J = 7.7 Hz, 1H), 7.58 (s, 1H), 7.34 (t, J = 7.6 Hz, 1H), 7.30 (d, J = 7.1 Hz, 1H), 6.51 (s, 1H), 5.18 (d, J = 8.2 Hz, 1H), 4.74 (q, J = 6.6 Hz, 1H), 3.83 (t, J = 5.1 Hz, 2H), 3.76 (dd, J = 5.8, 4.6 Hz, 2H), 3.73 (s, 3H), 3.27 (ddd, J = 62.3, 15.3, 5.6 Hz, 2H), 1.47 (s, 9H), 1.46 (s, 9H). **^{13}C NMR** (151 MHz, $CDCl_3$) δ 178.52, 172.24, 171.67, 171.10, 155.09, 149.83, 134.21, 133.57, 131.63, 127.65, 124.84, 123.54, 121.33, 121.00, 117.63, 115.47, 80.21, 61.13, 60.83, 53.27, 52.50, 41.25, 40.99, 40.67, 28.47, 28.33, 27.71. **HRMS** (ESI) m/z calcd for $C_{28}H_{35}N_3O_8Na$ $(M + Na)^+$ 564.2316, found 564.2317.

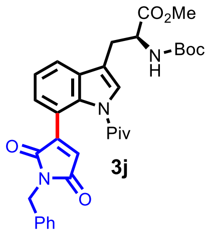

3j

According to the general procedure D, the crude residue was purified by PTLC (ethyl acetate: petroleum ether= 2:3; R_f =0.4) to yield compound **3j** (83.3 mg, 71% yield). **^{1}H NMR** (600 MHz, $CDCl_3$) δ 7.62 (d, J = 7.5 Hz, 1H), 7.56 (s, 1H), 7.43 – 7.37 (m, 2H), 7.36 – 7.29 (m, 4H), 7.28 – 7.21 (m, 1H), 6.52 (s, 1H), 5.17 (d, J = 7.6 Hz, 1H), 4.88 – 4.72 (m, 1H), 4.71 (s, 2H), 3.73 (s, 3H), 3.27 (ddd, J = 58.0, 15.3, 5.5 Hz, 2H), 1.46 (s, 9H), 1.37 (s, 9H). **^{13}C NMR** (151 MHz, $CDCl_3$) δ 178.33, 172.25, 170.95, 170.30, 155.08, 149.68, 136.42, 133.63, 131.59, 128.56, 128.21, 127.67, 127.58, 124.81, 123.42, 121.21, 120.96, 117.86, 115.23, 80.17, 53.30, 52.48, 41.40, 41.12, 28.38, 28.34, 27.70. **HRMS** (ESI) m/z calcd for $C_{33}H_{37}N_3O_7Na$ $(M + Na)^+$ 610.2524, found 610.2534.

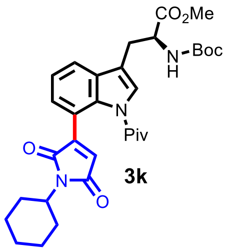
3k

According to the general procedure D, the crude residue was purified by PTLC (ethyl acetate: petroleum ether= 1:4; R_f =0.3) to yield compound **3k** (84.6 mg, 73% yield). **^{1}H NMR** (600 MHz, $CDCl_3$) δ 7.61 (d, J = 7.6 Hz, 1H), 7.57 (s, 1H), 7.32 (t, J = 7.6 Hz, 1H), 7.28 (dd, J = 7.4, 1.3 Hz, 1H), 6.42 (s, 1H), 5.16 (d, J = 8.3 Hz, 1H), 4.74 (d, J = 7.2 Hz, 1H), 3.93 (tt, J = 12.3, 3.8 Hz, 1H), 3.73 (s, 3H), 3.27 (ddd, J = 55.3, 15.4, 5.5 Hz, 2H), 2.10 (qd, J = 12.7, 3.6 Hz, 2H), 1.85 (dt, J = 13.3, 3.3 Hz, 2H), 1.79 – 1.70 (m, 2H), 1.66 (d, J = 9.4 Hz, 1H), 1.48 (s, 9H), 1.46 (s, 9H), 1.34 (dt, J = 13.2, 3.4 Hz, 2H), 1.26 (td, J = 9.3, 4.6 Hz, 1H). **^{13}C NMR** (151 MHz, $CDCl_3$) δ 178.23, 172.26, 171.45, 170.56, 155.08, 149.12, 133.68, 131.55, 127.67, 124.75, 123.39, 120.98, 118.10, 115.17, 80.16, 53.31, 52.48, 50.71, 41.20, 30.13, 28.47, 28.34, 27.71, 26.04, 25.15. **HRMS** (ESI) *m/z* calcd for $C_{32}H_{41}N_3O_7Na$ $(M + Na)^+$ 602.2837, found 602.2836.

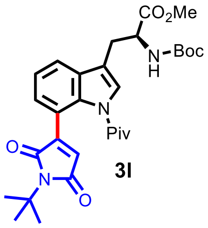
3l

According to the general procedure D, the crude residue was purified by PTLC (ethyl acetate: petroleum ether= 1:5; R_f =0.3) to yield compound **3l** (77.4 mg, 70% yield). **^{1}H NMR** (600 MHz, $CDCl_3$) δ 7.67 – 7.50 (m, 2H), 7.32 (t, J = 7.6 Hz, 1H), 7.26 (dd, J = 7.4, 1.2 Hz, 1H), 6.35 (s, 1H), 5.16 (d, J = 8.1 Hz, 1H), 4.74 (q, J = 6.5 Hz, 1H), 3.73 (s, 3H), 3.26 (ddd, J = 57.1, 15.3, 5.5 Hz, 2H), 1.62 (s, 9H), 1.50 (s, 9H), 1.46 (s, 9H). **^{13}C NMR** (151 MHz, $CDCl_3$) δ 177.72, 172.77, 172.27, 171.61, 155.08, 148.96, 133.82, 131.54, 127.81, 124.76, 123.43, 121.08, 120.84, 118.34, 115.30, 80.16, 57.19, 53.28, 52.48, 41.14, 29.07, 28.52, 28.34, 27.67. **HRMS** (ESI) *m/z* calcd for $C_{30}H_{39}N_3O_7Na$ $(M + Na)^+$ 576.2680, found 576.2692.

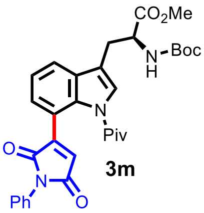
3m

According to the general procedure D, the crude residue was purified by PTLC (ethyl acetate: petroleum ether= 1:4; R_f =0.35) to yield compound **3m** (83.6 mg, 73% yield). **^{1}H NMR** (600 MHz, DMSO) ^{1}H NMR (600 MHz, DMSO) δ 7.91 (s, 1H), 7.76 (dd, J = 7.6, 1.4 Hz, 1H), 7.57 – 7.49 (m, 2H), 7.49 – 7.39 (m, 4H), 7.36 – 7.27 (m, 2H), 7.04 (s, 1H), 4.36 (ddd, J = 10.3, 8.1, 4.7 Hz, 1H), 3.66 (s, 3H), 3.29 – 2.95 (m, 2H), 1.35 (s, 9H), 1.32 (s, 9H). **^{13}C NMR** (151 MHz, DMSO) δ 178.80, 172.98, 170.51, 169.58, 155.84, 149.15, 133.31, 132.19, 131.35, 129.53, 128.42, 127.86, 127.38, 126.16, 123.79, 122.33, 121.58, 117.83, 116.41, 78.79, 53.70, 52.42, 41.29, 28.57, 28.25, 26.34. **HRMS** (ESI) *m/z* calcd for $C_{32}H_{35}N_3O_7Na$ $(M + Na)^+$ 596.2376, found 596.2386.

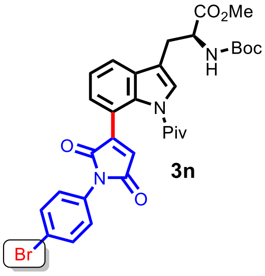
3n

According to the general procedure D, the crude residue was purified by flash column chromatography on silica gel (ethyl acetate: petroleum ether= 2:7; R_f =0.4) to yield compound **3n** (104.0 mg, 80% yield). **^{1}H NMR** (500 MHz, $CDCl_3$) δ 7.65 (dq, J = 7.6, 3.9 Hz, 1H), 7.62 – 7.58 (m, 2H), 7.57 (s, 1H), 7.35 (q, J = 3.8, 2.9 Hz, 2H), 7.32 – 7.23 (m, 2H), 6.62 (s, 1H), 5.20 (d, J = 8.2 Hz, 1H), 4.74 (q, J = 6.5 Hz, 1H), 3.72 (s, 3H), 3.27 (ddd, J = 53.5, 15.3, 5.6 Hz, 2H), 1.45 (s, 9H), 1.40 (s, 9H). **^{13}C NMR** (126 MHz, $CDCl_3$) δ 178.59, 172.22, 169.74, 168.95, 155.08, 149.84, 133.61, 132.21, 131.63, 130.92, 127.86, 127.64, 124.85, 123.52, 121.50, 121.43, 120.96, 117.46, 115.42, 80.18, 53.28, 52.47, 41.21, 28.43, 28.32, 27.73. **HRMS** (ESI) *m/z* calcd for $C_{32}H_{34}BrN_3O_7Na$ $(M + Na)^+$ 674.1472, found 674.1471.

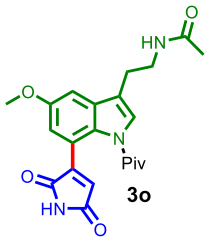

3o

According to the general procedure D, the crude residue was purified by flash column chromatography on silica gel (ethyl acetate: petroleum ether= 1:1; R_f =0.45) to yield compound **3o** (60.0 mg, 73% yield). **^{1}H NMR** (600 MHz, $CDCl_3$) δ 7.60 (s, 1H), 7.53 (s, 1H), 7.12 (d, *J* = 2.6 Hz, 1H), 6.90 (d, *J* = 2.5 Hz, 1H), 6.45 (d, *J* = 1.6 Hz, 1H), 5.76 (t, *J* = 6.2 Hz, 1H), 3.90 (s, 3H), 3.60 (q, *J* = 6.6 Hz, 2H), 2.92 (td, *J* = 6.8, 1.0 Hz, 2H), 1.98 (s, 3H), 1.45 (s, 9H). **^{13}C NMR** (151 MHz, $CDCl_3$) δ 178.33, 170.50, 156.17, 150.22, 132.67, 124.60, 122.06, 118.29, 117.60, 115.82, 103.95, 55.95, 41.16, 38.87, 28.45, 25.22, 23.38. **HRMS** (ESI) *m/z* calcd for $C_{22}H_{25}N_3O_5Na$ $(M + Na)^+$ 434.1686, found 434.1689.

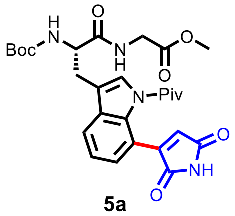

5a

According to the general procedure D, the crude residue was purified by flash column chromatography on silica gel (ethyl acetate: petroleum ether= 1:1; R_f =0.35) to yield compound **5a** (90.0 mg, 81% yield). **^{1}H NMR** (600 MHz, $CDCl_3$) δ 7.73 (d, *J* = 7.6 Hz, 1H), 7.69 (s, 1H), 7.63 (s, 1H), 7.34 (t, *J* = 7.6 Hz, 1H), 7.31 – 7.26 (m, 1H), 6.64 (t, *J* = 5.5 Hz, 1H), 6.46 (d, *J* = 1.5 Hz, 1H), 5.27 (d, *J* = 8.5 Hz, 1H), 4.59 (s, 1H), 3.98 (d, *J* = 5.2 Hz, 2H), 3.73 (s, 3H), 3.24 (d, *J* = 6.3 Hz, 2H), 1.45 (s, 18H). **^{13}C NMR** (151 MHz, $CDCl_3$) δ 178.74, 171.46, 170.92, 169.77, 150.36, 133.58, 131.44, 127.57, 125.32, 123.48, 121.88, 121.47, 117.38, 115.51, 80.61, 54.03, 52.41, 41.30, 41.16, 28.55, 28.35, 28.29, 27.73. **HRMS** (ESI) *m/z* calcd for $C_{28}H_{34}N_4O_8Na$ $(M + Na)^+$ 577.2269, found 577.2270.

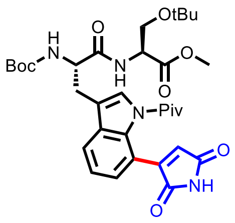

$5b_1$

According to the general procedure D, the crude residue was purified by flash column chromatography on silica gel (DCM: MeOH= 40:1; R_f =0.35) to yield compound **$5b_1$** (79.3 mg, 62% yield). **^{1}H NMR** (500 MHz, $CDCl_3$) δ 7.91 (s, 1H), 7.71 (dd, *J* = 7.8, 1.3 Hz, 1H), 7.67 (s, 1H), 7.31 (t, *J* = 7.6 Hz, 1H), 7.26 (dd, *J* = 7.3, 1.3 Hz, 1H), 6.76 (d, *J* = 8.3 Hz, 1H), 6.43 (d, *J* = 1.1 Hz, 1H), 5.31 (d, *J* = 8.5 Hz, 1H), 4.66 (dt, *J* = 8.3, 3.1 Hz, 2H), 3.79 (dd, *J* = 9.1, 3.0 Hz, 1H), 3.71 (s, 3H), 3.51 (dd, *J* = 9.2, 3.2 Hz, 1H), 3.38 – 3.11 (m, 2H), 1.44 (s, 9H), 1.43 (s, 9H), 1.09 (s, 9H). **^{13}C NMR** (151 MHz, $CDCl_3$) δ 178.78, 171.25, 171.04, 170.64, 170.52, 155.38, 150.42, 133.56, 131.73, 127.38, 125.33, 123.35, 121.89, 121.52, 117.35, 115.43, 80.19, 73.61, 61.68, 53.81, 52.96, 52.44, 41.28, 28.36, 28.32, 27.94, 27.21. **HRMS** (ESI) *m/z* calcd for $C_{33}H_{44}N_4O_9Na$ $(M + Na)^+$ 663.3001, found 663.3003.

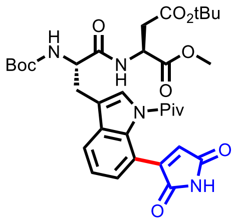

$5b_2$

According to the general procedure D, the crude residue was purified by flash column chromatography on silica gel (ethyl acetate: petroleum ether= 1:2; R_f =0.3) to yield compound **$5b_2$** (109.3mg, 82% yield). **^{1}H NMR** (500 MHz, $CDCl_3$) δ 7.92 (s, 1H), 7.71 (dd, *J* = 7.7, 1.4 Hz, 1H), 7.66 (s, 1H), 7.31 (t, *J* = 7.6 Hz, 1H), 7.28 – 7.23 (m, 1H), 6.99 (d, *J* = 8.4 Hz, 1H), 6.42 (d, *J* = 1.5 Hz, 1H), 5.28 (d, *J* = 8.3 Hz, 1H), 4.79 (dt, *J* = 8.7, 4.5 Hz, 1H), 4.70 – 4.51 (m, 1H), 3.70 (s, 3H), 3.25 (ddd, *J* = 54.4, 15.1, 6.2 Hz, 2H), 2.92 (dd, *J* = 17.0, 4.5 Hz, 1H), 2.66 (dd, *J* = 17.0, 4.6 Hz, 1H), 1.44 (s, 9H), 1.42 (s, 9H), 1.39 (s, 9H). **^{13}C NMR** (126 MHz, $CDCl_3$) δ 178.68, 171.17, 170.98, 170.84, 170.57, 169.88, 155.34, 150.39, 133.59, 131.59, 127.41, 125.32, 123.36, 121.90, 121.48, 117.37, 115.34, 82.00, 80.29, 53.90, 52.69, 48.67, 41.28, 37.27, 28.35, 28.28, 27.94, 27.80. **HRMS** (ESI) *m/z* calcd for $C_{34}H_{44}N_4O_{10}Na$ $(M + Na)^+$ 691.2950, found 691.2950.

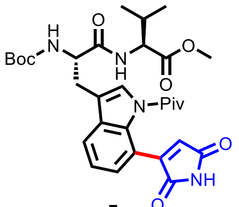

5c$_1$

According to the general procedure D, the crude residue was purified by flash column chromatography on silica gel (DCM: MeOH= 40:1; R$_f$ =0.5) to yield compound **5c₁** (95.3 mg, 80% yield). **^{1}H NMR** (600 MHz, $CDCl_3$) δ 8.05 (s, 1H), 7.70 (d, *J* = 7.7 Hz, 1H), 7.62 (s, 1H), 7.32 (t, *J* = 7.5 Hz, 1H), 7.28 (d, *J* = 7.5 Hz, 1H), 6.65 (d, *J* = 8.6 Hz, 1H), 6.44 (s, 1H), 5.33 (d, *J* = 8.4 Hz, 1H), 4.59 (q, *J* = 7.6 Hz, 1H), 4.48 (dd, *J* = 8.7, 5.0 Hz, 1H), 3.69 (s, 3H), 3.22 (qd, *J* = 15.9, 15.2, 6.5 Hz, 2H), 2.14 (h, *J* = 6.7 Hz, 1H), 1.44 (d, *J* = 3.8 Hz, 18H), 0.87 (dd, *J* = 22.3, 6.8 Hz, 6H). **^{13}C NMR** (151 MHz, $CDCl_3$) δ 178.73, 171.81, 171.29, 171.21, 170.68, 155.67, 150.32, 133.60, 131.46, 127.48, 125.10, 123.43, 121.93, 121.43, 117.41, 115.56, 80.52, 57.26, 54.08, 52.21, 41.28, 31.18, 28.37, 28.30, 27.16, 18.86, 17.64. **HRMS** (ESI) *m/z* calcd for $C_{31}H_{40}N_4O_8Na$ (M + Na)$^+$ 619.2738, found 619.2743.

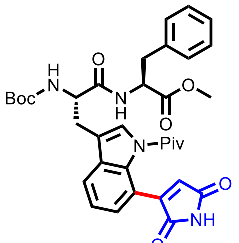

5c$_2$

According to the general procedure D, the crude residue was purified by flash column chromatography on silica gel (ethyl acetate: petroleum ether= 1:2; R$_f$ =0.25) to yield compound **5c₂** (95.4mg, 74% yield). **^{1}H NMR** (500 MHz, $CDCl_3$) δ 8.00 (s, 1H), 7.70 (dd, *J* = 7.8, 1.4 Hz, 1H), 7.61 (s, 1H), 7.32 (t, *J* = 7.6 Hz, 1H), 7.28 (dd, *J* = 7.4, 1.3 Hz, 1H), 7.20 (qd, *J* = 4.8, 1.6 Hz, 3H), 7.03 – 6.89 (m, 2H), 6.50 (d, *J* = 7.6 Hz, 1H), 6.43 (d, *J* = 1.5 Hz, 1H), 5.22 (d, *J* = 8.4 Hz, 1H), 4.76 (d, *J* = 6.6 Hz, 1H), 4.61 – 4.43 (m, 1H), 3.67 (s, 3H), 3.18 (d, *J* = 6.5 Hz, 2H), 3.11 – 2.92 (m, 2H), 1.44 (s, 18H). **^{13}C NMR** (151 MHz, $CDCl_3$) δ 178.74, 171.39, 171.28, 170.81, 170.66, 155.42, 150.30, 135.48, 133.61, 131.41, 129.14, 128.57, 127.50, 127.17, 125.22, 123.45, 121.95, 121.54, 117.44, 115.45, 80.44, 53.92, 53.34, 52.39, 41.30, 37.78, 28.38, 28.31, 27.69. **HRMS** (ESI) *m/z* calcd for $C_{35}H_{40}N_4O_8Na$ (M + Na)$^+$ 667.2738, found 667.2745.

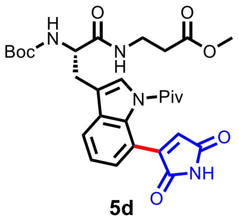

5d

According to the general procedure D, the crude residue was purified by flash column chromatography on silica gel (DCM: MeOH= 40:1; R$_f$ =0.45) to yield compound **5d** (89.7 mg, 79% yield). **^{1}H NMR** (600 MHz, $CDCl_3$) δ 7.77 (s, 1H), 7.70 (d, *J* = 7.7 Hz, 1H), 7.61 (s, 1H), 7.34 (t, *J* = 7.6 Hz, 1H), 7.29 (d, *J* = 9.9 Hz, 1H), 6.59 (t, *J* = 6.1 Hz, 1H), 6.46 (s, 1H), 5.21 (d, *J* = 8.6 Hz, 1H), 4.64 – 4.33 (m, 1H), 3.62 (s, 3H), 3.44 (tt, *J* = 13.7, 7.2 Hz, 2H), 3.21 (q, *J* = 8.8 Hz, 2H), 2.44 (dt, *J* = 17.2, 5.9 Hz, 1H), 2.35 (d, *J* = 16.7 Hz, 1H), 1.46 (s, 9H), 1.44 (s, 9H). **^{13}C NMR** (151 MHz, $CDCl_3$) δ 178.70, 172.55, 171.12, 171.01, 170.54, 155.45, 150.36, 133.57, 131.43, 127.56, 125.04, 123.51, 121.89, 121.47, 117.45, 115.64, 80.47, 54.37, 51.81, 41.31, 34.90, 33.43, 28.38, 28.29, 27.69. **HRMS** (ESI) *m/z* calcd for $C_{29}H_{36}N_4O_8Na$ (M + Na)$^+$ 591.2425, found 591.2429.

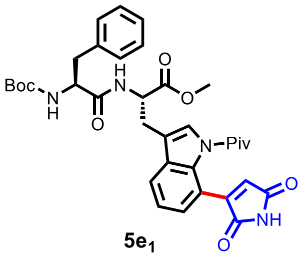

5e$_1$

According to the general procedure D, the crude residue was purified by flash column chromatography on silica gel (ethyl acetate: petroleum ether= 1:2; R$_f$ =0.4) to yield compound **5e₁** (92.7 mg, 72% yield). **^{1}H NMR** (600 MHz, $CDCl_3$) δ 7.60 – 7.52 (m, 2H), 7.37 (s, 1H), 7.31 – 7.28 (m, 2H), 7.26 (dd, *J* = 7.9, 6.2 Hz, 2H), 7.23 – 7.17 (m, 3H), 6.50 (d, *J* = 7.9 Hz, 1H), 6.46 (d, *J* = 1.6 Hz, 1H), 4.97 (dt, *J* = 7.6, 5.7 Hz, 2H), 4.45 – 4.28 (m, 1H), 3.66 (s, 3H), 3.24 (d, *J* = 5.7 Hz, 2H), 3.08 – 3.03 (m, 2H), 1.46 (s, 9H), 1.39 (s, 9H). **^{13}C NMR** (151 MHz, $CDCl_3$) δ 178.72, 171.40, 171.12, 170.69, 170.27, 150.36,

133.55, 131.43, 129.42, 129.24, 128.68, 127.60, 127.07, 124.80, 123.45, 121.95, 121.34, 119.67, 118.50, 117.46, 114.94, 80.34, 52.52, 52.15, 41.29, 38.26, 28.36, 28.19, 27.65. **HRMS** (ESI) *m/z* calcd for $C_{35}H_{40}N_4O_8Na$ (M + Na)$^+$ 667.2738, found 667.2745.

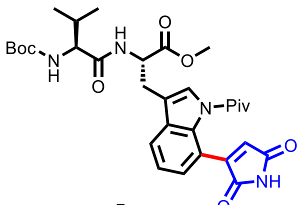

5e$_2$

According to the general procedure D, the crude residue was purified by flash column chromatography on silica gel (ethyl acetate: petroleum ether= 1:2; R_f =0.35) to yield compound **5e$_2$** (93.9 mg, 79% yield). **^{1}H NMR** (600 MHz, DMSO) δ 10.83 (s, 1H), 8.47 (d, *J* = 7.5 Hz, 1H), 7.84 (s, 1H), 7.70 (dd, *J* = 7.1, 2.0 Hz, 1H), 7.43 – 7.31 (m, 2H), 6.71 (s, 1H), 6.47 (d, *J* = 9.1 Hz, 1H), 4.72 (td, *J* = 8.1, 5.2 Hz, 1H), 3.85 (dd, *J* = 9.1, 6.8 Hz, 1H), 3.61 (s, 3H), 3.27 – 3.08 (m, 2H), 1.94 – 1.82 (m, 1H), 1.39 (s, 9H), 1.36 (s, 9H), 0.77 (dd, *J* = 6.8, 5.3 Hz, 6H). **^{13}C NMR** (151 MHz, DMSO) δ 179.01, 172.95, 172.30, 172.27, 172.08, 155.67, 149.35, 135.71, 133.38, 131.13, 127.57, 125.74, 123.56, 123.14, 117.98, 115.64, 78.47, 59.71, 52.39, 51.90, 51.90, 41.34, 31.30, 28.60, 28.27, 26.67, 19.44, 18.42. **HRMS** (ESI) *m/z* calcd for $C_{31}H_{40}N_4O_8Na$ (M + Na)$^+$ 619.2738, found 619.2742.

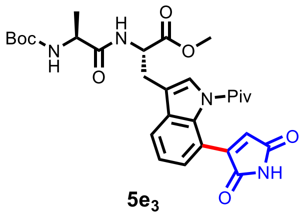

5e$_3$

According to the general procedure D, the crude residue was purified by flash column chromatography on silica gel (ethyl acetate: petroleum ether= 1:1; R_f =0.35) to yield compound **5e$_3$** (90.0 mg, 81% yield). **^{1}H NMR** (600 MHz, DMSO) δ 10.83 (s, 1H), 8.37 (dd, *J* = 7.4, 2.6 Hz, 1H), 7.86 (s, 1H), 7.70 (dd, *J* = 7.1, 1.9 Hz, 1H), 7.43 – 7.32 (m, 2H), 6.79 (d, *J* = 7.7 Hz, 1H), 6.71 (s, 1H), 4.69 (td, *J* = 7.9, 5.5 Hz, 1H), 4.04 (qt, *J* = 7.0, 4.0 Hz, 1H), 3.61 (s, 3H), 3.27 – 3.10 (m, 2H), 1.40 (s, 9H), 1.36 (s, 9H), 1.15 (d, *J* = 7.1 Hz, 3H). **^{13}C NMR** (151 MHz, DMSO) δ 179.05, 173.53, 172.95, 172.28, 155.35, 149.37, 136.73, 133.37, 131.22, 127.53, 125.89, 123.52, 123.15, 121.27, 117.97, 115.56, 99.99, 78.45, 52.45, 52.09, 49.89, 41.36, 28.63, 28.53, 28.30, 26.74, 18.71. **HRMS** (ESI) *m/z* calcd for $C_{29}H_{36}N_4O_8Na$ (M + Na)$^+$ 591.2425, found 591.2423.

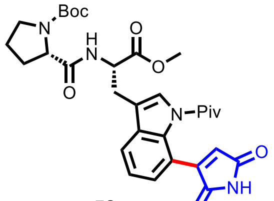

5f

According to the general procedure D, the crude residue was purified by flash column chromatography on silica gel (ethyl acetate: petroleum ether= 1:1; R_f =0.35) to yield compound **5f** (77.2 mg, 65% yield). **^{1}H NMR** (600 MHz, $CDCl_3$) δ 7.79 – 7.59 (m, 2H), 7.54 (t, *J* = 3.9 Hz, 1H), 7.45 – 7.22 (m, 3H), 6.46 (s, 1H), 4.96 (d, *J* = 60.6 Hz, 1H), 4.37 – 4.11 (m, 1H), 3.71 (s, 3H), 3.43 – 3.18 (m, 4H), 2.16 – 1.91 (m, 2H), 1.78 (d, *J* = 42.3 Hz, 2H), 1.48 (s, 9H), 1.40 (s, 9H). **^{13}C NMR** (151 MHz, DMSO) δ 172.95, 149.39, 133.48, 131.05, 127.52, 126.27, 123.52, 123.11, 121.12, 118.03, 111.87, 78.83, 60.05, 52.43, 52.14, 46.84, 41.38, 31.33, 28.58, 28.23, 28.16, 26.65, 23.48. **HRMS** (ESI) *m/z* calcd for $C_{31}H_{38}N_4O_8Na$ (M + Na)$^+$ 617.2582, found 617.2583.

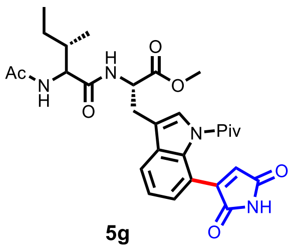

5g

According to the general procedure D, the crude residue was purified by flash column chromatography on silica gel (DCM: MeOH= 30:1; R_f =0.45) to yield compound **5g** (86.1 mg, 78% yield). **^{1}H NMR** (600 MHz, $CDCl_3$) δ 7.84 (s, 1H), 7.64 (dd, *J* = 7.8, 1.2 Hz, 1H), 7.61 (s, 1H), 7.34 (t, *J* = 7.6 Hz, 1H), 7.31 – 7.27 (m, 1H), 6.77 (d, *J* = 7.9 Hz, 1H), 6.44 (d, *J* = 1.5 Hz, 1H), 6.20 (d, *J* = 8.7 Hz, 1H), 5.02 (dt, *J* = 7.9, 5.9 Hz, 1H), 4.31 (dd, *J* =

8.6, 7.2 Hz, 1H), 3.72 (s, 3H), 3.28 (dd, *J* = 6.0, 3.6 Hz, 2H), 1.96 (s, 3H), 1.88 – 1.74 (m, 2H), 1.46 (s, 9H), 1.15 (dddd, *J* = 16.7, 14.2, 8.3, 4.9 Hz, 1H), 0.93 (d, *J* = 6.8 Hz, 3H), 0.90 (t, *J* = 7.4 Hz, 3H). **^{13}C NMR** (151 MHz, $CDCl_3$) δ 178.77, 171.66, 171.38, 171.11, 170.57, 169.98, 150.25, 133.58, 131.33, 127.62, 124.86, 123.56, 122.03, 121.29, 117.48, 114.92, 57.72, 52.56, 52.01, 41.29, 37.48, 28.34, 27.55, 25.01, 23.10, 15.23, 11.24. **HRMS** (ESI) *m/z* calcd for $C_{29}H_{36}N_4O_7Na$ (M + Na)$^+$ 575.2476, found 575.2484.

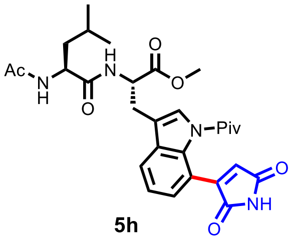

5h

According to the general procedure D, the crude residue was purified by flash column chromatography on silica gel (DCM: MeOH= 30:1; R_f =0.45) to yield compound **5h** (88.3 mg, 80% yield). **^{1}H NMR** (600 MHz, $CDCl_3$) δ 8.52 (s, 1H), 7.67 – 7.59 (m, 2H), 7.31 (t, *J* = 7.6 Hz, 1H), 7.25 (dd, *J* = 7.4, 1.2 Hz, 1H), 7.16 (d, *J* = 7.8 Hz, 1H), 6.39 (d, *J* = 1.4 Hz, 1H), 6.35 (d, *J* = 8.2 Hz, 1H), 4.98 (dt, *J* = 7.9, 6.2 Hz, 1H), 4.50 (td, *J* = 8.5, 5.3 Hz, 1H), 3.70 (s, 3H), 3.26 (qd, *J* = 15.1, 6.0 Hz, 2H), 1.88 (s, 3H), 1.64 (dtd, *J* = 13.7, 8.0, 6.5, 3.6 Hz, 2H), 1.54 – 1.47 (m, 1H), 1.44 (s, 9H), 0.91 (dd, *J* = 14.2, 6.1 Hz, 6H). **^{13}C NMR** (151 MHz, $CDCl_3$) δ 178.79, 172.50, 171.76, 171.67, 170.93, 170.36, 150.15, 133.57, 131.36, 127.51, 124.93, 123.46, 122.03, 121.30, 117.47, 114.98, 52.54, 52.08, 51.75, 41.28, 41.14, 28.33, 27.44, 24.70, 22.84, 22.82, 22.10. **HRMS** (ESI) *m/z* calcd for $C_{29}H_{36}N_4O_7Na$ (M + Na)$^+$ 575.2476, found 575.2483.

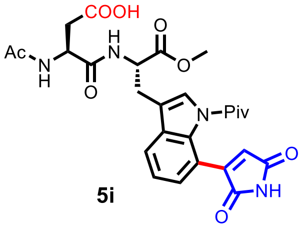

5i

According to the general procedure D, the crude residue was purified by flash column chromatography on silica gel (DCM: MeOH= 8:1; R_f =0.4) to yield compound **5i** (42.1 mg, 38% yield). **^{1}H NMR** (500 MHz, DMSO) δ 10.85 (s, 1H), 8.49 (d, *J* = 7.4 Hz, 1H), 8.09 (d, *J* = 8.0 Hz, 1H), 7.86 (s, 1H), 7.70 (dd, *J* = 7.1, 1.9 Hz, 1H), 7.42 – 7.30 (m, 2H), 6.72 (s, 1H), 4.62 (dtd, *J* = 15.8, 8.1, 5.3 Hz, 2H), 3.61 (s, 3H), 3.21 (dd, *J* = 15.1, 5.2 Hz, 1H), 3.13 (dd, *J* = 15.1, 8.7 Hz, 1H), 2.60 (dd, *J* = 16.3, 5.3 Hz, 1H), 2.42 (dd, *J* = 16.3, 8.4 Hz, 1H), 1.77 (s, 3H), 1.39 (s, 9H). **^{13}C NMR** (126 MHz, DMSO) δ 179.04, 172.95, 172.28, 172.19, 171.73, 169.58, 149.36, 133.39, 131.18, 127.53, 125.85, 123.57, 123.16, 121.22, 117.99, 115.73, 63.26, 55.36, 52.46, 49.71, 41.35, 36.97, 28.28, 26.48, 22.93. **HRMS** (ESI) *m/z* calcd for $C_{27}H_{30}N_4O_9Na$ (M + Na)$^+$ 577.1905, found 577.1906.

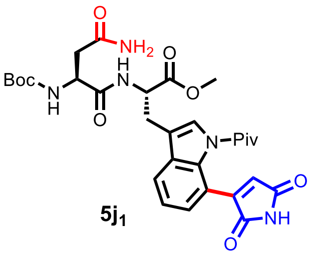

5j$_1$

According to the general procedure D, the crude residue was purified by flash column chromatography on silica gel (DCM: MeOH= 20:1; R_f =0.45) to yield compound **5j$_1$** (57.4 mg, 47% yield). **^{1}H NMR** (500 MHz, Chloroform-*d*) δ 7.86 (s, 1H), 7.68 – 7.64 (m, 2H), 7.60 (d, *J* = 7.9 Hz, 1H), 7.34 (t, *J* = 7.6 Hz, 1H), 7.28 – 7.25 (m, 1H), 6.43 (d, *J* = 1.5 Hz, 1H), 6.03 (d, *J* = 8.1 Hz, 1H), 5.88 (s, 1H), 5.69 (s, 1H), 4.99 (q, *J* = 6.7 Hz, 1H), 4.46 (s, 1H), 3.69 (s, 3H), 3.26 (tt, *J* = 14.7, 7.2 Hz, 2H), 2.87 (d, *J* = 17.3 Hz, 1H), 2.51 (dd, *J* = 15.8, 6.0 Hz, 1H), 1.46 (s, 9H), 1.41 (s, 9H). **^{13}C NMR** (126 MHz, $CDCl_3$) δ 178.87, 171.57, 171.16, 170.98, 170.74, 166.40, 155.60, 150.36, 133.57, 131.36, 127.49, 125.43, 125.18, 123.42, 121.85, 121.41, 117.37, 114.96, 80.48, 52.57, 52.05, 41.30, 28.58, 28.36, 28.20, 27.62. **HRMS** (ESI) *m/z* calcd for $C_{30}H_{37}N_5O_9Na$ (M + Na)$^+$ 634.2483, found 634.2479.

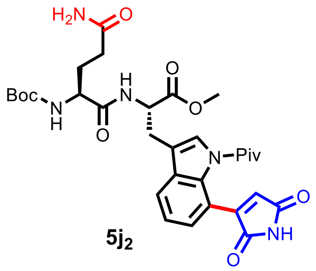

5j2

According to the general procedure D, the crude residue was purified by flash column chromatography on silica gel (DCM: MeOH= 20:1; R_f =0.45) to yield compound **5j2** (88.7 mg, 71% yield). **^{1}H NMR** (500 MHz, Chloroform-*d*) δ 8.26 (s, 1H), 7.94 (d, *J* = 8.0 Hz, 1H), 7.68 (s, 1H), 7.61 (d, *J* = 7.8 Hz, 1H), 7.32 (t, *J* = 7.6 Hz, 1H), 7.26 (d, *J* = 7.3 Hz, 1H), 6.41 (s, 1H), 6.37 (s, 1H), 6.02 (s, 1H), 5.72 (d, *J* = 7.5 Hz, 1H), 4.99 (q, *J* = 7.1 Hz, 1H), 4.21 (d, *J* = 7.3 Hz, 1H), 3.74 (s, 3H), 3.24 (ddd, *J* = 57.8, 15.2, 6.3 Hz, 2H), 2.27 (dd, *J* = 24.7, 6.1 Hz, 2H), 1.97 (dq, *J* = 20.4, 7.3, 6.6 Hz, 2H), 1.43 (s, 9H), 1.41 (s, 9H). **^{13}C NMR** (126 MHz, $CDCl_3$) δ 178.82, 175.55, 172.36, 171.29, 170.99, 155.75, 150.22, 133.55, 131.30, 127.50, 125.42, 125.05, 123.45, 121.91, 121.19, 117.45, 80.05, 53.43, 52.65, 51.80, 41.28, 41.17, 31.51, 28.56, 28.33, 28.28, 27.27. **HRMS** (ESI) *m/z* calcd for $C_{31}H_{39}N_5O_9Na$ $(M + Na)^+$ 648.2640, found 648.2647.

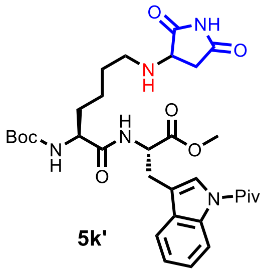

5k'

According to the general procedure D, the crude residue was purified by flash column chromatography on silica gel (DCM: MeOH= 20:1; R_f =0.5) to yield compound **5k'** (62.7 mg, 50% yield). **^{1}H NMR** (500 MHz, Chloroform-*d*) δ 8.48 (dd, *J* = 8.3, 2.4 Hz, 1H), 7.65 (d, *J* = 3.4 Hz, 1H), 7.47 (dd, *J* = 8.9, 3.4 Hz, 1H), 7.35 (t, *J* = 7.7 Hz, 1H), 7.30 – 7.25 (m, 1H), 6.97 (d, *J* = 7.8 Hz, 1H), 5.24 – 4.86 (m, 2H), 4.07 (s, 1H), 3.78 – 3.53 (m, 5H), 3.27 (d, *J* = 5.6 Hz, 2H), 2.84 (ddd, *J* = 18.1, 8.5, 1.8 Hz, 1H), 2.72 – 2.60 (m, 1H), 2.60 – 2.41 (m, 2H), 1.84 – 1.65 (m, 2H), 1.64 – 1.54 (m, 2H), 1.51 (s, 9H), 1.49 – 1.41 (m, 2H), 1.40 (d, *J* = 1.7 Hz, 9H). **^{13}C NMR** (126 MHz, $CDCl_3$) δ 177.02, 175.53, 172.04, 172.01, 171.98, 155.51, 136.95, 135.56, 129.39, 125.47, 123.95, 123.51, 118.30, 117.45, 115.70, 80.11, 57.53, 57.47, 54.38, 53.43, 52.58, 52.37, 51.95, 50.11, 46.97, 46.82, 41.19, 37.87, 37.09, 36.98, 32.18, 29.69, 29.20, 29.14, 28.60, 28.24, 27.58, 22.78. **HRMS** (ESI) *m/z* calcd for $C_{32}H_{45}N_5O_8Na$ $(M + Na)^+$ 650.3160, found 650.3143.

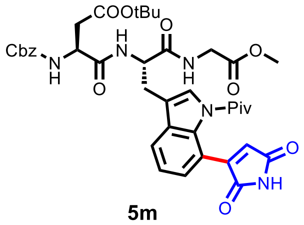

5m

According to the general procedure D, the crude residue was purified by flash column chromatography on silica gel (DCM: MeOH= 35:1; R_f =0.4) to yield compound **5m** (103.2 mg, 68% yield). **^{1}H NMR** (600 MHz, $CDCl_3$) δ 7.79 (d, *J* = 7.8 Hz, 1H), 7.68 (s, 1H), 7.57 (s, 1H), 7.39 – 7.30 (m, 6H), 7.27 – 7.21 (m, 2H), 6.80 (s, 1H), 6.40 (s, 1H), 5.81 (d, *J* = 8.3 Hz, 1H), 5.10 (d, *J* = 12.1 Hz, 1H), 5.02 (d, *J* = 12.3 Hz, 1H), 4.85 (q, *J* = 6.9 Hz, 1H), 4.49 (q, *J* = 6.5 Hz, 1H), 4.03 – 3.84 (m, 2H), 3.70 (s, 3H), 3.26 (qd, *J* = 14.8, 6.5 Hz, 2H), 2.87 (dd, *J* = 17.0, 4.8 Hz, 1H), 2.72 (dd, *J* = 17.0, 6.6 Hz, 1H), 1.44 (s, 9H), 1.41 (s, 9H). **^{13}C NMR** (151 MHz, $CDCl_3$) δ 178.73, 170.88, 170.80, 170.66, 170.50, 169.71, 162.60, 150.32, 135.94, 133.63, 131.31, 128.56, 128.30, 128.12, 127.57, 125.56, 123.52, 121.84, 121.63, 117.38, 115.36, 82.16, 67.34, 52.96, 52.33, 51.51, 41.29, 41.13, 37.31, 36.50, 31.46, 28.32, 27.96, 27.18. **HRMS** (ESI) *m/z* calcd for $C_{39}H_{45}N_5O_{11}Na$ $(M + Na)^+$ 782.3008, found 782.3021.

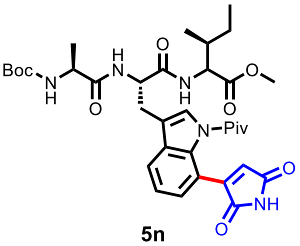

5n

According to the general procedure D, the crude residue was purified by flash column chromatography on silica gel (DCM: MeOH= 30:1; R_f =0.45) to yield compound **5n** (99.4 mg, 73% yield). **^{1}H NMR** (400 MHz, $CDCl_3$) δ 7.88 (s, 1H), 7.77 (dd, *J* = 7.7, 1.3 Hz, 1H), 7.67 (s, 1H), 7.34 (t, *J* = 7.6 Hz, 1H), 7.28 (dd, *J* = 7.6, 1.2 Hz, 1H), 7.10 (d, *J* = 8.0 Hz, 1H), 6.51 (d, *J* = 8.2 Hz, 1H), 6.42 (d, *J* = 1.5 Hz, 1H), 5.03 (d, *J* = 7.2 Hz, 1H), 4.89 (q, *J* = 6.9 Hz, 1H), 4.46 (dd, *J* = 8.2, 4.7 Hz, 1H), 4.26 – 4.12 (m, 1H), 3.68 (s, 3H), 3.30 – 3.18 (m, 2H), 1.81 (ddt, *J* = 9.2, 6.7, 4.6 Hz, 1H), 1.44 (s, 9H), 1.40 (s, 9H), 1.34 (d, *J* = 7.0 Hz, 3H), 1.11 – 0.90 (m, 2H), 0.86 (t, *J* = 7.4 Hz, 3H), 0.80 (d, *J* = 6.9 Hz, 3H).**^{13}C NMR** (101 MHz, $CDCl_3$) δ 178.80, 172.97, 171.62, 171.21, 170.55, 170.33, 150.26, 133.63, 131.30, 127.56, 125.38, 123.51, 121.92, 121.63, 117.42, 115.22, 80.36, 56.76, 52.77, 52.21, 41.32, 37.65, 28.57, 28.33, 28.22, 25.11, 18.29, 15.33, 11.58. **HRMS** (ESI) *m/z* calcd for $C_{35}H_{47}N_5O_9Na$ $(M + Na)^+$ 704.3266, found 704.3267.

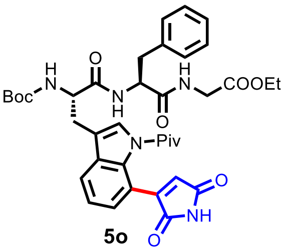

5o

According to the general procedure D, the crude residue was purified by flash column chromatography on silica gel (DCM: MeOH= 40:1; R_f =0.3) to yield compound **5o** (99.8 mg, 70% yield). **^{1}H NMR** (600 MHz, $CDCl_3$) δ 7.91 (d, *J* = 6.2 Hz, 1H), 7.67 (d, *J* = 7.6 Hz, 1H), 7.62 (s, 1H), 7.33 (t, *J* = 7.6 Hz, 1H), 7.28 (d, *J* = 7.0 Hz, 1H), 7.21 (t, *J* = 7.3 Hz, 2H), 7.18 (d, *J* = 7.0 Hz, 1H), 7.07 (d, *J* = 7.3 Hz, 2H), 6.78 (s, 1H), 6.56 (t, *J* = 5.3 Hz, 1H), 6.45 – 6.43 (m, 1H), 5.15 (s, 1H), 4.69 (q, *J* = 7.2 Hz, 1H), 4.52 (s, 1H), 4.18 (q, *J* = 7.1 Hz, 2H), 3.91 (ddd, *J* = 71.8, 18.1, 5.4 Hz, 2H), 3.15 (d, *J* = 6.0 Hz, 2H), 3.01 (ddd, *J* = 49.3, 14.4, 5.7 Hz, 2H), 1.45 (s, 9H), 1.39 (s, 9H), 1.26 (d, *J* = 7.2 Hz, 3H). **^{13}C NMR** (151 MHz, $CDCl_3$) δ 178.69, 171.22, 171.18, 170.70, 170.57, 169.32, 150.26, 136.09, 133.63, 131.32, 129.23, 128.62, 127.57, 127.04, 125.13, 123.50, 121.99, 121.53, 117.48, 115.45, 80.66, 61.50, 54.08, 41.34, 41.31, 37.88, 29.69, 28.38, 28.24, 27.41, 22.69, 14.12. **HRMS** (ESI) *m/z* calcd for $C_{38}H_{45}N_5O_9Na$ $(M + Na)^+$ 738.3109, found 738.3121.

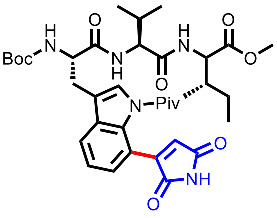

5p

According to the general procedure D, the crude residue was purified by flash column chromatography on silica gel (DCM: MeOH= 30:1; R_f =0.4) to yield compound **5p** (100.2 mg, 71% yield). **^{1}H NMR** (600 MHz, $CDCl_3$) δ 7.81 (s, 1H), 7.70 – 7.66 (m, 1H), 7.65 (s, 1H), 7.32 (t, *J* = 7.6 Hz, 1H), 7.29 – 7.26 (m, 1H), 6.99 (d, *J* = 8.5 Hz, 1H), 6.60 (d, *J* = 8.4 Hz, 1H), 6.44 (s, 1H), 5.29 – 5.19 (m, 1H), 4.65 – 4.56 (m, 2H), 4.38 – 4.32 (m, 1H), 3.75 (s, 3H), 3.30 – 3.12 (m, 2H), 2.13 (dtt, *J* = 13.8, 9.0, 5.4 Hz, 1H), 2.07 – 1.87 (m, 3H), 1.46 (s, 9H), 1.41 (s, 9H), 0.95 – 0.90 (m, 12H). **^{13}C NMR** (151 MHz, $CDCl_3$) δ 178.70, 172.09, 171.52, 171.10, 170.68, 170.50, 150.35, 137.30, 133.62, 131.49, 127.51, 125.59, 124.88, 123.59, 123.42, 121.92, 121.41, 118.65, 117.56, 117.41, 115.74, 80.61, 58.72, 58.47, 56.54, 54.00, 52.13, 52.11, 41.30, 41.25, 37.70, 31.92, 31.04, 30.85, 29.69, 29.35, 28.67, 28.43, 28.24, 26.81, 25.22, 22.69, 19.16, 19.06, 18.18, 17.97, 15.47, 14.11, 11.60, 11.57, 8.43. **HRMS** (ESI) *m/z* calcd for $C_{37}H_{51}N_5O_9Na$ $(M + Na)^+$ 732.3579, found 732.3577.

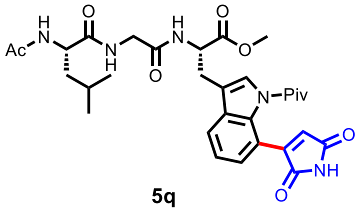

5q

According to the general procedure D, the crude residue was purified by flash column chromatography on silica gel (DCM: MeOH= 25:1; R_f =0.25) to yield compound **5q** (91.0 mg, 75% yield). **^{1}H NMR** (600 MHz, $CDCl_3$) δ 8.82 (dt, *J* = 24.1, 11.3 Hz, 1H), 7.59 (dt, *J* = 8.0, 4.5 Hz, 4H), 7.32 – 7.26 (m, 1H), 7.24 (d, *J* = 7.3 Hz, 1H), 6.86 (dt, *J* = 14.8, 7.0 Hz, 1H), 6.40 (s, 1H), 4.93 (q, *J* = 6.9 Hz, 1H), 4.40 (q, *J* = 7.5 Hz, 1H), 4.05 (dd, *J* = 16.9, 6.3 Hz, 1H), 3.79 (dd, *J* = 16.8, 5.0 Hz, 1H), 3.67 (s, 3H), 3.24 (ddd, *J* = 43.2, 15.4, 6.5 Hz, 2H), 2.74 – 2.58 (m, 1H), 1.84 (s, 3H), 1.59 (ddq, *J* = 27.3, 13.8, 6.7 Hz, 2H), 1.41 (s, 9H), 0.88 (dd, *J* = 13.0, 6.2 Hz, 6H). **^{13}C NMR** (151 MHz, $CDCl_3$) δ 178.84, 173.32, 172.26, 171.95, 171.27, 169.20, 150.16, 133.57, 131.25, 127.48, 124.84, 123.42, 122.02, 121.12, 117.47, 115.32, 52.59, 52.21, 52.06, 42.94, 41.24, 40.62, 28.32, 27.25, 24.69, 22.77, 22.68, 22.04. **HRMS** (ESI) *m/z* calcd for $C_{31}H_{39}N_5O_8Na$ $(M + Na)^+$ 632.2691, found 632.2701.

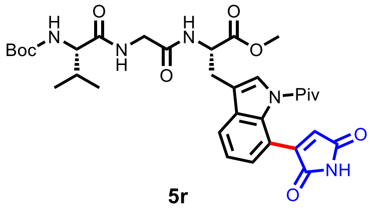

5r

According to the general procedure D, the crude residue was purified by flash column chromatography on silica gel (DCM: MeOH= 30:1; R_f =0.25) to yield compound **5r** (89.8 mg, 69% yield). **^{1}H NMR** (600 MHz, $CDCl_3$) δ 8.12 (dd, *J* = 20.4, 9.3 Hz, 1H), 7.61 (d, *J* = 8.4 Hz, 2H), 7.32 (t, *J* = 7.6 Hz, 1H), 7.27 (d, *J* = 7.3 Hz, 1H), 7.19 (t, *J* = 7.6 Hz, 1H), 7.04 – 6.98 (m, 1H), 6.43 (s, 1H), 5.22 (dd, *J* = 7.9, 4.5 Hz, 1H), 4.96 (q, *J* = 6.6 Hz, 1H), 3.93 (qd, *J* = 18.9, 16.2, 10.2 Hz, 3H), 3.70 (s, 3H), 3.26 (ddd, *J* = 55.4, 15.1, 6.2 Hz, 2H), 2.09 – 2.03 (m, 1H), 1.44 (s, 9H), 1.43 (s, 9H), 0.90 (dd, *J* = 19.9, 6.9 Hz, 6H). **^{13}C NMR** (151 MHz, $CDCl_3$) δ 178.77, 172.47, 171.80, 171.34, 170.77, 168.84, 156.06, 150.22, 133.59, 131.30, 127.57, 124.91, 123.44, 122.00, 121.16, 117.52, 115.03, 80.13, 60.10, 52.59, 52.26, 43.08, 41.28, 31.92, 30.77, 29.68, 29.35, 28.34, 28.31, 27.45, 22.68, 19.22, 17.80, 14.11. **HRMS** (ESI) *m/z* calcd for $C_{33}H_{43}N_5O_9Na$ $(M + Na)^+$ 676.2953, found 676.2952.

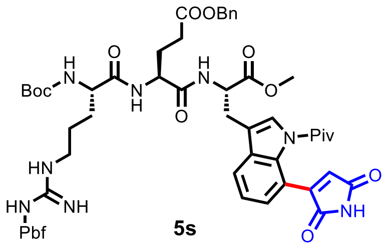

5s

According to the general procedure D, the crude residue was purified by flash column chromatography on silica gel (DCM: MeOH= 30:1; R_f =0.2) to yield compound **5s** (89.5 mg, 40% yield). **^{1}H NMR** (500 MHz, DMSO) δ 10.83 (s, 1H), 8.63 (d, *J* = 7.3 Hz, 1H), 7.91 – 7.80 (m, 2H), 7.68 (dt, *J* = 5.5, 2.1 Hz, 1H), 7.37 – 7.32 (m, 8H), 6.91 (d, *J* = 8.1 Hz, 1H), 6.69 (s, 1H), 6.39 (s, 1H), 5.08 (s, 2H), 4.71 – 4.65 (m, 1H), 4.39 (q, *J* = 7.1 Hz, 1H), 3.89 (t, *J* = 8.0 Hz, 1H), 3.57 (s, 3H), 3.22 – 3.12 (m, 2H), 2.98 (s, 2H), 2.95 (s, 2H), 2.81 (s, 1H), 2.49 (s, 3H), 2.43 (s, 3H), 2.42 – 2.38 (m, 2H), 2.19 (td, *J* = 7.4, 1.6 Hz, 1H), 2.00 (s, 3H), 1.94 (q, *J* = 7.9 Hz, 1H), 1.87 – 1.80 (m, 1H), 1.57 (s, 1H), 1.48 (dd, *J* = 6.6, 1.8 Hz, 1H), 1.43 (s, 1H), 1.40 (s, 9H), 1.36 (d, *J* = 2.6 Hz, 15H). **^{13}C NMR** (126 MHz, DMSO) δ 178.98, 172.92, 172.64, 172.12, 171.57, 157.91, 156.51, 155.81, 149.36, 137.72, 136.61, 134.67, 133.35, 131.90, 131.18, 128.86, 128.45, 128.37, 127.55, 125.77, 124.76, 123.56, 123.12, 121.22, 117.98, 116.71, 115.47, 86.73, 78.66, 65.95, 52.41, 51.65, 42.94, 41.30, 34.13, 31.74, 31.61, 30.10, 29.46, 29.14, 28.74, 28.60, 28.50, 28.23, 24.95, 22.54, 19.39, 18.04, 14.39, 12.71. **HRMS** (ESI) *m/z* calcd for $C_{57}H_{72}N_8O_{14}SNa$ $(M + Na)^+$ 1147.4781, found 1147.4782.

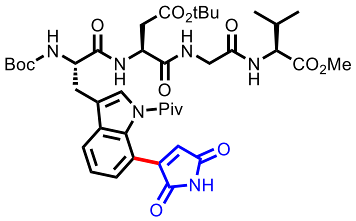

5t

According to the general procedure D, the crude residue was purified by flash column chromatography on silica gel (DCM: MeOH= 40:1; R_f =0.25) to yield compound **5t** (80.8 mg, 49% yield). **^{1}H NMR** (600 MHz, $CDCl_3$) δ 7.90 (d, *J* = 19.9 Hz, 1H), 7.69 (dd, *J* = 7.7, 1.4 Hz, 1H), 7.63 (s, 1H), 7.48 (d, *J* = 8.0 Hz, 1H), 7.32 (t, *J* = 7.6 Hz, 1H), 7.29 (dd, *J* = 7.4, 1.3 Hz, 2H), 6.87 (d, *J* = 8.4 Hz, 1H), 6.46 (s, 1H), 5.31 (d, *J* = 6.6 Hz, 1H), 4.74 – 4.66 (m, 1H), 4.50 (dd, *J* = 8.7, 5.5 Hz, 2H), 3.93 (qd, *J* = 16.9, 5.7 Hz, 2H), 3.72 (s, 3H), 3.24 (ddd, *J* = 54.7, 15.5, 6.4 Hz, 2H), 2.90 (d, *J* = 16.3 Hz, 1H), 2.60 (dd, *J* = 16.9, 6.2 Hz, 1H), 2.19 – 2.14 (m, 1H), 1.45 (s, 9H), 1.42 (s, 9H), 1.41 (s, 9H), 0.93 (t, *J* = 7.1 Hz, 6H). **^{13}C NMR** (151 MHz, $CDCl_3$) δ 178.68, 172.33, 171.63, 171.15, 170.93, 170.62, 170.53, 168.68, 150.20, 133.63, 131.20, 127.65, 125.05, 123.51, 122.06, 121.36, 117.51, 115.43, 81.98, 81.00, 57.37, 54.70, 52.14, 49.88, 43.27, 41.31, 36.52, 31.11, 29.68, 28.60, 28.37, 28.25, 27.96, 18.95, 18.00. **HRMS** (ESI) *m/z* calcd for $C_{41}H_{56}N_6O_{12}Na$ $(M + Na)^+$ 847.3848, found 847.3844.

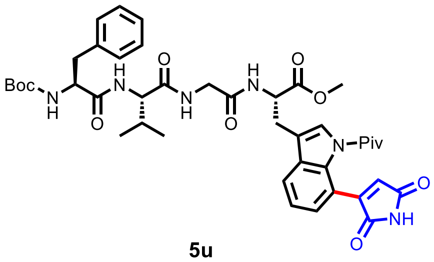

5u

According to the general procedure D, the crude residue was purified by flash column chromatography on silica gel (DCM: MeOH= 25:1; R_f =0.25) to yield compound **5u** (74.7 mg, 47% yield). **^{1}H NMR** (500 MHz, $CDCl_3$) δ 7.98 (s, 1H), 7.63 (dd, *J* = 7.7, 1.3 Hz, 1H), 7.60 (s, 1H), 7.31 (t, *J* = 7.6 Hz, 1H), 7.27 – 7.23 (m, 4H), 7.23 – 7.19 (m, 1H), 7.14 – 7.10 (m, 2H), 6.94 (s, 1H), 6.73 (d, *J* = 5.9 Hz, 1H), 6.43 (d, *J* = 1.5 Hz, 1H), 5.16 (d, *J* = 7.1 Hz, 1H), 4.97 (q, *J* = 6.8 Hz, 1H), 4.31 (q, *J* = 7.0 Hz, 1H), 4.19 (t, *J* = 6.9 Hz, 1H), 3.97 (dd, *J* = 16.6, 5.9 Hz, 1H), 3.81 (dd, *J* = 16.4, 5.3 Hz, 1H), 3.71 (s, 3H), 3.36 – 3.21 (m, 2H), 2.93 (qd, *J* = 14.5, 13.9, 7.5 Hz, 2H), 2.08 – 1.99 (m, 1H), 1.46 (s, 9H), 1.38 (s, 9H), 0.81 (d, *J* = 6.8 Hz, 3H), 0.73 (d, *J* = 6.8 Hz, 3H). **^{13}C NMR** (126 MHz, $CDCl_3$) δ 178.69, 172.00, 171.89, 171.34, 171.10, 170.74, 168.93, 155.85, 150.27, 136.25, 133.64, 131.37, 129.21, 128.66, 127.51, 127.01, 124.74, 123.43, 121.83, 121.18, 117.54, 115.23, 80.69, 58.80, 55.94, 52.58, 52.00, 43.21, 41.26, 37.80, 30.07, 28.38, 28.23, 27.44, 19.16, 17.43. **HRMS** (ESI) *m/z* calcd for $C_{42}H_{52}N_6O_{10}Na$ $(M + Na)^+$ 823.3637, found 823.3636.

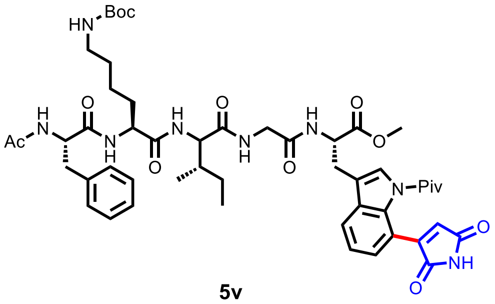

5v

According to the general procedure D, the crude residue was purified by flash column chromatography on silica gel (DCM: MeOH= 20:1; R_f =0.2) to yield compound **5v** (82.1 mg, 42% yield). **^{1}H NMR** (500 MHz, DMSO) δ 10.84 (s, 1H), 8.48 (d, *J* = 7.6 Hz, 1H), 8.16 (t, *J* = 5.8 Hz, 1H), 8.11 (d, *J* = 7.8 Hz, 1H), 8.08 (d, *J* = 8.3 Hz, 1H), 7.83 (s, 1H), 7.74 (d, *J* = 8.2 Hz, 1H), 7.69 (dd, *J* = 7.0, 2.1 Hz, 1H), 7.38 – 7.33 (m, 2H), 7.27 – 7.22 (m, 5H), 7.18 (ddd, *J* = 8.7, 4.9, 3.9 Hz, 1H), 6.72 (t, *J* = 4.8 Hz, 1H), 6.71 (d, *J* = 1.2 Hz, 1H), 4.65 (td, *J* = 7.9, 5.6 Hz, 1H), 4.52 (ddd, *J* = 10.1, 8.3, 4.0 Hz, 1H), 4.28 – 4.22 (m, 1H), 4.19 – 4.14 (m, 1H), 3.74 (t, *J* = 5.5 Hz, 2H), 3.62 (q, *J* = 1.6 Hz, 1H), 3.60 (s, 3H), 3.23 (dd, *J* = 14.9, 5.6 Hz, 1H), 3.11 (dd, *J* = 15.0, 8.2 Hz, 1H), 2.99 (dd, *J* = 13.9, 4.0 Hz, 1H), 2.87 (q, *J* = 6.4, 5.7 Hz, 2H), 2.71 (dd, *J* = 13.9, 10.2 Hz, 1H), 1.75 (d, *J* = 4.2 Hz, 1H), 1.74 (s, 3H), 1.73 – 1.60 (m, 2H), 1.46 – 1.44 (m, 2H), 1.39 (s, 9H), 1.36 (s, 9H), 1.23 – 1.16 (m, 1H), 1.12 – 1.04 (m, 1H), 0.84 – 0.80 (m, 6H). **^{13}C NMR** (126 MHz, DMSO) δ 178.98, 172.93, 172.28, 172.24, 171.92, 171.89, 171.51, 169.66, 169.20, 155.99, 149.37, 138.50, 133.37,

131.19, 129.58, 128.42, 127.54, 126.62, 125.90, 123.57, 123.17, 121.21, 118.00, 115.65, 99.99, 77.79, 57.41, 54.30, 53.11, 52.44, 41.96, 41.33, 37.91, 37.13, 31.89, 29.71, 28.73, 28.52, 28.25, 26.80, 24.70, 23.22, 22.86, 15.72, 11.54. **HRMS** (ESI) *m/z* calcd for $C_{51}H_{68}N_8O_{12}Na$ $(M + Na)^+$ 1007.4849, found 1007.4842.

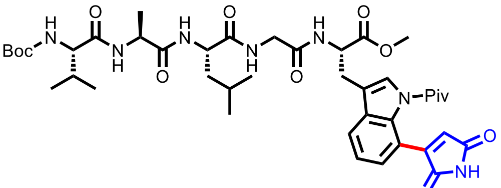

5w

According to the general procedure D, the crude residue was purified by flash column chromatography on silica gel (DCM: MeOH= 20:1; R_f =0.25) to yield compound **5w** (83.2 mg, 50% yield). **^{1}H NMR** (500 MHz, $CDCl_3$) δ 8.00 (s, 1H), 7.72 (s, 1H), 7.64 (d, *J* = 7.5 Hz, 2H), 7.58 (s, 1H), 7.30 (t, *J* = 7.6 Hz, 1H), 7.25 (dd, *J* = 7.4, 1.3 Hz, 1H), 6.42 (d, *J* = 1.5 Hz, 1H), 5.47 (s, 1H), 5.03 (q, *J* = 7.1 Hz, 1H), 4.60 (s, 1H), 4.34 (s, 1H), 4.26 (d, *J* = 16.1 Hz, 1H), 4.01 (s, 1H), 3.90 – 3.82 (m, 1H), 3.70 (s, 3H), 3.28 (qd, *J* = 15.5, 6.6 Hz, 2H), 2.01 (t, *J* = 7.5 Hz, 3H), 1.67 – 1.57 (m, 2H), 1.50 – 1.48 (m, 1H), 1.43 (s, 9H), 1.42 (s, 9H), 1.21 (d, *J* = 6.9 Hz, 3H), 0.92 – 0.84 (m, 12H). **^{13}C NMR** (126 MHz, $CDCl_3$) δ 178.60, 172.72, 172.09, 171.92, 171.10, 170.70, 169.32, 156.40, 150.32, 133.59, 131.43, 127.34, 124.62, 123.35, 121.80, 121.23, 117.43, 115.61, 80.36, 60.38, 52.48, 52.14, 51.85, 49.51, 43.06, 41.21, 30.64, 29.67, 28.36, 28.26, 27.44, 24.77, 22.91, 21.79, 19.23, 17.73. **HRMS** (ESI) *m/z* calcd for $C_{42}H_{59}N_7O_{11}Na$ $(M + Na)^+$ 860.4165, found 860.4166.

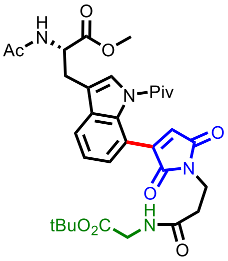

7a

According to the general procedure D, the crude residue was purified by flash column chromatography on silica gel (DCM: MeOH= 25:1; R_f =0.2) to yield compound **7a** (86.7 mg, 70% yield). **^{1}H NMR** (500 MHz, $CDCl_3$) δ 7.59 (dd, *J* = 7.5, 1.5 Hz, 1H), 7.53 (s, 1H), 7.31 (t, *J* = 7.5 Hz, 1H), 7.27 (dd, *J* = 7.4, 1.5 Hz, 1H), 6.46 (s, 1H), 6.21 (dd, *J* = 6.5, 4.2 Hz, 2H), 5.02 (dt, *J* = 7.9, 5.7 Hz, 1H), 3.91 (d, *J* = 5.0 Hz, 2H), 3.87 – 3.83 (m, 2H), 3.73 (s, 3H), 3.37 – 3.21 (m, 2H), 2.59 (dd, *J* = 8.5, 6.6 Hz, 2H), 1.99 (s, 3H), 1.45 (s, 9H), 1.45 (s, 9H). **^{13}C NMR** (126 MHz, $CDCl_3$) δ 178.59, 172.09, 170.93, 170.23, 169.81, 169.78, 168.89, 149.60, 133.53, 131.57, 127.67, 124.72, 123.45, 121.15, 121.11, 117.76, 115.18, 82.27, 52.56, 52.14, 42.02, 41.25, 34.78, 34.12, 29.66, 28.42, 28.17, 28.01, 27.39, 23.15. **HRMS** (ESI) *m/z* calcd for $C_{42}H_{40}N_4O_9Na$ $(M + Na)^+$ 647.2687, found 647.2688.

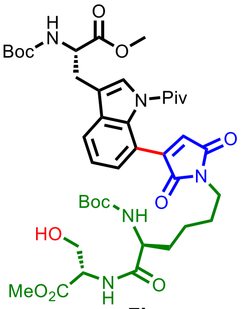

7b

According to the general procedure D, the crude residue was purified by flash column chromatography on silica gel (DCM: MeOH= 30:1; R_f=0.35) to yield compound **7b** (77.6 mg, 47% yield). **^{1}H NMR** (500 MHz, DMSO-d_6) δ 8.05 (d, *J* = 7.7 Hz, 1H), 7.90 (s, 1H), 7.72 (dd, *J* = 6.8, 2.2 Hz, 1H), 7.45 (d, *J* = 8.1 Hz, 1H), 7.41 – 7.32 (m, 2H), 6.84 (d, *J* = 4.7 Hz, 2H), 5.06 (t, *J* = 5.6 Hz, 1H), 4.39 – 4.30 (m, 2H), 3.96 (dt, *J* = 13.2, 5.0 Hz, 1H), 3.71 (dt, *J* = 10.8, 5.4 Hz, 1H), 3.66 (s, 3H), 3.62 (s, 3H), 3.61 – 3.58 (m, 1H), 3.39 (d, *J* = 7.2 Hz, 1H), 3.34 (d, *J* = 2.5 Hz, 1H), 3.18 (dd, *J* = 14.9, 4.6 Hz, 1H), 3.02 (dd, *J* = 14.9, 10.3 Hz, 1H), 1.67 – 1.52 (m, 2H), 1.52 – 1.44 (m, 4H), 1.40 (s, 9H), 1.37 (s, 9H), 1.32 (s, 9H). **^{13}C NMR** (126 MHz, DMSO) δ 178.65, 172.98, 171.54, 171.41, 170.69, 155.84, 155.74, 148.96, 133.26, 131.31, 127.71, 126.12, 123.70, 122.10, 121.37, 119.56, 118.04, 116.28, 78.79, 78.47, 61.71,

55.36, 54.96, 54.29, 53.71, 52.42, 52.26, 41.27, 37.65, 31.99, 28.64, 28.57, 28.29, 26.32, 23.16. **HRMS** (ESI) *m/z* calcd for $C_{41}H_{57}N_5O_{13}Na$ $(M + Na)^+$ 850.3845, found 850.3847

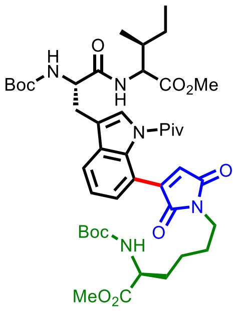

7c

According to the general procedure D, the crude residue was purified by flash column chromatography on silica gel (ethyl acetate: petroleum ether= 1:1; R_f =0.4) to yield compound **7c** (124.0mg, 73% yield). **^{1}H NMR** (400 MHz, $CDCl_3$) δ 7.69 (dd, *J* = 7.6, 1.5 Hz, 1H), 7.62 (s, 1H), 7.33 (t, *J* = 7.5 Hz, 1H), 7.29 (dd, *J* = 7.5, 1.5 Hz, 1H), 6.54 (d, *J* = 8.5 Hz, 1H), 6.46 (s, 1H), 5.19 (d, *J* = 8.3 Hz, 1H), 5.06 (d, *J* = 8.4 Hz, 1H), 4.63 – 4.45 (m, 2H), 4.29 (td, *J* = 7.9, 4.9 Hz, 1H), 3.74 (s, 3H), 3.68 (s, 3H), 3.51 (t, *J* = 7.4 Hz, 2H), 3.23 (d, *J* = 5.8 Hz, 2H), 1.87 – 1.78 (m, 2H), 1.67 – 1.61 (m, 2H), 1.48 – 1.42 (m, 27H), 1.38 – 1.32 (m, 2H), 1.16 – 1.08 (m, 1H), 0.90 – 0.86 (m, 5H), 0.84 (d, *J* = 6.9 Hz, 3H). **^{13}C NMR** (101 MHz, $CDCl_3$) δ 178.51, 173.28, 171.73, 171.35, 170.87, 170.55, 155.60, 155.37, 149.54, 133.62, 131.49, 127.61, 125.13, 123.51, 121.31, 121.01, 117.77, 115.54, 80.52, 79.88, 56.60, 54.01, 53.28, 52.32, 52.19, 41.24, 37.83, 37.44, 32.19, 29.07, 28.46, 28.31, 28.27, 25.03, 22.53, 15.36, 11.58, 11.47. **HRMS** (ESI) *m/z* calcd for $C_{44}H_{63}N_5O_{12}Na$ $(M + Na)^+$ 876.4365, found 876.4366.

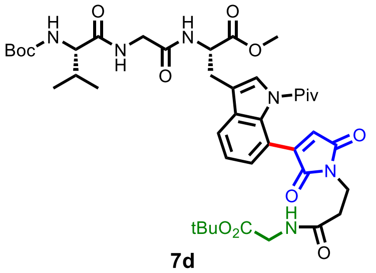

7d

According to the general procedure D, the crude residue was purified by flash column chromatography on silica gel (DCM: MeOH= 30:1; R_f =0.5) to yield compound **7d** (115.6 mg, 69% yield). **^{1}H NMR** (500 MHz, $CDCl_3$) δ 7.59 – 7.57 (m, 1H), 7.30 (t, *J* = 7.6 Hz, 1H), 7.25 (dd, *J* = 7.4, 1.4 Hz, 1H), 7.18 (d, *J* = 7.7 Hz, 1H), 6.97 (q, *J* = 5.5, 4.9 Hz, 1H), 6.44 (s, 1H), 6.38 (t, *J* = 5.2 Hz, 1H), 5.19 (d, *J* = 8.3 Hz, 1H), 4.94 (dt, *J* = 7.8, 6.2 Hz, 1H), 3.90 (t, *J* = 6.7 Hz, 6H), 3.86 – 3.82 (m, 2H), 3.69 (s, 3H), 3.25 (ddd, *J* = 50.6, 15.2, 6.3 Hz, 2H), 2.66 – 2.55 (m, 3H), 1.45 (s, 9H), 1.43 (s, 9H), 1.41 (s, 9H), 0.90 (d, *J* = 6.8 Hz, 3H), 0.86 (d, *J* = 6.7 Hz, 3H). **^{13}C NMR** (126 MHz, $CDCl_3$) δ 178.64, 172.35, 171.74, 170.96, 170.79, 170.28, 169.94, 169.88, 168.99, 168.93, 168.79, 149.57, 133.57, 131.32, 127.61, 124.96, 123.45, 121.09, 121.06, 117.73, 115.14, 82.21, 80.03, 60.02, 52.55, 52.22, 43.03, 42.00, 41.97, 41.24, 35.57, 35.25, 34.73, 34.24, 34.14, 33.56, 33.19, 31.88, 30.75, 30.29, 29.65, 29.61, 29.31, 28.95, 28.40, 28.29, 28.08, 28.02, 28.00, 27.40, 25.39, 22.65, 19.21, 17.68, 14.07. **HRMS** (ESI) *m/z* calcd for $C_{42}H_{58}N_6O_{12}Na$ $(M + Na)^+$ 861.4005, found 861.4006.

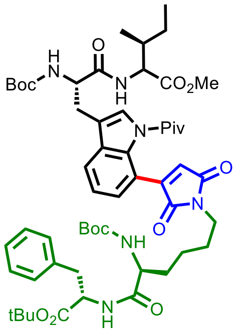

7e

According to the general procedure D, the crude residue was purified by flash column chromatography on silica gel (ethyl acetate: petroleum ether= 1:4; R_f =0.3) to yield compound **7e** (100.0mg, 48% yield). **^{1}H NMR** (400 MHz, $CDCl_3$) δ 7.68 (dd, *J* = 7.5, 1.6 Hz, 1H), 7.62 (s, 1H), 7.34 – 7.19 (m, 6H), 7.17 – 7.12 (m, 2H), 6.58 (dd, *J* = 13.1, 7.9 Hz, 2H), 6.44 (s, 1H), 5.23 (d, *J* = 8.4 Hz, 1H), 5.03 (d, *J* = 8.1 Hz, 1H), 4.69 (q, *J* = 6.5 Hz, 1H), 4.52 (td, *J* = 10.2, 8.5, 5.4 Hz, 2H), 4.05 (q, *J* = 7.1, 6.4 Hz, 1H), 3.68 (s, 3H), 3.48 (q, *J* = 6.5, 5.8 Hz, 2H), 3.21 (t, *J* = 6.3 Hz, 2H), 3.05 (h, *J* = 6.9, 6.1 Hz, 2H), 1.88 – 1.78 (m, 2H), 1.66 – 1.54 (m, 4H), 1.43 (d, *J* = 6.8 Hz, 27H), 1.39 (s, 9H), 0.90 – 0.82 (m, 6H). **^{13}C NMR** (101 MHz, $CDCl_3$) δ 178.52, 171.75, 171.49, 171.41, 170.93, 170.56, 170.26, 155.57, 149.52, 136.12, 133.61, 131.49, 129.54, 128.36, 127.59, 126.94, 125.10, 123.48, 121.29, 120.99, 117.79, 115.56,

82.30, 80.47, 79.98, 56.60, 54.49, 54.00, 53.66, 53.48, 52.18, 41.23, 38.02, 37.81, 37.31, 31.90, 28.45, 28.29, 27.93, 25.02, 22.65, 15.36, 11.57. **HRMS** (ESI) *m/z* calcd for $C_{56}H_{78}N_6O_{13}Na$ $(M + Na)^+$ 1065.5519, found 1065.5517.

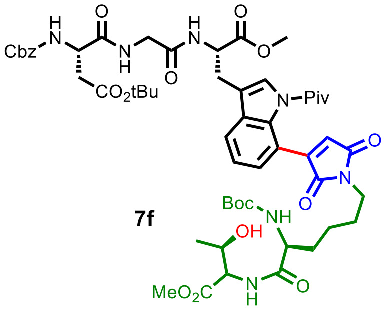

7f

According to the general procedure D, the crude residue was purified by flash column chromatography on silica gel (DCM: MeOH= 25:1; R_f =0.4) to yield compound **7f** (96.8 mg, 44% yield). **^{1}H NMR** (500 MHz, $CDCl_3$) δ 7.61 – 7.57 (m, 2H), 7.37 – 7.32 (m, 5H), 7.30 (t, *J* = 7.6 Hz, 1H), 7.27 – 7.23 (m, 2H), 7.17 (d, *J* = 8.1 Hz, 1H), 7.02 (d, *J* = 8.9 Hz, 1H), 6.43 (s, 1H), 6.05 (d, *J* = 8.4 Hz, 1H), 5.24 (d, *J* = 8.1 Hz, 1H), 5.15 – 5.07 (m, 2H), 4.96 (q, *J* = 7.1 Hz, 1H), 4.61 – 4.54 (m, 1H), 4.50 (dt, *J* = 8.5, 5.7 Hz, 1H), 4.35 – 4.27 (m, 1H), 4.12 (t, *J* = 7.3 Hz, 1H), 4.05 (dd, *J* = 16.9, 6.3 Hz, 1H), 3.86 – 3.78 (m, 1H), 3.72 (s, 3H), 3.69 (s, 3H), 3.50 (t, *J* = 7.2 Hz, 2H), 3.30 (dd, *J* = 15.2, 5.9 Hz, 2H), 3.19 (dd, *J* = 15.2, 7.1 Hz, 1H), 2.85 (dd, *J* = 16.9, 5.5 Hz, 1H), 2.70 (dd, *J* = 16.9, 6.0 Hz, 1H), 1.87 – 1.78 (m, 1H), 1.65 (dh, *J* = 21.6, 7.4, 6.9 Hz, 4H), 1.56 – 1.49 (m, 1H), 1.43 (s, 9H), 1.41 (s, 9H), 1.40 (s, 9H), 1.16 (d, *J* = 6.4 Hz, 3H). **^{13}C NMR** (126 MHz, $CDCl_3$) δ 178.55, 172.57, 172.04, 171.40, 171.12, 171.05, 170.93, 170.64, 168.82, 156.22, 155.74, 149.37, 135.83, 133.60, 131.27, 128.58, 128.37, 128.17, 127.60, 124.82, 123.42, 120.99, 117.83, 115.19, 81.97, 80.10, 68.09, 67.40, 57.36, 54.57, 52.59, 52.47, 52.11, 51.45, 43.15, 41.20, 40.90, 37.34, 37.24, 31.75, 29.68, 28.38, 28.28, 28.01, 27.39, 22.60, 19.86. **HRMS** (ESI) *m/z* calcd for $C_{55}H_{73}N_7O_{17}Na$ $(M + Na)^+$ 1126.4955, found 1126.4973

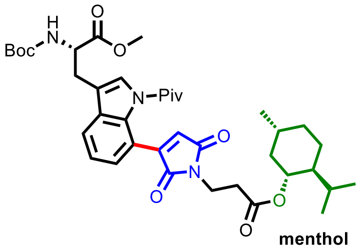

7g

According to the general procedure D, the crude residue was purified by flash column chromatography on silica gel (ethyl acetate: petroleum ether= 1:4; R_f =0.3) to yield compound **7g** (81.4mg, 58% yield). **^{1}H NMR** (500 MHz, $CDCl_3$) δ 7.61 (d, *J* = 7.6 Hz, 1H), 7.55 (s, 1H), 7.31 (t, *J* = 7.5 Hz, 1H), 7.27 (dd, *J* = 7.6, 1.3 Hz, 1H), 6.47 (s, 1H), 5.19 (d, *J* = 8.2 Hz, 1H), 4.70 (ddt, *J* = 15.3, 10.9, 5.6 Hz, 2H), 3.82 (t, *J* = 7.7 Hz, 2H), 3.71 (s, 3H), 3.25 (ddd, *J* = 50.3, 15.1, 5.8 Hz, 2H), 2.72 – 2.60 (m, 2H), 2.01 – 1.94 (m, 1H), 1.82 (pt, *J* = 7.2, 3.6 Hz, 1H), 1.66 (dp, *J* = 13.1, 3.1 Hz, 2H), 1.46 (s, 9H), 1.44 (s, 9H), 1.39 – 1.32 (m, 2H), 1.29 (s, 1H), 1.04 (qd, *J* = 13.5, 12.7, 3.8 Hz, 1H), 0.99 – 0.91 (m, 1H), 0.88 (t, *J* = 6.5 Hz, 6H), 0.74 (d, *J* = 7.0 Hz, 3H). **^{13}C NMR** (126 MHz, $CDCl_3$) δ 178.48, 172.22, 170.78, 170.27, 170.17, 155.07, 149.65, 133.55, 131.57, 127.55, 124.78, 123.38, 121.05, 117.69, 115.22, 80.14, 74.64, 53.29, 52.44, 46.90, 41.22, 40.79, 34.19, 33.56, 33.25, 31.34, 29.66, 28.46, 28.31, 26.28, 23.46, 21.96, 20.72, 16.37, 14.09. **HRMS** (ESI) *m/z* calcd for $C_{39}H_{53}N_3O_9Na$ $(M + Na)^+$ 730.3674, found 730.3678.

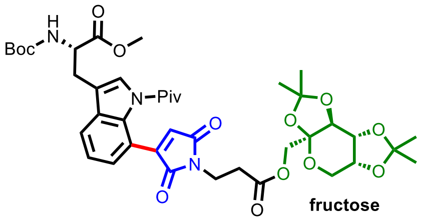

7h

According to the general procedure D, the crude residue was purified by flash column chromatography on silica gel (ethyl acetate: petroleum ether= 1:2; R_f =0.3) to yield compound **7h** (108.4mg, 67% yield). **^{1}H NMR** (500 MHz, $CDCl_3$) δ 7.62 – 7.58 (m, 1H), 7.54 (s, 1H), 7.30 (t, *J* = 7.5 Hz, 1H), 7.28 – 7.25 (m, 1H), 6.46 (s, 1H), 5.18 (d, *J* = 8.2 Hz, 1H), 4.71 (q, *J* = 6.4

Hz, 1H), 4.59 (dd, J = 7.9, 2.6 Hz, 1H), 4.41 (d, J = 11.7 Hz, 1H), 4.30 (d, J = 2.6 Hz, 1H), 4.22 (dd, J = 8.0, 1.6 Hz, 1H), 4.04 (d, J = 11.7 Hz, 1H), 3.89 (dd, J = 13.0, 2.0 Hz, 1H), 3.86 – 3.82 (m, 2H), 3.74 (d, J = 13.0 Hz, 1H), 3.70 (s, 3H), 3.24 (ddd, J = 52.8, 15.0, 5.8 Hz, 2H), 2.73 – 2.69 (m, 2H), 1.52 (s, 3H), 1.46 (s, 3H), 1.44 (s, 9H), 1.43 (s, 9H), 1.37 (s, 3H), 1.32 (s, 3H). **^{13}C NMR** (126 MHz, $CDCl_3$) δ 178.44, 172.17, 170.66, 170.07, 169.96, 155.03, 149.67, 134.15, 133.50, 131.54, 127.54, 124.71, 123.37, 121.00, 117.62, 115.26, 109.08, 108.66, 101.39, 80.10, 70.69, 70.44, 70.01, 65.29, 61.21, 53.24, 52.40, 41.18, 33.31, 32.78, 29.61, 28.42, 28.27, 26.39, 25.83, 25.18, 24.01, 14.04. **HRMS** (ESI) *m/z* calcd for $C_{41}H_{53}N_3O_{14}Na$ $(M + Na)^+$ 834.3420, found 834.3426.

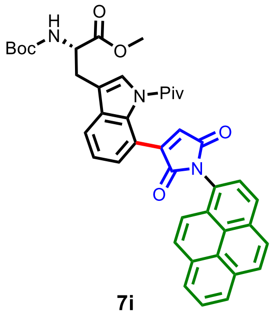

7i

According to the general procedure D, the crude residue was purified by flash column chromatography on silica gel (petroleum ether: DCM = 1:2; R_f =0.25) to yield compound **7i** (104.1mg, 75% yield). **^{1}H NMR** (500 MHz, $CDCl_3$) δ 8.28 – 8.21 (m, 3H), 8.16 – 8.10 (m, 3H), 8.08 (dd, J = 9.4, 2.7 Hz, 1H), 8.05 (t, J = 7.6 Hz, 1H), 7.91 (dd, J = 8.0, 1.5 Hz, 1H), 7.68 (d, J = 7.8 Hz, 1H), 7.57 (s, 1H), 7.51 (dd, J = 7.4, 1.2 Hz, 1H), 7.43 (t, J = 7.7 Hz, 1H), 6.82 (s, 1H), 5.18 (d, J = 8.2 Hz, 1H), 4.75 (q, J = 6.4 Hz, 1H), 3.71 (s, 3H), 3.28 (ddd, J = 48.1, 14.9, 4.7 Hz, 2H), 1.45 (s, 9H), 1.37 (s, 9H). **^{13}C NMR** (126 MHz, $CDCl_3$) δ 178.67, 172.23, 170.90, 170.18, 150.34, 133.68, 131.92, 130.99, 130.85, 128.96, 128.69, 128.35, 127.84, 127.08, 126.58, 126.29, 125.79, 125.73, 125.35, 125.21, 125.02, 124.40, 123.57, 122.24, 121.44, 121.30, 117.80, 115.35, 80.19, 53.33, 52.47, 41.26, 31.92, 29.70, 28.79, 28.53, 28.32, 22.69, 14.11. **HRMS** (ESI) *m/z* calcd for $C_{42}H_{39}N_3O_7Na$ $(M + Na)^+$ 720.2683, found 720.2685.

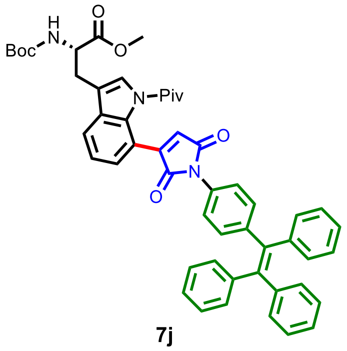

7j

According to the general procedure D, the crude residue was purified by flash column chromatography on silica gel (ethyl acetate: petroleum ether= 1:3; R_f =0.3) to yield compound **7j** (112.7mg, 70% yield). **^{1}H NMR** (400 MHz, $CDCl_3$) δ 7.65 (p, J = 3.8 Hz, 1H), 7.58 (s, 1H), 7.36 (q, J = 3.8, 2.8 Hz, 2H), 7.18 – 7.11 (m, 13H), 7.07 (ddt, J = 8.0, 5.2, 2.7 Hz, 6H), 6.61 (s, 1H), 5.19 (d, J = 8.2 Hz, 1H), 4.84 – 4.68 (m, 1H), 3.74 (s, 3H), 3.41 – 3.18 (m, 2H), 1.45 (d, J = 23.8 Hz, 18H). **^{13}C NMR** (101 MHz, $CDCl_3$) δ 178.68, 172.30, 170.16, 169.26, 155.12, 149.51, 143.56, 143.40, 143.37, 143.00, 141.64, 140.12, 133.66, 131.85, 131.62, 131.44, 131.37, 131.34, 129.96, 127.78, 127.75, 127.69, 127.64, 126.63, 126.59, 126.56, 125.38, 125.28, 124.81, 123.50, 121.43, 121.01, 117.62, 115.26, 80.23, 53.31, 52.56, 41.24, 28.48, 28.37, 27.73. **HRMS** (ESI) *m/z* calcd for $C_{52}H_{49}N_3O_7Na$ $(M + Na)^+$ 827.3571, found 827.3573.

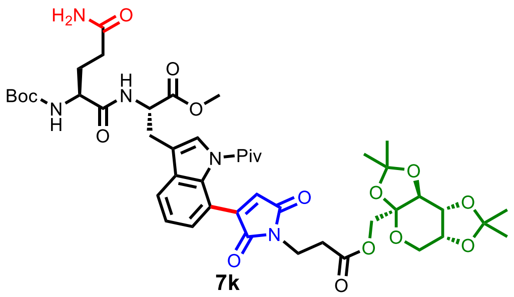

7k

According to the general procedure D, the crude residue was purified by flash column chromatography on silica gel (DCM: MeOH= 18:1; R_f =0.3) to yield compound **7k** (122.0 mg, 65% yield). **^{1}H NMR** (500 MHz, $CDCl_3$) δ 8.20 (d, J = 7.9 Hz, 1H), 7.71 (s, 1H), 7.62 (d, J = 7.6 Hz, 1H), 7.30 (t, J = 7.6 Hz, 1H), 7.25 (d, J = 7.3 Hz, 1H), 6.56 (s, 1H), 6.45 (s, 1H), 6.06 (s, 1H), 5.69 (d, J = 7.4 Hz, 1H), 4.93 (q, J = 7.7 Hz, 1H), 4.58 (dd, J = 7.9, 2.7 Hz, 1H), 4.40 (d, J = 11.7 Hz, 1H), 4.29 (d, J = 2.6 Hz, 1H), 4.22 (dd, J = 7.9, 1.7

Hz, 2H), 4.03 (d, J = 11.7 Hz, 1H), 3.88 (dd, J = 13.0, 1.9 Hz, 2H), 3.83 (t, J = 7.7 Hz, 2H), 3.75 (s, 1H), 3.73 (s, 3H), 3.29 (dd, J = 15.1, 4.7 Hz, 1H), 3.15 (dd, J = 15.0, 8.5 Hz, 1H), 2.75 – 2.65 (m, 2H), 2.24 (q, J = 7.5, 6.8 Hz, 2H), 1.95 (q, J = 7.7 Hz, 2H), 1.52 (s, 3H), 1.46 (s, 3H), 1.42 (s, 9H), 1.39 (s, 9H), 1.37 (s, 3H), 1.32 (s, 3H). **^{13}C NMR** (126 MHz, $CDCl_3$) δ 178.66, 175.47, 175.32, 172.60, 171.87, 170.73, 170.22, 170.09, 170.02, 155.71, 149.71, 133.54, 131.29, 127.59, 127.54, 125.29, 125.15, 123.55, 123.48, 121.13, 120.93, 117.61, 115.33, 109.10, 108.74, 108.72, 101.38, 99.99, 79.98, 70.69, 70.56, 70.44, 70.00, 65.63, 65.31, 61.22, 53.45, 53.23, 52.63, 51.86, 41.21, 33.33, 32.77, 31.51, 31.37, 29.27, 28.91, 28.71, 28.35, 28.28, 27.75, 27.23, 26.44, 25.87, 25.20, 24.03. **HRMS** (ESI) *m/z* calcd for $C_{46}H_{61}N_5O_{16}Na$ $(M + Na)^+$ 962.4006, found 962.4031.

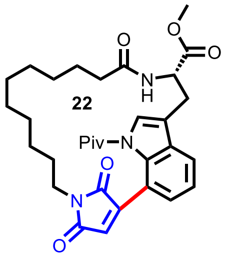

9a

According to the general procedure E, the crude residue was purified by PTLC (ethyl acetate: petroleum ether= 2:3; R_f =0.4) to yield compound **9a** (14.9 mg, 22% yield). **^{1}H NMR** (500 MHz, DMSO) δ 8.15 (d, J = 8.7 Hz, 1H), 7.96 (s, 1H), 7.74 (dd, J = 7.7, 1.3 Hz, 1H), 7.39 (t, J = 7.6 Hz, 1H), 7.34 (dd, J = 7.4, 1.3 Hz, 1H), 6.84 (s, 1H), 4.56 (ddd, J = 12.4, 8.6, 3.3 Hz, 1H), 3.75 (s, 3H), 3.52 (ddd, J = 13.9, 6.8, 3.8 Hz, 1H), 3.43 (ddd, J = 14.0, 8.2, 3.7 Hz, 1H), 3.31 (dd, J = 13.9, 3.3 Hz, 1H), 2.93 (dd, J = 14.1, 12.7 Hz, 1H), 2.04 – 1.90 (m, 2H), 1.47 (s, 1H), 1.41 (s, 9H), 1.33 – 1.25 (m, 3H), 1.22 – 1.15 (m, 3H), 1.14 – 0.78 (m, 9H). **^{13}C NMR** (126 MHz, DMSO) δ 178.26, 172.95, 172.27, 171.48, 171.22, 147.67, 133.89, 130.46, 127.39, 127.07, 123.53, 122.56, 120.79, 117.97, 115.32, 52.60, 51.10, 41.35, 36.18, 36.12, 30.02, 29.99, 29.48, 28.87, 28.63, 28.25, 27.50, 26.69, 25.58, 24.99. **HRMS** (ESI) *m/z* calcd for $C_{32}H_{41}N_3O_6Na$ $(M + Na)^+$ 586.2888, found 586.2893.

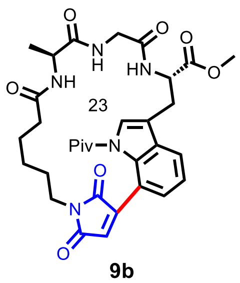

9b

According to the general procedure E, the crude residue was purified by PTLC (DCM: MeOH= 25:1; R_f =0.35) to yield compound **9b** (11.2 mg, 15% yield). **^{1}H NMR** (500 MHz, $CDCl_3$) δ 7.63 (dd, J = 7.8, 1.3 Hz, 1H), 7.59 (s, 1H), 7.33 (t, J = 7.7 Hz, 1H), 7.28 (d, J = 1.3 Hz, 1H), 7.27 (d, J = 1.2 Hz, 0H), 6.91 (d, J = 7.1 Hz, 1H), 6.74 (d, J = 6.1 Hz, 1H), 6.44 (s, 1H), 6.34 (d, J = 8.3 Hz, 1H), 4.89 (ddd, J = 8.7, 7.1, 4.7 Hz, 1H), 4.32 (dd, J = 8.1, 7.0 Hz, 1H), 3.97 (dd, J = 15.6, 6.0 Hz, 1H), 3.87 (s, 3H), 3.75 – 3.62 (m, 2H), 3.56 – 3.47 (m, 2H), 3.41 (dd, J = 14.8, 4.7 Hz, 1H), 3.10 (dd, J = 14.9, 8.6 Hz, 1H), 2.24 (ddd, J = 13.5, 7.3, 5.8 Hz, 1H), 2.17 – 2.07 (m, 1H), 1.87 – 1.78 (m, 1H), 1.67 (tt, J = 13.5, 6.3 Hz, 2H), 1.48 (s, 9H), 1.32 (ddd, J = 9.1, 6.9, 3.0 Hz, 2H), 0.46 (d, J = 7.1 Hz, 3H). **^{13}C NMR** (126 MHz, $CDCl_3$) δ 178.63, 173.37, 172.27, 172.11, 171.30, 171.07, 169.31, 147.81, 133.99, 130.82, 127.46, 125.56, 123.47, 121.34, 120.67, 117.88, 114.22, 52.76, 51.47, 47.92, 43.50, 41.34, 36.71, 29.68, 28.58, 28.31, 27.20, 25.18, 24.16, 18.32. **HRMS** (ESI) *m/z* calcd for $C_{32}H_{39}N_5O_8Na$ $(M + Na)^+$ 644.2691, found 644.2694.

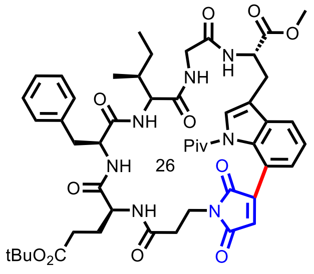

9c

According to the general procedure E, the crude residue was purified by flash column chromatography on silica gel (DCM: MeOH= 25:1; R_f =0.55) to yield compound **9c** (40.0 mg, 35% yield).**^{1}H NMR** (500 MHz, DMSO) δ 8.53 (d, J = 8.4 Hz, 1H), 7.98 (s, 1H), 7.89 (s, 1H), 7.69 (t, J = 26.4 Hz, 2H), 7.44 – 7.22 (m, 4H), 7.09 (s, 4H), 6.81 (d, J = 37.3 Hz, 3H), 4.51 (d, J = 74.3 Hz, 2H), 4.06 (t, J = 7.2 Hz, 1H), 3.74 (s, 9H), 3.29 (d, J = 14.0 Hz, 1H), 2.93 (t, J = 13.4 Hz, 1H), 2.78 (d, J = 15.6 Hz, 1H),

2.36 (d, *J* = 37.1 Hz, 3H), 1.94 – 1.78 (m, 2H), 1.73 – 1.60 (m, 2H), 1.47 (s, 1H), 1.38 (d, *J* = 3.0 Hz, 18H), 0.74 (d, *J* = 18.6 Hz, 6H). **^{13}C NMR** (126 MHz, DMSO) δ 171.88, 171.39, 170.84, 170.77, 170.74, 170.45, 170.43, 170.30, 170.28, 170.27, 170.11, 168.31, 168.28, 167.40, 137.86, 137.52, 132.74, 130.38, 128.75, 127.70, 127.14, 125.80, 123.01, 120.69, 117.35, 79.56, 57.30, 53.54, 52.25, 41.43, 40.93, 36.44, 34.51, 30.90, 28.98, 28.04, 27.95, 27.72, 26.59, 26.24, 24.17, 15.36, 11.38, 11.26. **HRMS** (ESI) *m/z* calcd for $C_{50}H_{63}N_7O_{12}Na$ $(M + Na)^+$ 976.4427, found 976.4429.

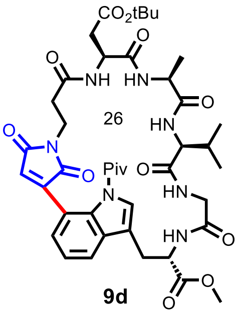

9d

According to the general procedure E, the crude residue was purified by flash column chromatography on silica gel (DCM: MeOH= 25:1; R_f =0.3) to yield compound **9d** (44.2 mg, 43% yield). **^{1}H NMR** (500 MHz, $CDCl_3$) δ 7.68 (d, *J* = 7.8 Hz, 1H), 7.37 (t, *J* = 7.7 Hz, 1H), 7.29 (s, 1H), 7.12 (d, *J* = 7.2 Hz, 2H), 7.03 (d, *J* = 8.5 Hz, 1H), 6.70 (d, *J* = 9.1 Hz, 1H), 6.54 (d, *J* = 6.4 Hz, 2H), 6.31 (s, 1H), 5.22 (ddd, *J* = 12.2, 8.5, 3.9 Hz, 1H), 4.73 (ddd, *J* = 12.0, 9.2, 3.3 Hz, 1H), 4.43 (dd, *J* = 17.2, 8.3 Hz, 1H), 3.95 (dqt, *J* = 14.7, 9.5, 5.5 Hz, 2H), 3.82 (d, *J* = 9.2 Hz, 4H), 3.46 – 3.32 (m, 2H), 3.28 (dd, *J* = 17.2, 4.5 Hz, 1H), 2.75 (dd, *J* = 16.9, 11.2 Hz, 1H), 2.56 (dd, *J* = 13.6, 6.6 Hz, 1H), 2.40 (dd, *J* = 16.9, 3.3 Hz, 1H), 2.35 – 2.28 (m, 1H), 2.14 (h, *J* = 6.8 Hz, 1H), 1.84 – 1.76 (m, 1H), 1.38 (s, 9H), 1.33 (s, 9H), 0.92 (dd, *J* = 9.4, 6.8 Hz, 6H), 0.64 (d, *J* = 7.3 Hz, 3H). **^{13}C NMR** (126 MHz, $CDCl_3$) δ 179.04, 173.65, 171.70, 171.28, 170.99, 170.45, 170.06, 147.86, 133.35, 131.92, 126.08, 123.95, 123.36, 122.79, 120.91, 117.69, 116.36, 81.68, 60.63, 52.74, 51.59, 49.81, 48.32, 42.72, 41.41, 38.19, 36.61, 34.53, 29.64, 28.64, 28.59, 27.83, 26.11, 19.41, 19.31, 16.05, 9.45. **HRMS** (ESI) *m/z* calcd for $C_{42}H_{55}N_7O_{12}Na$ $(M + Na)^+$ 872.3801, found 872.3803.

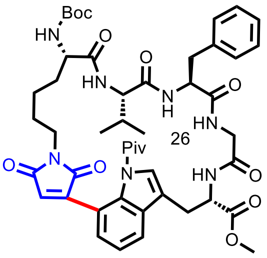

9e

According to the general procedure E, the crude residue was purified by PTLC (ethyl acetate: petroleum ether= 3:1; R_f =0.4) to yield compound **9e** (42.8 mg, 39% yield).**^{1}H NMR** (500 MHz, $CDCl_3$) δ 7.81 (s, 1H), 7.62 (d, *J* = 7.7 Hz, 1H), 7.45 (s, 1H), 7.39 (d, *J* = 6.1 Hz, 1H), 7.29 (d, *J* = 8.2 Hz, 2H), 7.24 (d, *J* = 7.6 Hz, 1H), 7.20 – 7.08 (m, 6H), 6.49 (s, 1H), 5.87 (s, 1H), 5.32 (d, *J* = 11.3 Hz, 1H), 4.91 (s, 1H), 4.61 (s, 1H), 4.50 (dd, *J* = 17.6, 7.4 Hz, 1H), 3.95 (d, *J* = 5.5 Hz, 1H), 3.82 (s, 3H), 3.68 (dd, *J* = 17.6, 8.6 Hz, 1H), 3.56 (dd, *J* = 17.4, 4.9 Hz, 1H), 3.42 (dq, *J* = 27.8, 15.9, 15.4 Hz, 5H), 3.05 – 2.97 (m, 1H), 2.51 (s, 1H), 2.30 (t, *J* = 13.8 Hz, 1H), 2.14 – 1.98 (m, 2H), 1.79 – 1.68 (m, 1H), 1.58 (s, 1H), 1.42 (s, 9H), 1.15 (s, 9H), 0.43 (d, *J* = 6.9 Hz, 3H), 0.21 (d, *J* = 6.9 Hz, 3H). **^{13}C NMR** (126 MHz, $CDCl_3$) δ 178.51, 172.68, 171.87, 171.81, 171.04, 169.88, 156.72, 137.45, 133.96, 131.72, 129.45, 128.27, 127.92, 127.01, 126.35, 123.07, 121.06, 118.98, 117.40, 115.98, 81.31, 59.52, 56.33, 54.93, 52.71, 49.68, 48.00, 43.24, 41.03, 35.24, 34.91, 29.65, 28.94, 28.69, 28.59, 28.44, 27.83, 26.23, 19.52, 19.30, 16.57. **HRMS** (ESI) *m/z* calcd for $C_{48}H_{61}N_7O_{11}Na$ $(M + Na)^+$ 934.4321, found 934.4328.

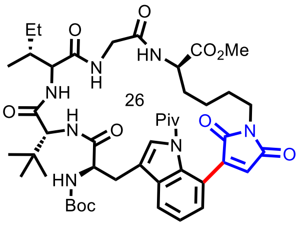

9f

According to the general procedure E, the crude residue was purified by flash column chromatography on silica gel (DCM: MeOH= 25:1; R_f =0.45) to yield compound **9f** (36.4 mg, 34% yield). **^{1}H NMR** (600 MHz, $CDCl_3$) δ 7.77 (s, 1H), 7.60 – 7.34 (m, 2H), 7.27 (d, *J* = 5.2 Hz, 1H), 7.09 – 6.49 (m, 3H), 6.10 (t, *J* = 31.0 Hz, 2H), 5.63 (s, 1H), 5.03 (s, 1H), 4.80 (s, 1H), 4.47 (d, *J* = 34.4 Hz, 2H), 3.75 (s, 3H), 3.70 – 3.45 (m, 3H), 3.37

– 3.15 (m, 2H), 3.12 – 2.77 (m, 2H), 1.94 (dt, J = 11.8, 6.0 Hz, 2H), 1.69 – 1.60 (m, 2H), 1.50 (d, J = 15.4 Hz, 18H), 1.42 (d, J = 16.1 Hz, 2H), 1.30 – 1.19 (m, 2H), 1.02 (s, 3H), 0.90 (qd, J = 6.1, 3.5, 2.7 Hz, 3H), 0.80 (s, 9H). **^{13}C NMR** (151 MHz, $CDCl_3$) δ 172.29, 171.56, 170.66, 170.14, 168.83, 162.02, 155.25, 148.98, 145.86, 140.76, 137.32, 134.22, 132.13, 126.97, 125.39, 125.34, 123.38, 117.00, 112.77, 80.32, 60.20, 59.61, 56.08, 52.47, 51.38, 42.50, 41.35, 36.94, 35.97, 34.99, 31.14, 28.69, 28.62, 28.38, 27.77, 26.30, 22.67, 21.12, 14.84, 11.71. **HRMS** (ESI) *m/z* calcd for $C_{46}H_{65}N_7O_{11}Na$ $(M + Na)^+$ 914.4634, found 914.4668.

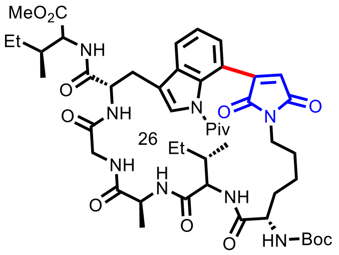

9g

According to the general procedure E, the crude residue was purified by flash column chromatography on silica gel (DCM: MeOH= 25:1; R_f =0.45) to yield compound **9g** (37.0 mg, 32% yield). **^{1}H NMR** (400 MHz, DMSO) δ 8.62 (s, 1H), 8.25 – 7.71 (m, 5H), 7.71 – 7.41 (m, 3H), 7.38 (d, J = 6.4 Hz, 2H), 7.34 – 7.13 (m, 1H), 6.97 (d, J = 32.2 Hz, 1H), 6.85 (s, 1H), 4.72 (s, 1H), 4.30 (q, J = 6.8 Hz, 1H), 4.22 – 4.01 (m, 2H), 3.88 (d, J = 15.9 Hz, 2H), 3.68 (s, 4H), 3.49 – 3.44 (m, 1H), 3.19 – 3.07 (m, 1H), 2.95 – 2.79 (m, 1H), 1.88 (p, J = 7.0, 6.3 Hz, 2H), 1.54 (d, J = 4.1 Hz, 2H), 1.45 (s, 2H), 1.37 (d, J = 5.4 Hz, 18H), 1.22 (d, J = 9.1 Hz, 3H), 1.12 (t, J = 7.2 Hz, 2H), 0.93 – 0.87 (m, 6H), 0.83 (d, J = 13.2 Hz, 9H). **^{13}C NMR** (101 MHz, DMSO) δ 178.80, 172.58, 172.43, 172.34, 172.07, 170.49, 168.52, 155.95, 148.62, 133.58, 127.49, 123.32, 121.91, 121.09, 119.13, 118.04, 116.30, 112.60, 78.55, 68.12, 60.66, 57.11, 54.44, 52.23, 48.81, 42.06, 41.53, 41.22, 36.61, 34.36, 29.48, 28.80, 28.61, 28.33, 27.10, 26.97, 25.37, 21.97, 18.28, 15.95, 11.72, 11.66, 9.05, 8.19. **HRMS** (ESI) *m/z* calcd for $C_{49}H_{70}N_8O_{12}Na$ $(M + Na)^+$ 985.5005, found 985.5007.

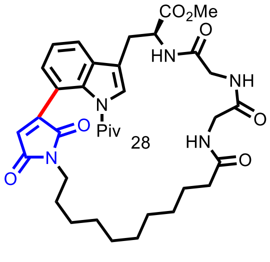

9h

According to the general procedure E, the crude residue was purified by PTLC (ethyl acetate; R_f =0.2) to yield compound **9h** (30.9 mg, 38% yield). **^{1}H NMR** (500 MHz, $CDCl_3$) δ 7.59 (s, 1H), 7.55 (dd, J = 7.8, 1.3 Hz, 1H), 7.33 (t, J = 7.6 Hz, 1H), 7.26 (dd, J = 7.4, 1.3 Hz, 1H), 7.08 (d, J = 8.3 Hz, 1H), 7.01 (t, J = 5.9 Hz, 1H), 6.44 (s, 1H), 6.43 (d, J = 5.4 Hz, 1H), 5.04 (td, J = 8.0, 4.3 Hz, 1H), 3.89 (dd, J = 16.3, 5.9 Hz, 1H), 3.82 – 3.77 (m, 4H), 3.54 (t, J = 6.8 Hz, 4H), 3.36 (dd, J = 15.7, 4.0 Hz, 1H), 3.19 (dd, J = 15.6, 7.8 Hz, 1H), 2.05 (t, J = 7.6 Hz, 2H), 1.70 – 1.60 (m, 2H), 1.53 (d, J = 8.0 Hz, 3H), 1.45 (s, 9H), 1.34 (t, J = 3.3 Hz, 5H), 1.30 – 1.26 (m, 6H).**^{13}C NMR** (126 MHz, $CDCl_3$) δ 178.49, 174.37, 171.83, 171.19, 169.84, 169.06, 149.07, 133.55, 131.49, 127.40, 125.00, 123.62, 121.51, 120.97, 117.81, 115.02, 52.73, 51.36, 43.27, 42.90, 41.22, 37.76, 35.74, 31.42, 29.66, 28.98, 28.50, 28.24, 27.91, 27.71, 27.53, 26.98, 25.42, 24.94. **HRMS** (ESI) *m/z* calcd for $C_{36}H_{47}N_5O_8Na$ $(M + Na)^+$ 700.3317, found 700.3317.

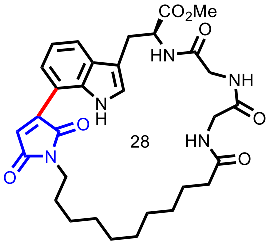

9ha

According to the general procedure G, the crude residue was purified by PTLC (DCM: MeOH= 20:1; R_f =0.2) to yield compound **9ha** (46 mg, 78% yield). **^{1}H NMR** (400 MHz, DMSO) δ 10.99 (d, J = 2.6 Hz, 1H), 8.14 (d, J = 8.2 Hz, 1H), 8.06 (t, J = 6.0 Hz, 1H), 7.93 (t, J = 5.8 Hz, 1H), 7.78 – 7.63 (m, 2H), 7.30 (d, J = 2.5 Hz, 1H), 7.22 (s, 1H), 7.16 (t, J = 7.7 Hz, 1H), 4.57 (ddd, J = 11.1, 8.2, 3.1 Hz, 1H), 3.71 (s, 3H), 3.68 – 3.61 (m, 4H), 3.58 – 3.54 (m, 2H), 3.26 (dd, J = 14.9, 3.0 Hz, 1H), 3.06 (dd, J = 14.8, 10.6 Hz,

1H), 2.04 (td, J = 7.3, 4.1 Hz, 2H), 1.63 – 1.54 (m, 2H), 1.45 – 1.34 (m, 3H), 1.29 – 1.16 (m, 11H). ^{13}**C NMR** (101 MHz, DMSO) δ 173.22, 172.61, 171.49, 171.31, 169.87, 169.34, 141.40, 133.86, 128.73, 125.76, 125.60, 123.89, 121.94, 119.22, 113.32, 110.94, 52.79, 52.63, 42.27, 42.08, 37.83, 35.50, 29.47, 28.98, 28.80, 28.63, 28.48, 27.95, 26.82, 26.17, 25.03. **HRMS** (ESI) *m/z* calcd for $C_{31}H_{39}N_5O_7Na$ (M + Na)$^+$ 616.2742, found 616.2741.

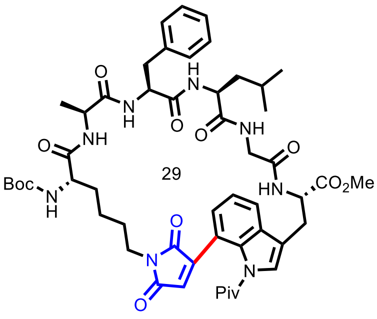

9i

According to the general procedure E, the crude residue was purified by flash column chromatography on silica gel (DCM: MeOH= 20:1; R_f =0.4) to yield compound **9i** (41.9 mg, 35% yield). ^{1}**H NMR** (500 MHz, DMSO) δ 8.50 (d, J = 8.4 Hz, 1H), 8.01 (d, J = 8.0 Hz, 1H), 7.90 (s, 1H), 7.80 – 7.66 (m, 3H), 7.58 – 7.49 (m, 1H), 7.41 – 7.35 (m, 2H), 7.21 – 7.10 (m, 6H), 6.94 (d, J = 7.8 Hz, 1H), 6.84 (s, 1H), 4.63 (t, J = 10.6 Hz, 1H), 4.43 (td, J = 8.4, 5.2 Hz, 1H), 4.29 (td, J = 9.2, 4.6 Hz, 1H), 4.15 (t, J = 7.3 Hz, 1H), 3.83 – 3.58 (m, 7H), 3.35 (d, J = 7.3 Hz, 1H), 3.33 – 3.27 (m, 1H), 3.07 (dd, J = 14.2, 5.1 Hz, 1H), 2.99 (dd, J = 14.9, 11.9 Hz, 1H), 2.76 (dd, J = 14.3, 8.9 Hz, 1H), 1.61 – 1.44 (m, 8H), 1.37 (s, 9H), 1.36 (s, 9H), 1.11 (d, J = 7.0 Hz, 3H), 0.83 (d, J = 6.5 Hz, 6H). ^{13}**C NMR** (126 MHz, DMSO) δ 178.71, 172.63, 172.42, 172.21, 171.94, 171.43, 170.67, 170.56, 168.93, 155.72, 148.77, 137.93, 133.38, 131.04, 129.51, 128.45, 127.81, 126.63, 126.26, 123.68, 121.56, 121.20, 118.06, 115.65, 78.50, 54.98, 53.95, 52.73, 51.80, 51.05, 48.59, 41.90, 41.19, 37.52, 37.06, 32.16, 28.63, 28.52, 28.38, 26.65, 24.46, 23.49, 22.81, 22.10, 18.66. **HRMS** (ESI) *m/z* calcd for $C_{52}H_{68}N_8O_{12}Na$ (M + Na)$^+$ 1019.4849, found 1019.4853.

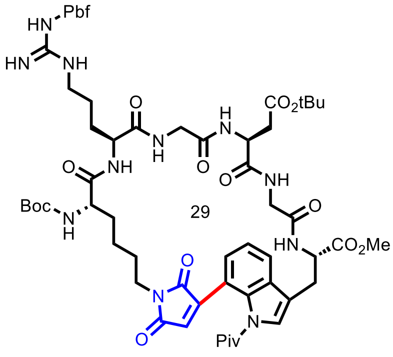

9j

According to the general procedure E, the crude residue was purified by flash column chromatography on silica gel (DCM: MeOH= 20:1; R_f =0.2) to yield compound **9j** (50.0 mg, 32% yield). ^{1}**H NMR** (500 MHz, DMSO) δ 8.56 (d, J = 8.1 Hz, 1H), 8.30 (d, J = 8.2 Hz, 1H), 8.04 (s, 1H), 7.92 (d, J = 8.3 Hz, 2H), 7.82 (d, J = 8.5 Hz, 1H), 7.76 – 7.71 (m, 1H), 7.41 – 7.35 (m, 2H), 7.31 (ddd, J = 9.0, 7.3, 3.6 Hz, 1H), 6.86 (d, J = 6.6 Hz, 2H), 6.62 (s, 1H), 6.40 (d, J = 32.5 Hz, 2H), 4.66 – 4.57 (m, 2H), 4.45 (d, J = 7.1 Hz, 1H), 4.09 (d, J = 18.1 Hz, 1H), 3.96 (dd, J = 16.9, 6.7 Hz, 1H), 3.91 – 3.82 (m, 1H), 3.76 (s, 3H), 3.71 (d, J = 3.4 Hz, 1H), 3.61 (s, 1H), 3.45 (dd, J = 17.5, 3.5 Hz, 2H), 3.28 (d, J = 14.4 Hz, 2H), 3.01 (t, J = 10.2 Hz, 3H), 2.96 (s, 2H), 2.70 (dd, J = 16.0, 5.0 Hz, 1H), 2.47 (s, 3H), 2.42 (s, 3H), 2.40 – 2.35 (m, 1H), 2.01 (s, 3H), 1.64 – 1.44 (m, 8H), 1.41 (s, 6H), 1.36 (t, J = 2.9 Hz, 27H). ^{13}**C NMR** (126 MHz, DMSO) δ 178.86, 172.45, 172.27, 171.72, 171.52, 170.77, 170.75, 169.75, 169.00, 168.82, 157.88, 156.46, 155.96, 148.80, 137.72, 134.86, 134.67, 133.39, 131.87, 130.89, 129.49, 127.81, 125.35, 124.73, 123.74, 121.55, 121.12, 118.82, 118.07, 116.69, 86.72, 80.63, 78.40, 54.20, 52.77, 52.49, 51.72, 50.03, 42.95, 42.26, 41.95, 41.27, 41.23, 38.17, 37.24, 31.66, 28.76, 28.62, 28.52, 28.28, 28.12, 28.09, 26.67, 22.77, 19.40, 18.04, 12.73. **HRMS** (ESI) *m/z* calcd for $C_{63}H_{87}N_{11}O_{17}Na$ (M + Na)$^+$ 1324.5894, found 1324.5931.

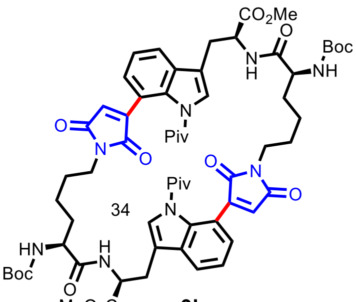

9k

According to the general procedure E, the crude residue was purified by PTLC (ethyl acetate: petroleum ether= 3:2; R_f =0.45) to yield compound **9k** (22.1 mg, 30% yield). **^{1}H NMR** (500 MHz, $CDCl_3$) δ 7.60 (s, 2H), 7.57 (d, *J* = 7.7 Hz, 2H), 7.28 – 7.23 (m, 2H), 7.19 (d, *J* = 7.4 Hz, 2H), 6.80 (d, *J* = 8.1 Hz, 2H), 6.39 (s, 2H), 4.99 (t, *J* = 8.7 Hz, 2H), 4.89 (s, 2H), 3.95 (s, 2H), 3.87 – 3.54 (m, 8H), 3.45 (d, *J* = 17.5 Hz, 4H), 3.27 (d, *J* = 14.5 Hz, 4H), 2.07 – 1.81 (m, 4H), 1.72 (q, *J* = 9.5, 8.0 Hz, 2H), 1.62 – 1.51 (m, 4H), 1.40 (d, *J* = 1.4 Hz, 36H). **^{13}C NMR** (126 MHz, $CDCl_3$) δ 178.40, 172.04, 171.60, 171.25, 170.72, 149.18, 133.56, 131.51, 127.44, 125.03, 123.45, 121.08, 117.81, 114.87, 80.19, 52.49, 51.93, 41.19, 37.07, 31.49, 29.66, 28.59, 28.36, 28.19, 27.82, 27.18, 22.47, 14.06. **HRMS** (ESI) *m/z* calcd for $C_{64}H_{80}N_8O_{16}Na$ $(M + Na)^+$ 1239.5584, found 1239.5589.

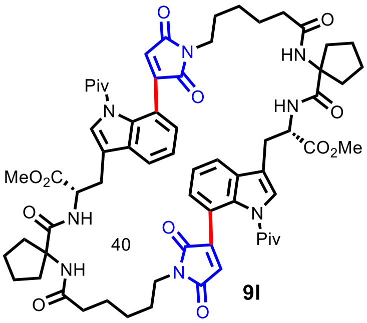

9l

According to the general procedure E, the crude residue was purified by PTLC (DCM: MeOH= 25:1; R_f =0.35) to yield compound **9l** (26.1 mg, 36% yield). **^{1}H NMR** (500 MHz, $CDCl_3$) δ 7.75 (d, *J* = 7.8 Hz, 2H), 7.69 (s, 2H), 7.60 (dd, *J* = 7.7, 1.4 Hz, 2H), 7.30 (t, *J* = 7.5 Hz, 2H), 7.26 (dd, *J* = 7.4, 1.4 Hz, 2H), 6.42 (s, 2H), 5.66 (s, 2H), 5.00 (td, *J* = 7.4, 5.0 Hz, 2H), 3.72 (s, 6H), 3.37 (t, *J* = 7.3 Hz, 4H), 3.33 – 3.21 (m, 4H), 2.35 – 2.20 (m, 4H), 2.06 (dt, *J* = 13.5, 6.4 Hz, 2H), 1.98 – 1.85 (m, 5H), 1.73 – 1.61 (m, 14H), 1.53 – 1.45 (m, 4H), 1.40 (s, 18H), 1.20 (q, *J* = 7.6 Hz, 4H). **^{13}C NMR** (126 MHz, $CDCl_3$) δ 178.57, 173.76, 173.72, 172.07, 171.35, 170.51, 149.40, 133.62, 131.63, 127.35, 124.96, 123.29, 121.17, 120.88, 117.79, 115.22, 67.63, 52.46, 51.76, 41.16, 37.41, 36.81, 36.46, 36.11, 29.68, 28.34, 28.03, 27.27, 26.16, 24.85, 23.87, 23.50. **HRMS** (ESI) *m/z* calcd for $C_{66}H_{80}N_8O_{14}Na$ $(M + Na)^+$ 1231.5686, found 1231.5689.

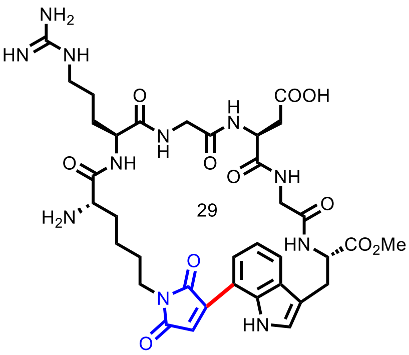

10a 75%

^{1}H NMR (400 MHz, DMSO-d_6) δ 10.86 (s, 1H), 8.57 (d, *J* = 8.4 Hz, 1H), 8.47 – 8.27 (m, 1H), 8.21 (t, *J* = 5.5 Hz, 1H), 8.12 (s, 3H), 7.97 (dd, *J* = 12.3, 8.0 Hz, 1H), 7.69 (dd, *J* = 11.5, 7.7 Hz, 1H), 7.64 – 7.33 (m, 2H), 7.30 (d, *J* = 2.8 Hz, 1H), 7.26 (s, 1H), 7.15 (t, *J* = 7.7 Hz, 1H), 7.08 – 6.78 (m, 2H), 4.66 – 4.51 (m, 2H), 4.41 (t, *J* = 7.5 Hz, 1H), 3.81 (d, *J* = 6.4 Hz, 4H), 3.72 (d, *J* = 3.9 Hz, 1H), 3.68 (s, 3H), 3.60 (d, *J* = 6.2 Hz, 1H), 3.57 – 3.42 (m, 3H), 3.25 (q, *J* = 16.4, 13.7 Hz, 2H), 3.15 – 3.03 (m, 3H), 2.80 – 2.54 (m, 2H), 1.75 – 1.64 (m, 2H), 1.63 – 1.40 (m, 6H), 1.37 – 1.21 (m, 2H). **^{13}C NMR** (101 MHz, DMSO) δ 172.39, 172.21, 171.46, 171.37, 171.23, 171.12, 169.11, 168.99, 168.97, 168.73, 162.78, 159.17, 158.83, 158.54, 157.17, 150.30, 141.86, 138.14, 134.94, 133.71, 128.75, 125.86, 125.64, 113.34, 110.43, 80.25, 52.72, 52.62, 52.56, 52.27, 49.85, 49.79, 49.75, 42.75, 42.29, 28.50, 25.30, 21.33. **HRMS** (ESI) *m/z* calcd for $C_{36}H_{48}N_{11}O_{11}$ $(M + H)^+$ 810.3529, found 810.3510.

LCMS

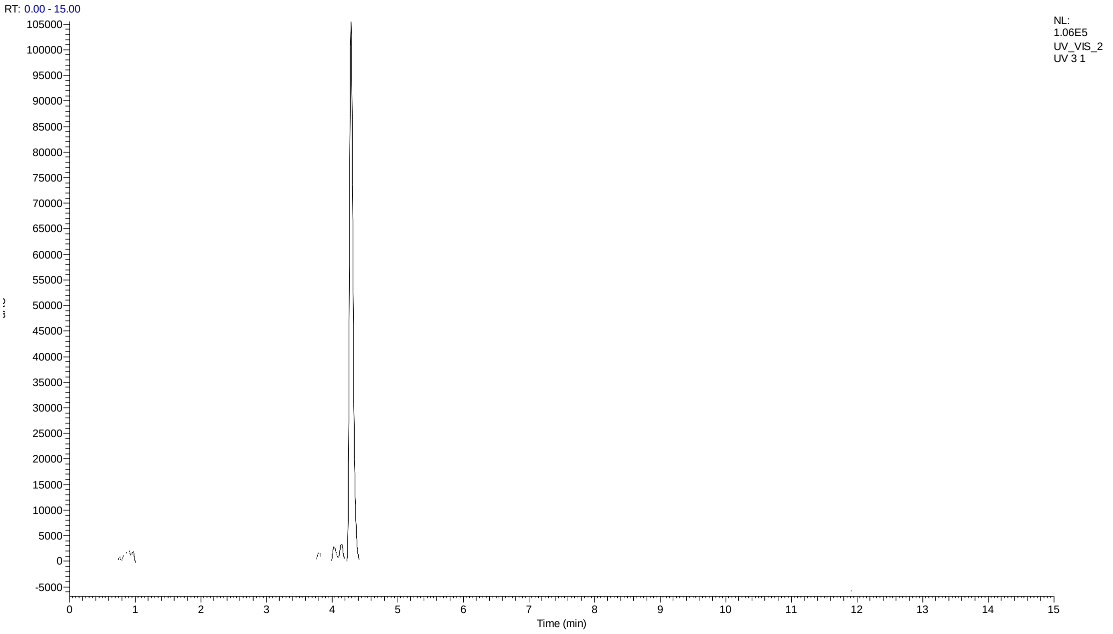

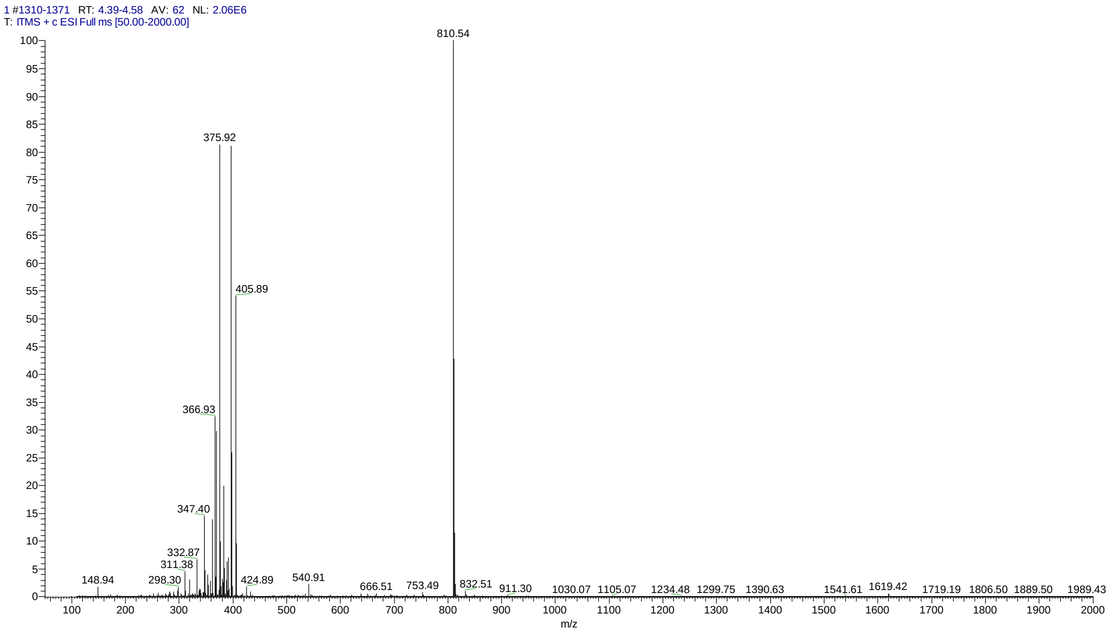

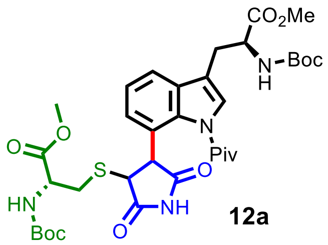

12a

The crude residue was purified by flash column chromatography on silica gel (ethyl acetate: petroleum ether= 2:3; R_f =0.25) to yield compound **12a** (36.1 mg, 82% yield). **^{1}H NMR** (500 MHz, $CDCl_3$) δ 8.75 (d, J = 217.2 Hz, 1H), 7.60 – 7.45 (m, 2H), 7.40 – 7.04 (m, 2H), 6.02 – 5.31 (m, 1H), 5.29 – 4.87 (m, 1H), 4.79 – 4.26 (m, 3H), 3.92 (s, 1H), 3.65 (d, J = 69.6 Hz, 6H), 3.47 – 3.09 (m, 4H), 1.59 – 1.33 (m, 27H). **^{13}C NMR** (126 MHz, $CDCl_3$) δ 179.11, 179.01, 174.48, 172.32, 172.28, 171.13, 171.06, 155.21, 155.14, 155.10, 132.59, 131.52, 124.35, 123.81, 123.71, 119.26, 80.18, 80.14, 56.37, 53.54, 53.42, 53.36, 53.20, 52.63, 52.47, 41.21, 39.03, 35.17, 34.51, 29.67, 28.62, 28.59,

28.32, 28.27, 28.19, 27.57. **HRMS** (ESI) *m/z* calcd for $C_{35}H_{48}N_4O_{11}SNa$ (M + Na)$^+$ 755.2933, found 755.2953.

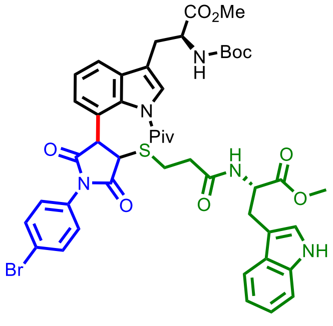

12b 93% (reaction time: 4min)

^{1}H NMR (500 MHz, DMSO-d_6) δ 10.86 (d, *J* = 13.0 Hz, 1H), 8.47 – 8.26 (m, 1H), 7.97 – 7.57 (m, 3H), 7.53 – 7.27 (m, 7H), 7.18 – 7.11 (m, 1H), 7.11 – 6.92 (m, 3H), 5.22 – 5.03 (m, 0H), 4.62 – 4.09 (m, 4H), 3.71 – 3.52 (m, 6H), 3.20 – 3.16 (m, 1H), 3.16 – 2.78 (m, 5H), 2.60 (dtd, *J* = 8.1, 6.9, 1.7 Hz, 1H), 2.49 – 2.36 (m, 2H), 2.18 (t, *J* = 8.0 Hz, 0H), 1.54 – 1.09 (m, 18H). **^{13}C NMR** (126 MHz, DMSO) δ 174.89, 174.85, 173.95, 173.00, 172.86, 172.72, 170.92, 170.70, 155.85, 136.53, 132.56, 132.46, 132.35, 132.26, 132.07, 129.73, 129.17, 127.53, 124.14, 124.09, 121.43, 121.41, 118.85, 118.43, 111.88, 109.91, 109.88, 109.80, 78.81, 55.33, 53.66, 53.56, 52.43, 52.26, 52.21, 49.07, 41.28, 35.60, 35.56, 28.88, 28.56, 28.23, 27.60, 27.57, 26.23, 20.28. **HRMS** (ESI) *m/z* calcd for $C_{47}H_{52}N_5O_{10}SBrNa$ (M + Na)$^+$ 980.2510, found 980.2523.

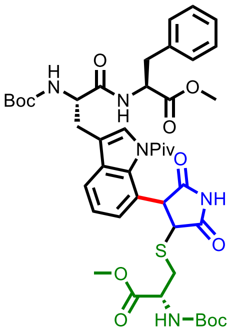

12c 85% (reaction time: 5min)

^{1}H NMR (500 MHz, DMSO-d_6) δ 11.48 (d, *J* = 144.9 Hz, 1H), 8.49 (dd, *J* = 7.7, 2.7 Hz, 1H), 7.82 (d, *J* = 24.0 Hz, 1H), 7.66 (t, *J* = 7.5 Hz, 1H), 7.45 – 7.18 (m, 8H), 7.00 (d, *J* = 8.5 Hz, 1H), 4.85 (s, 0H), 4.55 (td, *J* = 8.9, 8.1, 4.8 Hz, 1H), 4.42 – 4.29 (m, 2H), 4.29 – 4.05 (m, 2H), 3.67 – 3.47 (m, 6H), 3.24 – 2.90 (m, 5H), 2.88 – 2.79 (m, 1H), 1.52 – 1.38 (m, 10H), 1.36 (d, *J* = 10.7 Hz, 8H), 1.28 (d, *J* = 3.5 Hz, 9H). **^{13}C NMR** (126 MHz, DMSO) δ 177.24, 172.32, 172.27, 171.75, 155.69, 155.60, 137.45, 132.36, 131.36, 129.54, 129.43, 129.32, 128.73, 128.21, 127.78, 127.04, 123.87, 123.43, 119.34, 119.23, 78.95, 78.71, 78.57, 54.02, 53.77, 53.71, 53.65, 52.52, 52.36, 41.89, 41.36, 37.09, 29.46, 28.56, 28.25, 27.36. **HRMS** (ESI) *m/z* calcd for $C_{44}H_{57}N_5O_{12}SNa$ (M + Na)$^+$ 902.3617, found 902.3652.

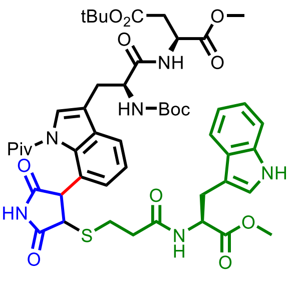

12d 90% (reaction time: 4min)

^{1}H NMR (500 MHz, DMSO-d_6) δ 11.43 (d, *J* = 137.6 Hz, 1H), 10.85 (d, *J* = 2.3 Hz, 1H), 8.51 (t, *J* = 7.8 Hz, 1H), 8.46 – 8.34 (m, 1H), 7.84 (d, *J* = 27.5 Hz, 1H), 7.71 – 7.57 (m, 1H), 7.53 – 7.44 (m, 1H), 7.38 – 7.22 (m, 3H), 7.15 (d, *J* = 2.5 Hz, 1H), 7.11 – 7.03 (m, 2H), 6.98 (ddt, *J* = 8.1, 7.0, 1.2 Hz, 1H), 5.02 – 4.79 (m, 0H), 4.76 – 4.62 (m, 1H), 4.51 (td, *J* = 7.7, 5.0 Hz, 1H), 4.40 – 4.12 (m, 3H), 3.64 (d, *J* = 1.9 Hz, 3H), 3.60 – 3.51 (m, 3H), 3.18 – 2.97 (m, 3H), 2.95 – 2.81 (m, 3H), 2.76 (ddd, *J* = 16.2, 5.8, 3.8 Hz, 1H), 2.65 (ddd, *J* = 16.2, 7.2, 3.3 Hz, 1H), 2.45 (q, *J* = 6.9 Hz, 2H), 1.41 (s, 18H), 1.34 – 1.22 (m, 9H). **^{13}C NMR** (126 MHz, DMSO) δ 179.69, 179.66, 177.34, 172.74, 172.37, 172.25, 171.57, 170.71, 169.48, 155.66, 136.54, 132.32, 127.54, 124.13, 124.09, 123.47, 121.41, 119.22, 118.86, 118.44, 111.88, 109.82, 87.07, 81.12, 78.56, 53.83, 53.77, 53.67, 52.65, 52.21, 50.60, 49.25, 49.17, 41.30, 37.52, 35.46, 34.09, 29.47, 28.94, 28.54, 28.11, 27.66, 27.30. **HRMS** (ESI) *m/z* calcd for $C_{49}H_{62}N_6O_{13}SNa$ (M + Na)$^+$ 997.3988, found 997.3989.

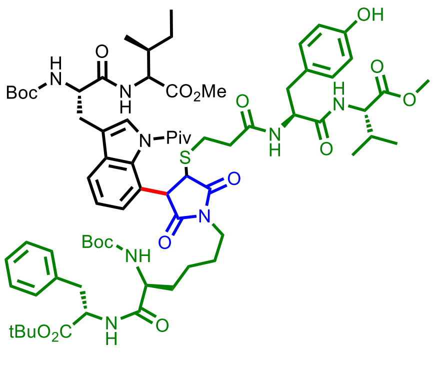

12e 89% (reaction time: 6min)

^{1}H NMR (500 MHz, DMSO-d_6) δ 9.20 – 9.12 (m, 1H), 8.24 (t, J = 6.8 Hz, 1H), 8.17 (d, J = 8.2 Hz, 1H), 8.05 (dd, J = 7.2, 3.6 Hz, 2H), 7.96 – 7.55 (m, 2H), 7.41 – 7.14 (m, 7H), 7.13 – 6.95 (m, 3H), 6.87 – 6.74 (m, 1H), 6.67 – 6.56 (m, 2H), 4.91 (s, 0H), 4.60 – 4.50 (m, 1H), 4.49 – 4.38 (m, 1H), 4.38 – 4.17 (m, 3H), 4.14 (dd, J = 8.1, 6.4 Hz, 1H), 3.89 (s, 1H), 3.68 – 3.58 (m, 6H), 3.07 – 2.77 (m, 7H), 2.61 (dd, J = 13.4, 8.7 Hz, 1H), 2.45 – 2.29 (m, 2H), 2.01 (dq, J = 13.5, 6.8 Hz, 1H), 1.80 (d, J = 26.1 Hz, 1H), 1.57 – 1.19 (m, 47H), 0.90 – 0.83 (m, 12H). **^{13}C NMR** (126 MHz, DMSO) δ 172.47, 172.34, 172.28, 172.18, 172.05, 170.83, 170.83, 170.60, 170.31, 156.17, 155.71, 155.66, 152.24, 144.47, 137.55, 135.35, 130.53, 129.68, 129.59, 128.56, 128.15, 126.91, 125.71, 125.61, 123.80, 115.22, 99.99, 81.10, 78.60, 78.46, 57.90, 56.81, 54.41, 54.36, 54.30, 53.60, 52.18, 52.13, 52.09, 37.32, 37.27, 36.96, 35.64, 35.60, 32.08, 32.06, 30.35, 29.47, 28.87, 28.83, 28.64, 28.55, 28.40, 28.31, 27.96, 27.40, 27.36, 25.19, 25.19, 24.77, 23.33, 23.27, 19.36, 18.75, 15.82, 11.59. **HRMS** (ESI) *m/z* calcd for $C_{74}H_{104}N_8O_{18}SNa$ $(M + Na)^+$ 1447.7082, found 1447.7079.

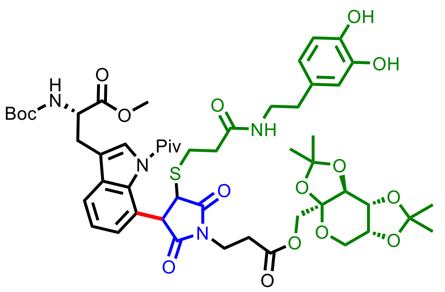

12f 93% (reaction time: 3min)

^{1}H NMR (500 MHz, DMSO-d_6) δ 8.74 (s, 1H), 8.64 (s, 1H), 7.88 (d, J = 21.2 Hz, 2H), 7.59 (d, J = 7.9 Hz, 1H), 7.44 (d, J = 8.1 Hz, 1H), 7.38 – 7.17 (m, 2H), 6.62 (d, J = 8.0 Hz, 1H), 6.56 (d, J = 2.1 Hz, 1H), 6.41 (dd, J = 8.0, 2.1 Hz, 1H), 4.88 (d, J = 31.8 Hz, 0H), 4.59 (dt, J = 7.8, 3.3 Hz, 1H), 4.40 – 4.08 (m, 6H), 3.95 (d, J = 11.5 Hz, 1H), 3.80 – 3.69 (m, 3H), 3.66 (d, J = 4.4 Hz, 3H), 3.61 (dd, J = 13.0, 3.3 Hz, 1H), 3.21 – 3.06 (m, 3H), 2.95 (ddd, J = 44.5, 13.9, 8.4 Hz, 3H), 2.72 (dd, J = 15.4, 8.0 Hz, 2H), 2.47 (t, J = 7.6 Hz, 2H), 2.35 (t, J = 7.5 Hz, 2H), 1.51 – 1.27 (m, 30H). **^{13}C NMR** (126 MHz, DMSO) δ 173.00, 170.39, 170.35, 170.24, 155.84, 145.50, 143.96, 132.16, 130.61, 126.23, 124.01, 123.60, 119.60, 119.26, 116.35, 115.92, 108.60, 108.57, 101.34, 99.98, 99.46, 79.43, 78.78, 75.14, 70.44, 70.34, 69.71, 65.05, 61.00, 53.64, 52.43, 41.94, 41.12, 35.76, 35.14, 35.09, 31.75, 31.68, 29.47, 28.90, 28.57, 26.64, 26.30, 26.16, 26.15, 25.47, 25.44, 24.42. **HRMS** (ESI) *m/z* calcd for $C_{52}H_{68}N_4O_{17}SNa$ $(M + Na)^+$ 1075.4192, found 1075.4188.

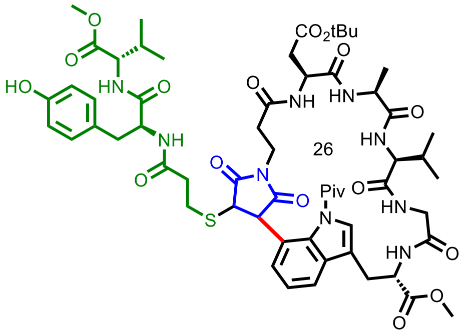

12g 87% (reaction time: 5min)

^{1}H NMR (500 MHz, DMSO-d_6) δ 9.22 – 9.12 (m, 1H), 8.48 – 7.98 (m, 4H), 7.97 – 7.68 (m, 2H), 7.67 – 7.39 (m, 2H), 7.39 – 7.16 (m, 2H), 7.15 – 6.78 (m, 3H), 6.69 – 6.56 (m, 2H), 5.02 – 4.61 (m, 1H), 4.59 – 4.00 (m, 6H), 3.99 – 3.62 (m, 7H), 3.62 – 3.55 (m, 3H), 3.35 – 3.14 (m, 2H), 3.12 – 2.86 (m, 1H), 2.86 – 2.75 (m, 3H), 2.71 – 2.54 (m, 2H), 2.50 – 2.39 (m, 2H), 2.40 – 2.10 (m, 3H), 2.08 – 1.92 (m, 2H), 1.53 – 1.26 (m, 18H), 1.08 – 0.40 (m, 16H). **^{13}C NMR** (126 MHz, DMSO) δ 179.70, 179.58, 179.12, 175.73, 175.66, 173.78, 172.47, 172.21, 172.19, 172.03, 171.21, 170.43, 170.37, 170.32, 170.17, 169.80, 169.75, 156.16, 131.79, 130.54, 130.49, 128.14, 119.10,

115.21, 85.26, 80.85, 80.62, 57.91, 54.28, 52.60, 52.10, 49.20, 48.61, 42.31, 41.49, 37.24, 37.14, 35.53, 30.33, 29.36, 28.96, 28.86, 28.47, 28.15, 28.07, 27.56, 19.71, 19.35, 18.76, 18.72, 18.32, 18.28. **HRMS** (ESI) *m/z* calcd for $C_{60}H_{81}N_9O_{17}SNa$ $(M + Na)^+$ 1254.5363, found 1254.5365.

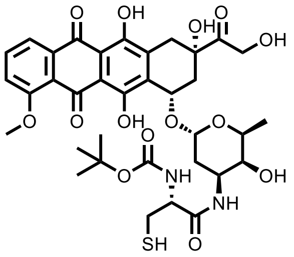

^{1}H NMR (500 MHz, $CDCl_3$) δ 13.91 (s, 1H), 13.14 (s, 1H), 7.98 (d, *J* = 7.7 Hz, 1H), 7.76 (t, *J* = 8.1 Hz, 1H), 7.37 (d, *J* = 8.4 Hz, 1H), 6.73 (d, *J* = 8.2 Hz, 1H), 5.48 (t, *J* = 6.0 Hz, 2H), 5.22 (d, *J* = 3.4 Hz, 1H), 4.76 (d, *J* = 2.0 Hz, 2H), 4.57 (s, 1H), 4.25 (s, 1H), 4.14 (q, *J* = 6.4 Hz, 2H), 4.06 (s, 3H), 3.69 (s, 1H), 3.24 – 3.17 (m, 1H), 3.00 (ddd, *J* = 13.1, 7.8, 4.8 Hz, 1H), 2.91 (d, *J* = 19.3 Hz, 1H), 2.70 (ddd, *J* = 13.8, 10.1, 6.1 Hz, 1H), 2.33 (d, *J* = 14.6 Hz, 1H), 2.15 (dd, *J* = 14.6, 4.1 Hz, 2H), 1.84 (dtd, *J* = 29.0, 13.7, 5.0 Hz, 2H), 1.53 (dd, *J* = 10.0, 7.8 Hz, 1H), 1.43 (s, 9H), 1.37 (s, 1H), 1.30 (d, *J* = 6.6 Hz, 3H). **^{13}C NMR** (126 MHz, $CDCl_3$) δ 213.84, 186.87, 186.47, 169.40, 162.59, 160.96, 156.15, 155.52, 135.71, 135.36, 133.59, 120.73, 119.78, 118.43, 111.46, 111.28, 100.61, 80.79, 76.54, 69.59, 68.86, 67.21, 65.52, 56.61, 45.68, 36.50, 35.62, 33.86, 31.46, 29.63, 28.26, 26.89, 16.84. **HRMS** (ESI) *m/z* calcd for $C_{35}H_{42}N_2O_{14}SNa$ $(M + Na)^+$ 769.2249, found 769.2248.

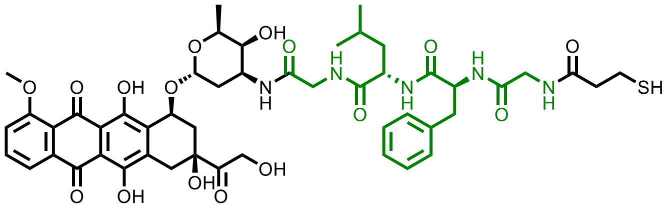

15

^{1}H NMR (400 MHz, MeOD) δ 7.64 – 7.40 (m, 2H), 7.26 (dd, *J* = 11.3, 5.6 Hz, 1H), 7.16 (dq, *J* = 14.9, 7.6 Hz, 5H), 5.42 – 5.25 (m, 1H), 4.75 (s, 3H), 4.65 (s, 2H), 4.57 (dd, *J* = 8.6, 5.6 Hz, 2H), 4.23 (dt, *J* = 9.8, 5.3 Hz, 2H), 4.18 – 4.06 (m, 1H), 3.86 (d, *J* = 3.4 Hz, 3H), 3.82 (d, *J* = 3.2 Hz, 1H), 3.73 (t, *J* = 15.7 Hz, 2H), 3.64 (d, *J* = 2.6 Hz, 1H), 3.10 (dd, *J* = 14.0, 5.4 Hz, 1H), 2.89 (dd, *J* = 14.3, 8.3 Hz, 2H), 2.67 (t, *J* = 6.8 Hz, 3H), 2.49 (t, *J* = 7.3 Hz, 2H), 2.30 (d, *J* = 14.0 Hz, 1H), 2.13 – 1.96 (m, 2H), 1.80 – 1.68 (m, 1H), 1.56 (tq, *J* = 11.4, 8.4, 7.6 Hz, 3H), 1.28 (td, *J* = 6.7, 5.6, 3.0 Hz, 6H), 0.85 (dd, *J* = 13.7, 5.3 Hz, 6H). **^{13}C NMR** (101 MHz, MeOD) δ 205.27, 177.82, 165.51, 165.04, 164.42, 162.56, 161.24, 152.56, 147.54, 146.30, 128.49, 127.55, 126.28, 125.87, 125.46, 120.79, 120.10, 118.40, 111.44, 110.89, 110.57, 102.57, 102.36, 92.61, 67.84, 61.51, 60.28, 59.08, 56.31, 47.61, 46.74, 44.47, 37.99, 34.31, 31.48, 31.19, 28.80, 27.56, 24.47, 21.22, 20.60, 16.20, 14.00, 12.38, 11.52, 7.90. **HRMS** (ESI) *m/z* calcd for $C_{49}H_{59}N_5O_{16}SNa$ $(M + Na)^+$ 1028.3570, found 1028.3575.

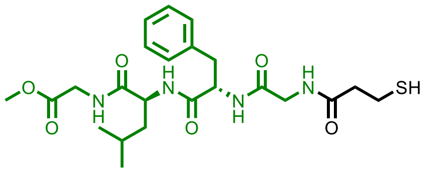

^{1}H NMR (500 MHz, DMSO-d_6) δ 8.19 (q, *J* = 6.3 Hz, 2H), 8.10 (d, *J* = 8.2 Hz, 1H), 8.03 (d, *J* = 8.1 Hz, 1H), 7.27 – 7.16 (m, 5H), 4.51 (td, *J* = 8.9, 4.3 Hz, 1H), 4.30 (td, *J* = 8.4, 6.4 Hz, 1H), 3.88 – 3.76 (m, 2H), 3.72 (dd, *J* = 16.5, 5.8 Hz, 1H), 3.60 (s, 3H), 3.55 (d, *J* = 5.7 Hz, 1H), 3.03 (dd, *J* = 13.9, 4.3 Hz, 1H), 2.76 (dd, *J* = 13.9, 9.4 Hz, 1H), 2.62 (q, *J* = 7.1 Hz, 2H), 2.41 (t, *J* = 6.9 Hz, 2H), 2.29 (t, *J* = 8.1 Hz, 1H), 1.65 – 1.54 (m, 1H), 1.48 (ddd, *J* = 8.2, 5.9, 2.0 Hz, 2H), 0.88 (d, *J* = 6.6 Hz, 3H), 0.83 (d, *J* = 6.5 Hz, 3H). **^{13}C NMR** (126 MHz, DMSO) δ 172.98, 171.53, 171.26, 170.57, 169.43, 137.97, 129.64, 128.53, 126.78, 54.33, 52.18, 51.43, 42.44, 41.19, 40.99, 37.77, 24.48, 23.38, 22.03, 20.31. **HRMS** (ESI) *m/z* calcd for $C_{32}H_{45}N_5O_8Na$ $(M + Na)^+$ 517.2091, found 517.2089.

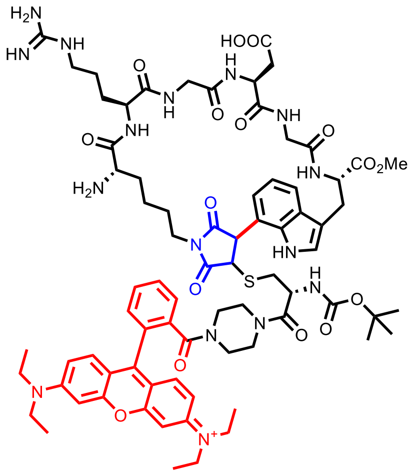

MS (ESI) m/z (relative intensity) 762.75 (64) [(M^++H^+)/2], 1523.64 (3) [M^+].

LCMS

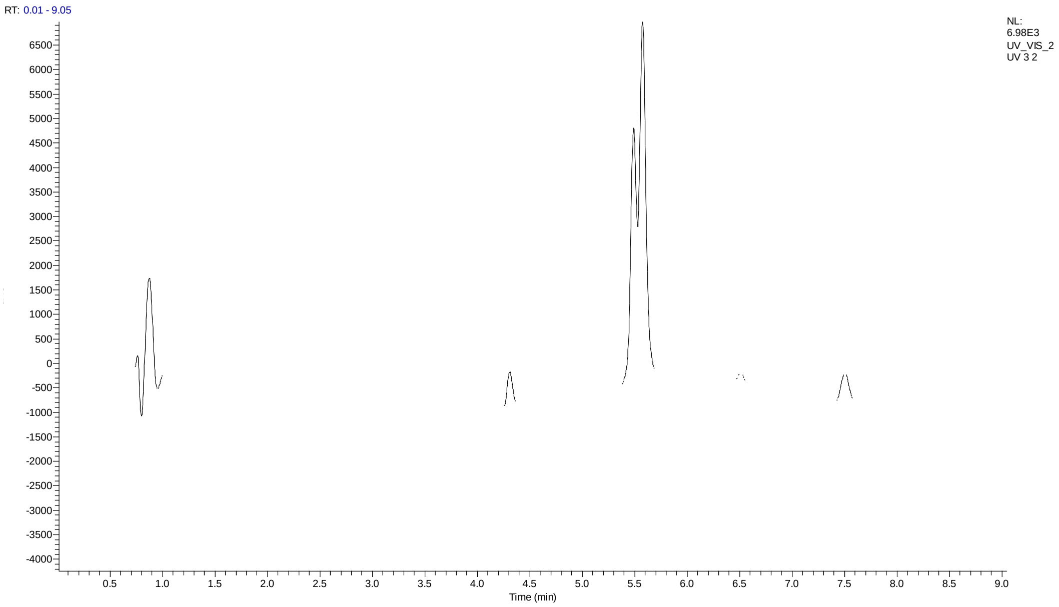

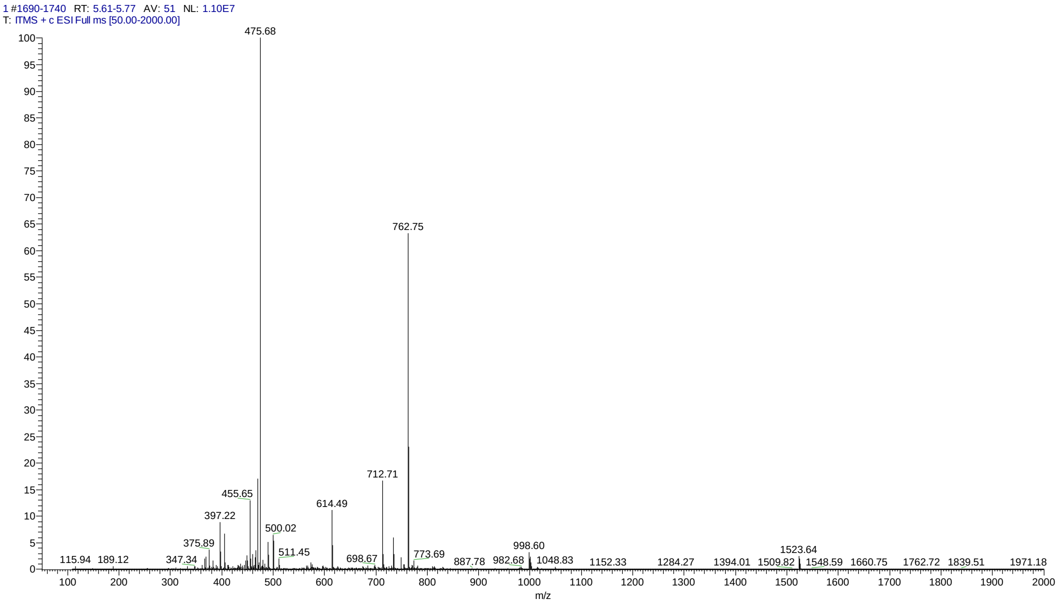

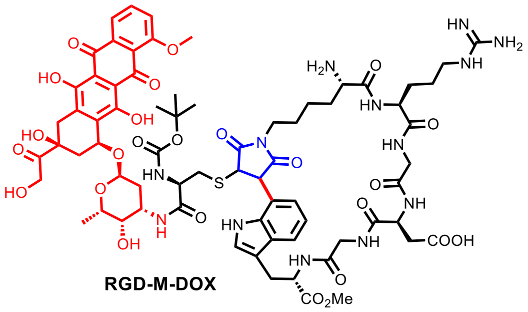

RGD-M-DOX

MS (ESI) m/z (relative intensity) 778.99 (62) [(M+2H$^+$)/2], 1556.68 (25) [M+H$^+$].

LCMS

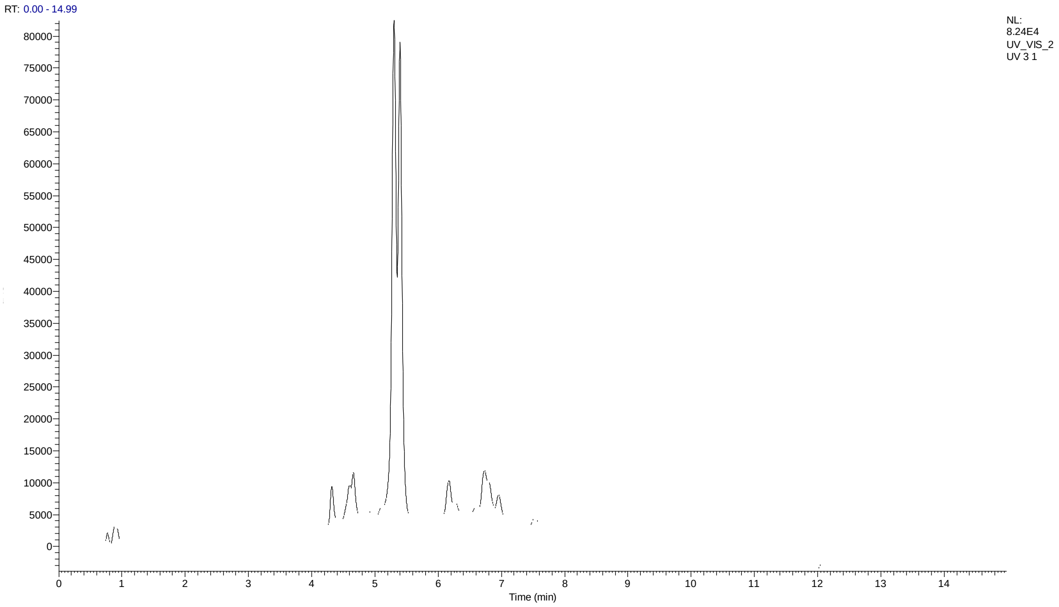

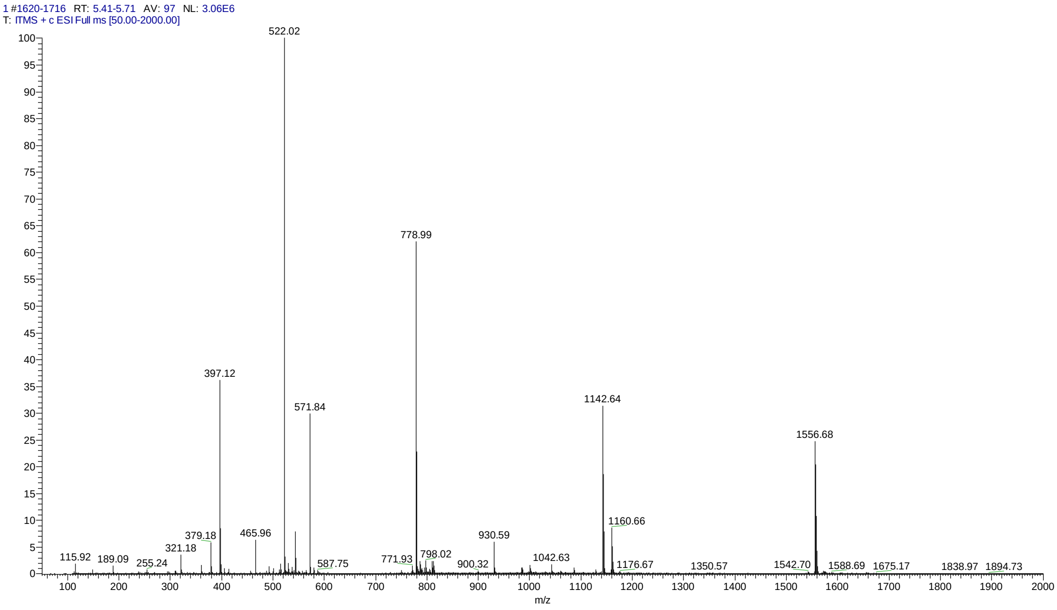

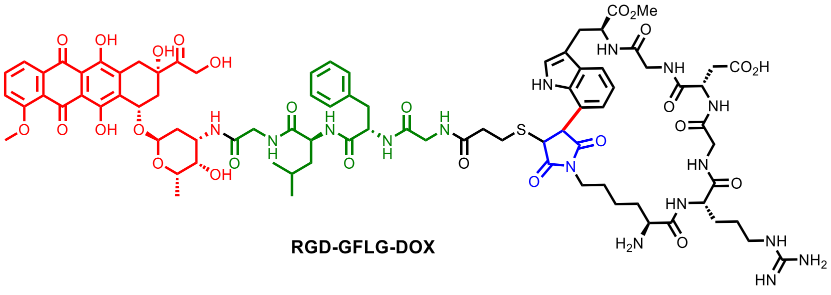

RGD-GFLG-DOX

MS (ESI) m/z (relative intensity) 908.72 (100) [(M+2H$^+$)/2].

LCMS

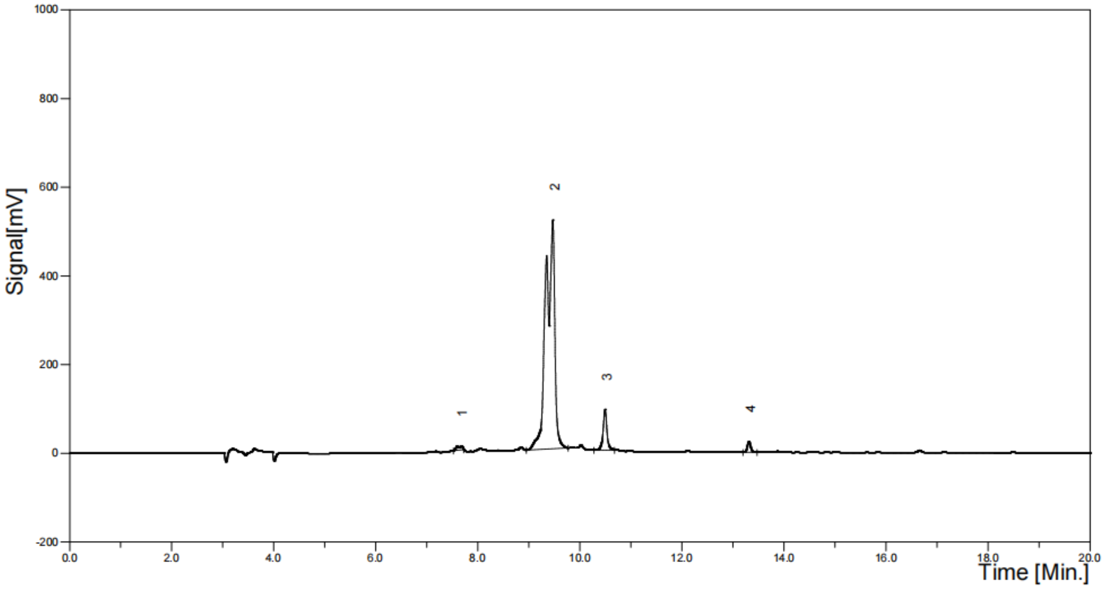

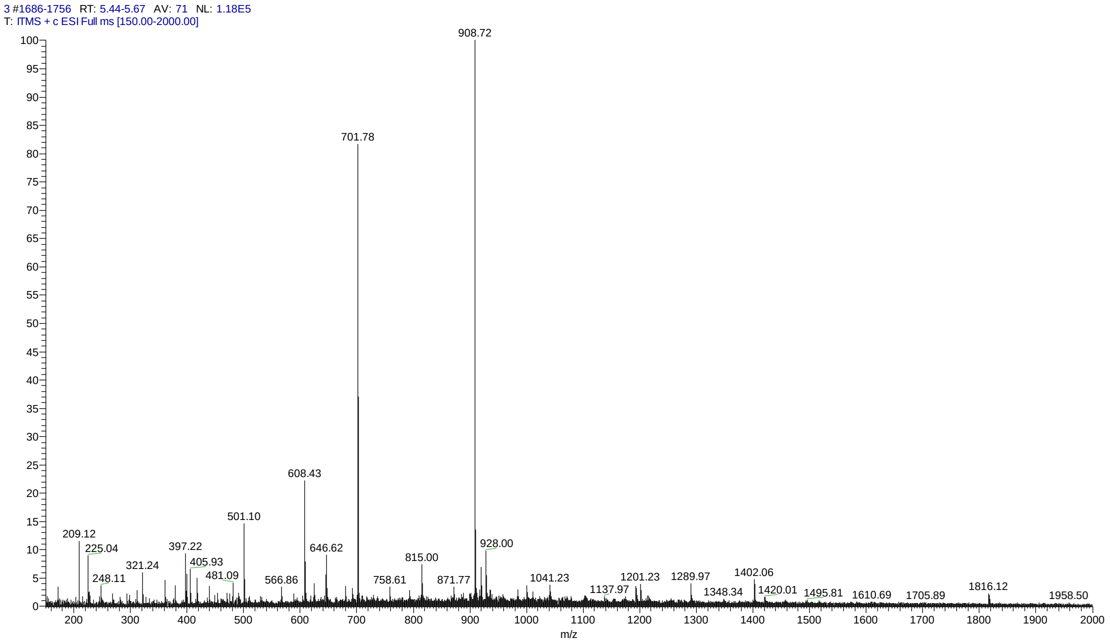

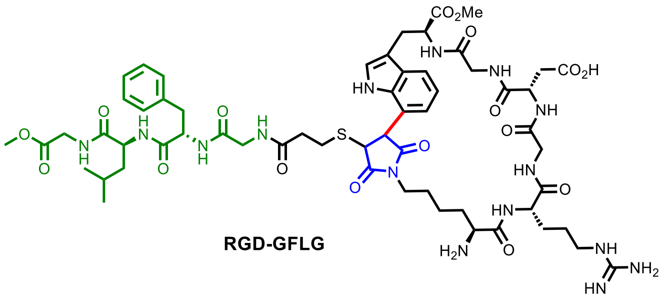

MS (ESI) m/z (relative intensity) 653.15 (100) [(M+2H$^+$)/2], 1304.72 (28) [M+H$^+$].

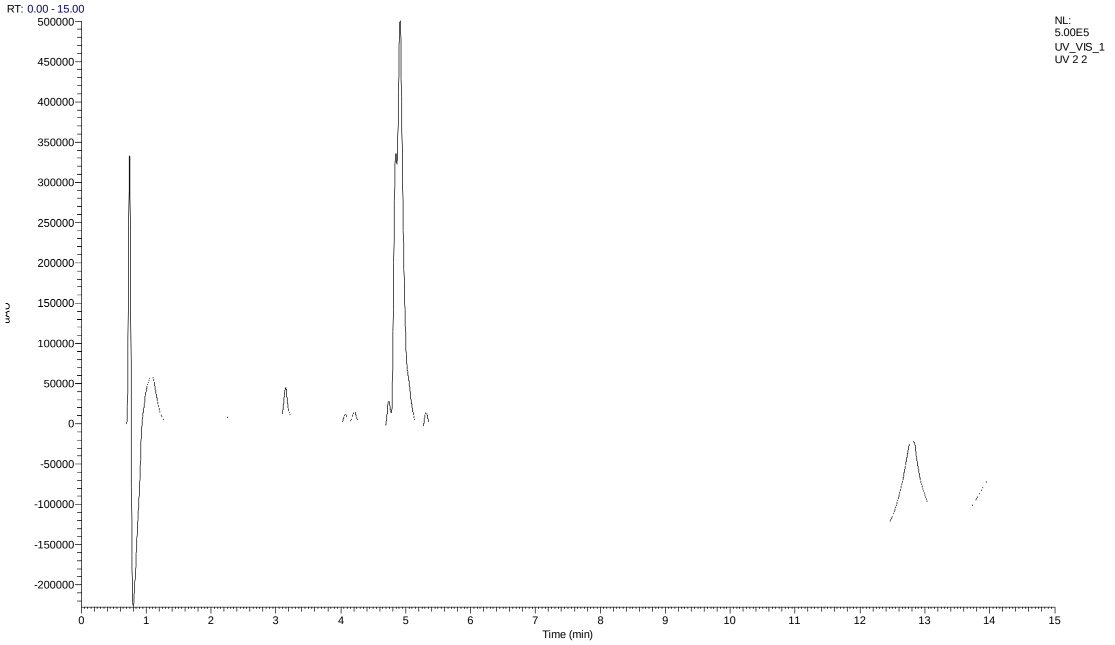

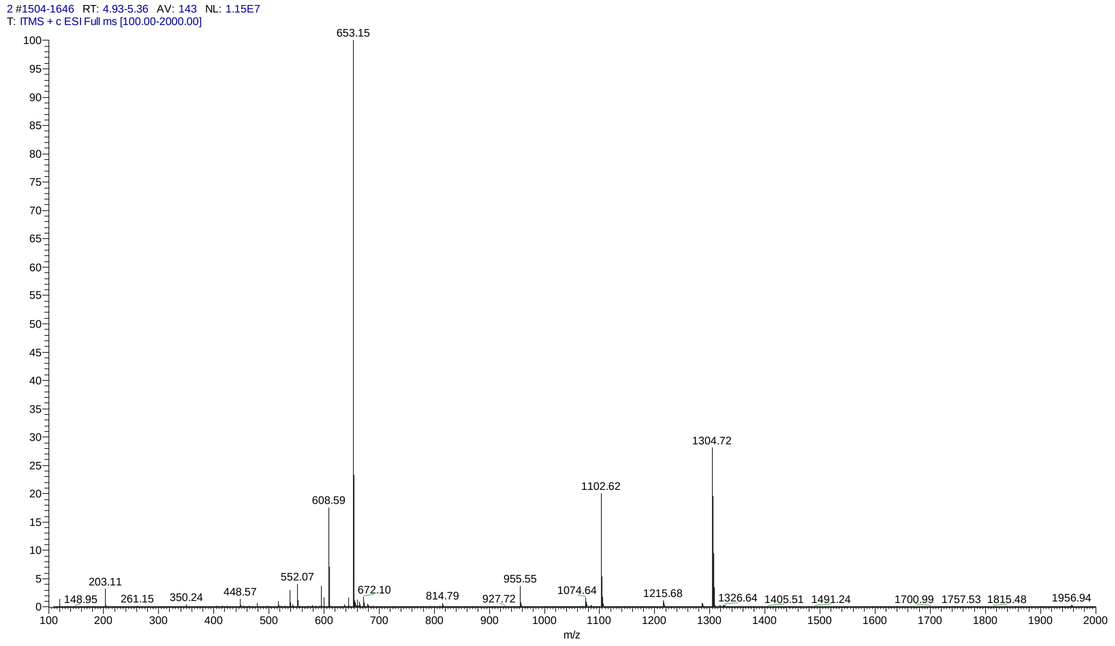

R. ^{1}H NMR and ^{13}C NMR of products

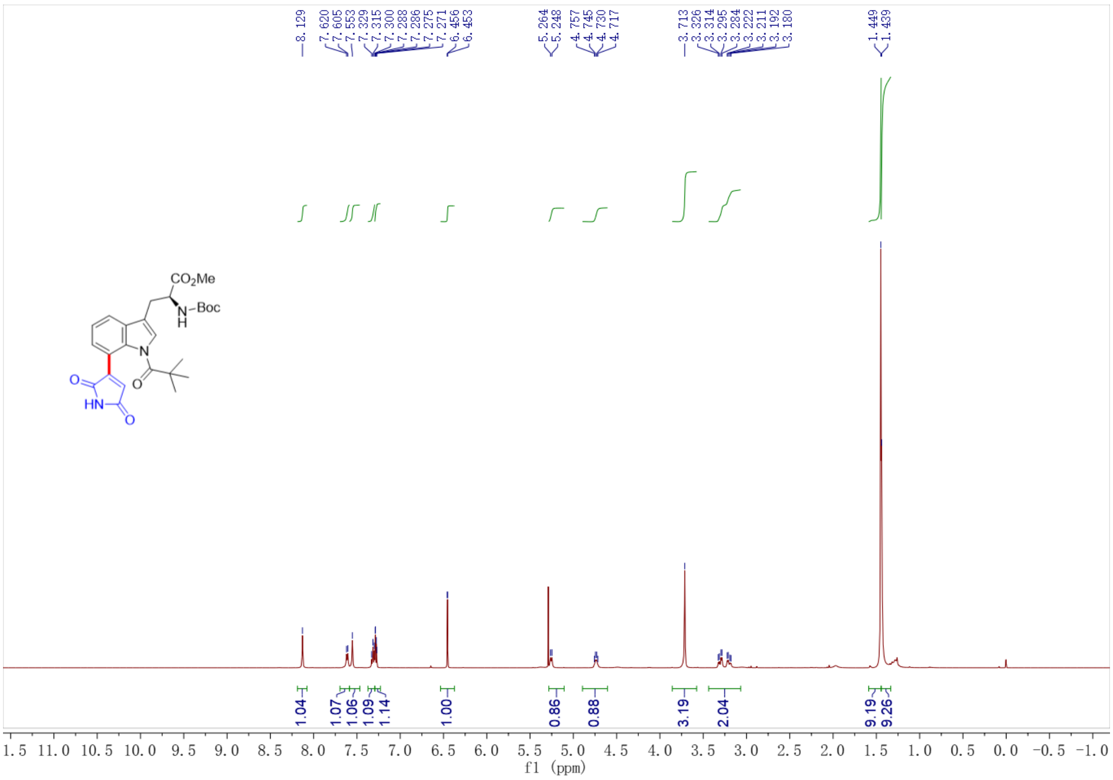

^{1}H NMR (500 MHz, $CDCl_3$) spectrum of **3a**

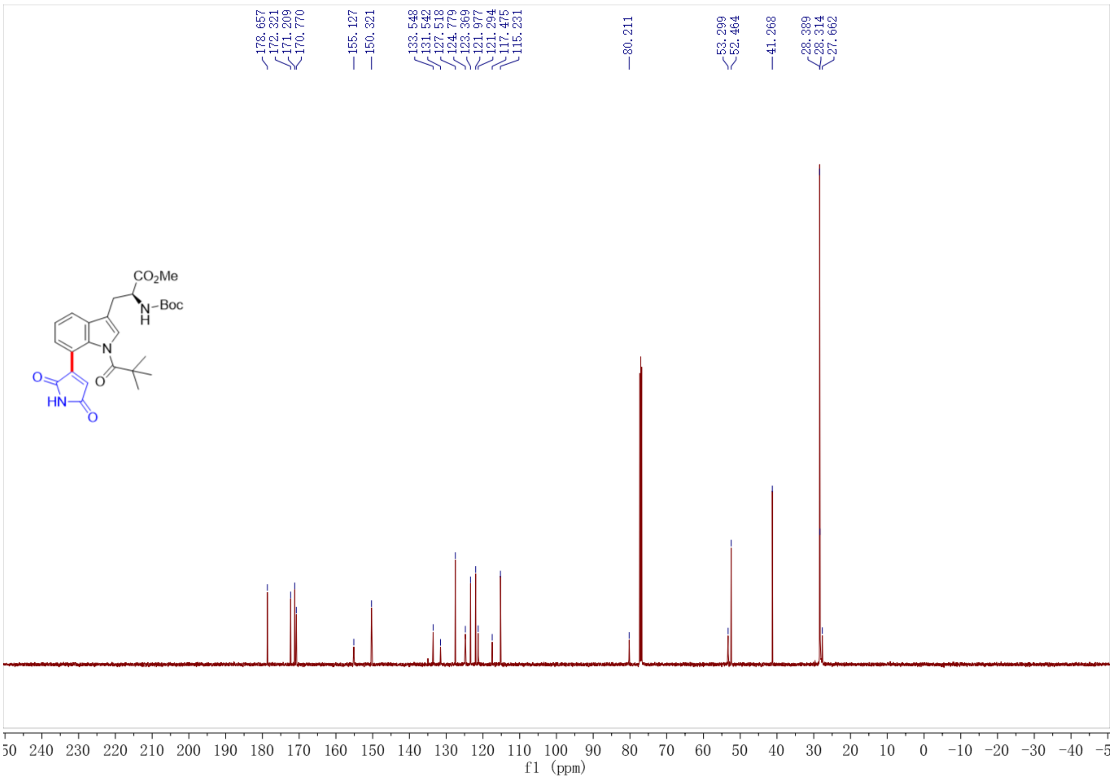

^{1}C NMR (126 MHz, $CDCl_3$) spectrum of **3a**

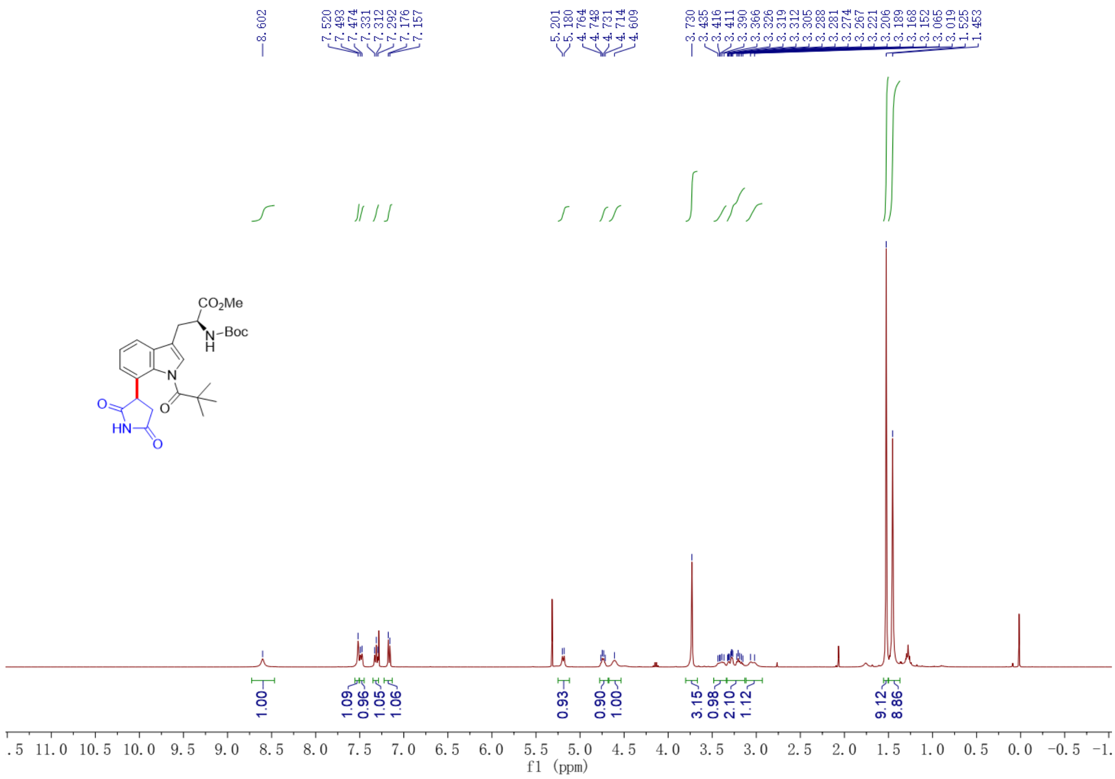

^{1}H NMR (400 MHz, $CDCl_3$) spectrum of **3aa**

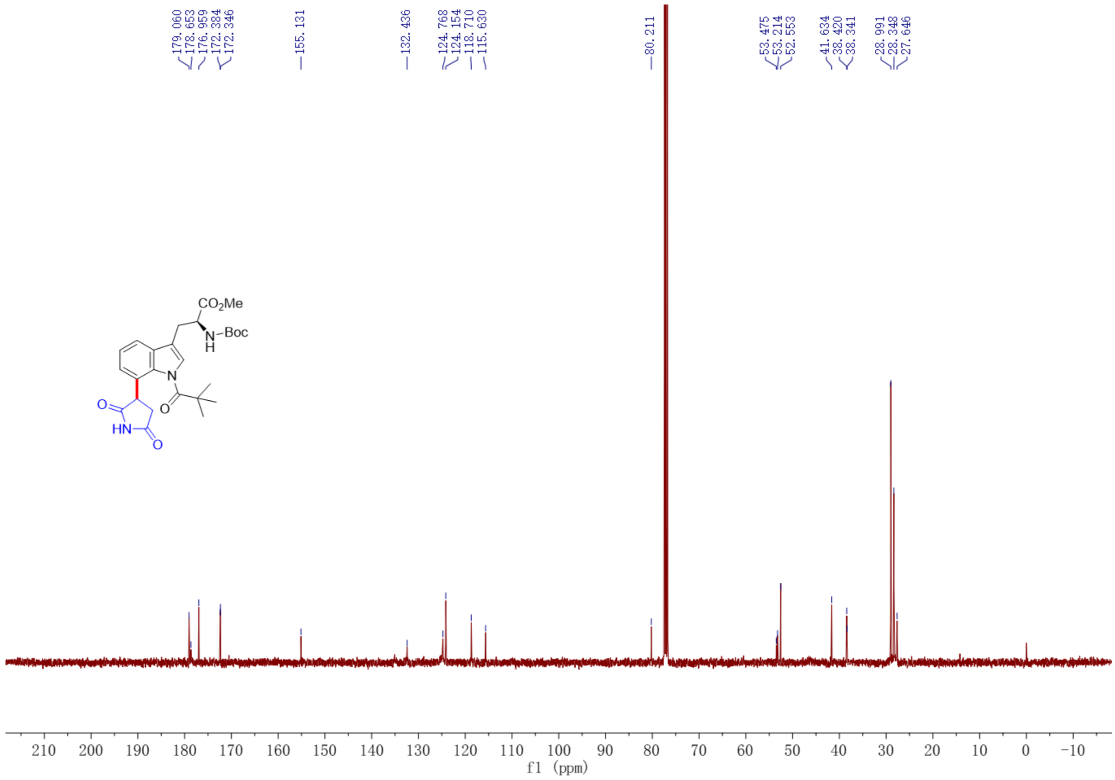

[1]C NMR (101MHz, $CDCl_3$) spectrum of **3aa**

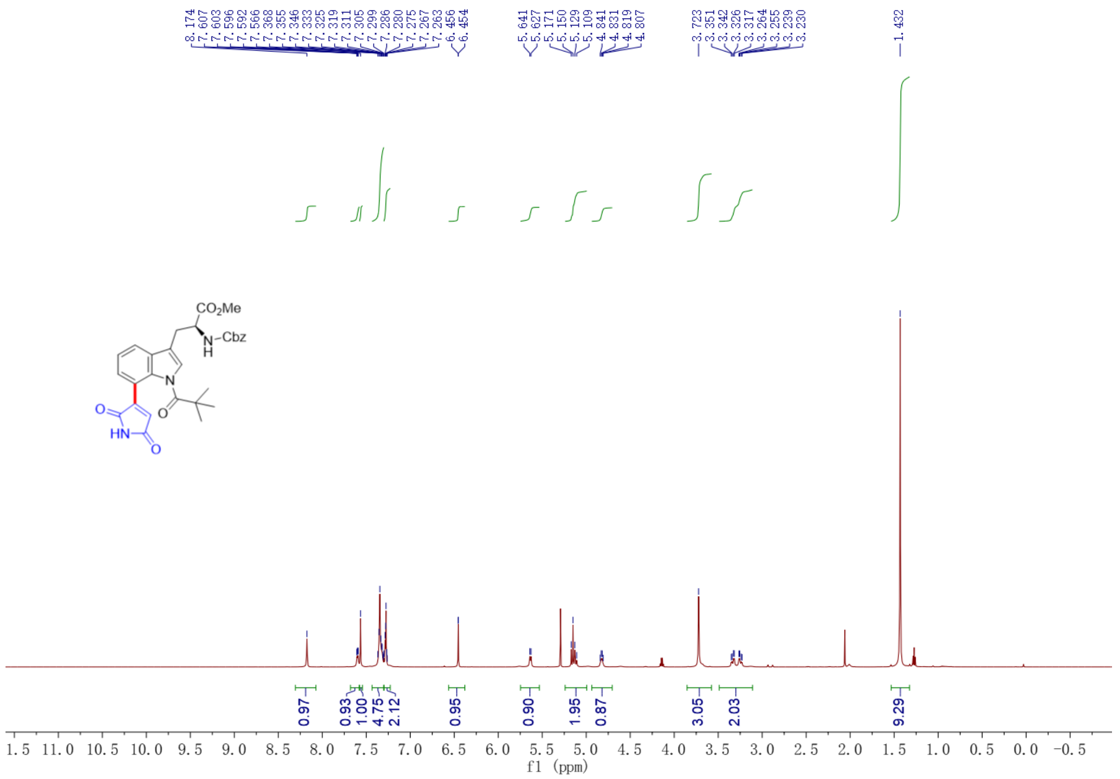

^{1}H NMR (600 MHz, $CDCl_3$) spectrum of **3b**

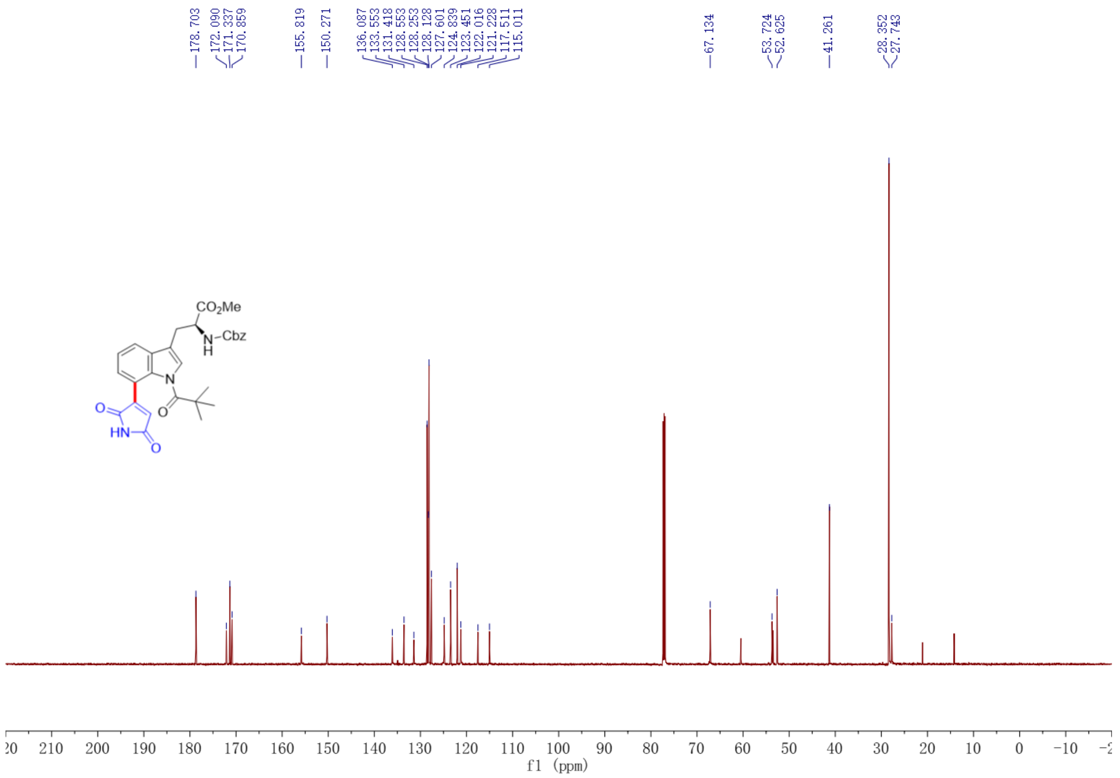

[1]C NMR (151 MHz, $CDCl_3$) spectrum of **3b**

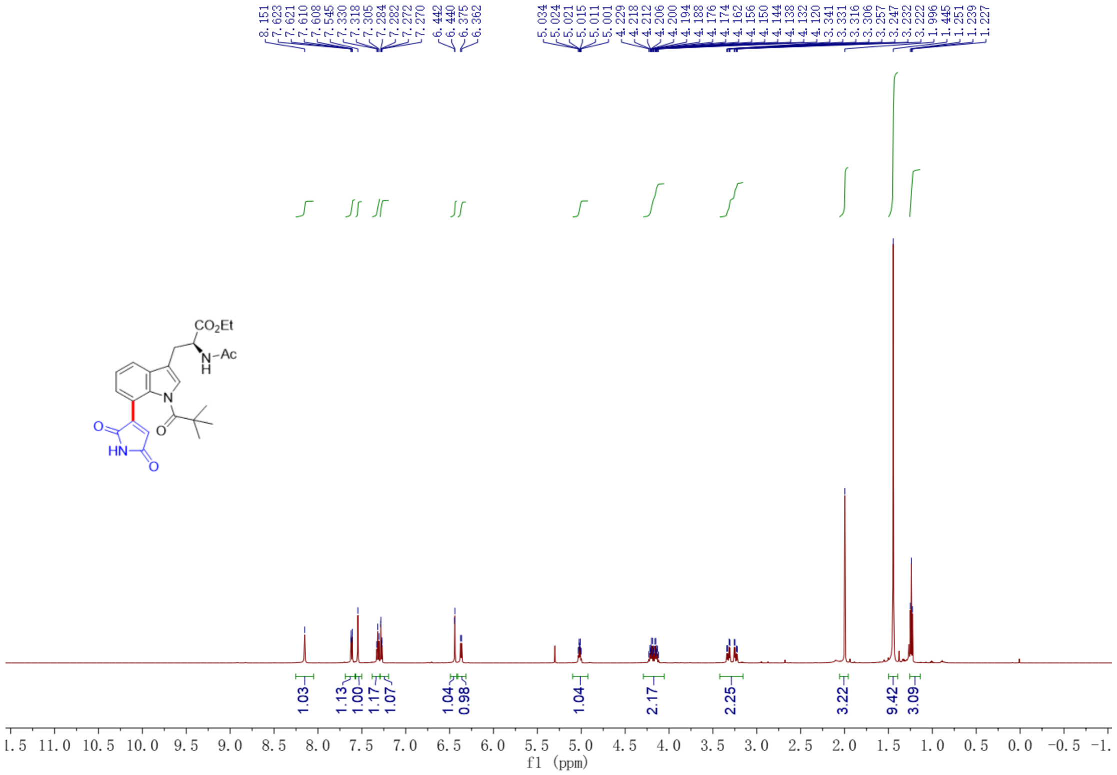

^{1}H NMR (600 MHz, $CDCl_3$) spectrum of **3c**

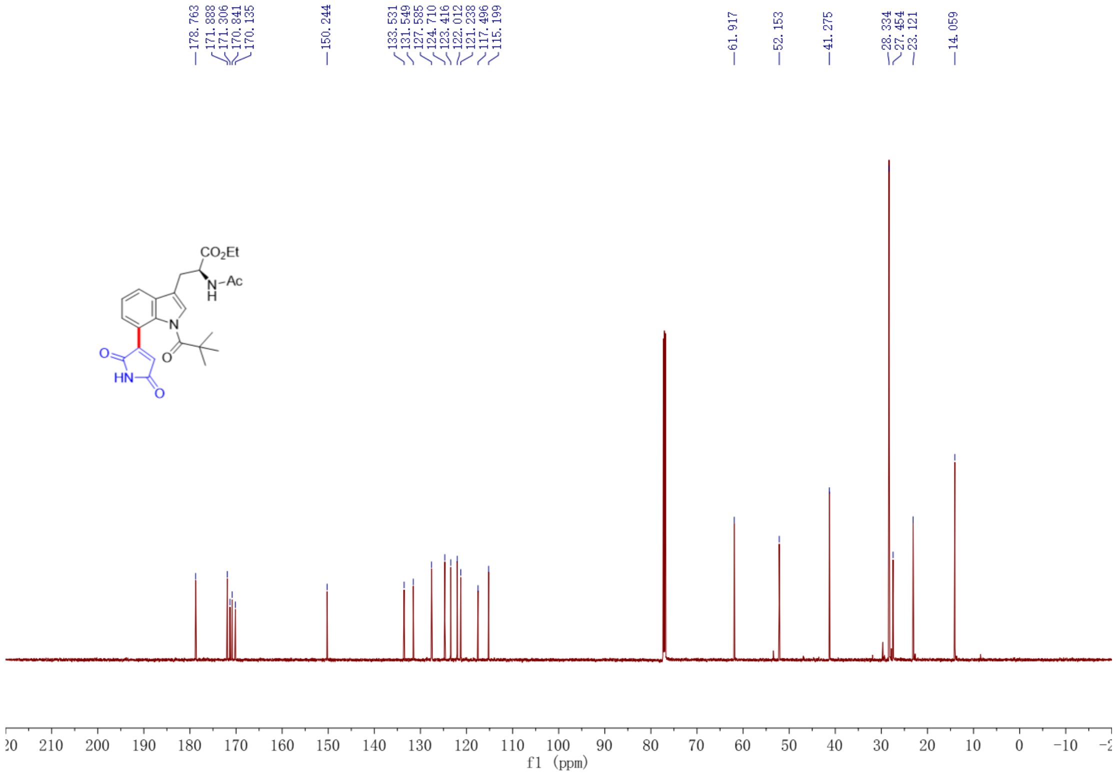

^{1}C NMR (151 MHz, DMSO) spectrum of **3c**

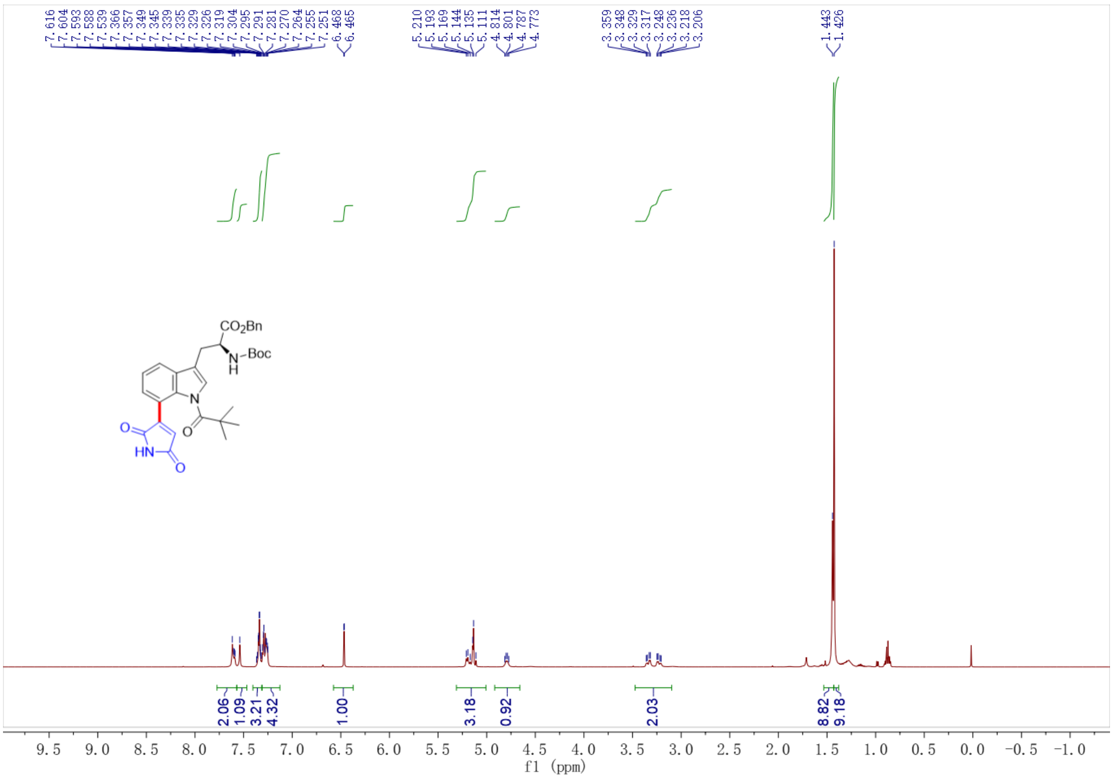

^{1}H NMR (500 MHz, $CDCl_3$) spectrum of **3d**

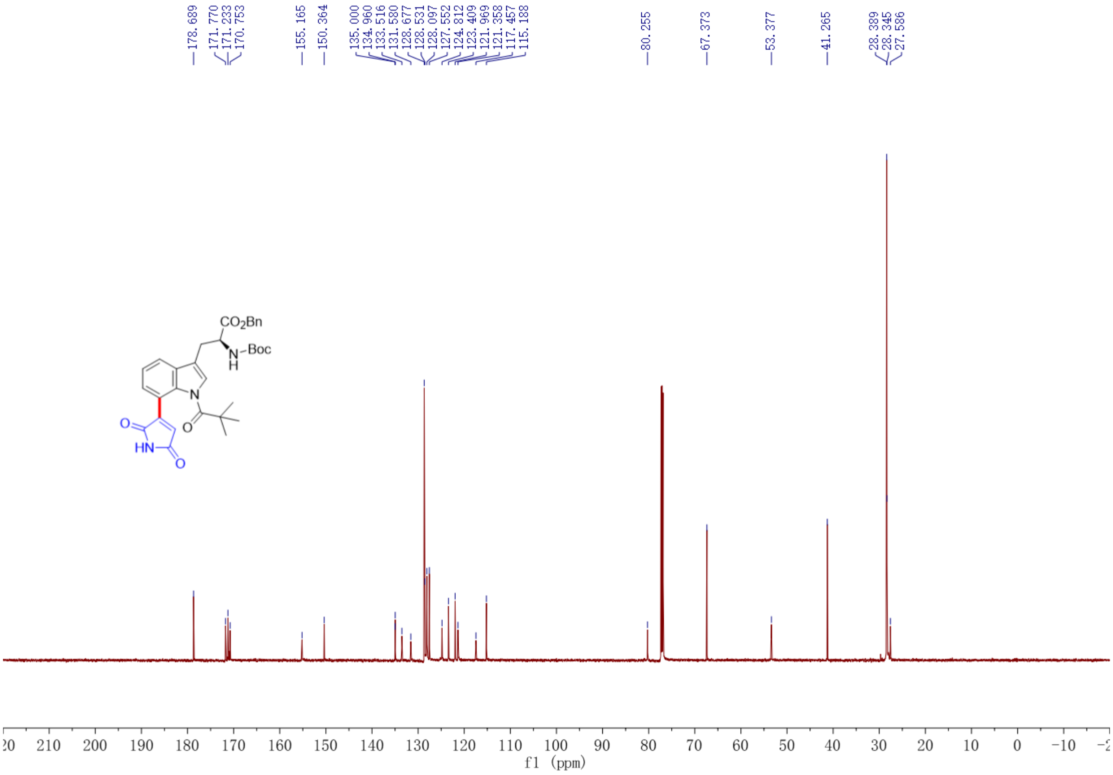

^{1}C NMR (126 MHz, $CDCl_3$) spectrum of **3d**

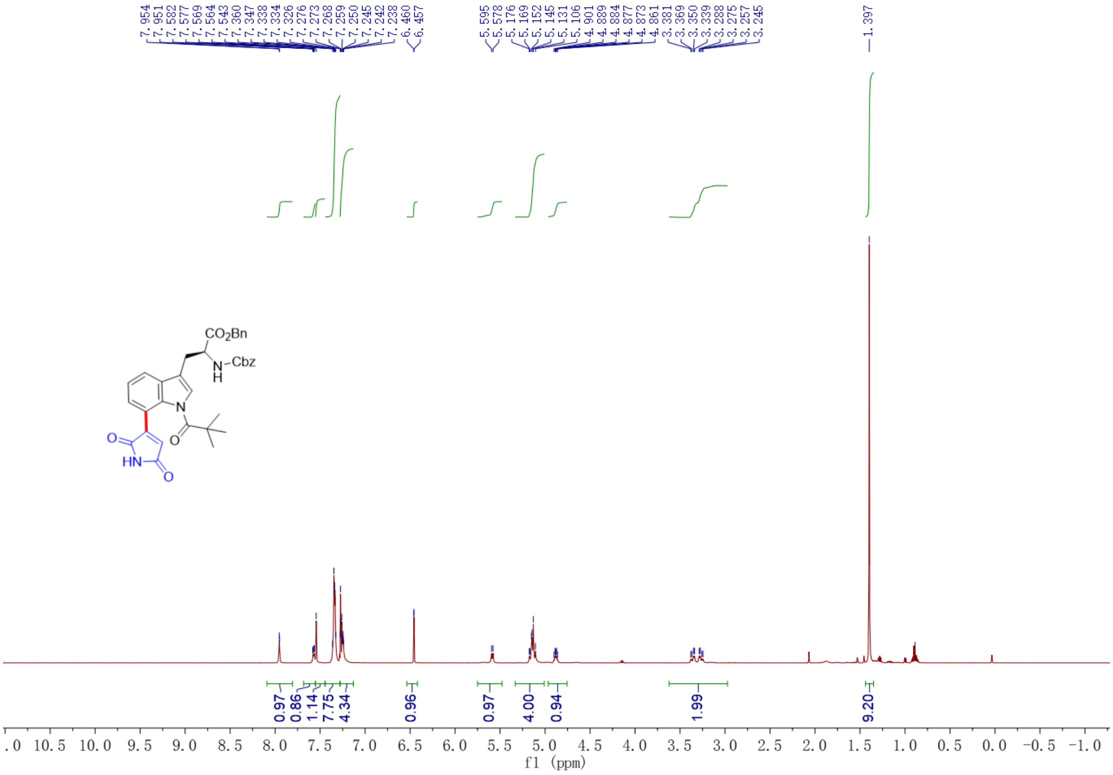

^{1}H NMR (500 MHz, CDCl$_3$) spectrum of **3e**

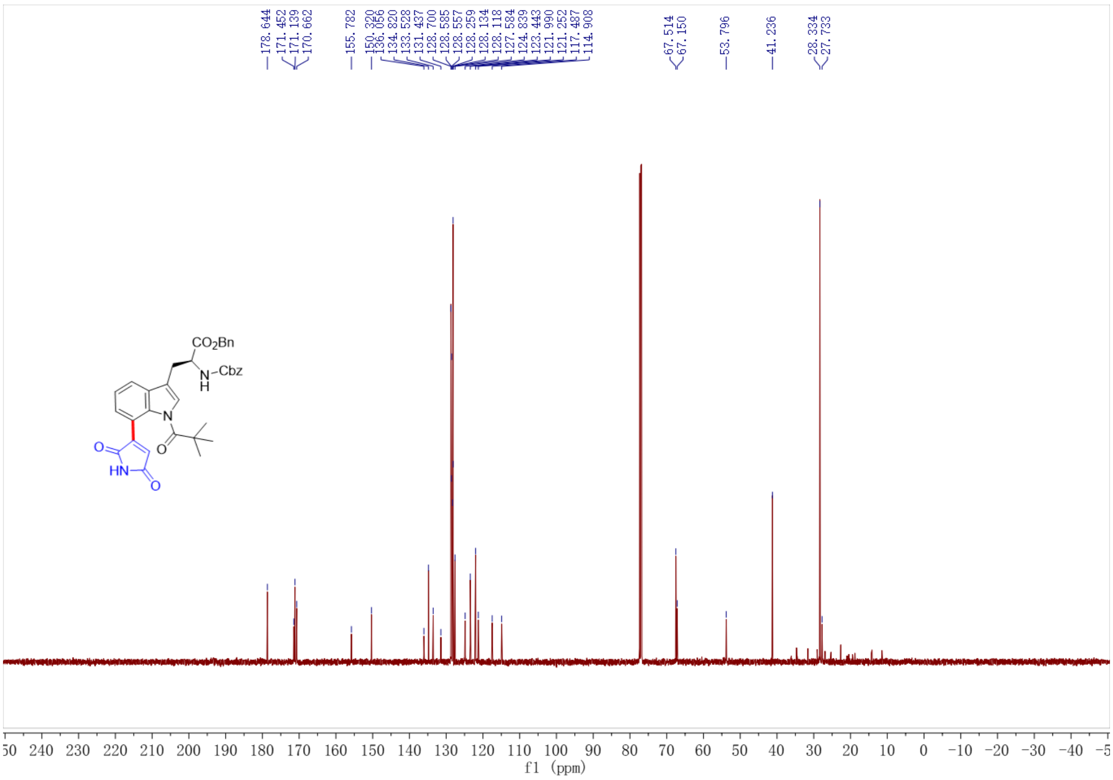

^{1}C NMR (126 MHz, CDCl$_3$) spectrum of **3e**

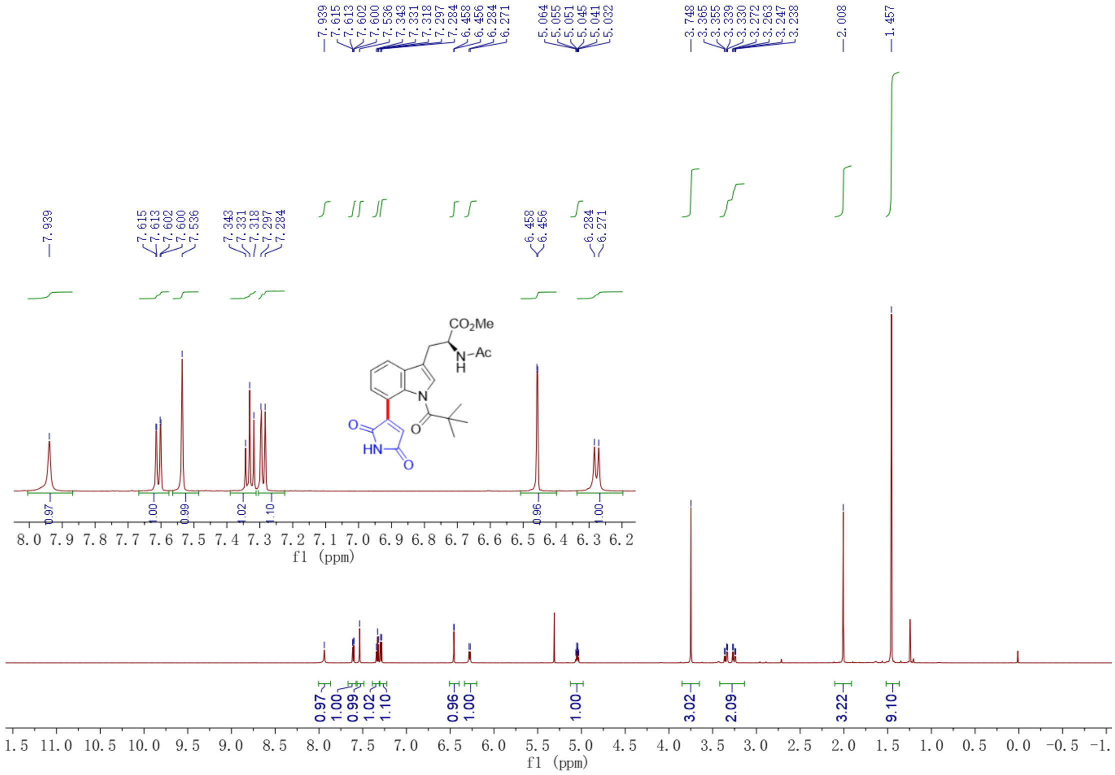

^{1}H NMR (600 MHz, $CDCl_3$) spectrum of **3f**

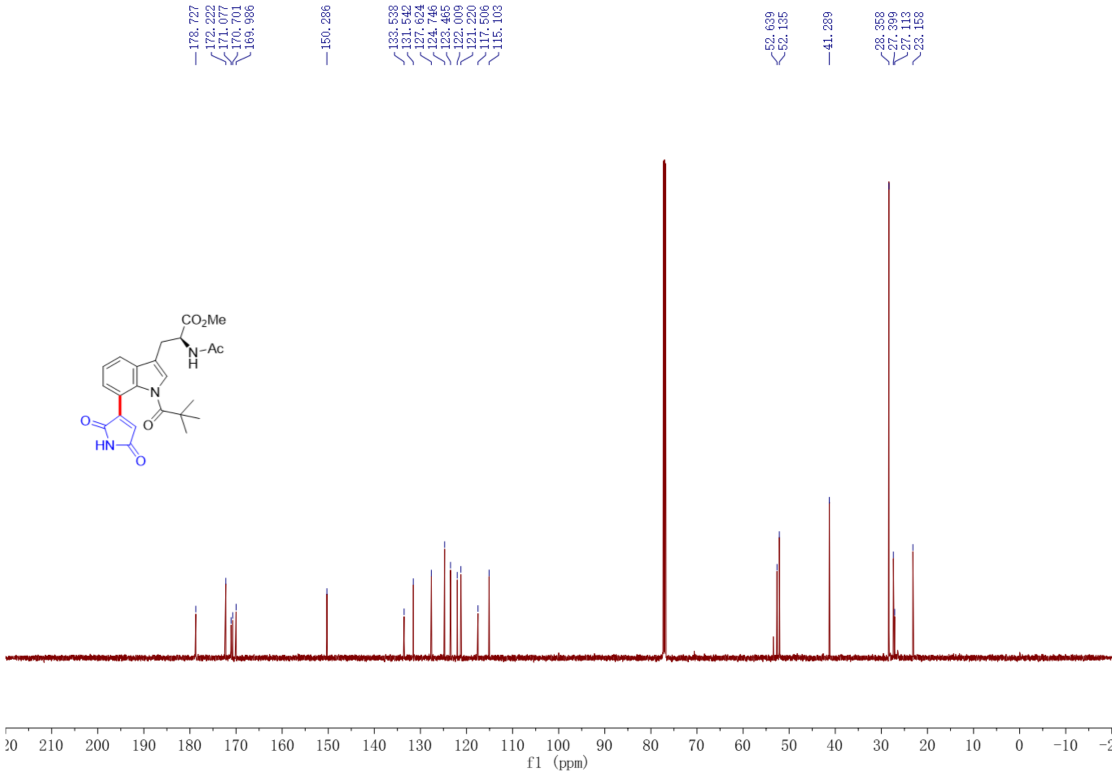

^{1}C NMR (151 MHz, $CDCl_3$) spectrum of **3f**

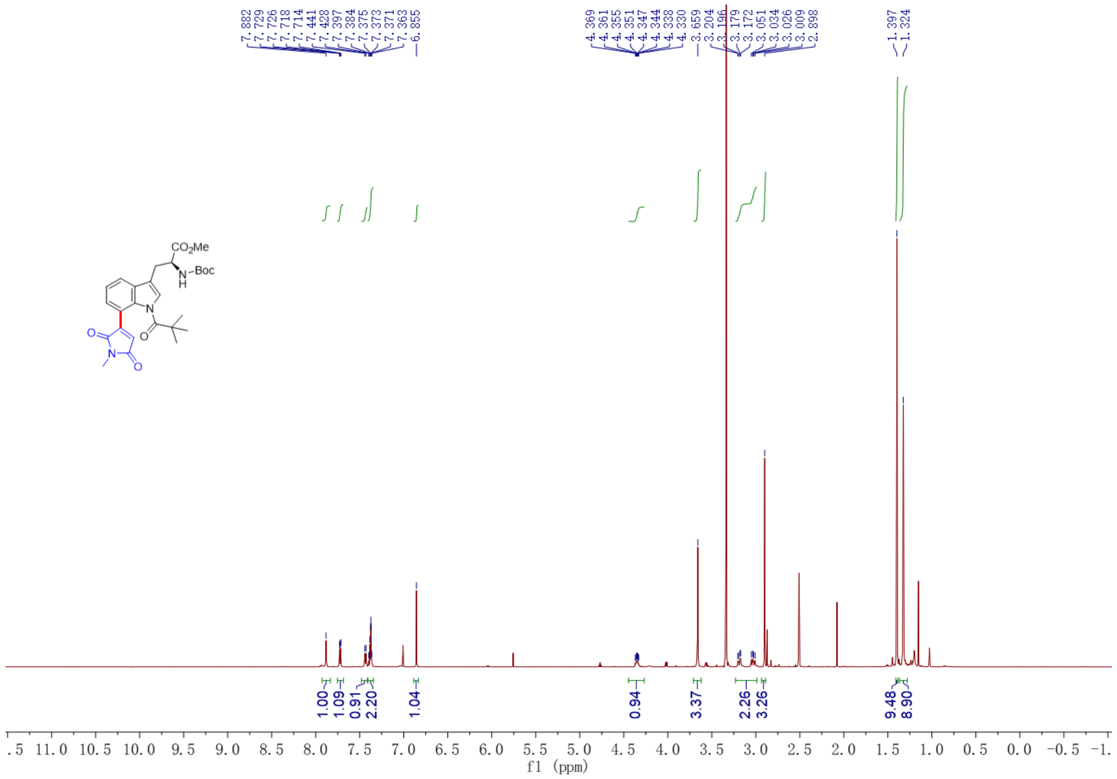

^{1}H NMR (600 MHz, DMSO) spectrum of **3g**

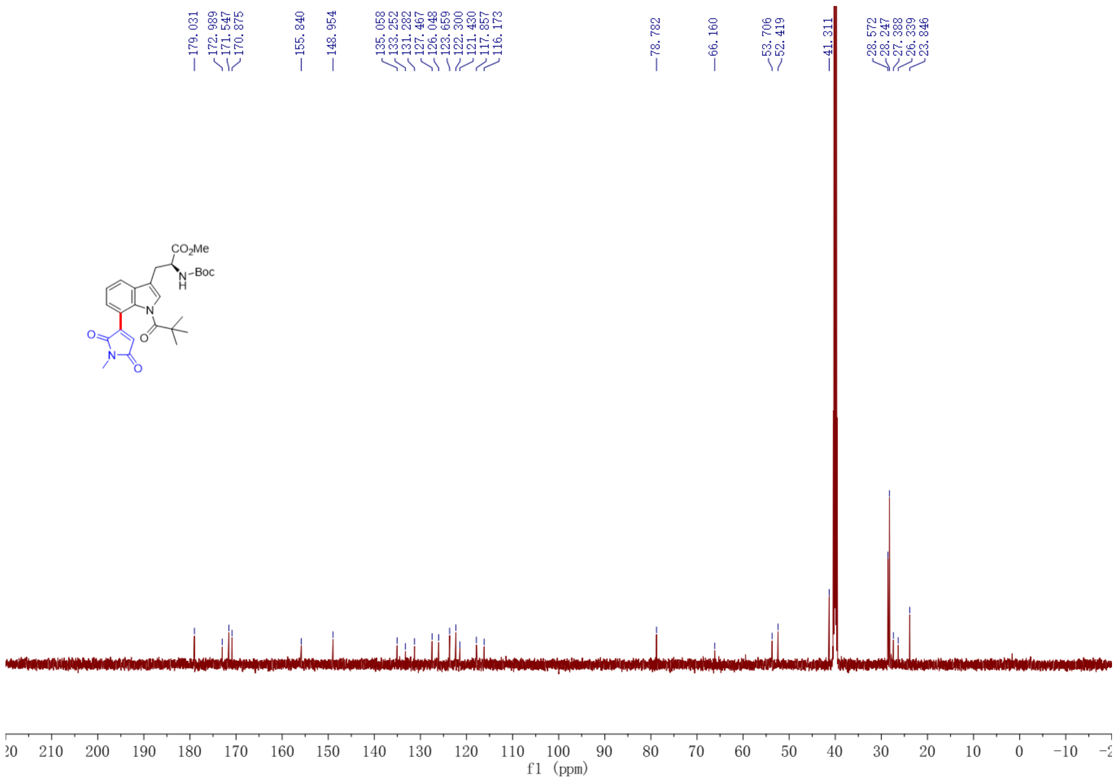

^{1}C NMR (151MHz, DMSO) spectrum of **3g**

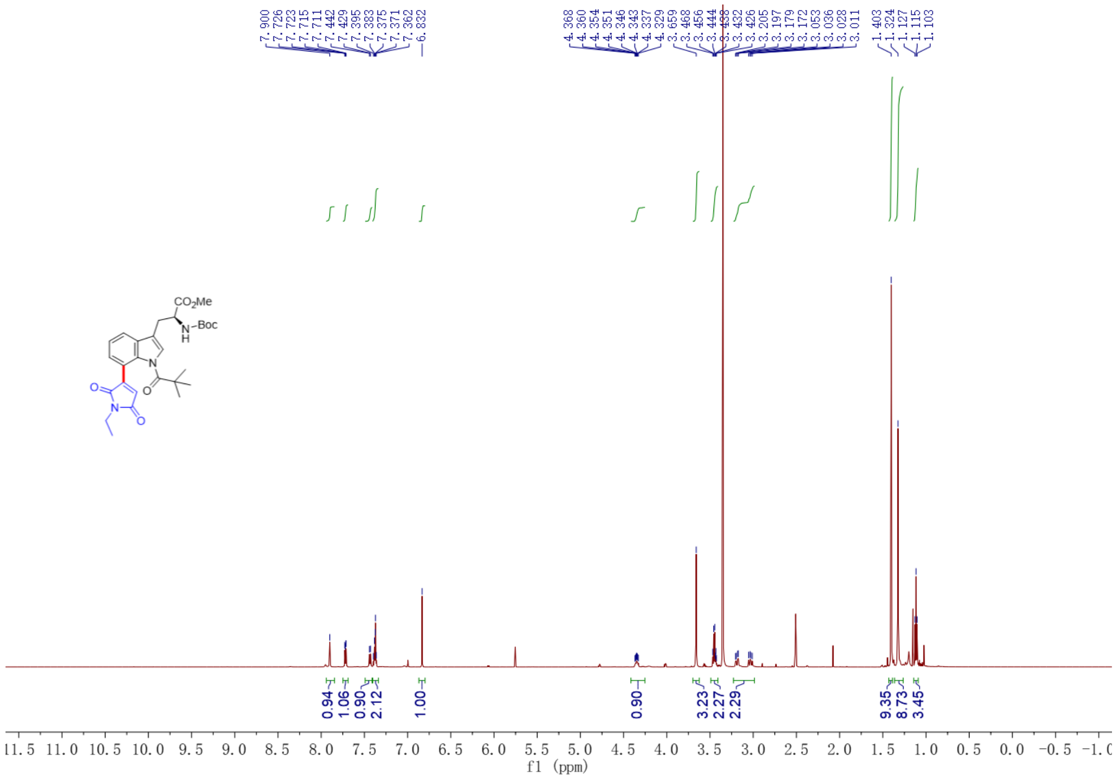

^{1}H NMR (600 MHz, DMSO) spectrum of **3h**

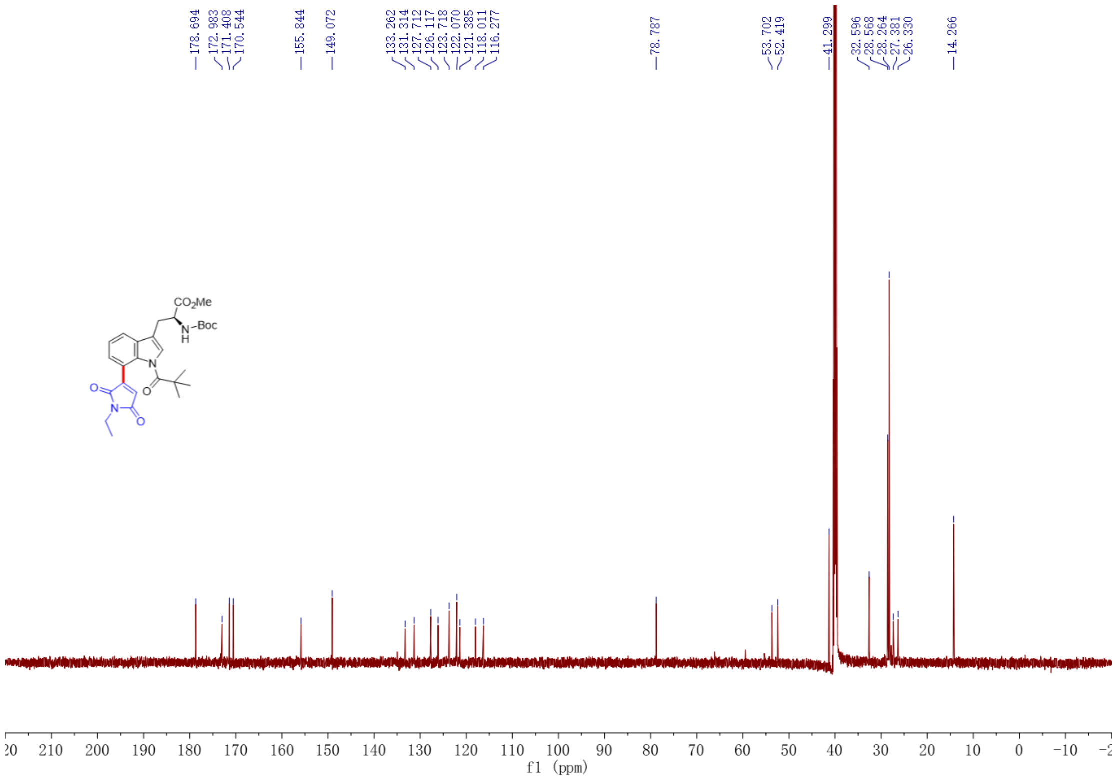

[1]C NMR (151 MHz, DMSO) spectrum of **3h**

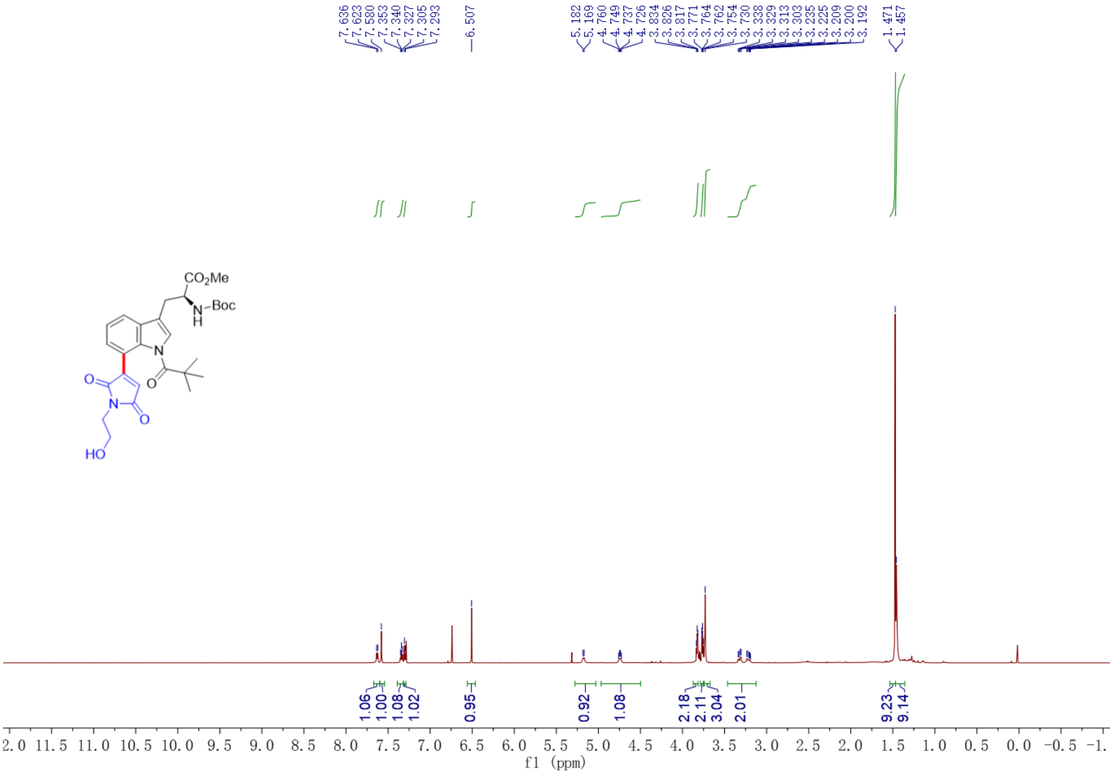

^{1}H NMR (600 MHz, CDCl$_3$) spectrum of **3i**

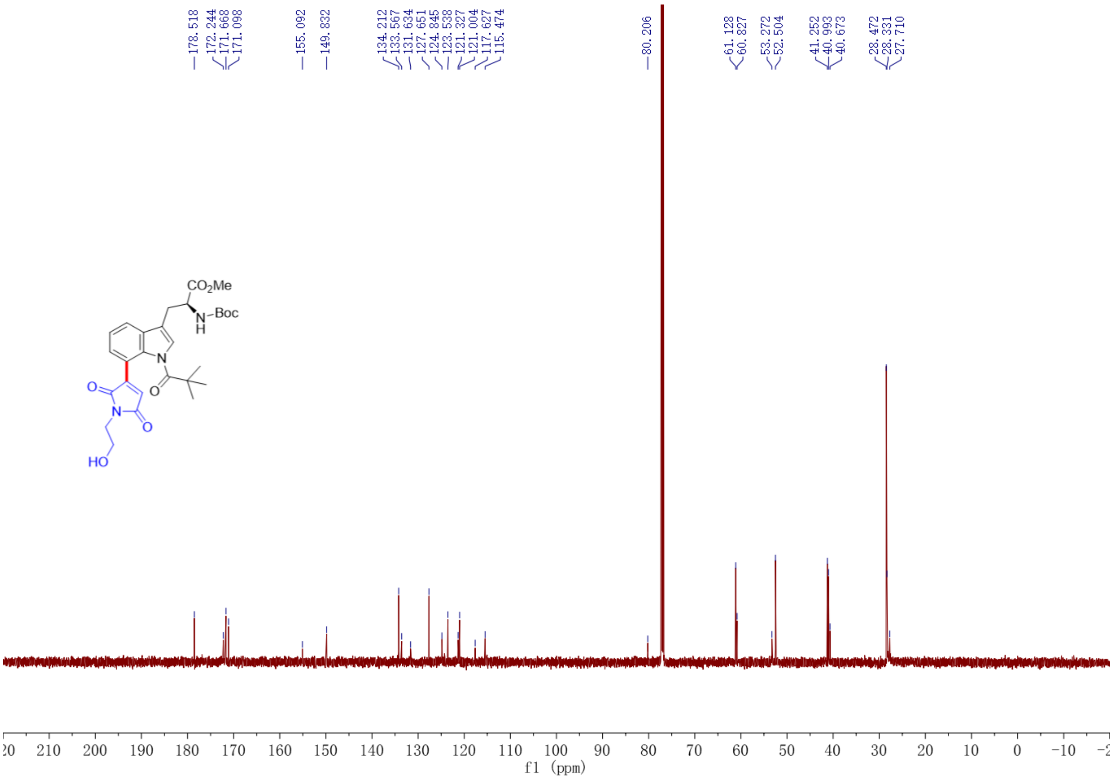

^{1}C NMR (151 MHz, CDCl$_3$) spectrum of **3i**

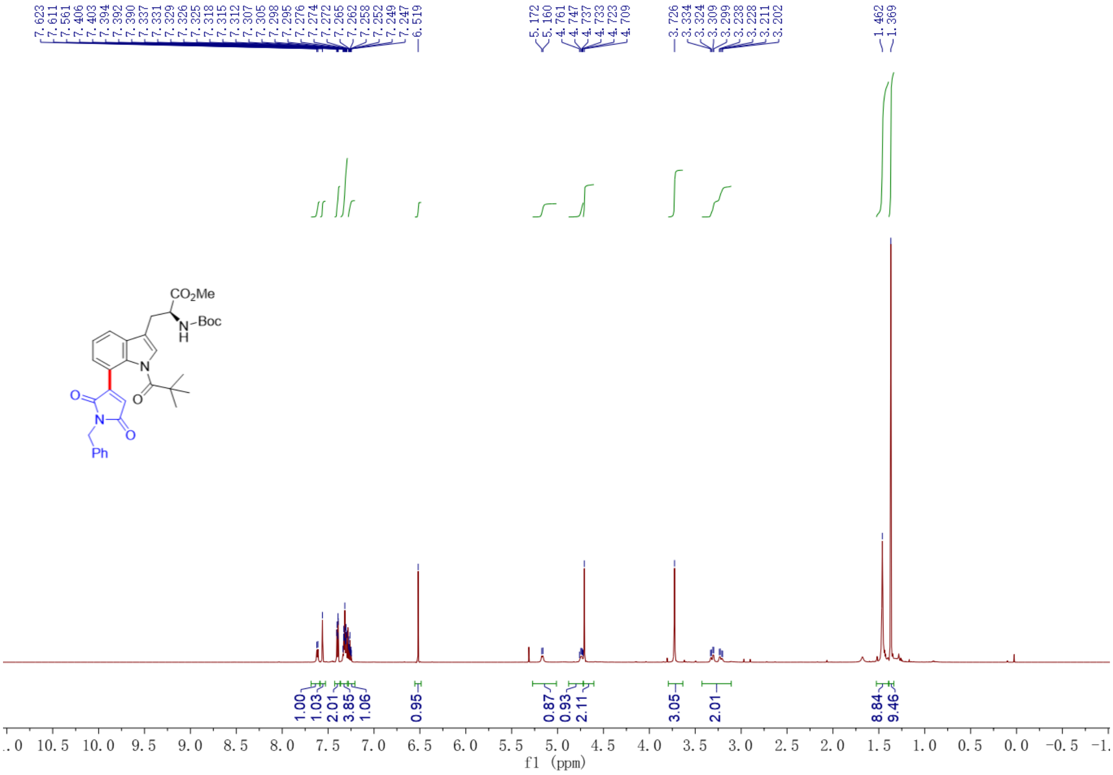

^{1}H NMR (600MHz, CDCl$_3$) spectrum of **3j**

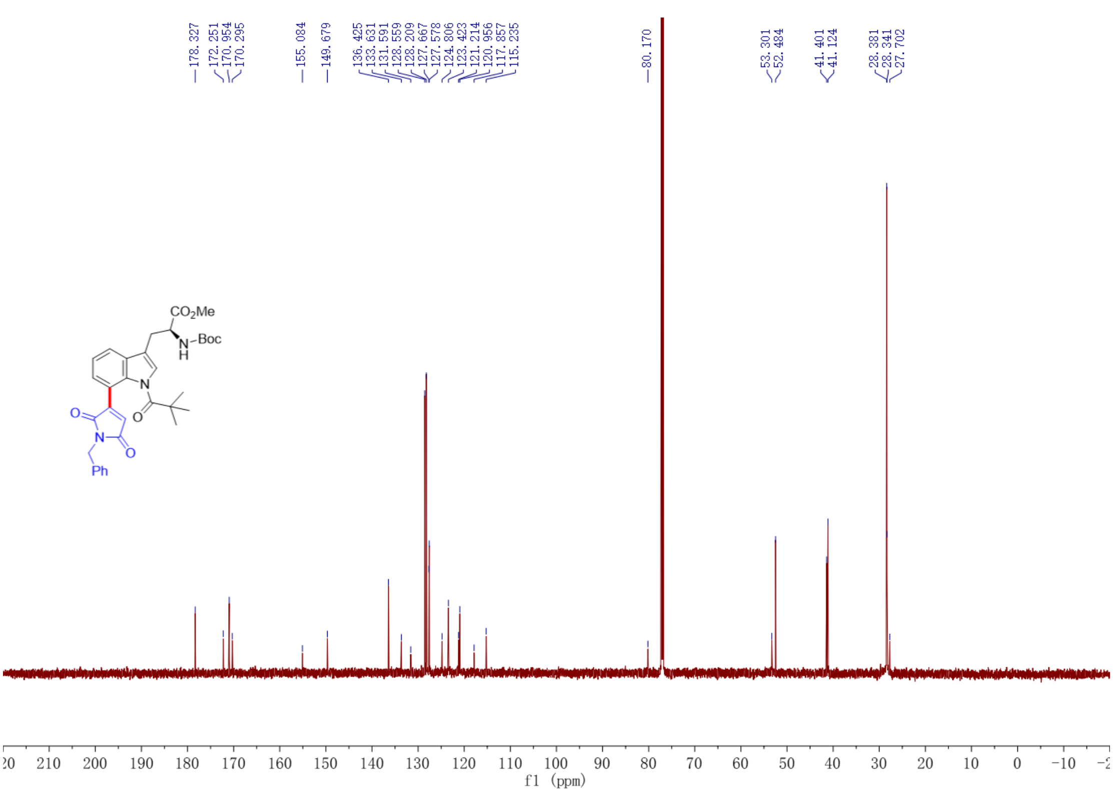

^{1}C NMR (101 MHz, DMSO) spectrum of **3j**

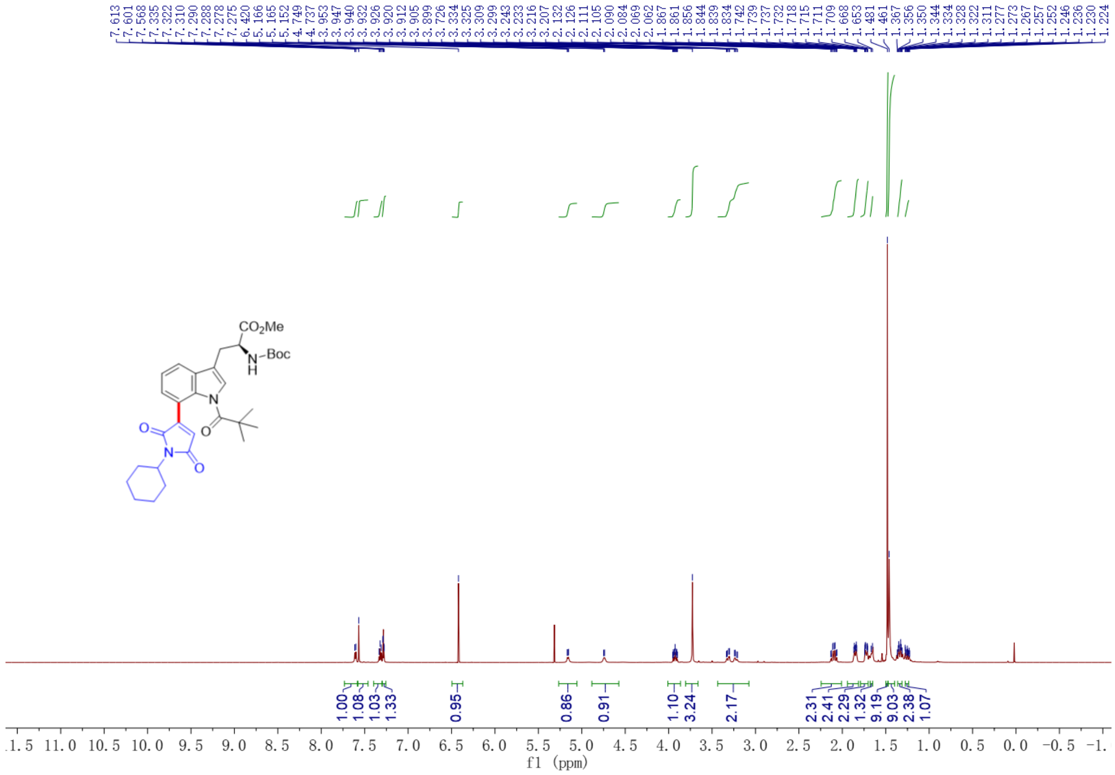

^{1}H NMR (600MHz, $CDCl_3$) spectrum of **3k**

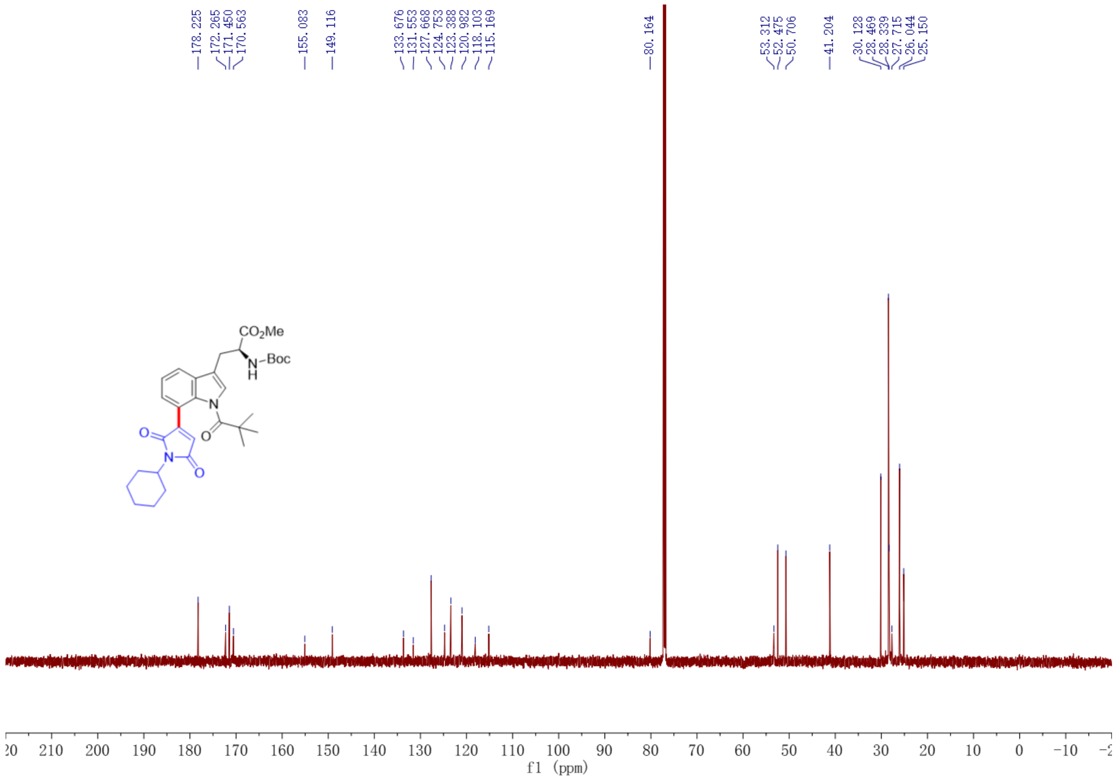

^{1}C NMR (151MHz, $CDCl_3$) spectrum of **3k**

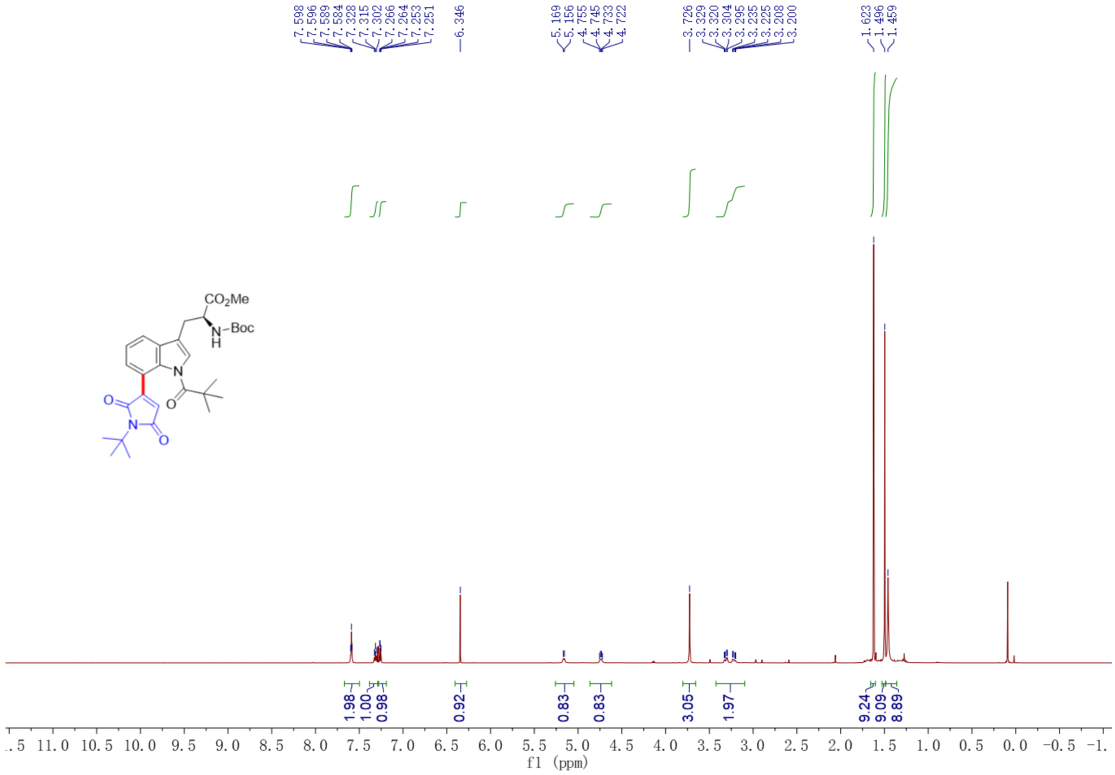

^{1}H NMR (600MHz, $CDCl_3$) spectrum of **3l**

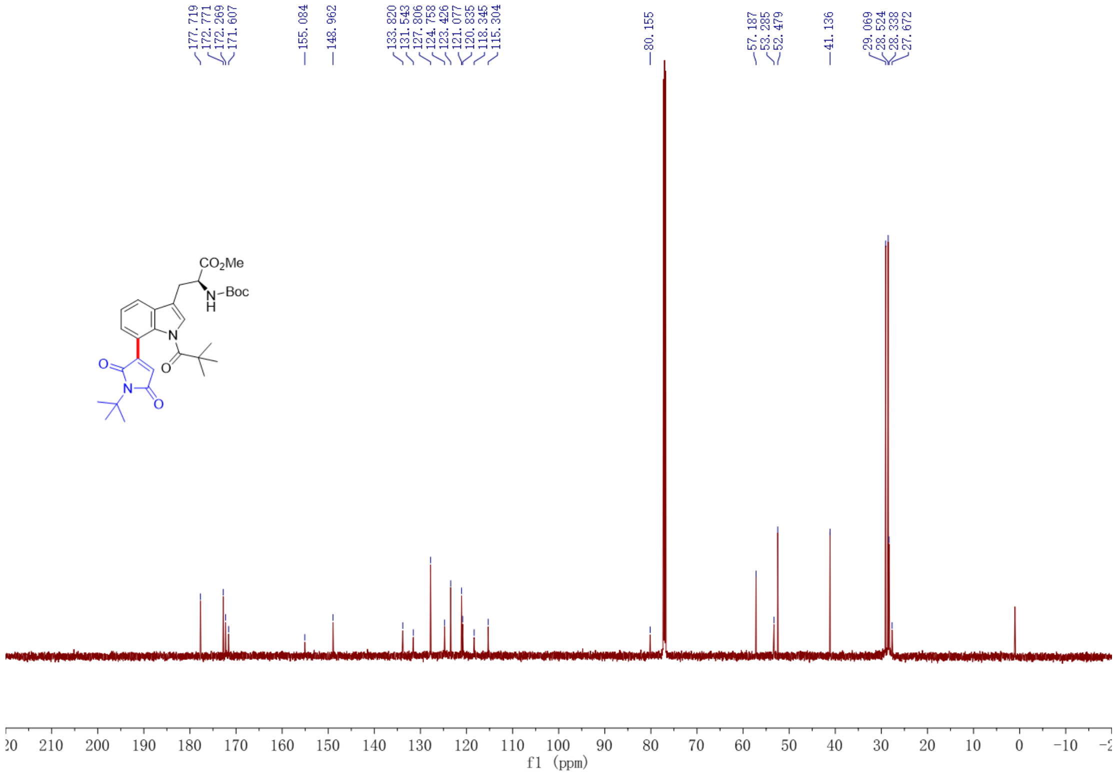

^{1}C NMR (151MHz, $CDCl_3$) spectrum of **3l**

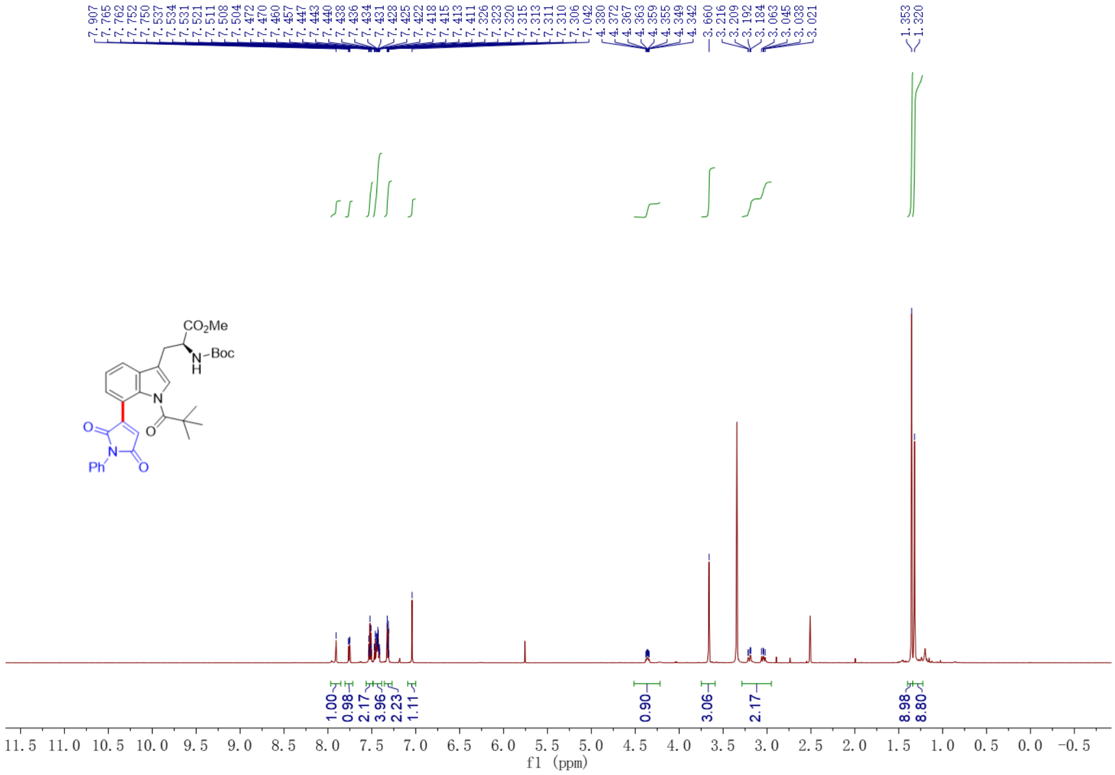

^{1}H NMR (600MHz, $CDCl_3$) spectrum of **3m**

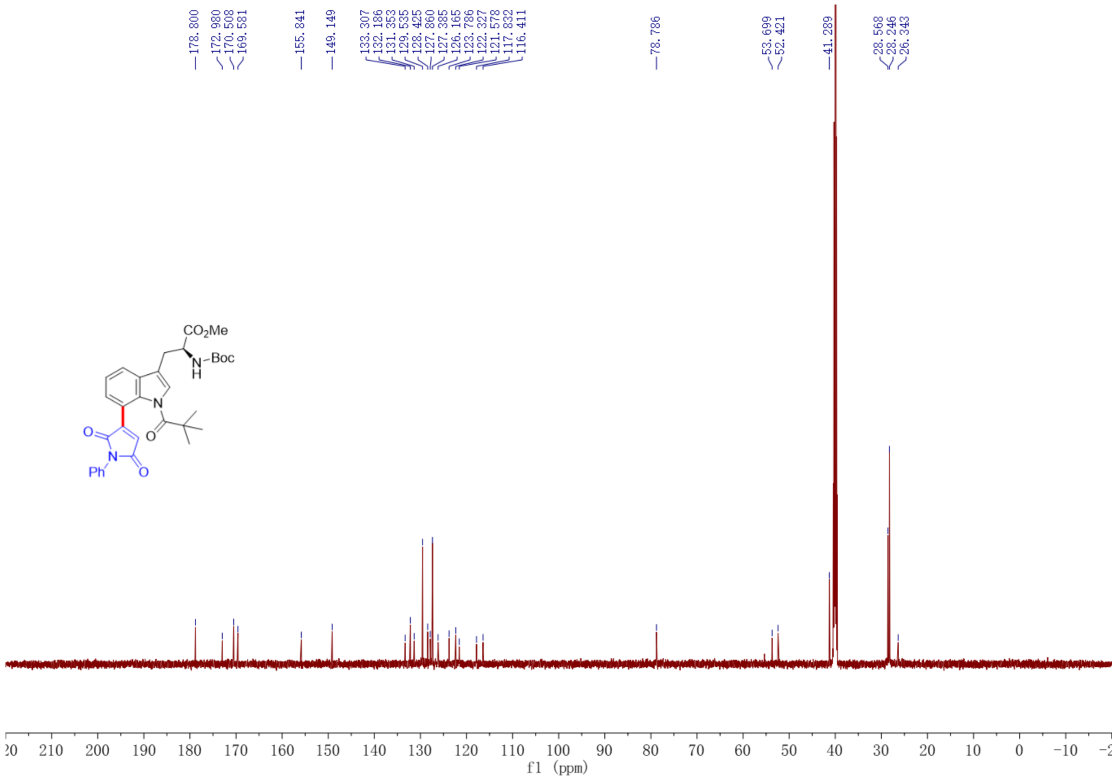

^{1}C NMR (151MHz, $CDCl_3$) spectrum of **3m**

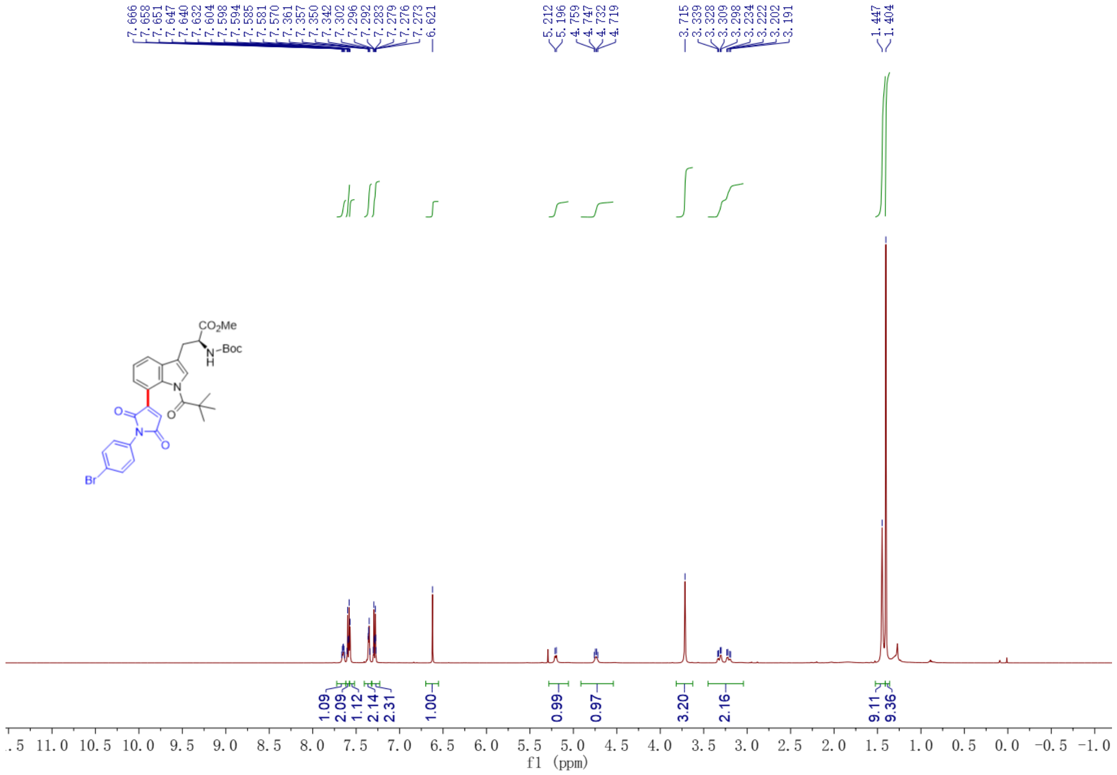

^{1}H NMR (500MHz, $CDCl_3$) spectrum of **3n**

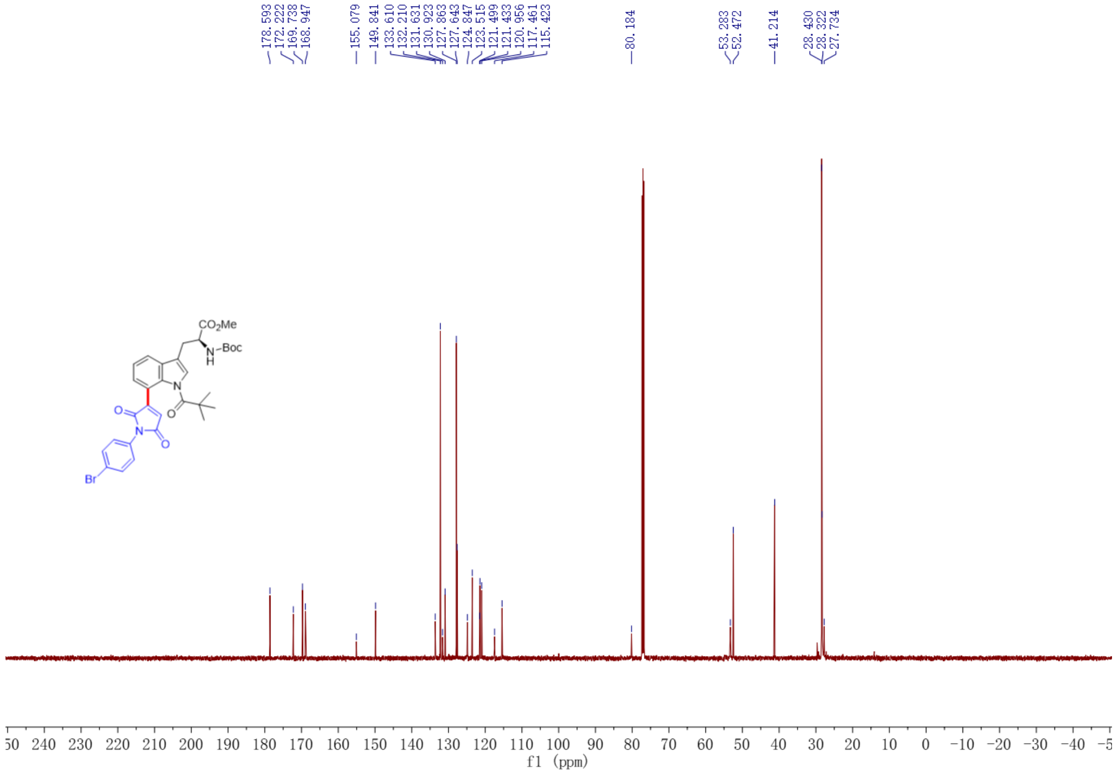

^{1}C NMR (126MHz, $CDCl_3$) spectrum of **3n**

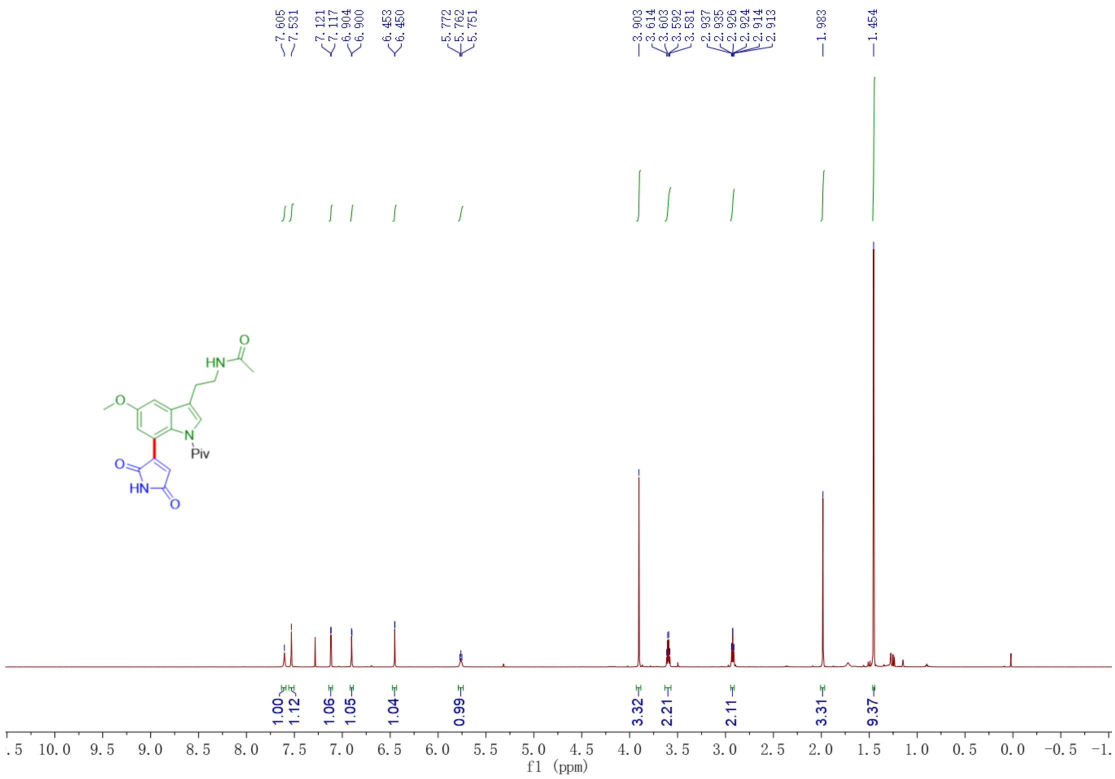

^{1}H NMR (600MHz, $CDCl_3$) spectrum of **3o**

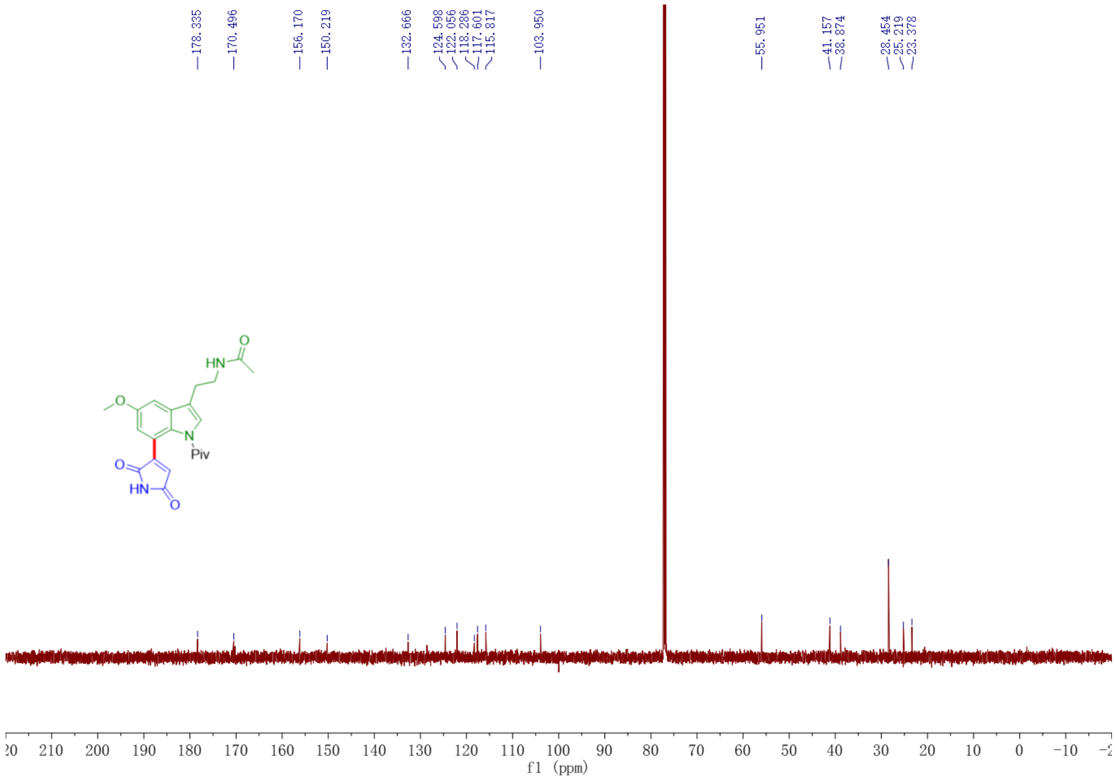

[1]C NMR (151MHz, $CDCl_3$) spectrum of **3o**

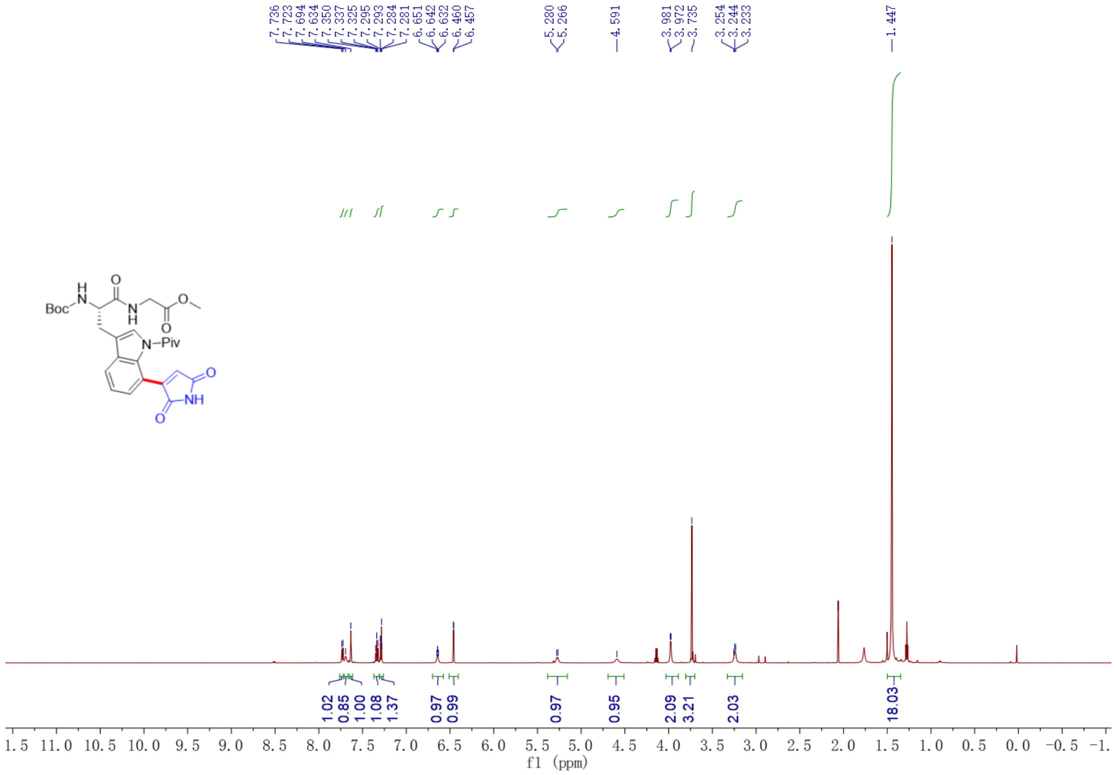

^{1}H NMR (600 MHz, $CDCl_3$) spectrum of **5a**

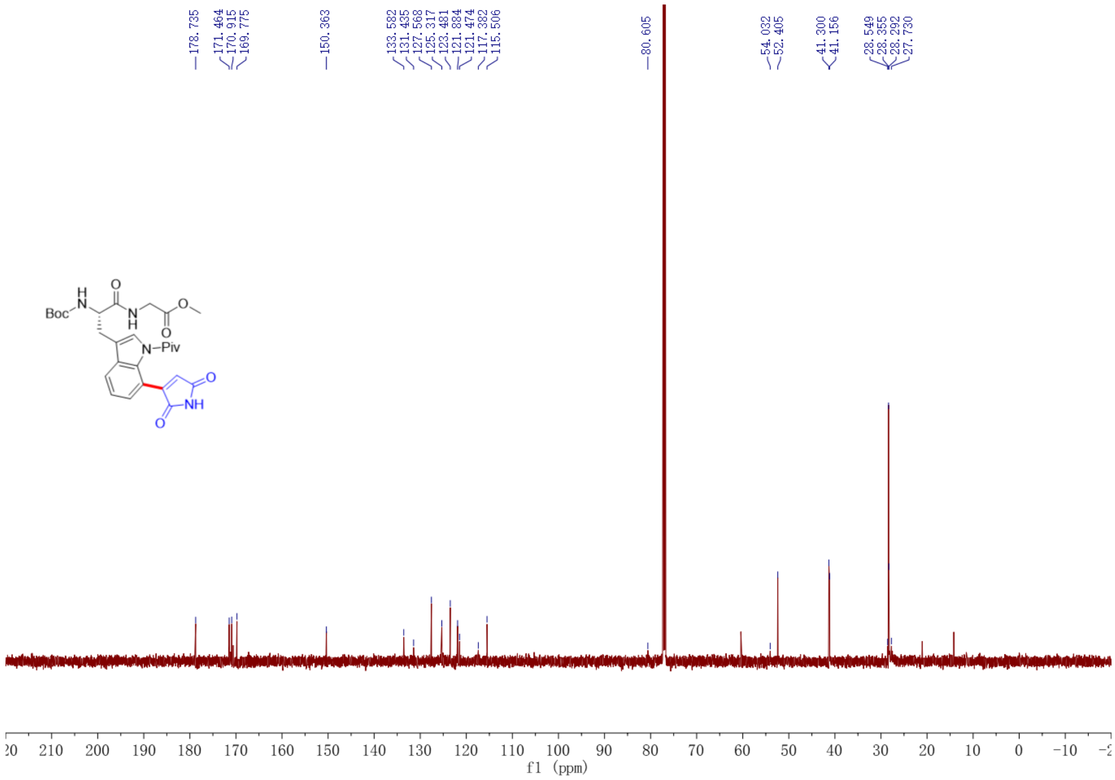

[1]C NMR (151 MHz, $CDCl_3$) spectrum of **5a**

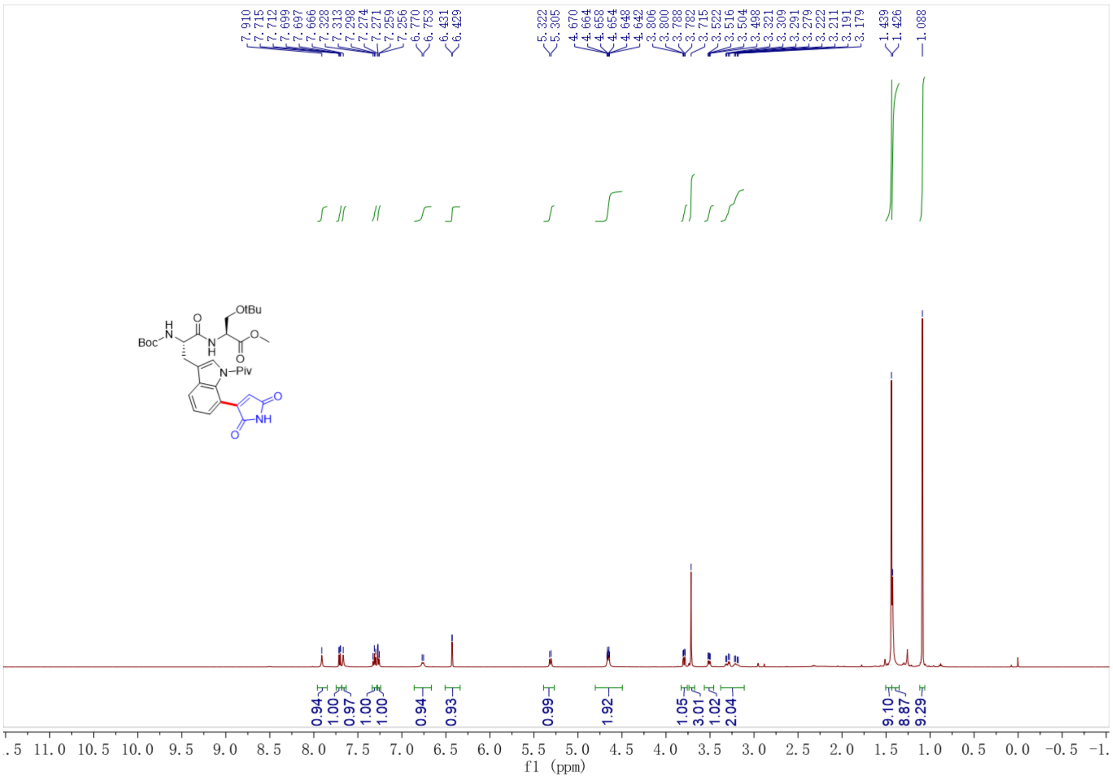

^{1}H NMR (500 MHz, $CDCl_3$) spectrum of **5b₁**

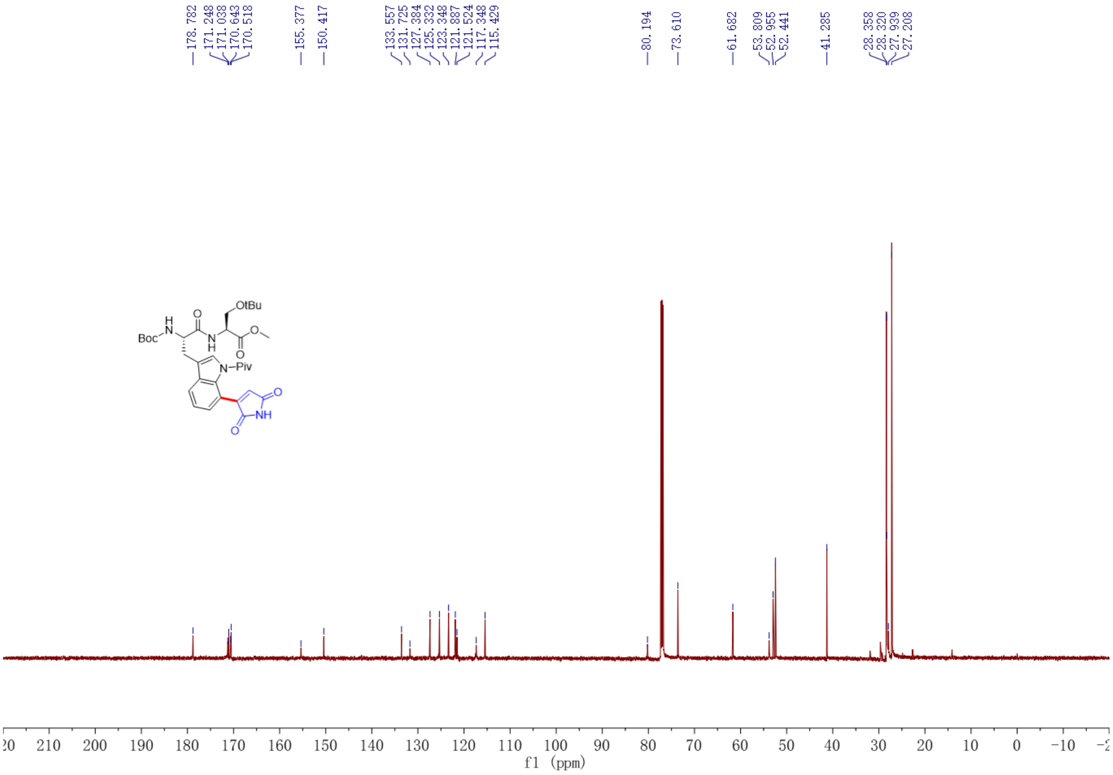

^{1}C NMR (126 MHz, $CDCl_3$) spectrum of **5b₁**

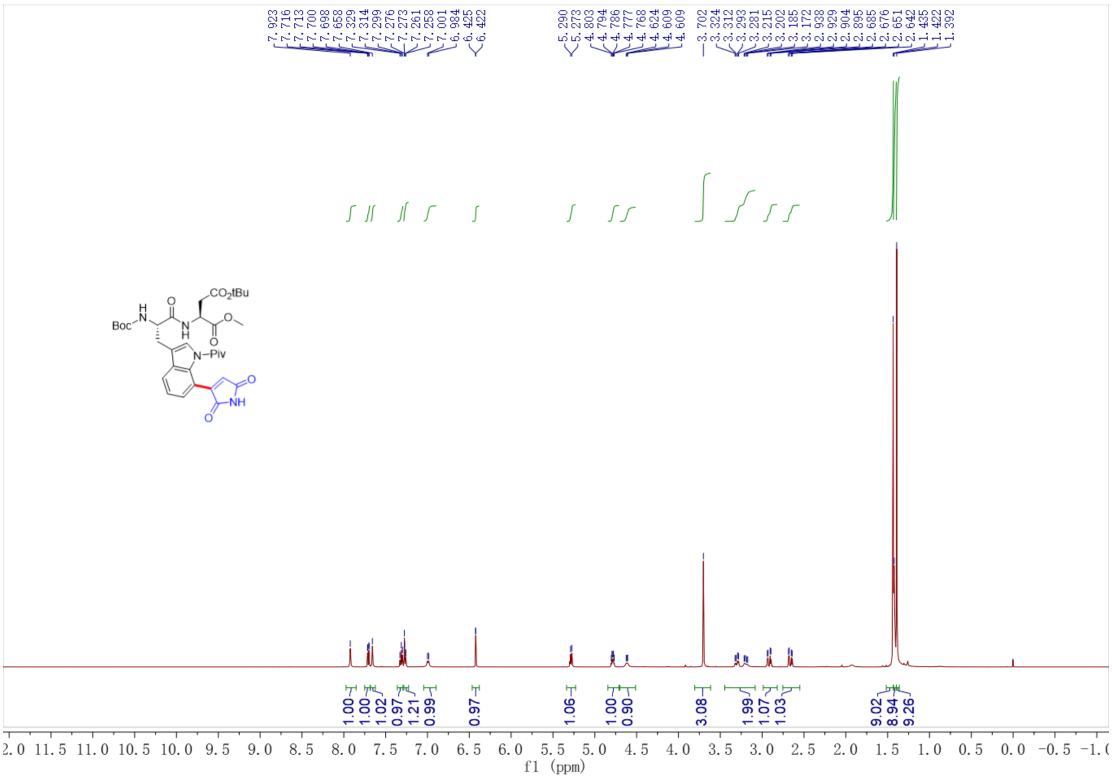

^{1}H NMR (500 MHz, CDCl$_3$) spectrum of **5b$_2$**

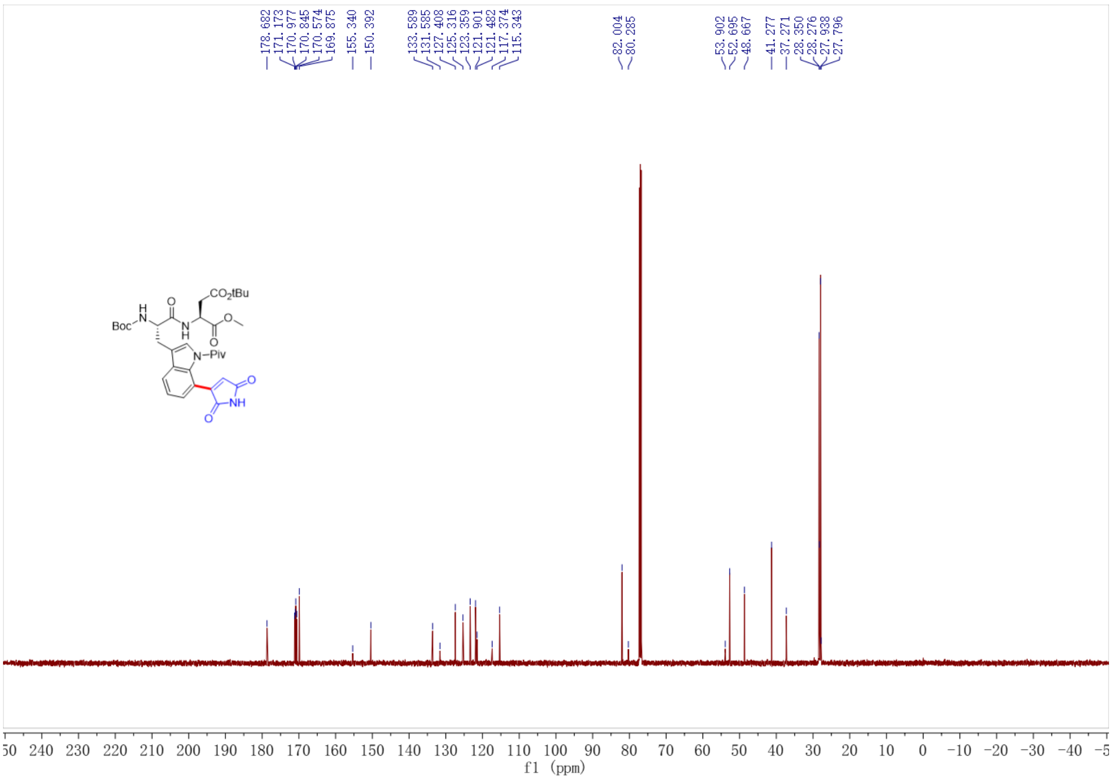

^{1}C NMR (126 MHz, CDCl$_3$) spectrum of **5b$_2$**

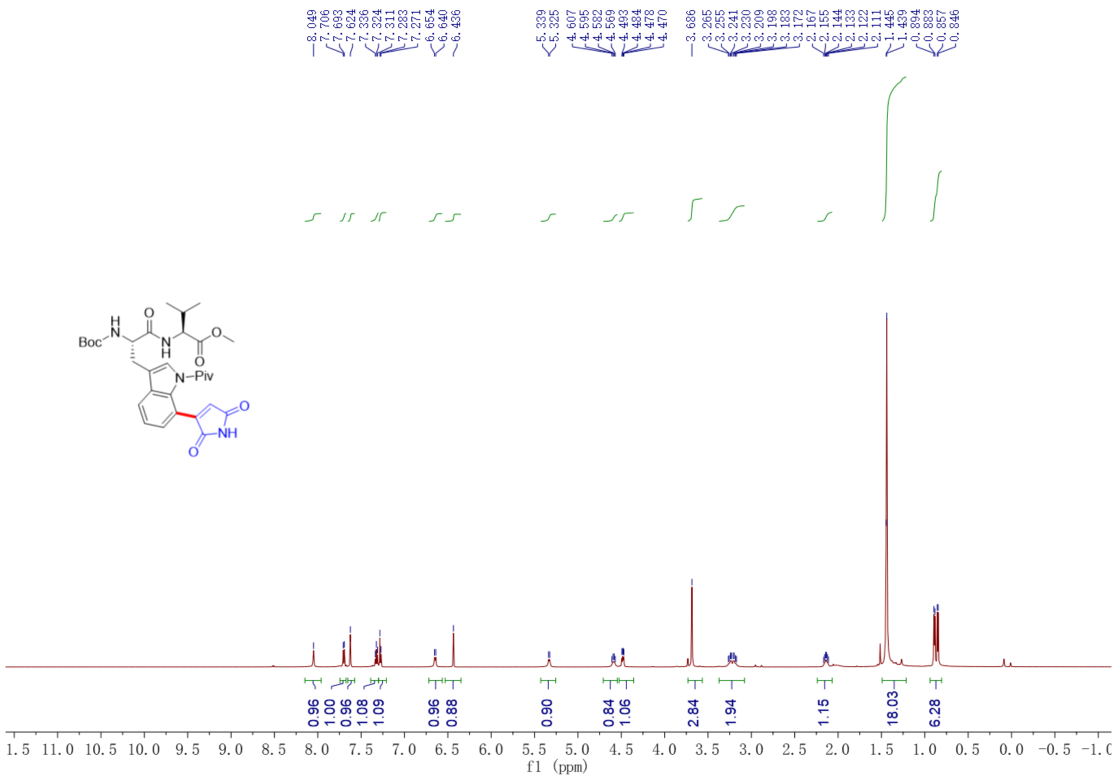

^{1}H NMR (600 MHz, $CDCl_3$) spectrum of **5c₁**

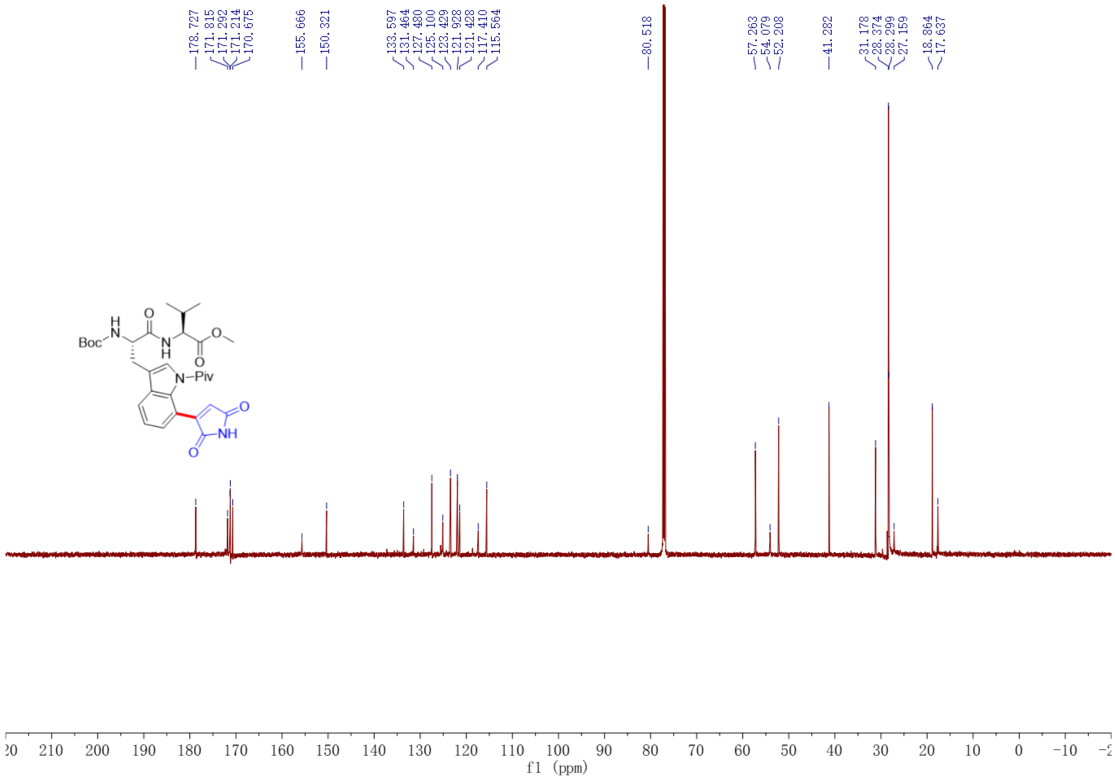

^{1}C NMR (151 MHz, $CDCl_3$) spectrum of **5c₁**

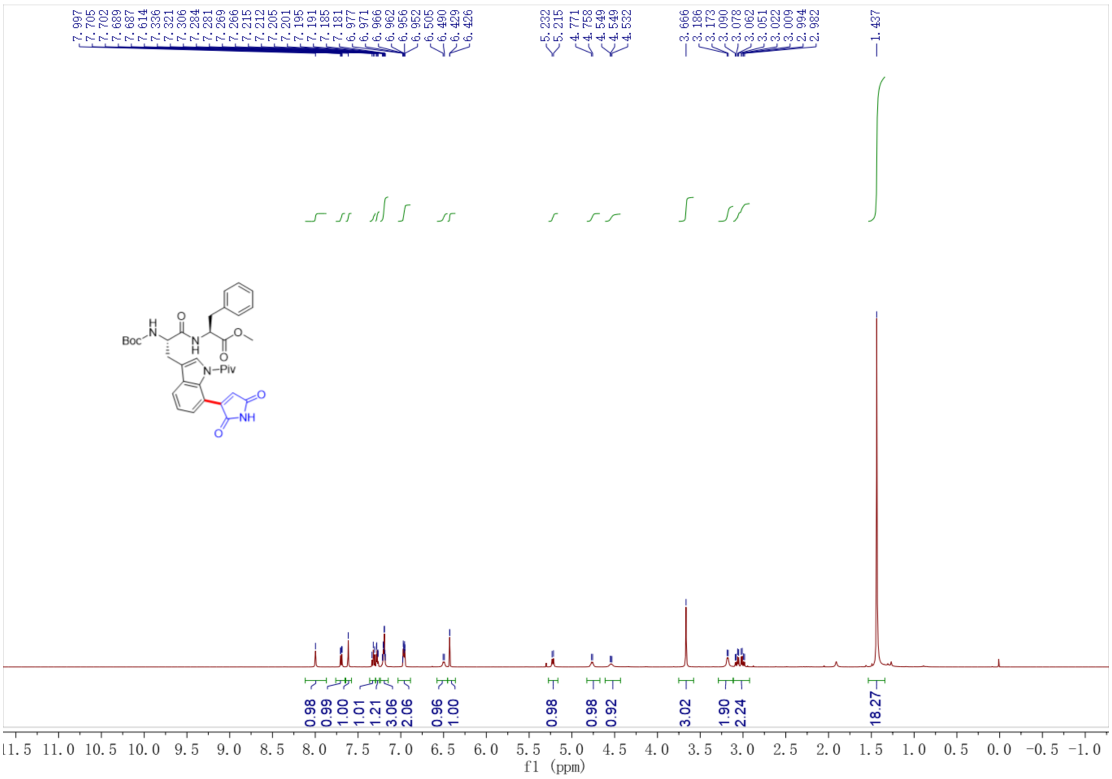

^{1}H NMR (500 MHz, CDCl$_3$) spectrum of **5c$_2$**

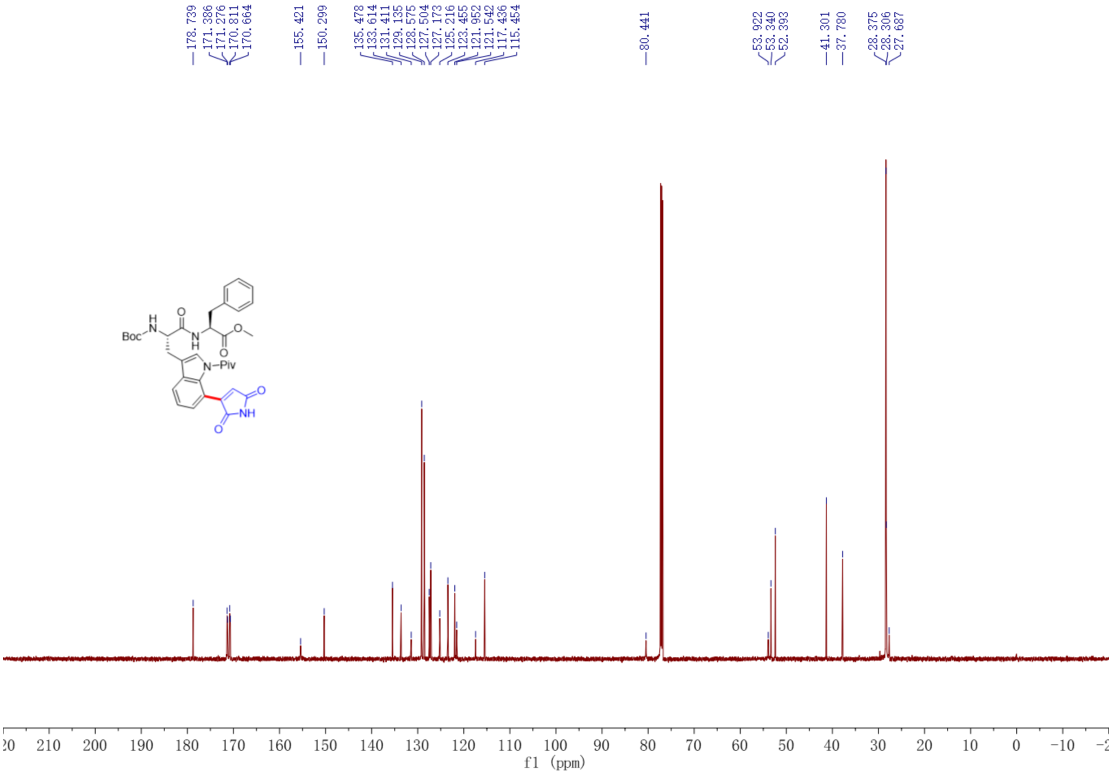

^{1}C NMR (126 MHz, CDCl$_3$) spectrum of **5c$_2$**

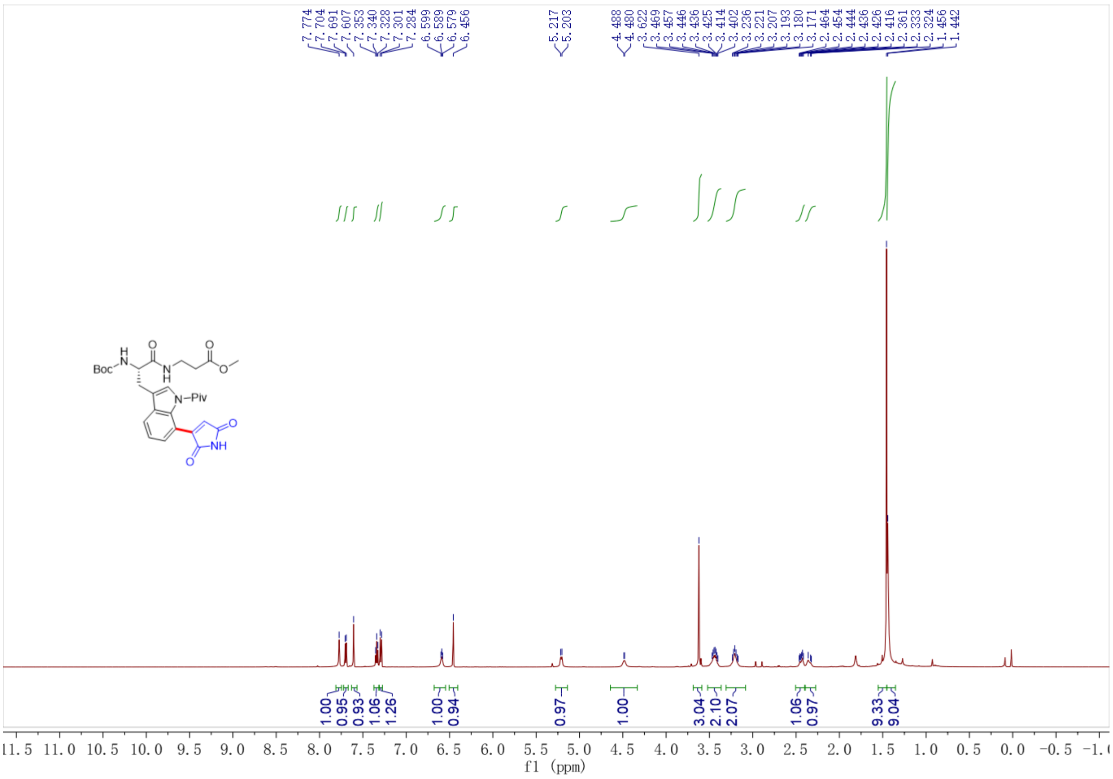

^{1}H NMR (600 MHz, $CDCl_3$) spectrum of **5d**

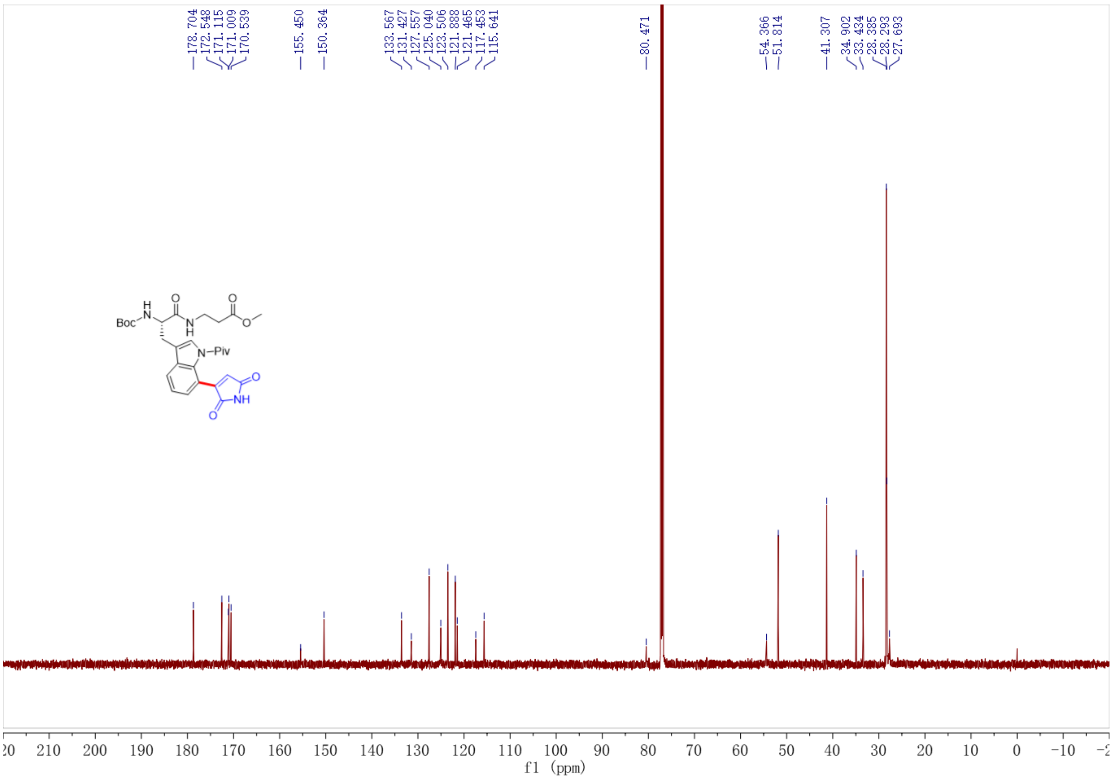

^{1}C NMR (151 MHz, $CDCl_3$) spectrum of **5d**

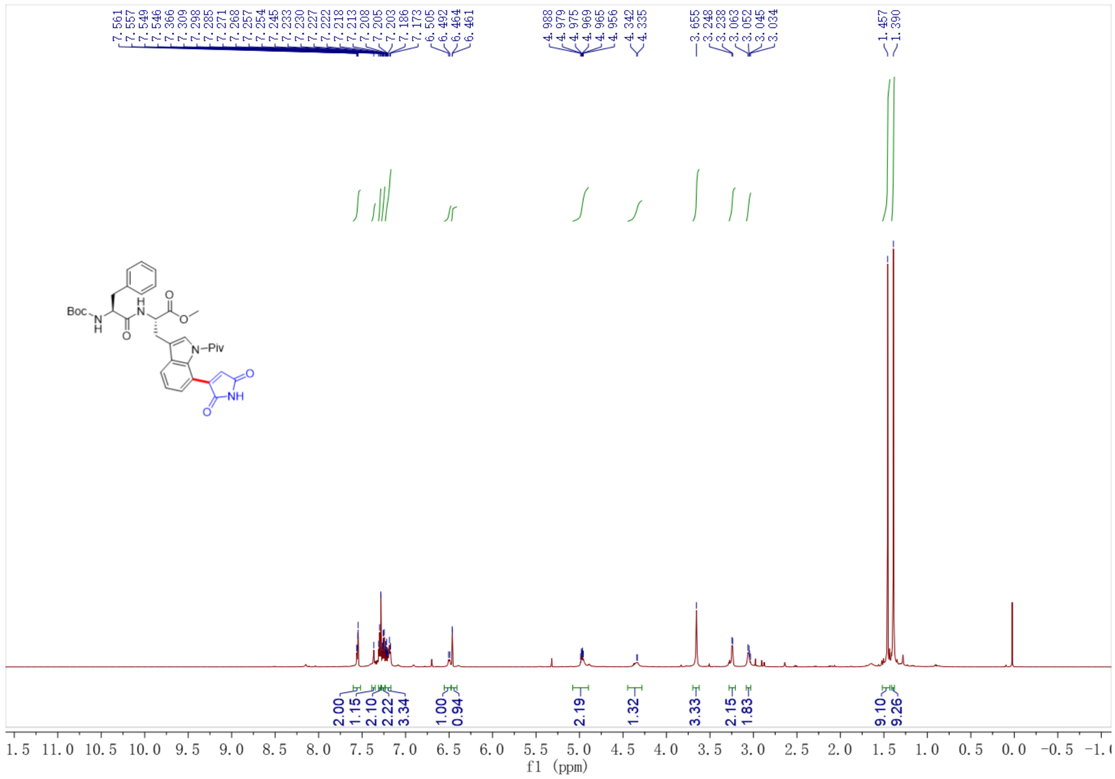

^{1}H NMR (600 MHz, $CDCl_3$) spectrum of **5e$_1$**

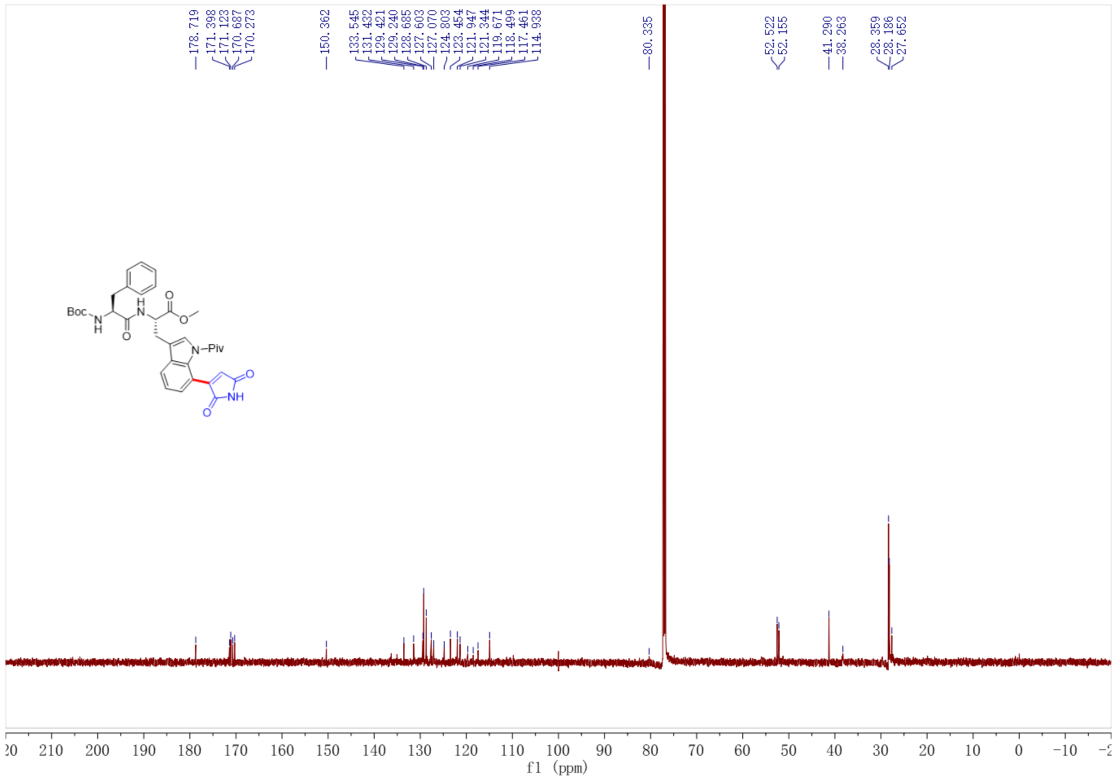

^{1}C NMR (151 MHz, $CDCl_3$) spectrum of **5e$_1$**

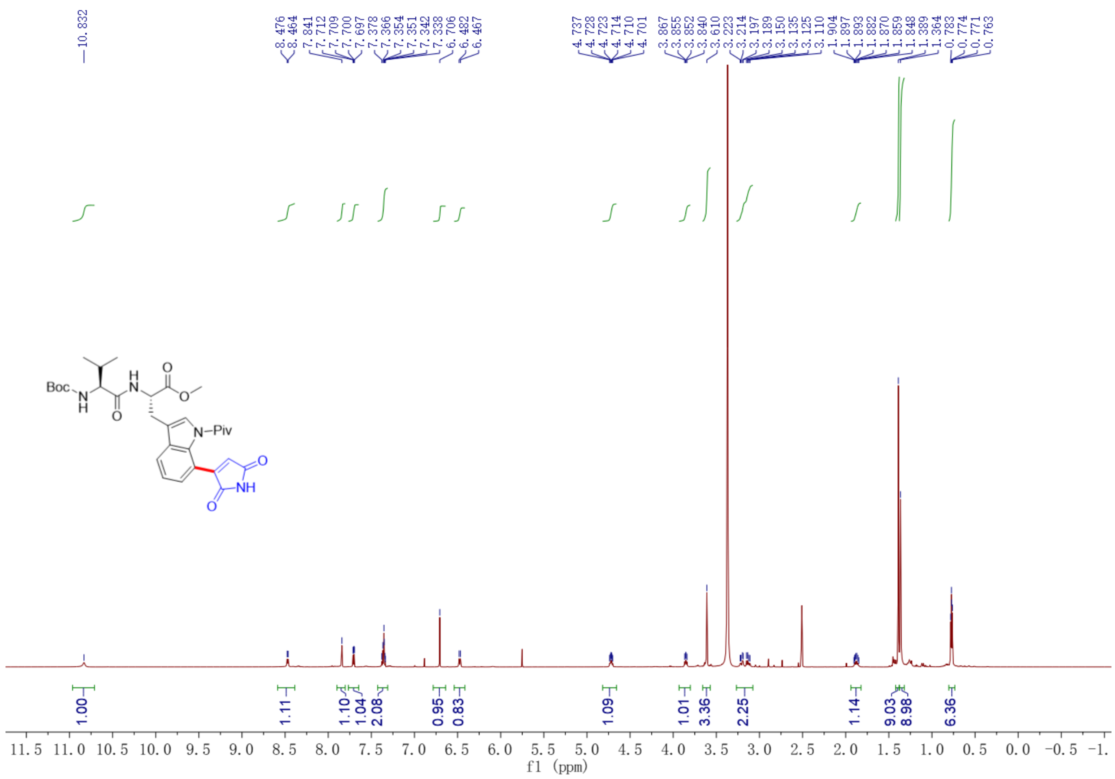

^{1}H NMR (600 MHz, DMSO) spectrum of **5e$_2$**

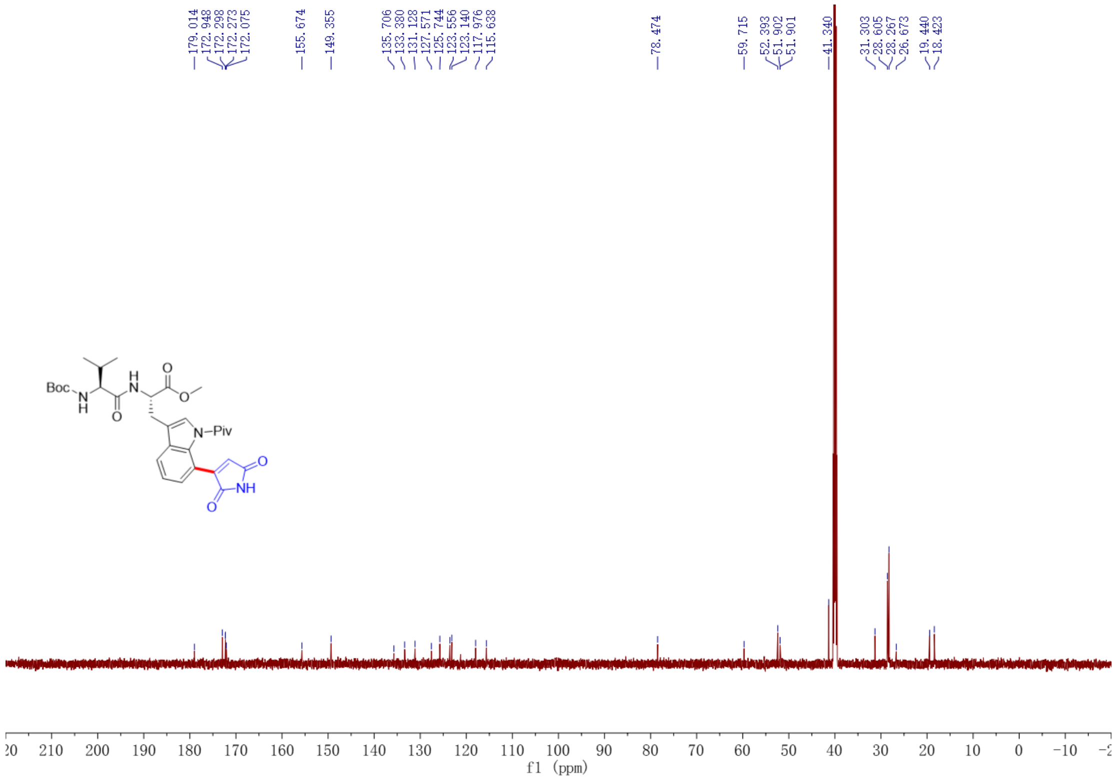

^{1}C NMR (151 MHz, DMSO) spectrum of **5e$_2$**

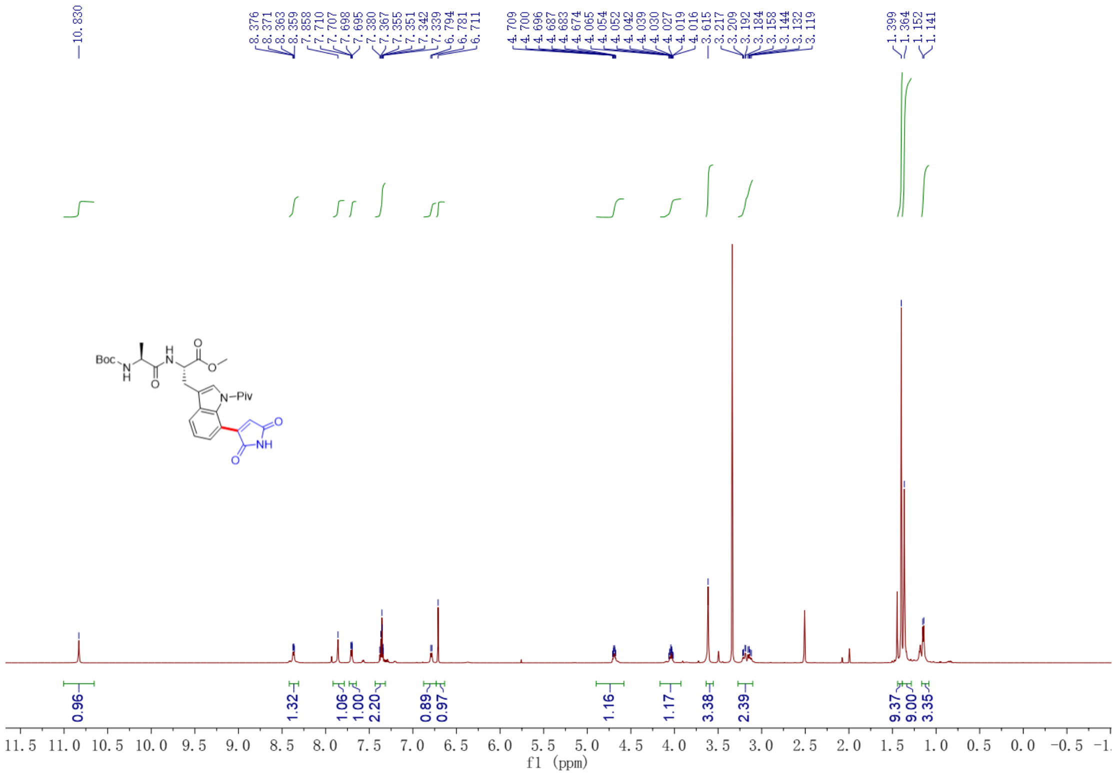

^{1}H NMR (600 MHz, DMSO) spectrum of **5e3**

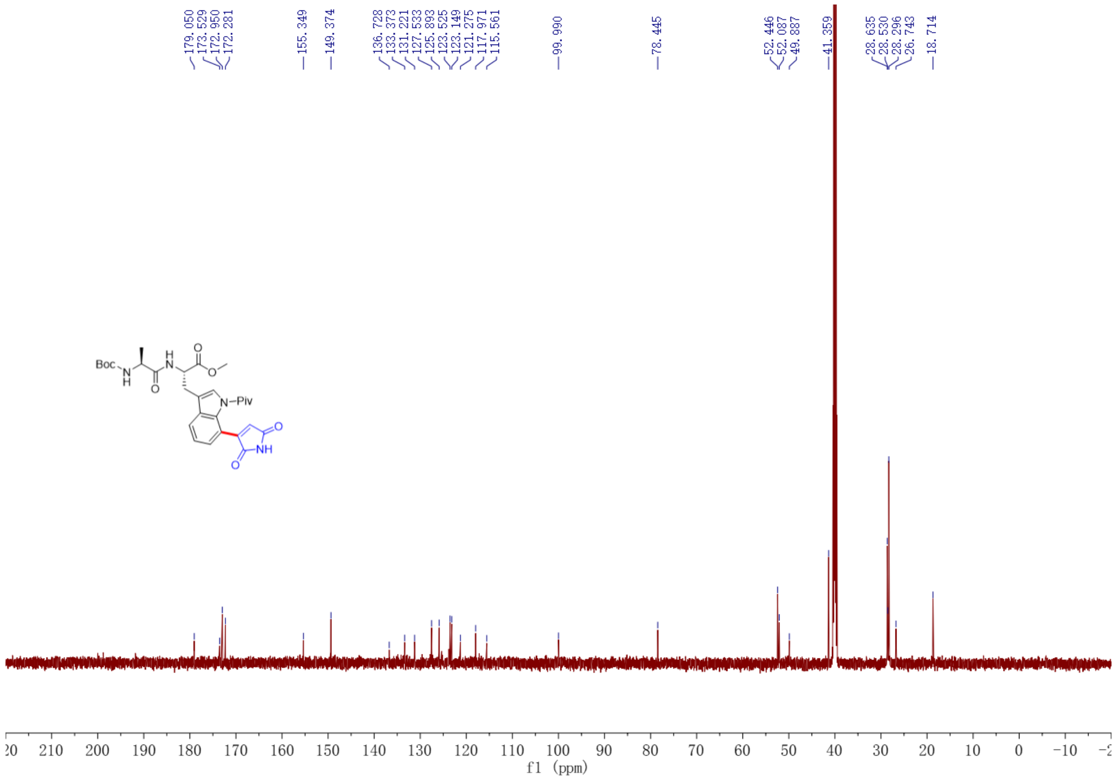

^{1}C NMR (151 MHz, DMSO) spectrum of **5e3**

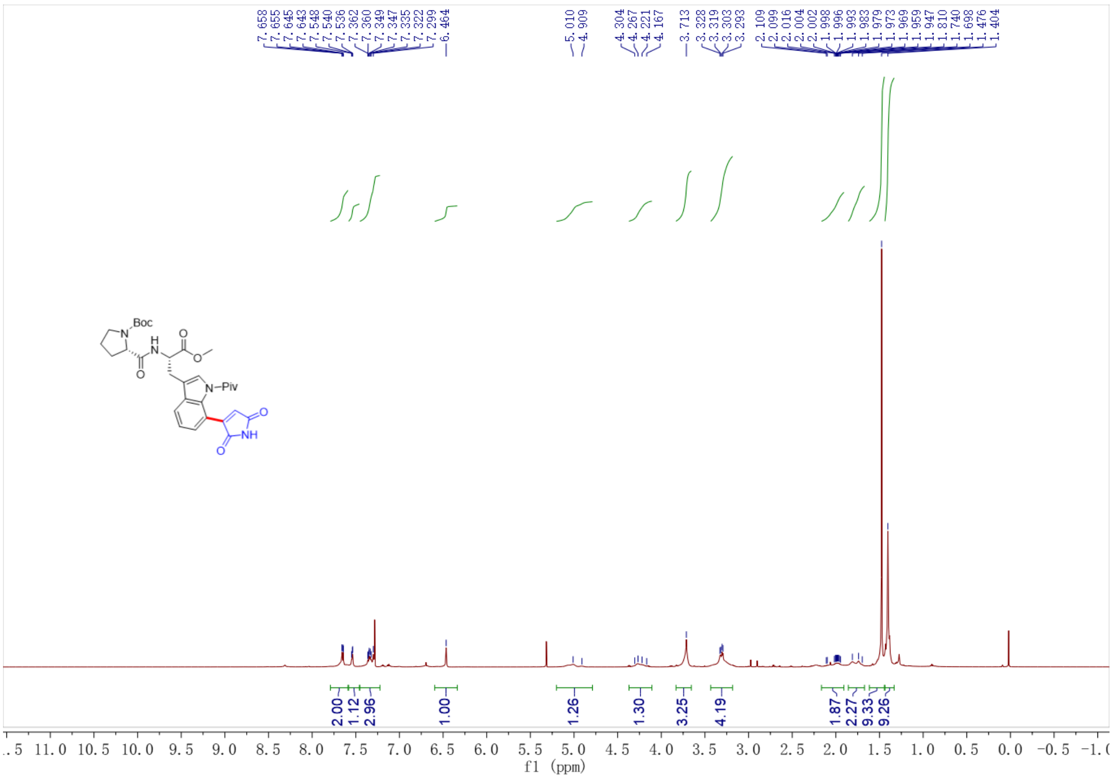

^{1}H NMR (600 MHz, $CDCl_3$) spectrum of **5f**

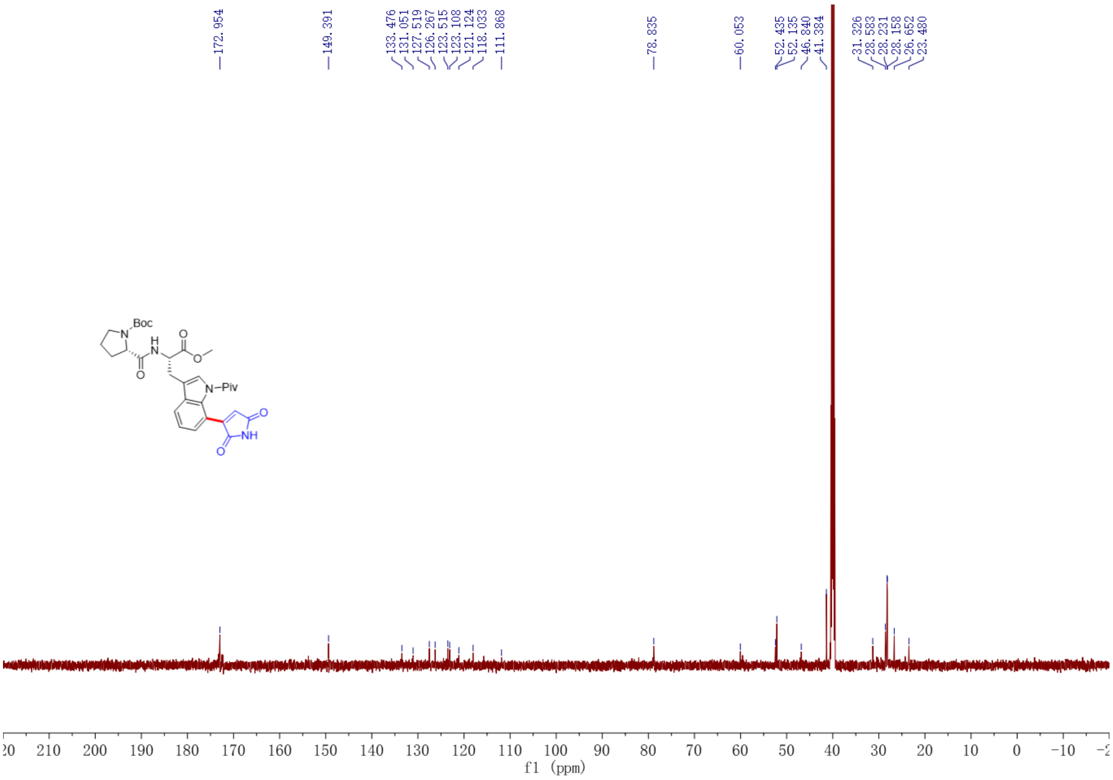

[1]C NMR (151 MHz, DMSO) spectrum of **5f**

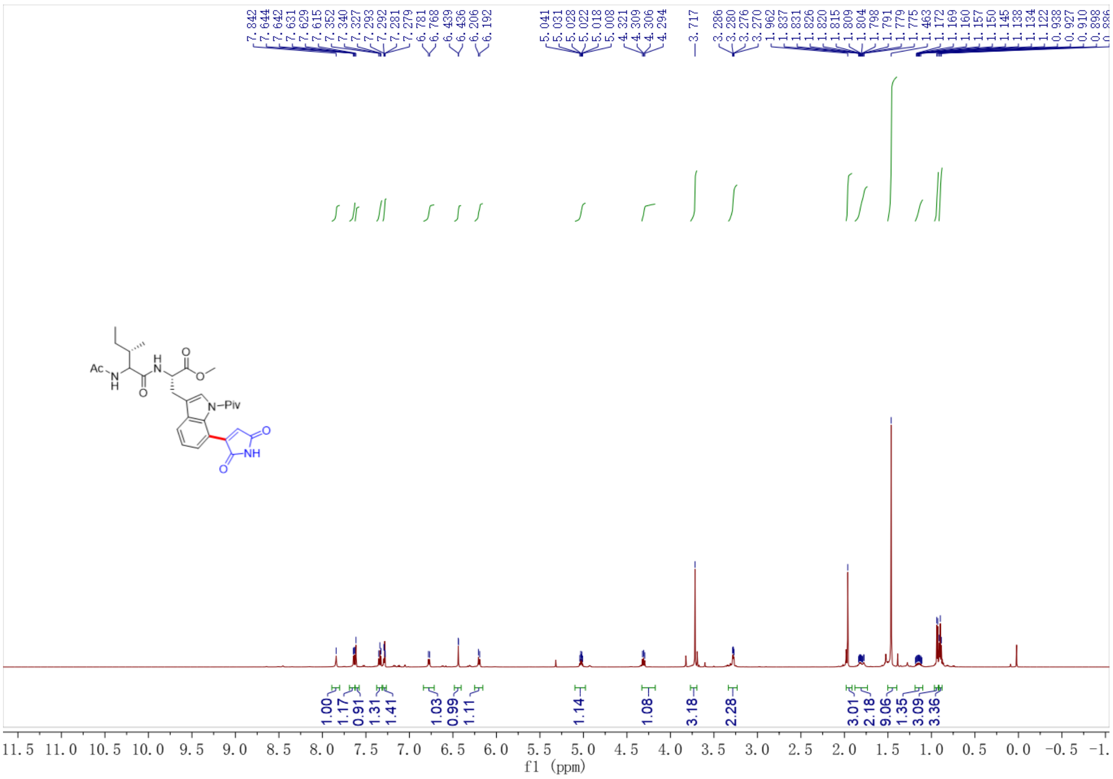

^{1}H NMR (600 MHz, $CDCl_3$) spectrum of **5g**

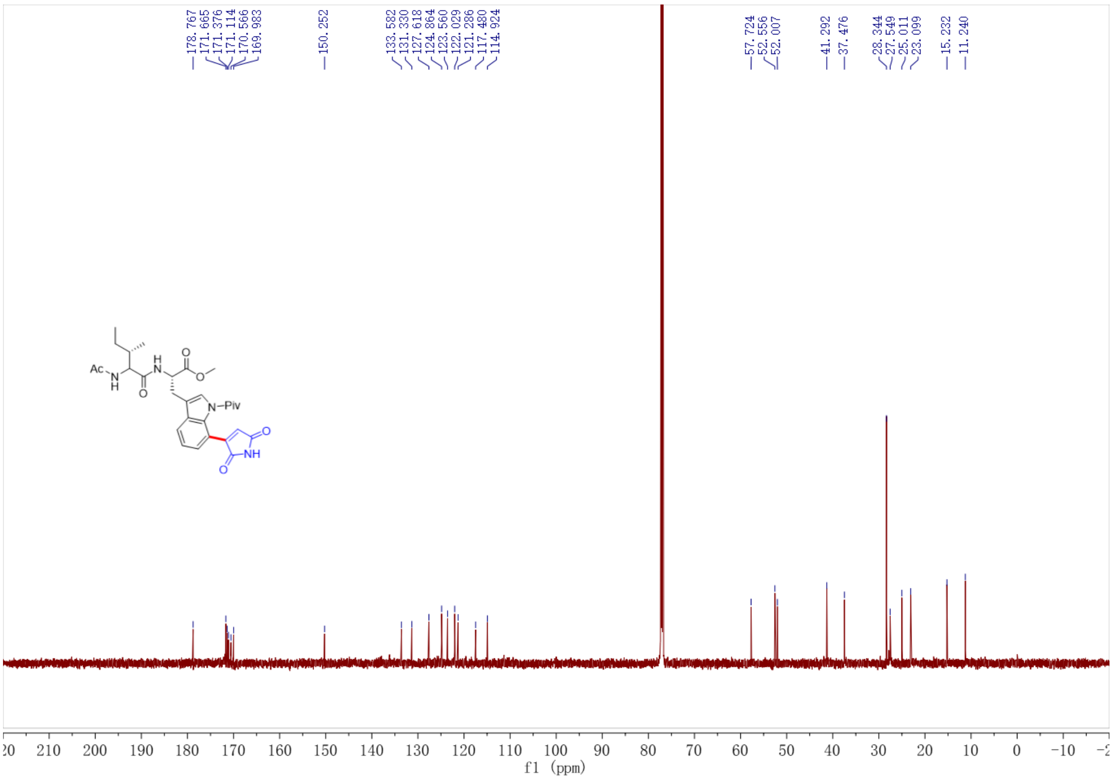

^{1}C NMR (151 MHz, $CDCl_3$) spectrum of **5g**

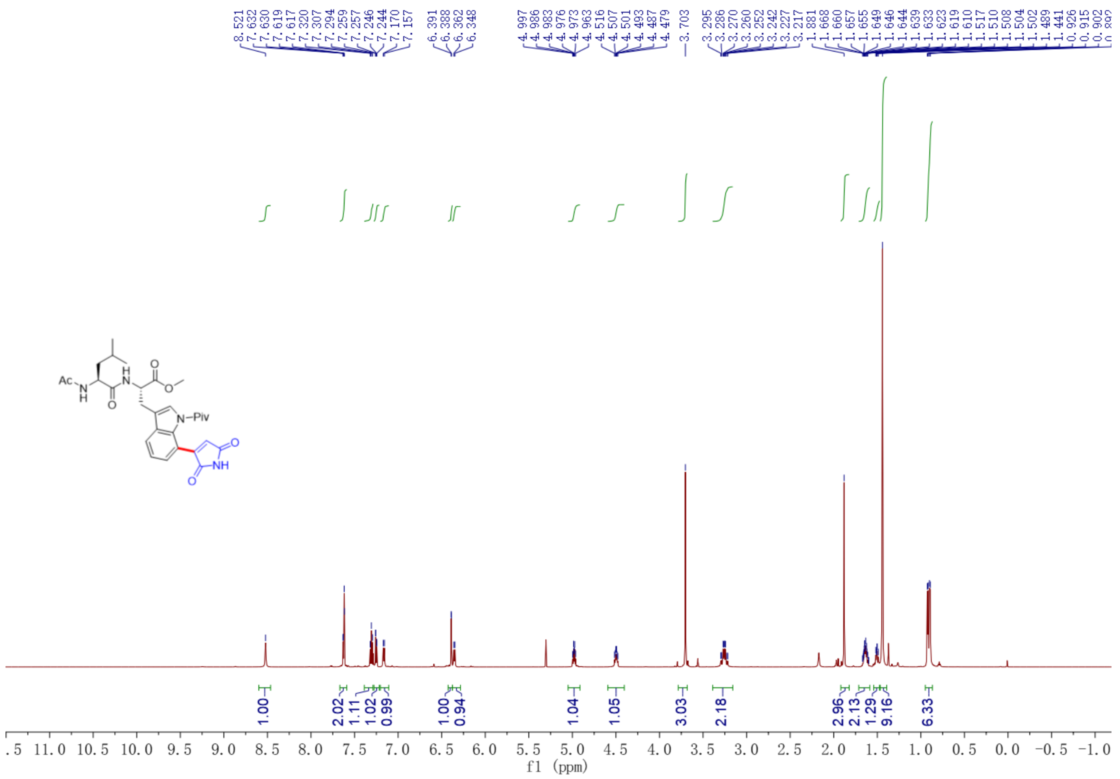

^{1}H NMR (600 MHz, $CDCl_3$) spectrum of **5h**

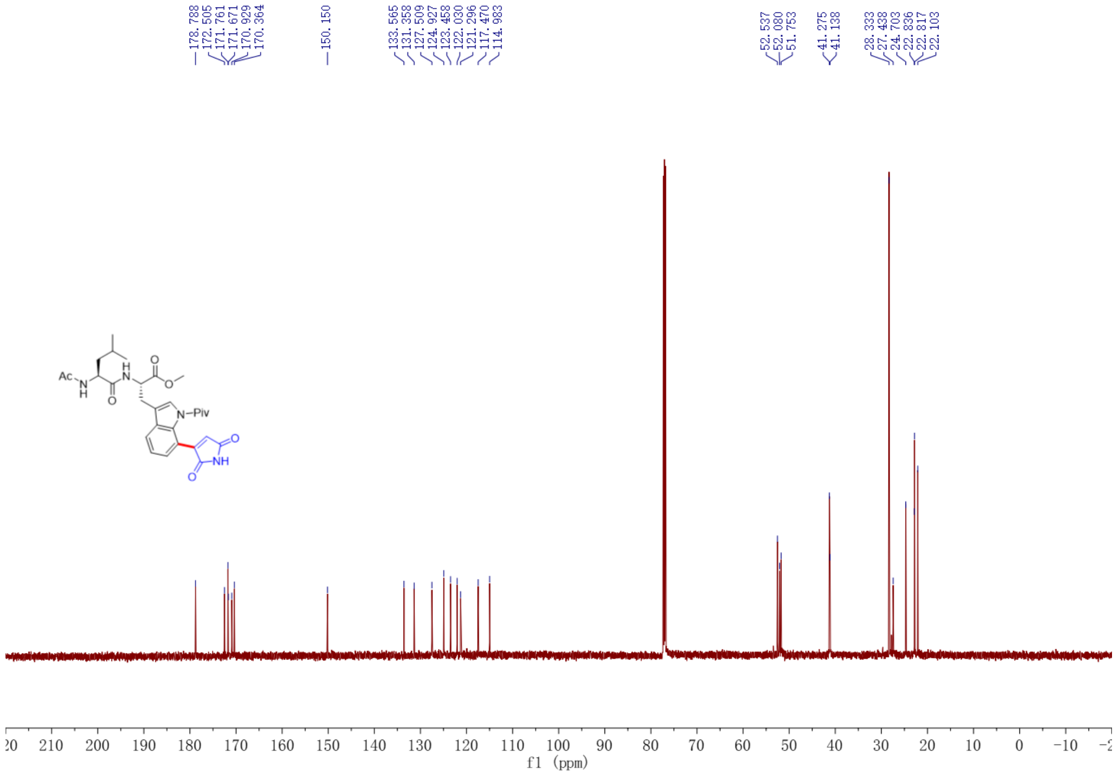

[1]C NMR (151 MHz, $CDCl_3$) spectrum of **5h**

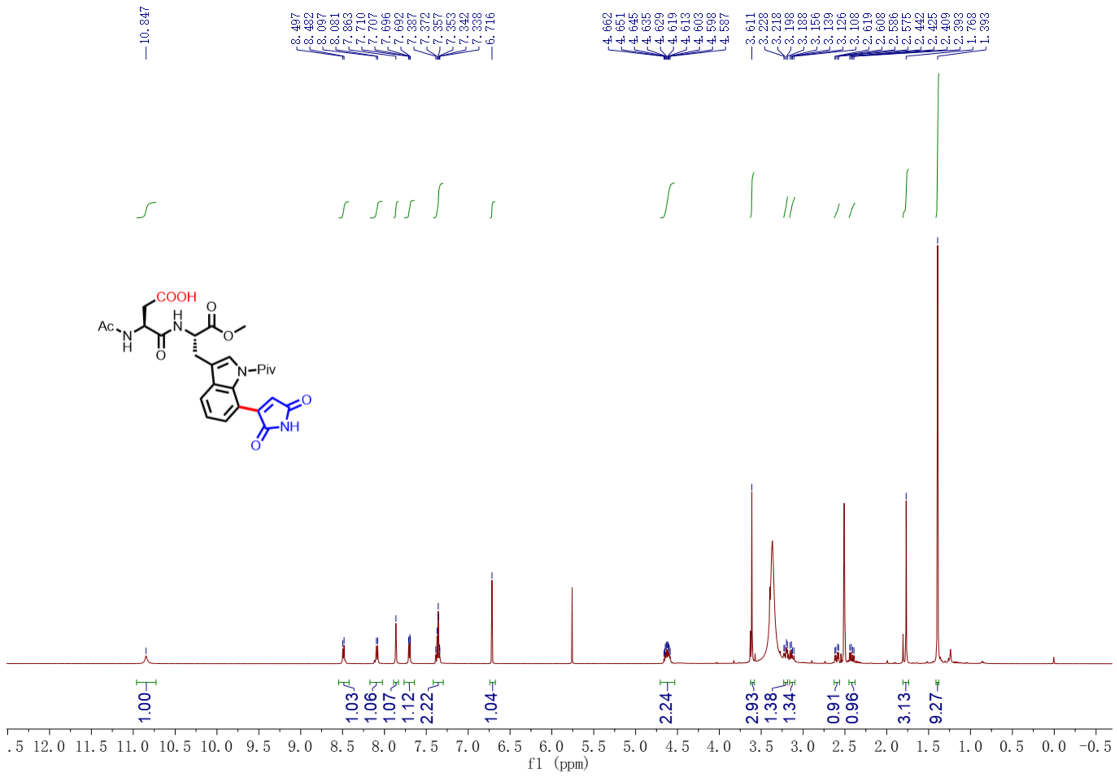

^{1}H NMR (500 MHz, DMSO) spectrum of **5i**

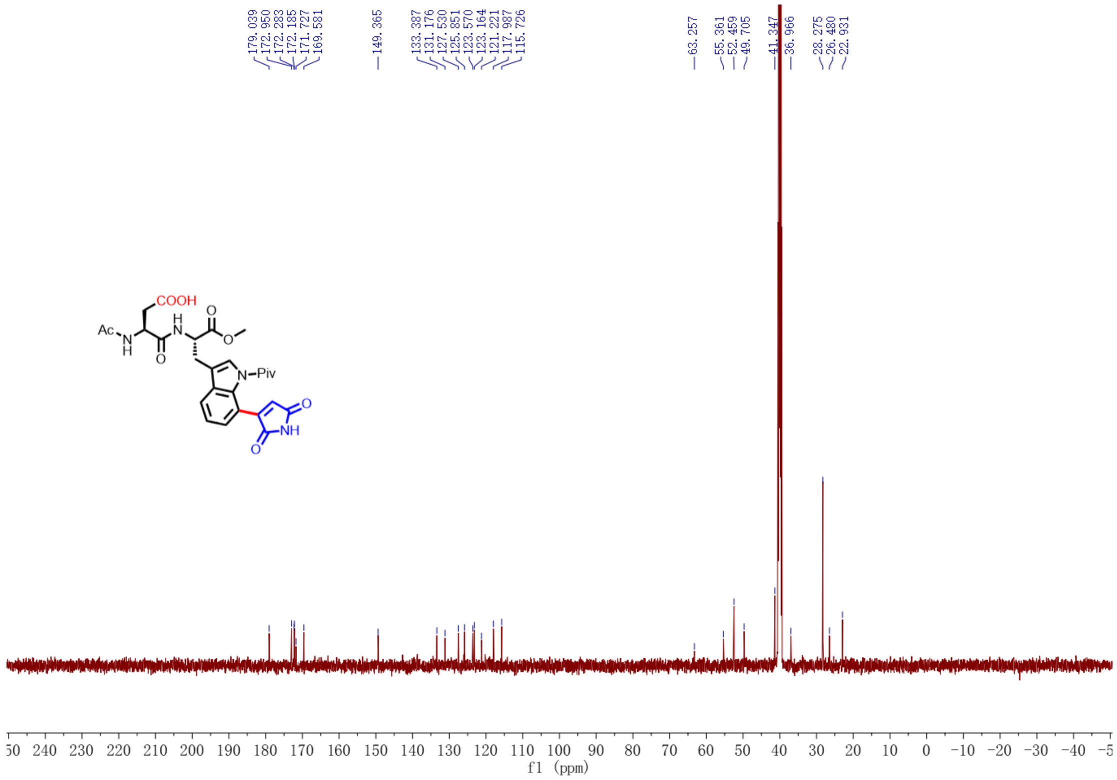

[1]C NMR (126 MHz, DMSO) spectrum of **5i**

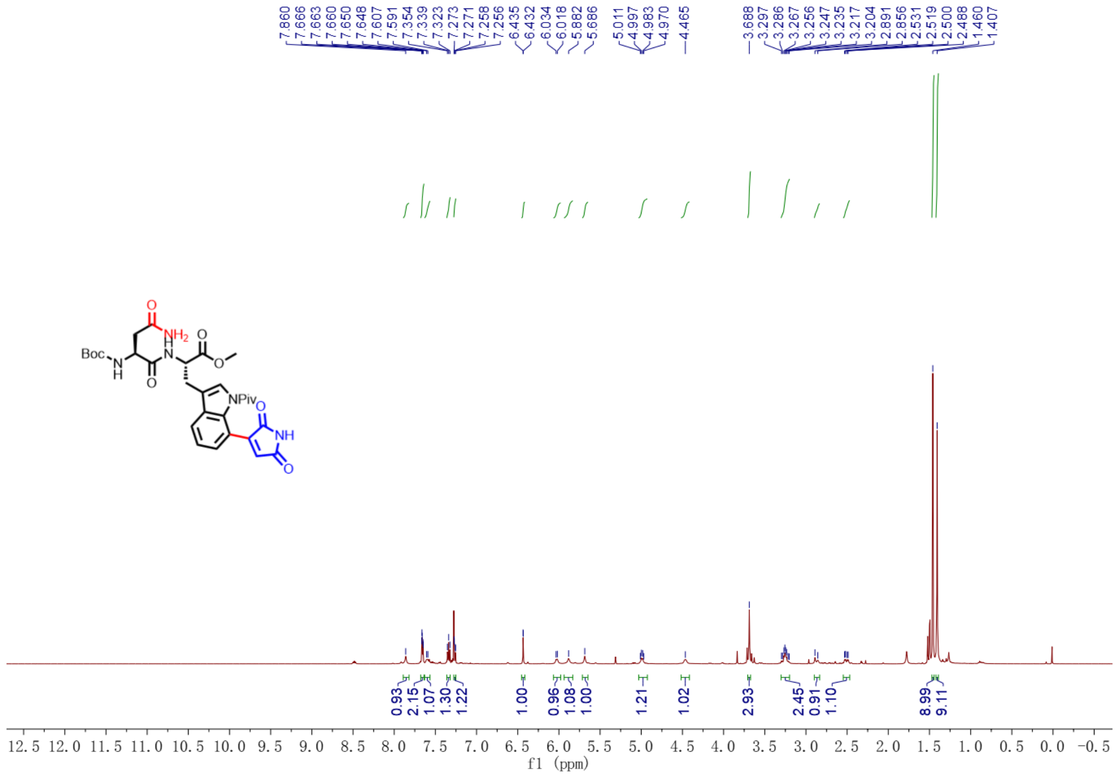

^{1}H NMR (500 MHz, $CDCl_3$) spectrum of **5j$_1$**

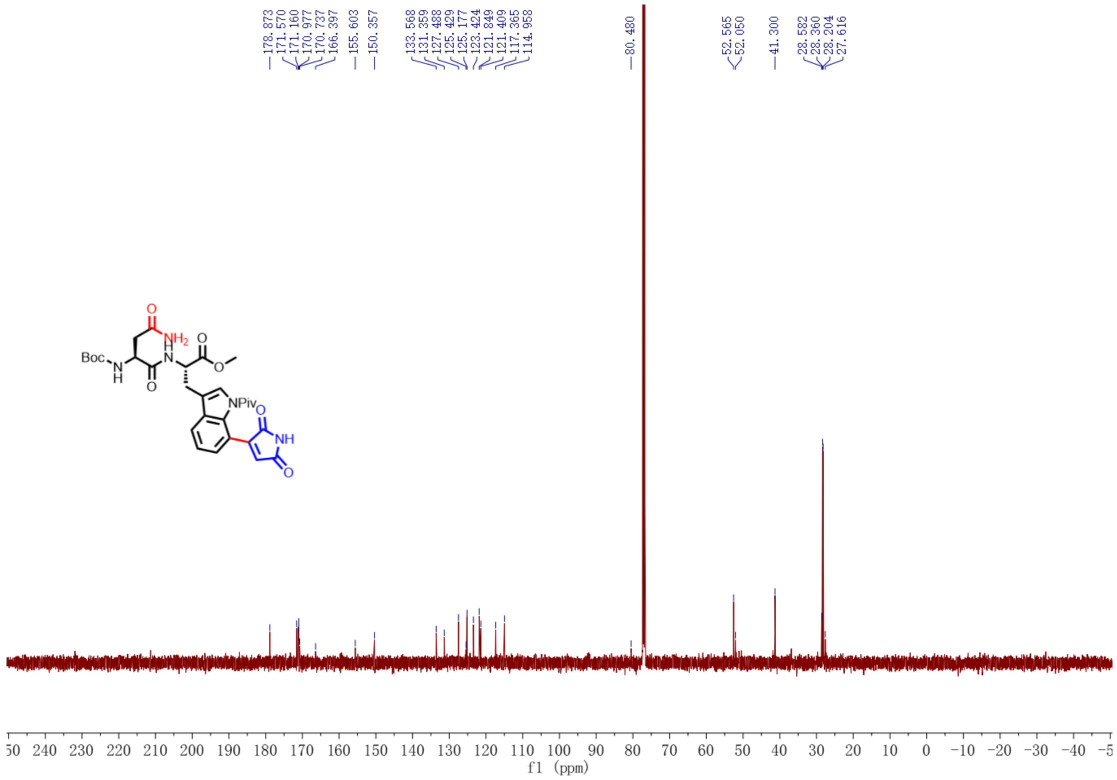

^{1}C NMR (126 MHz, $CDCl_3$) spectrum of **5j$_1$**

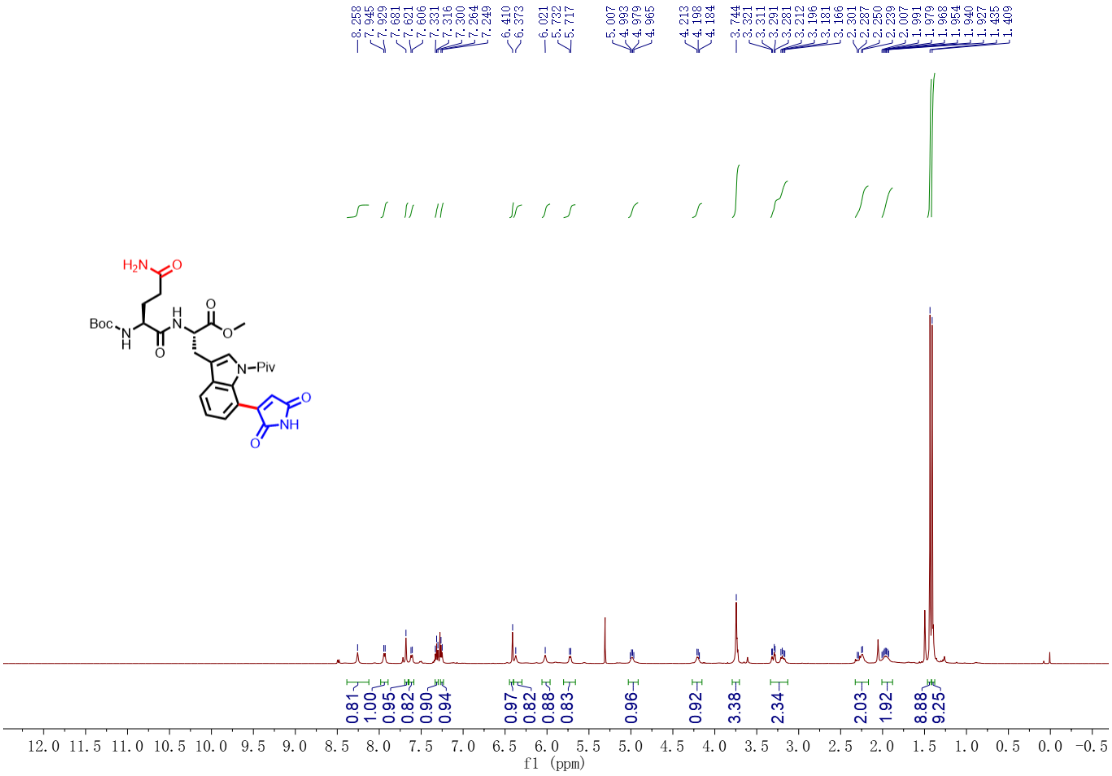

^{1}H NMR (500 MHz, $CDCl_3$) spectrum of **5j$_2$**

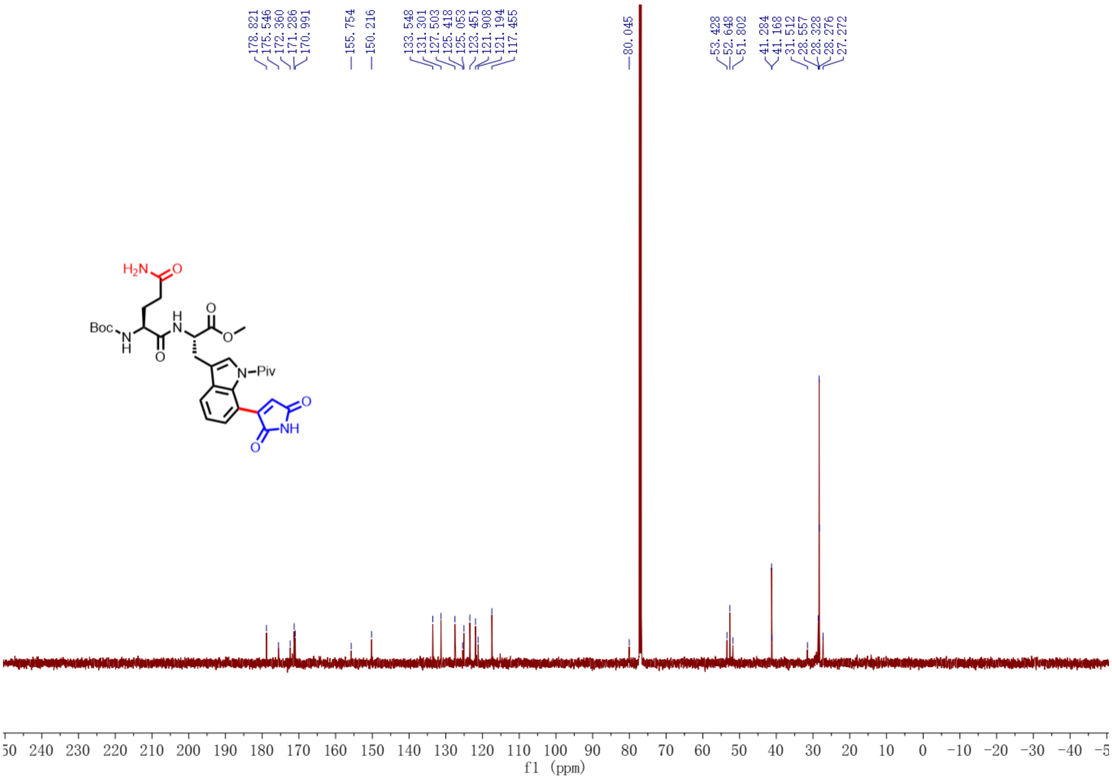

^{1}C NMR (126 MHz, $CDCl_3$) spectrum of **5j$_2$**

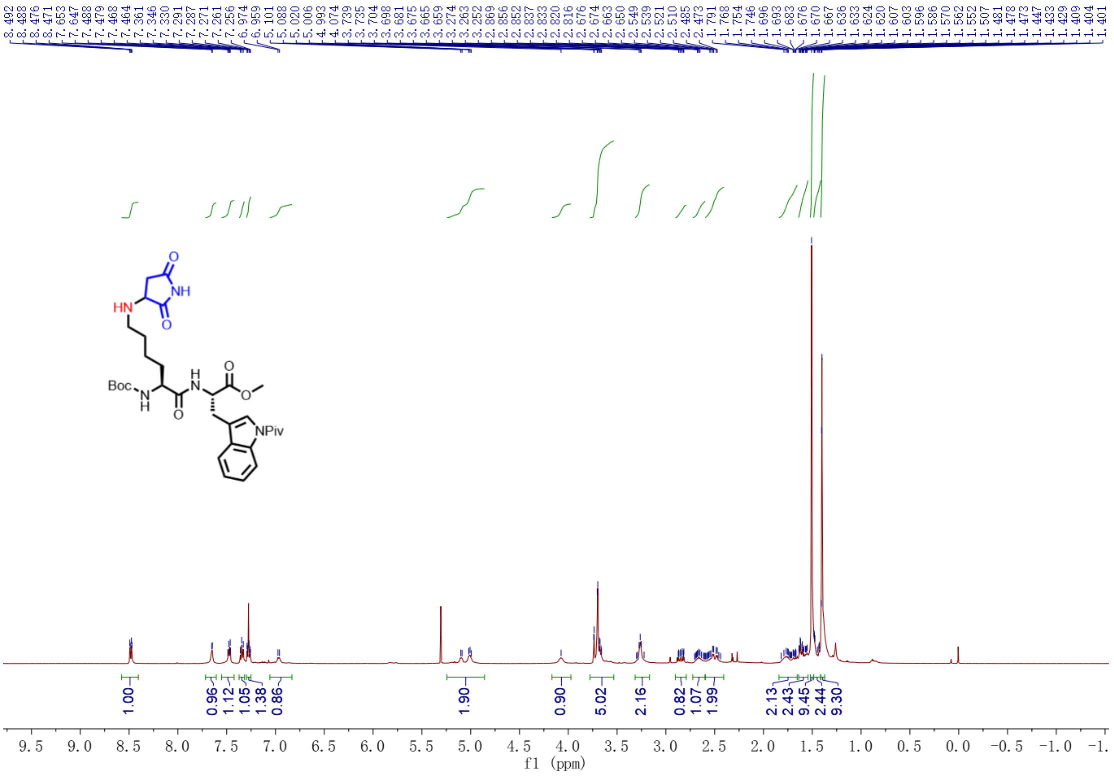

^{1}H NMR (500 MHz, $CDCl_3$) spectrum of **5k'**

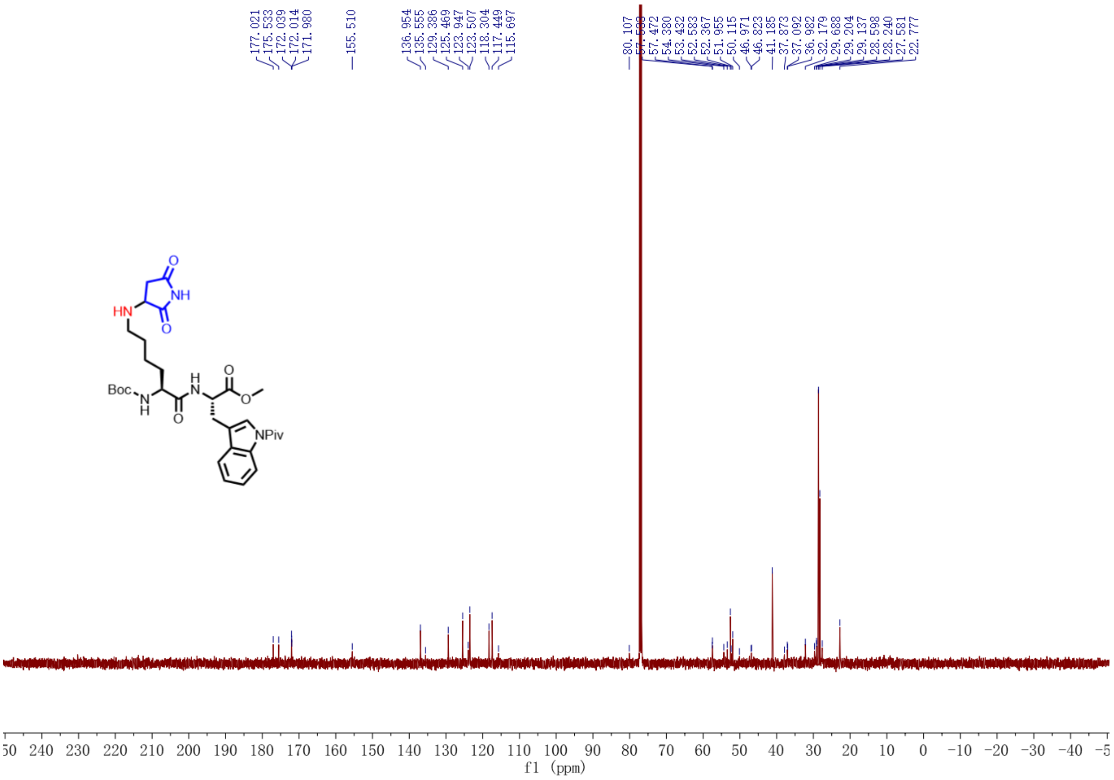

^{1}H NMR (500 MHz, $CDCl_3$) spectrum of **5k'**

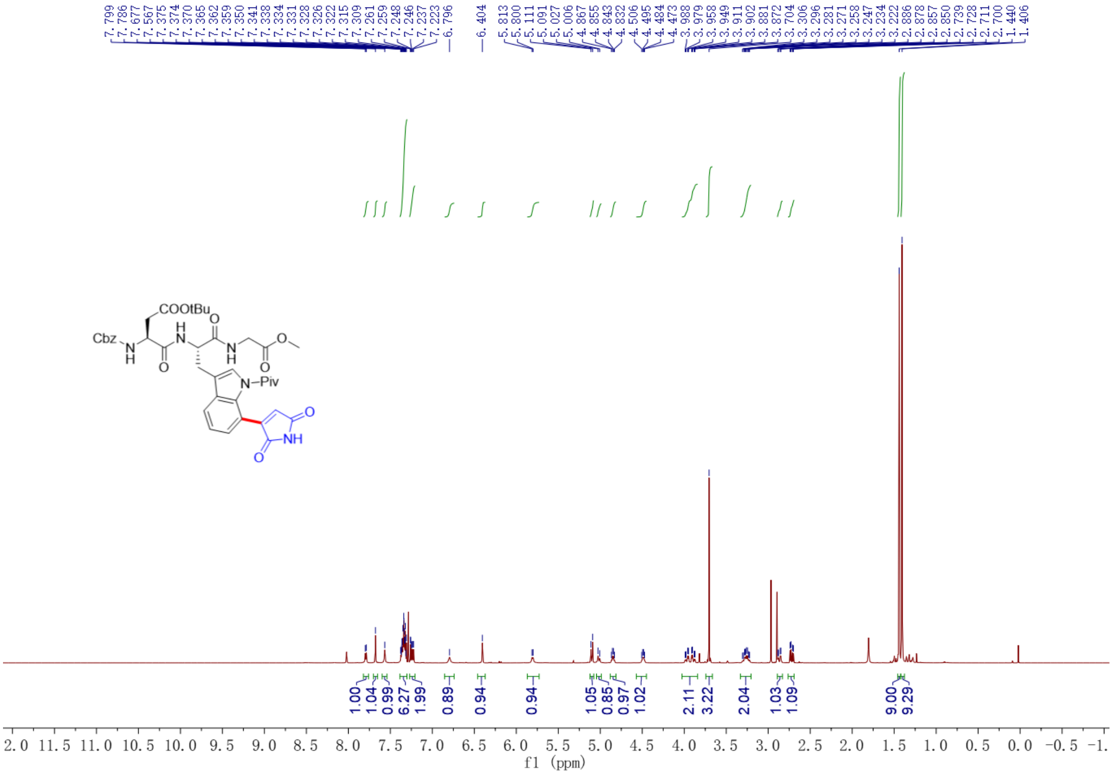

^{1}H NMR (600 MHz, $CDCl_3$) spectrum of **5m**

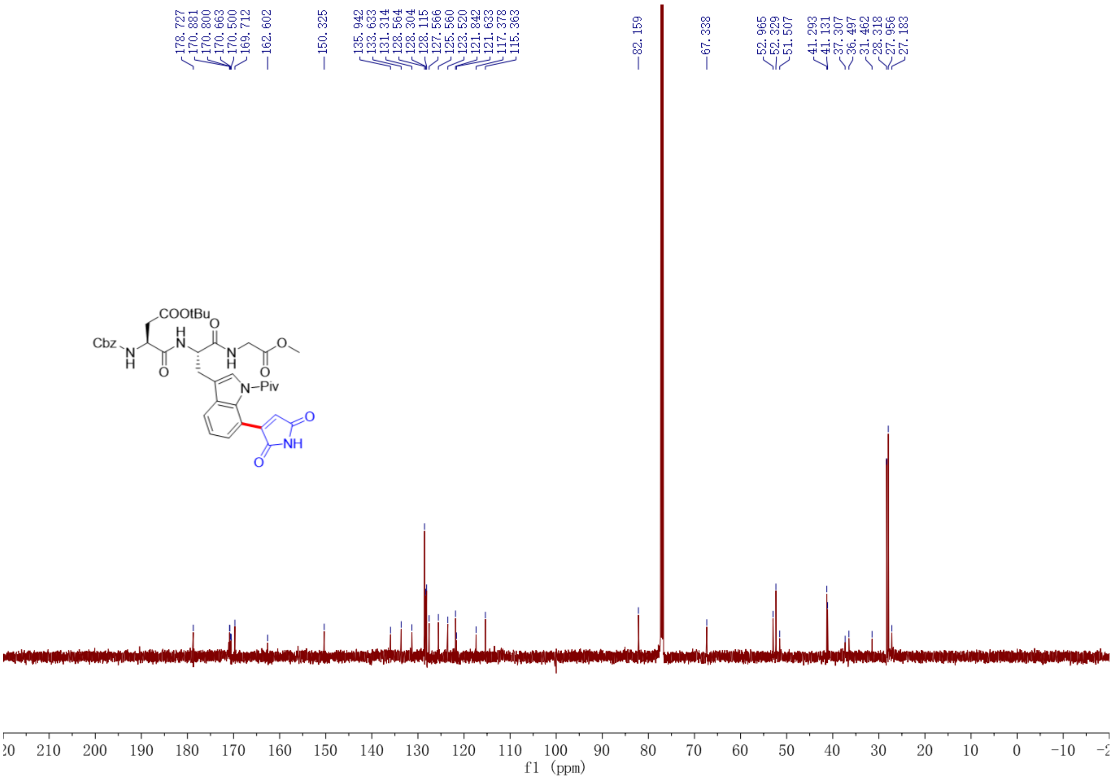

[1]C NMR (151 MHz, $CDCl_3$) spectrum of **5m**

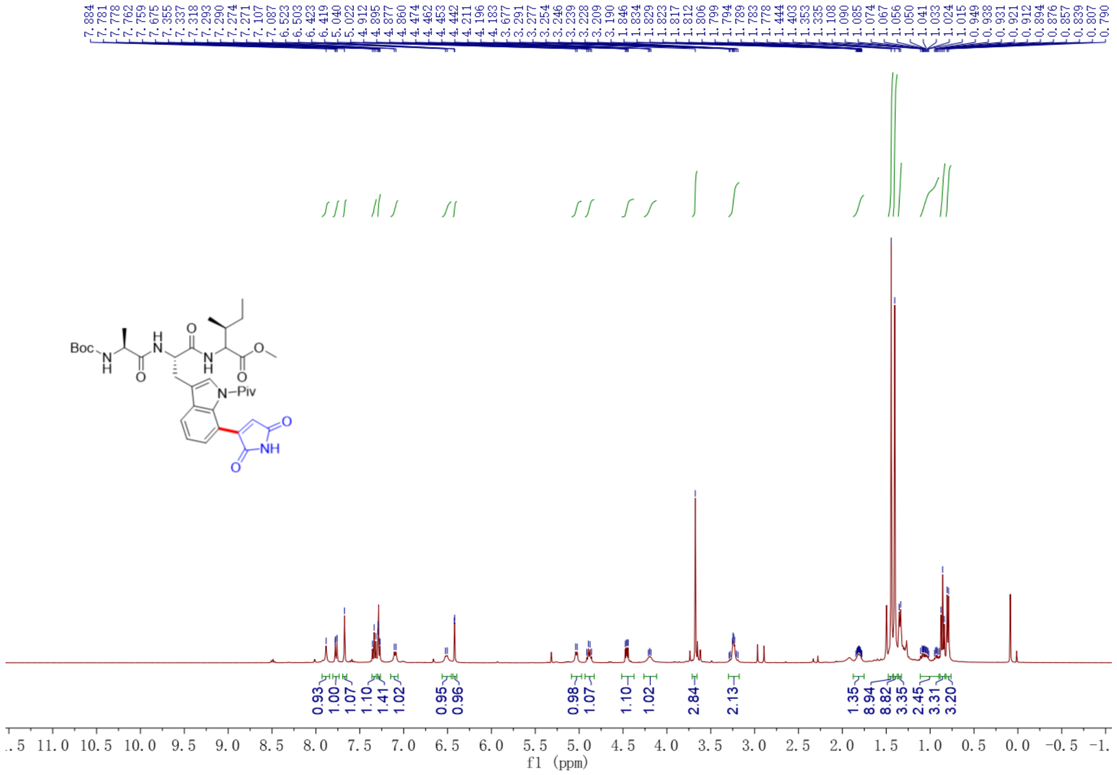

^{1}H NMR (400 MHz, $CDCl_3$) spectrum of **5n**

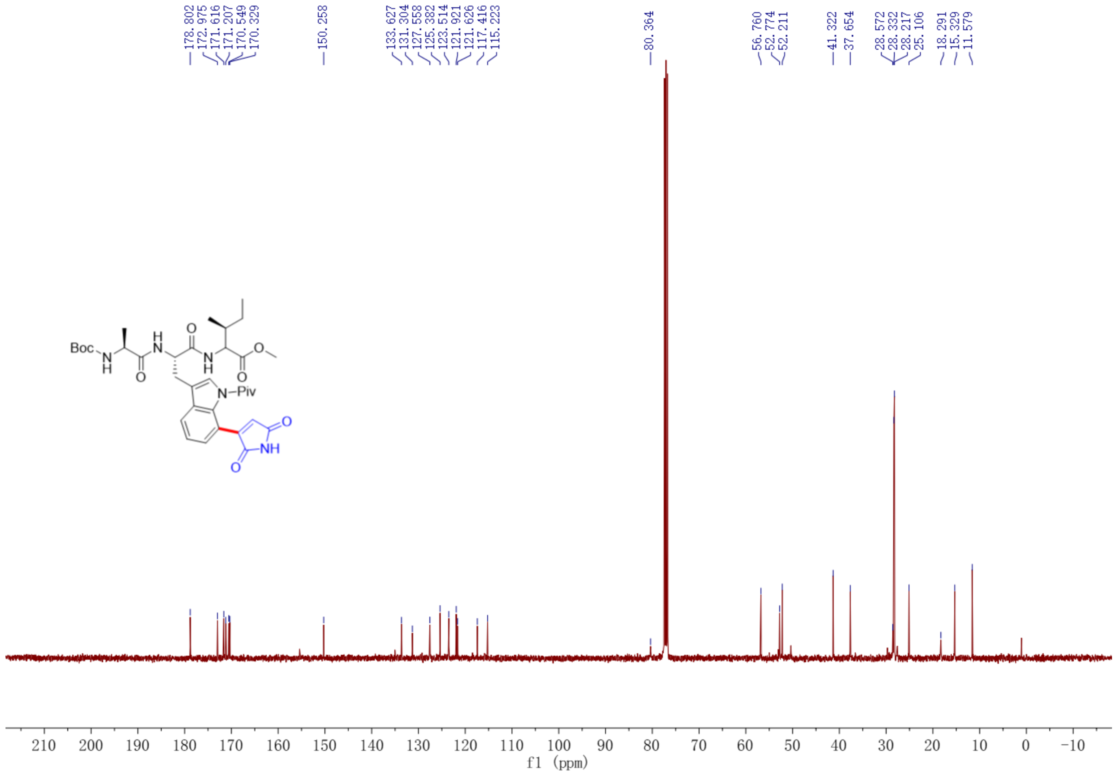

^{1}C NMR (101 MHz, $CDCl_3$) spectrum of **5n**

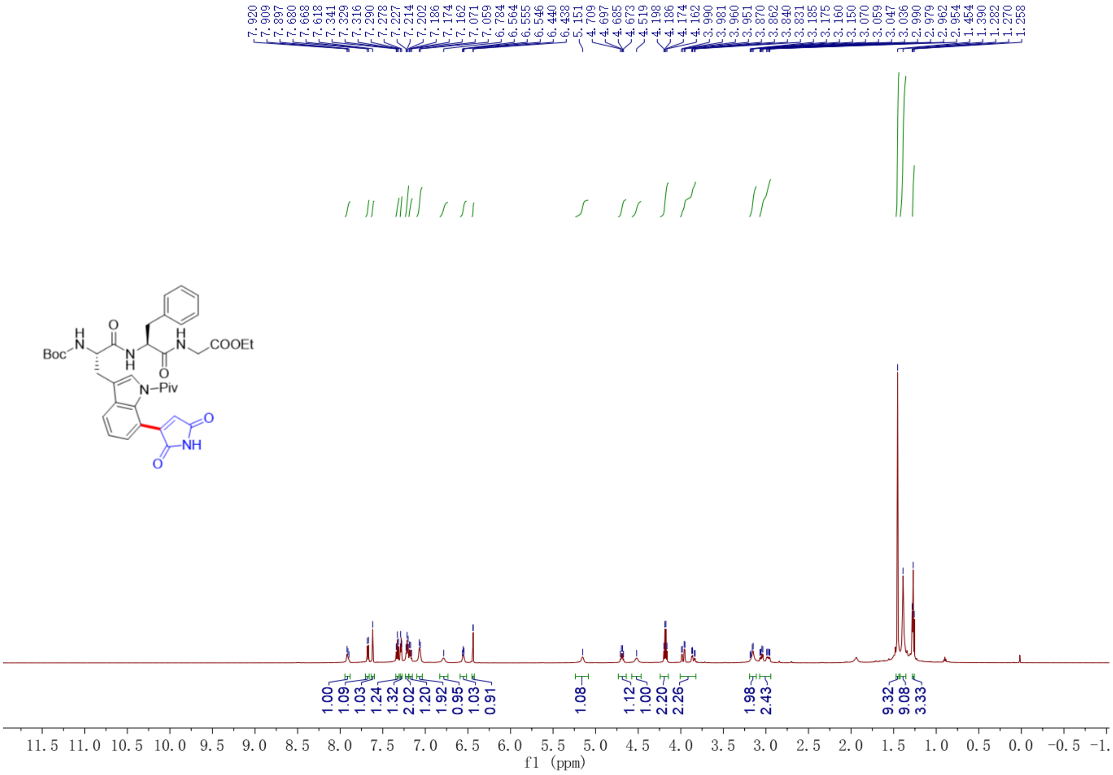

^{1}H NMR (600 MHz, $CDCl_3$) spectrum of **5o**

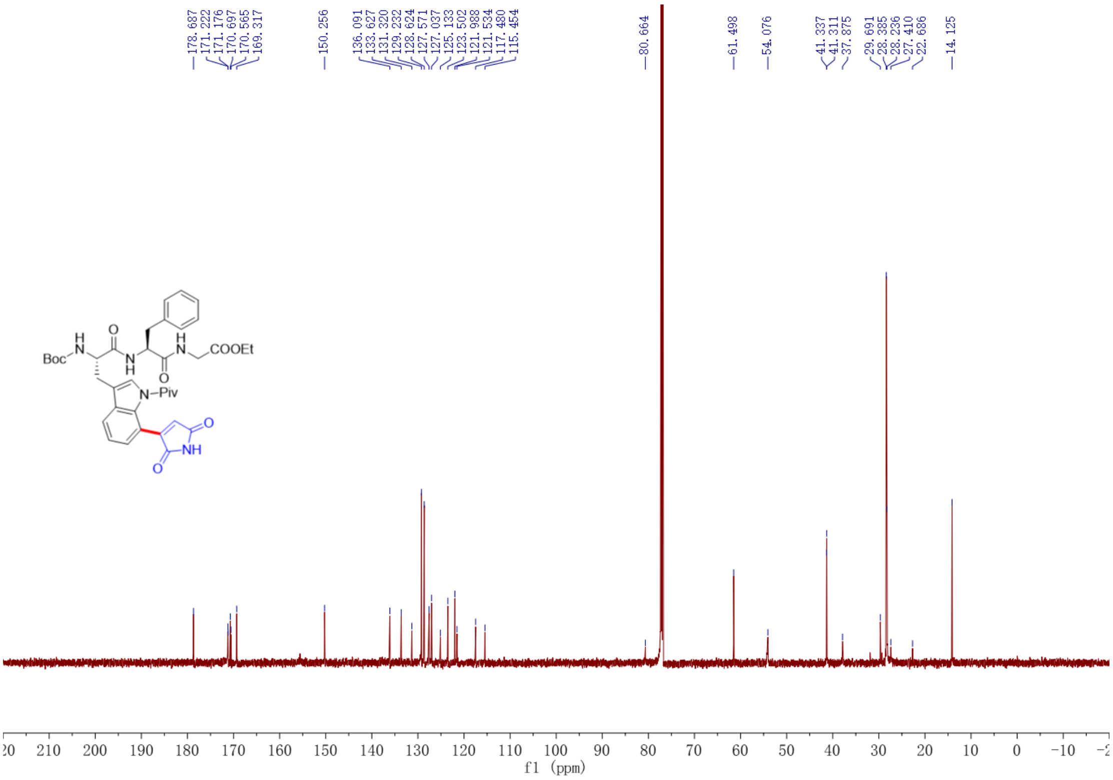

^{1}C NMR (151 MHz, $CDCl_3$) spectrum of **5o**

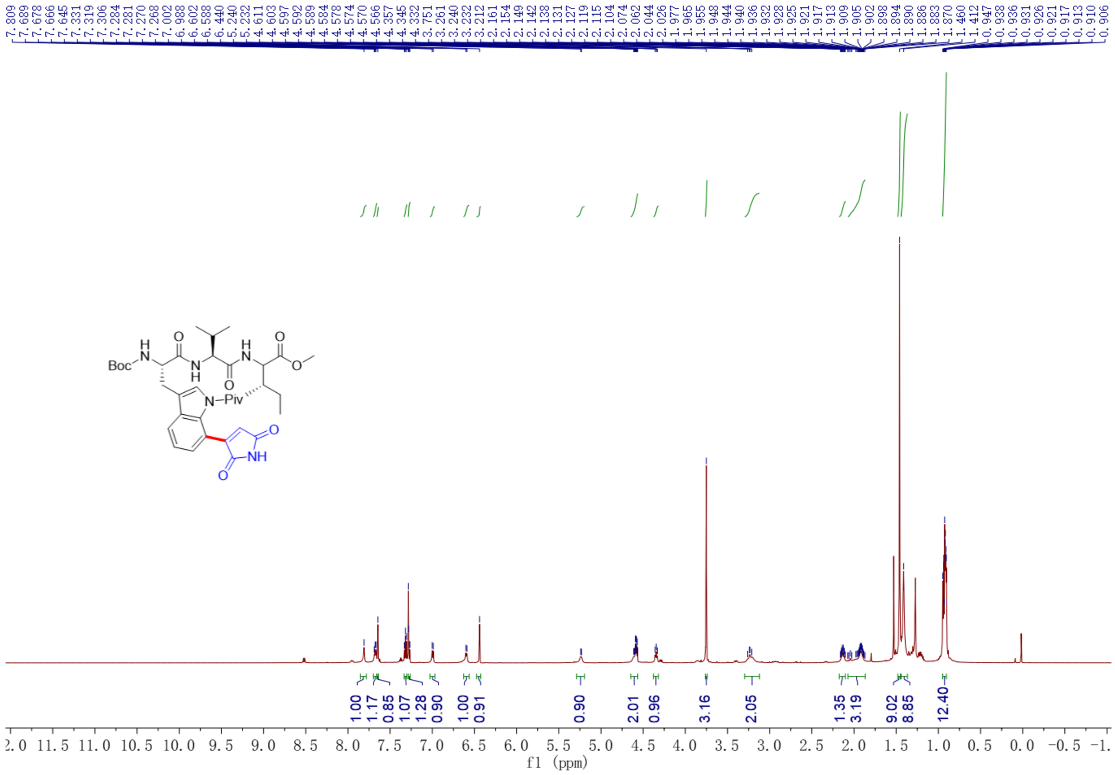

^{1}H NMR (600 MHz, $CDCl_3$) spectrum of **5p**

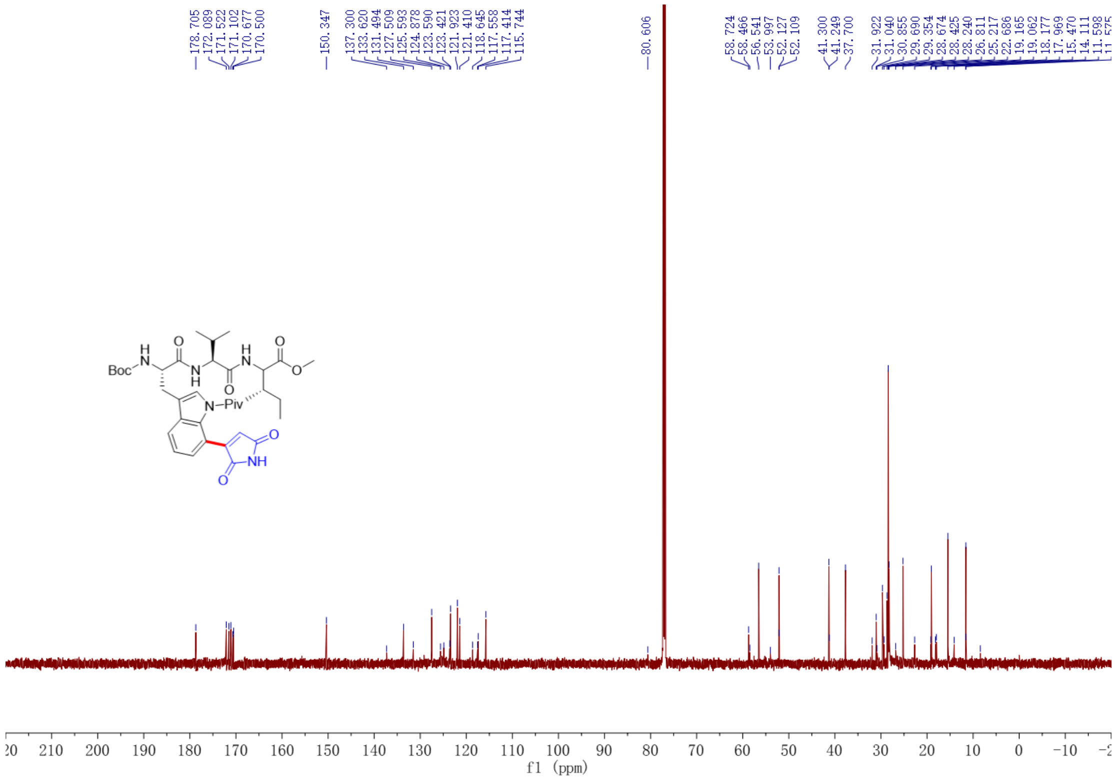

^{1}C NMR (151 MHz, $CDCl_3$) spectrum of **5p**

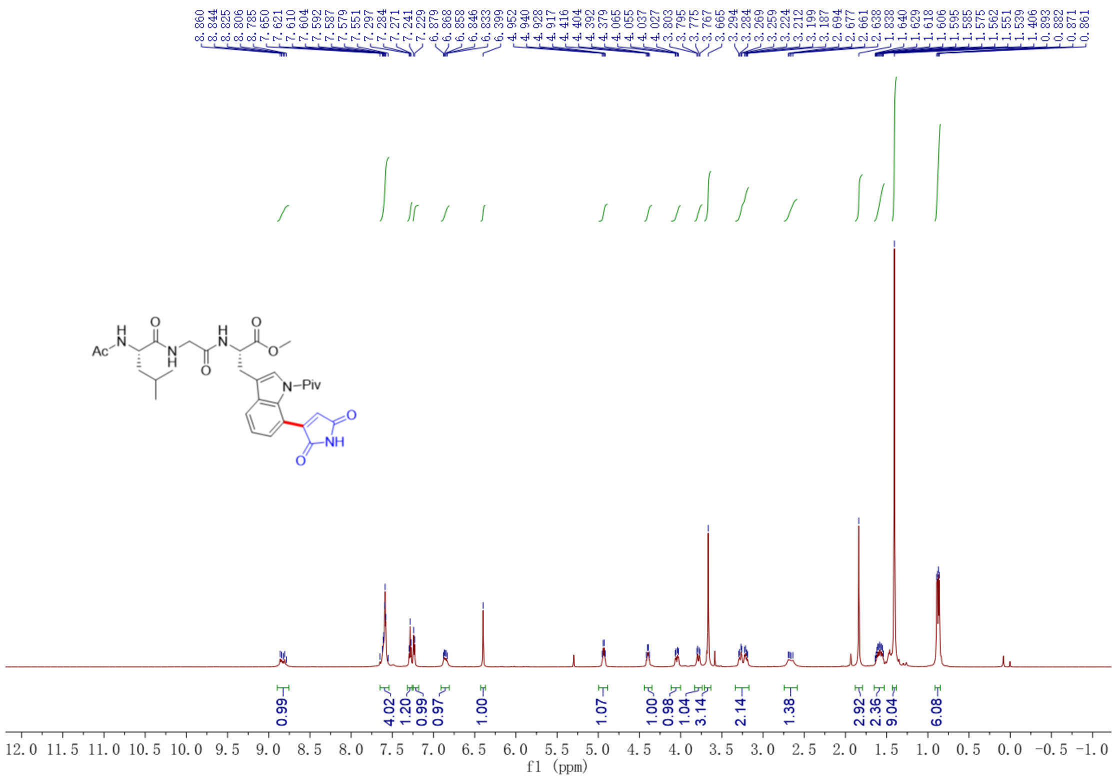

^{1}H NMR (600 MHz, $CDCl_3$) spectrum of **5q**

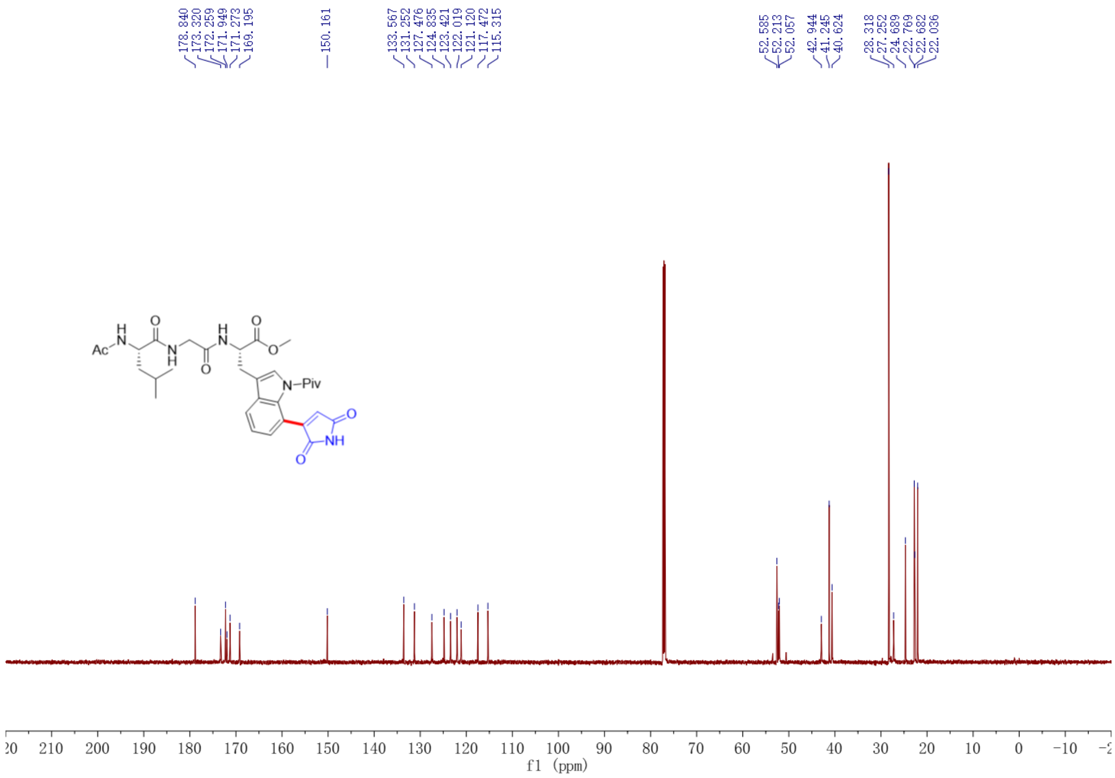

[1]C NMR (151 MHz, $CDCl_3$) spectrum of **5q**

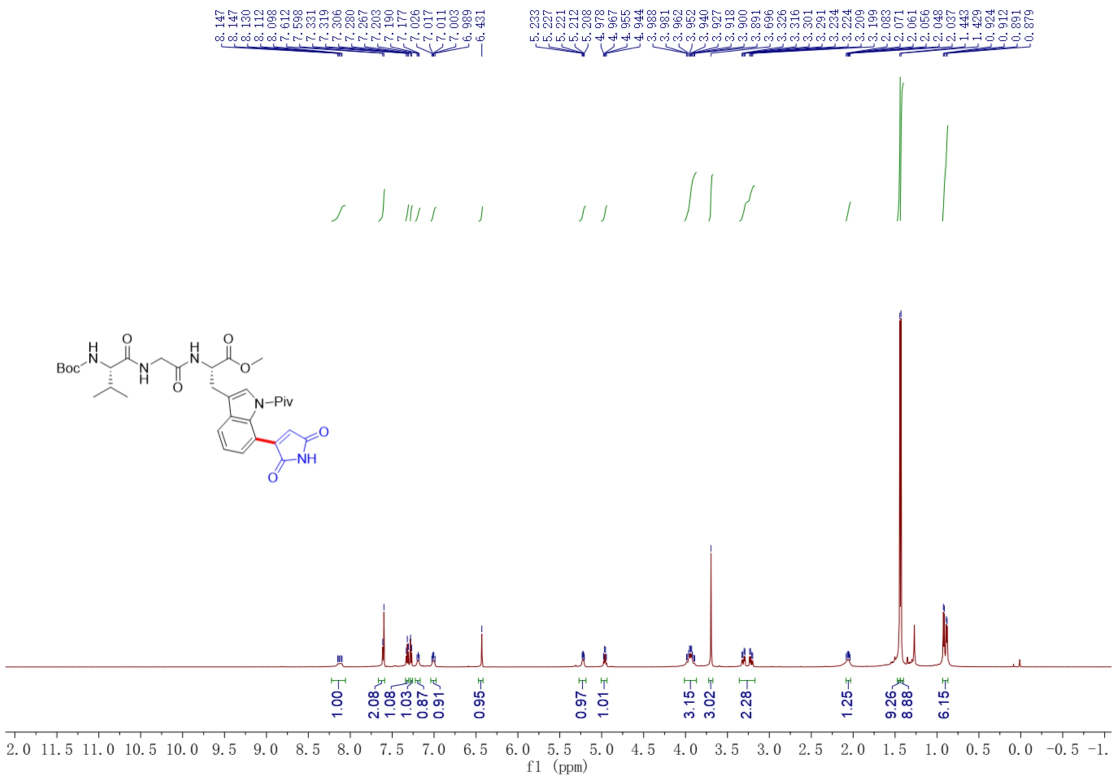

^{1}H NMR (600 MHz, $CDCl_3$) spectrum of **5r**

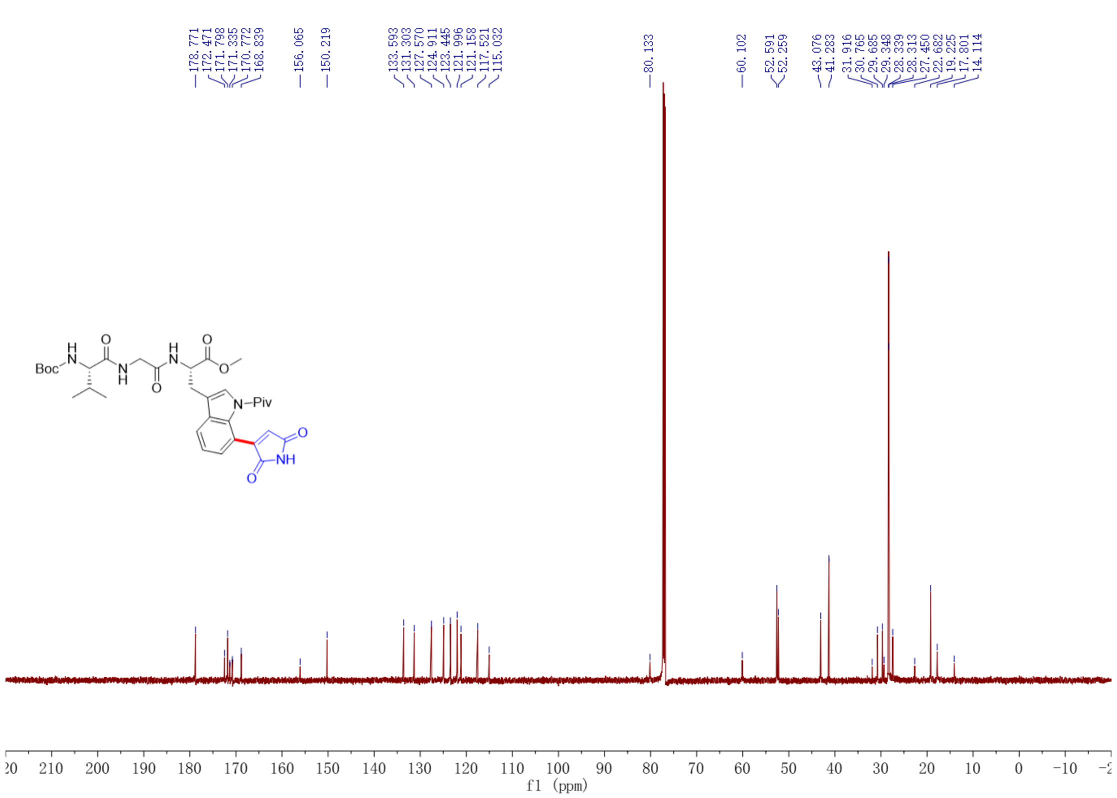

[1]C NMR (151 MHz, $CDCl_3$) spectrum of **5r**

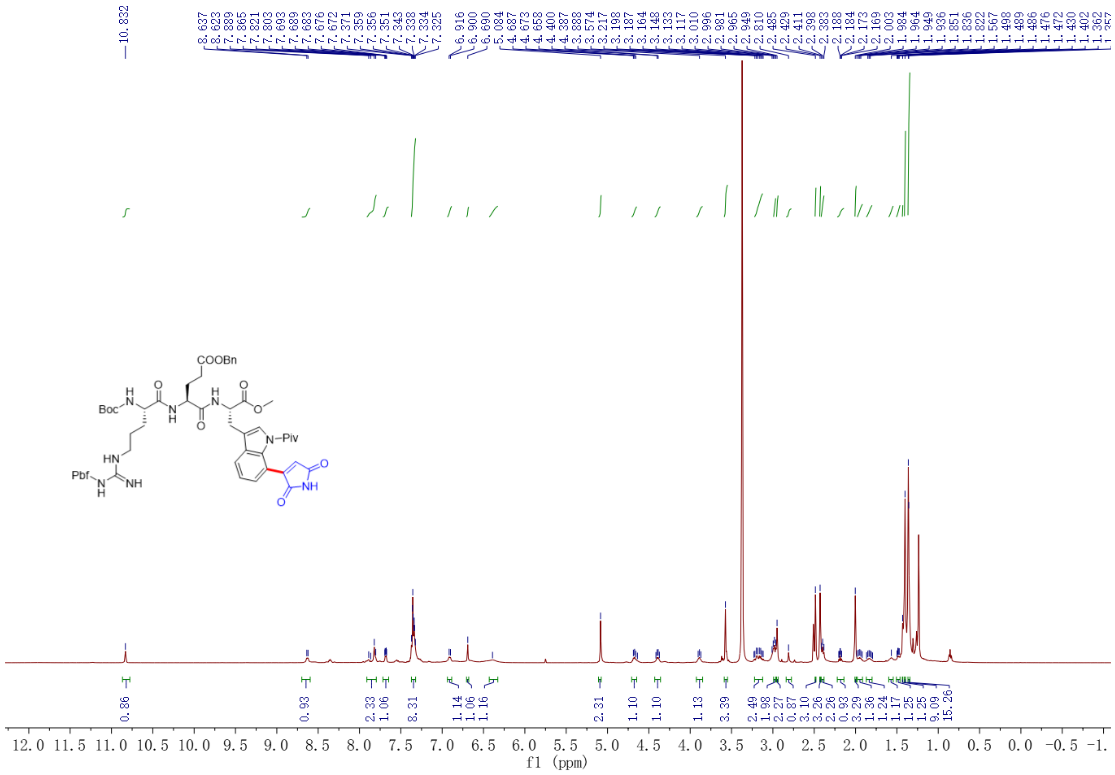

^{1}H NMR (500 MHz, DMSO) spectrum of **5s**

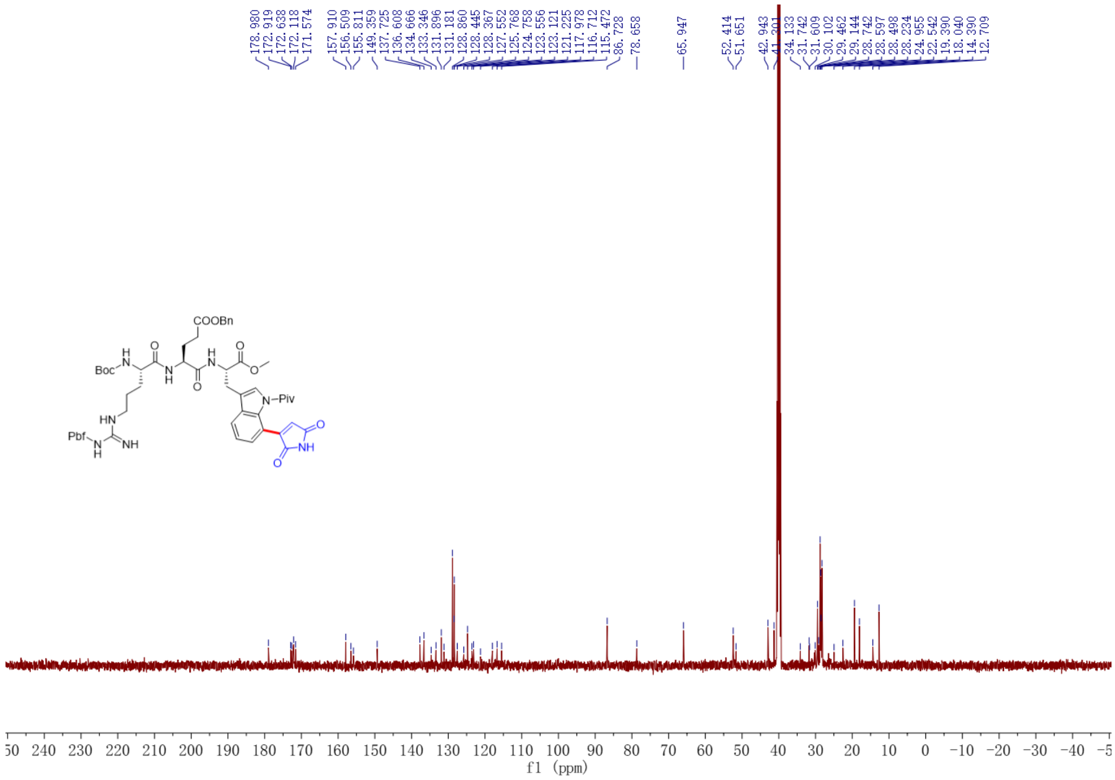

^{1}C NMR (126 MHz, DMSO) spectrum of **5s**

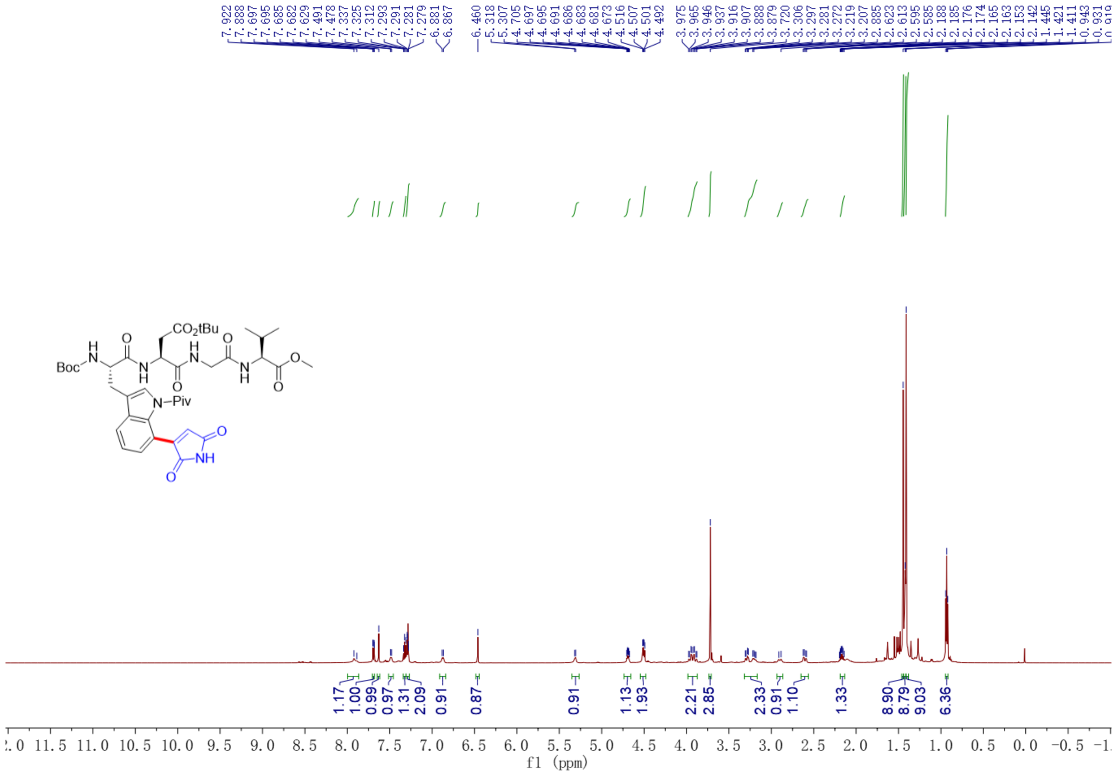

^{1}H NMR (600 MHz, $CDCl_3$) spectrum of **5t**

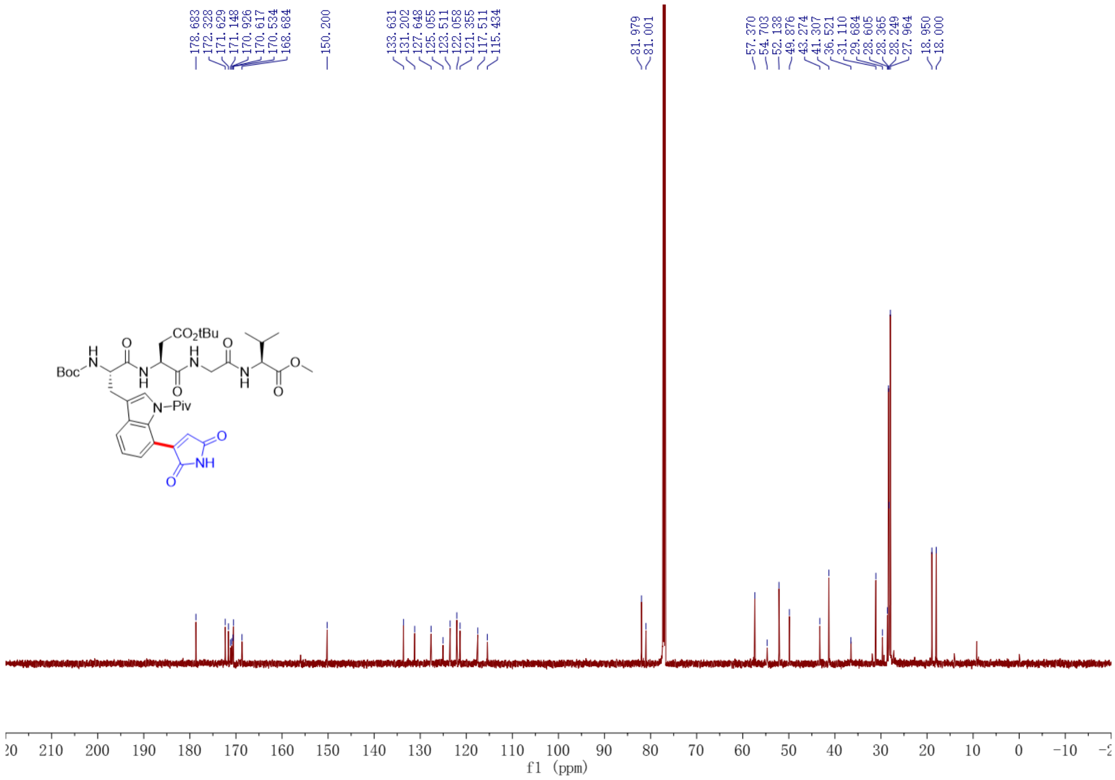

^{1}C NMR (151 MHz, $CDCl_3$) spectrum of **5t**

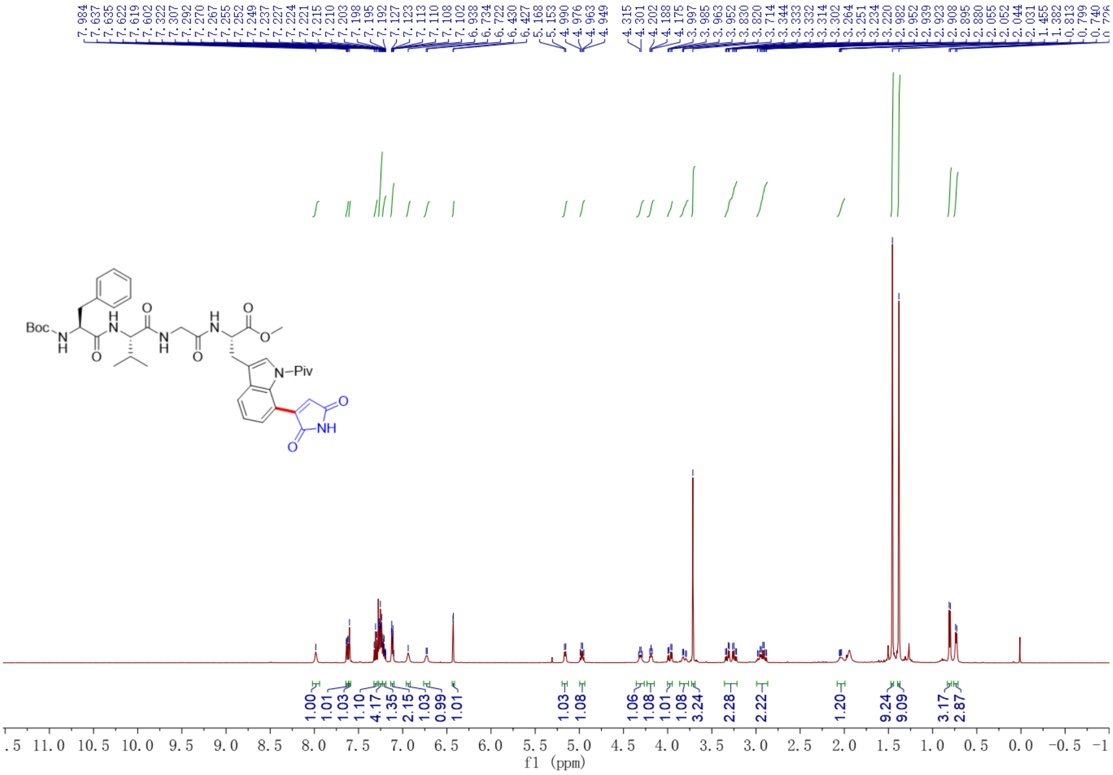

^{1}H NMR (500 MHz, $CDCl_3$) spectrum of **5u**

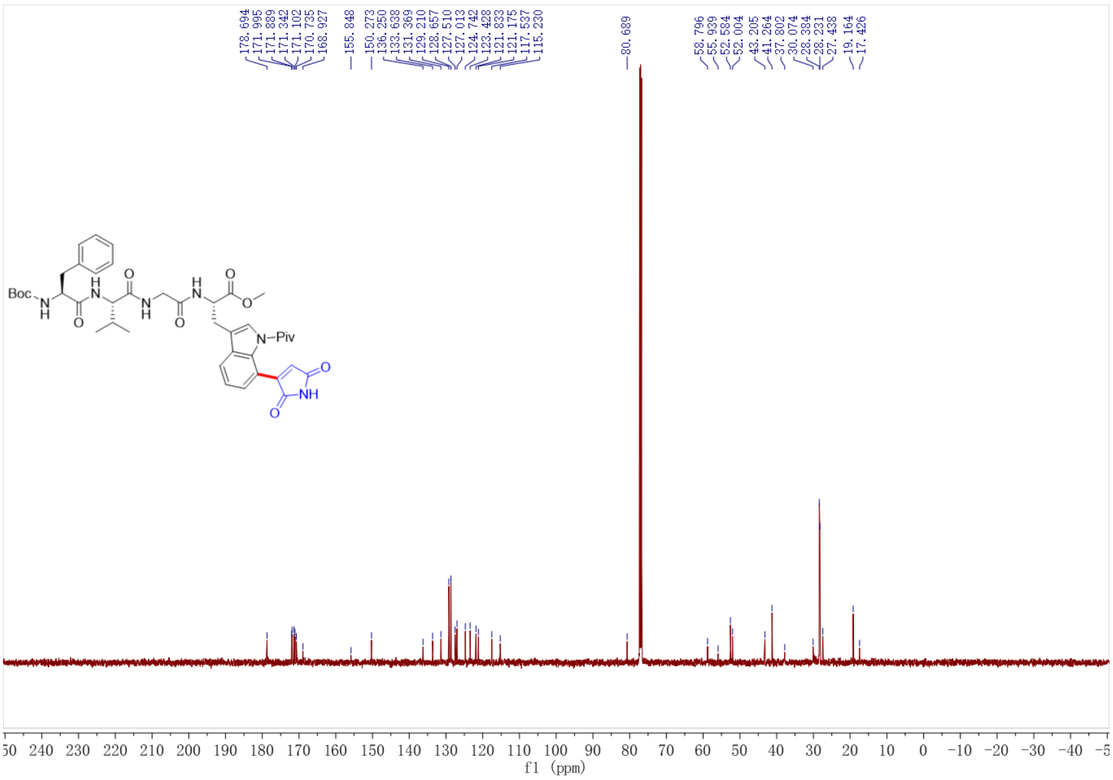

^{1}C NMR (126 MHz, $CDCl_3$) spectrum of **5u**

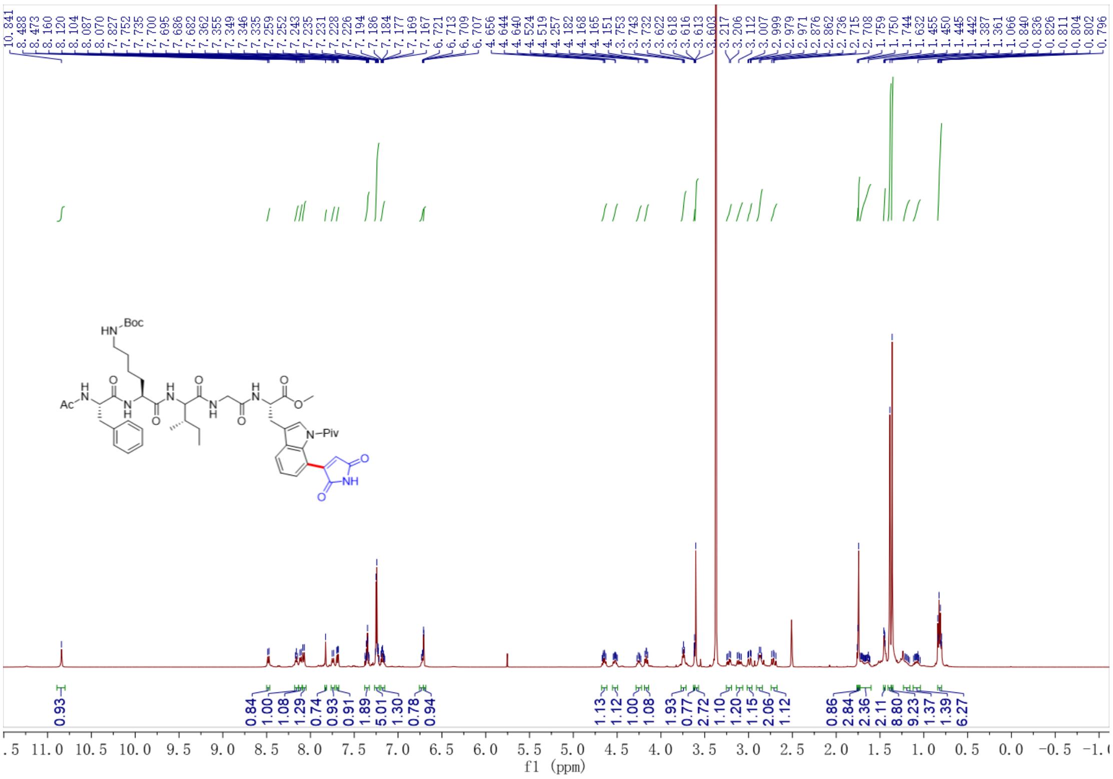

^{1}H NMR (500 MHz, DMSO) spectrum of **5v**

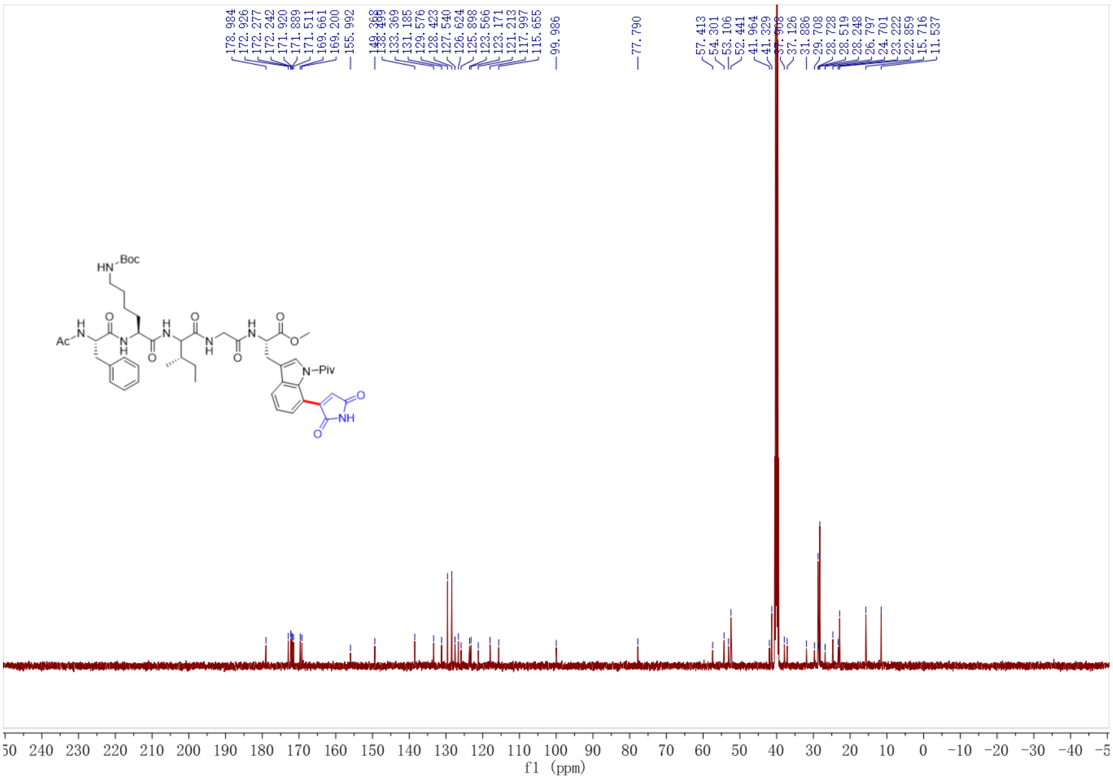

[1]C NMR (126 MHz, DMSO) spectrum of **5v**

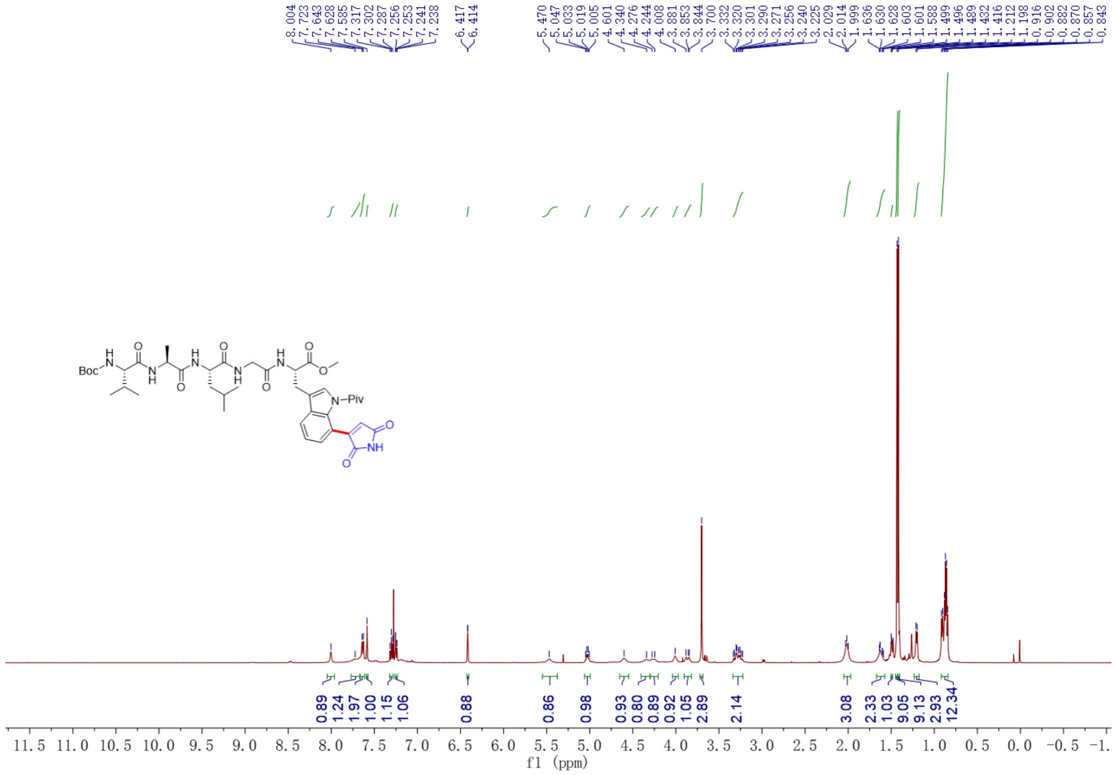

^{1}H NMR (500 MHz, $CDCl_3$) spectrum of **5w**

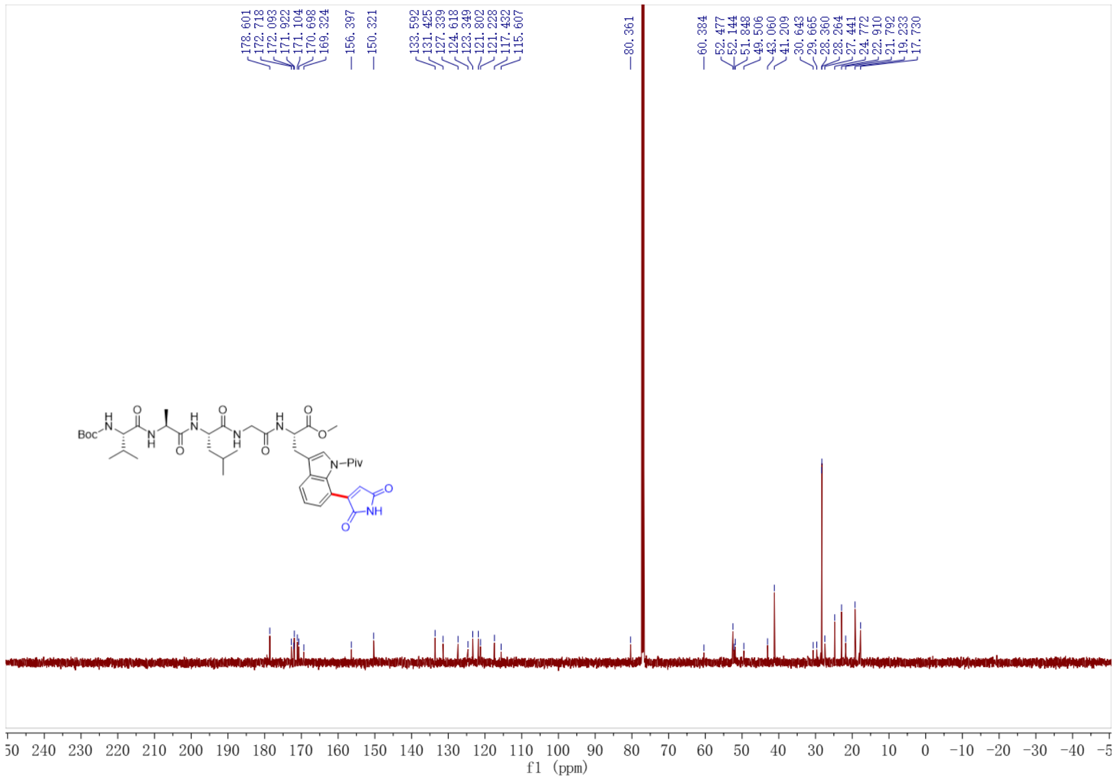

^{1}C NMR (126 MHz, $CDCl_3$) spectrum of **5w**

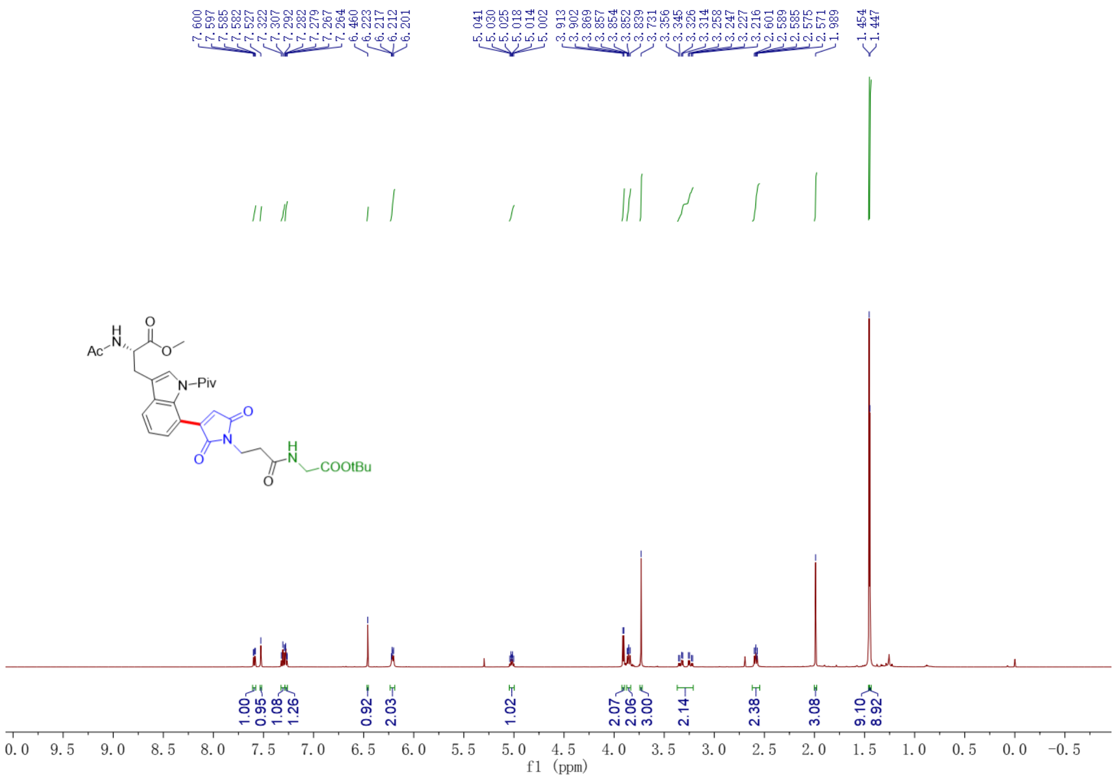

^{1}H NMR (500 MHz, $CDCl_3$) spectrum of **7a**

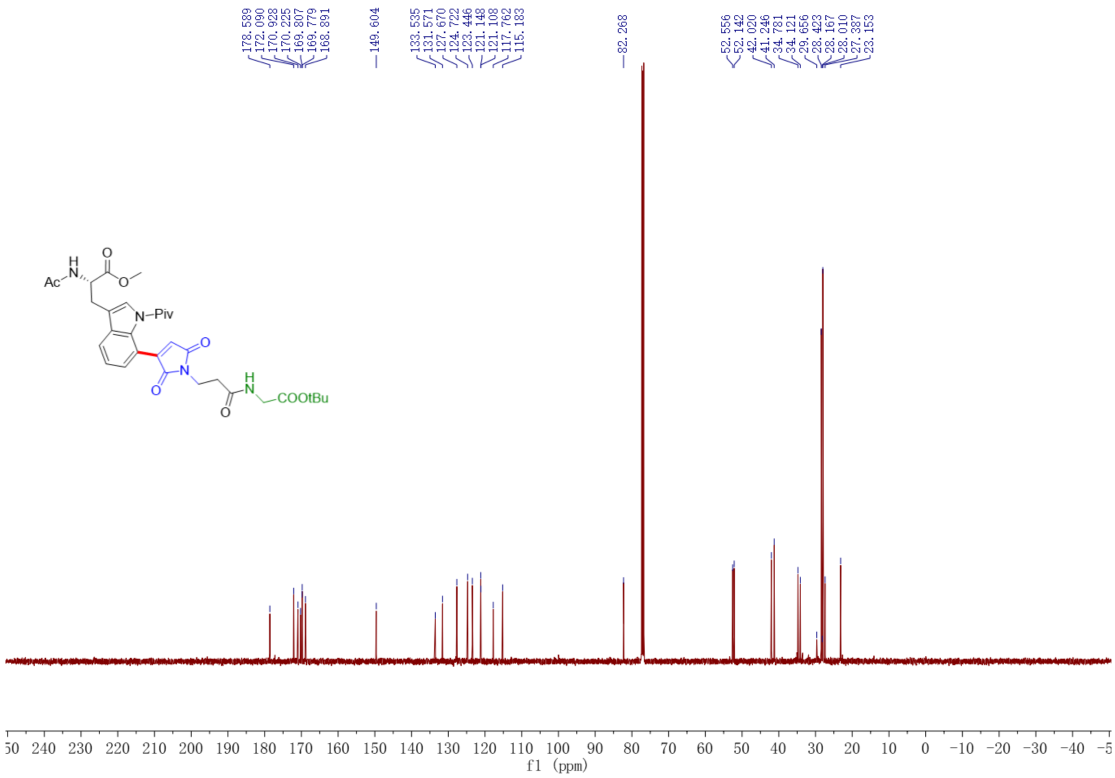

^{1}C NMR (126 MHz, $CDCl_3$) spectrum of **7a**

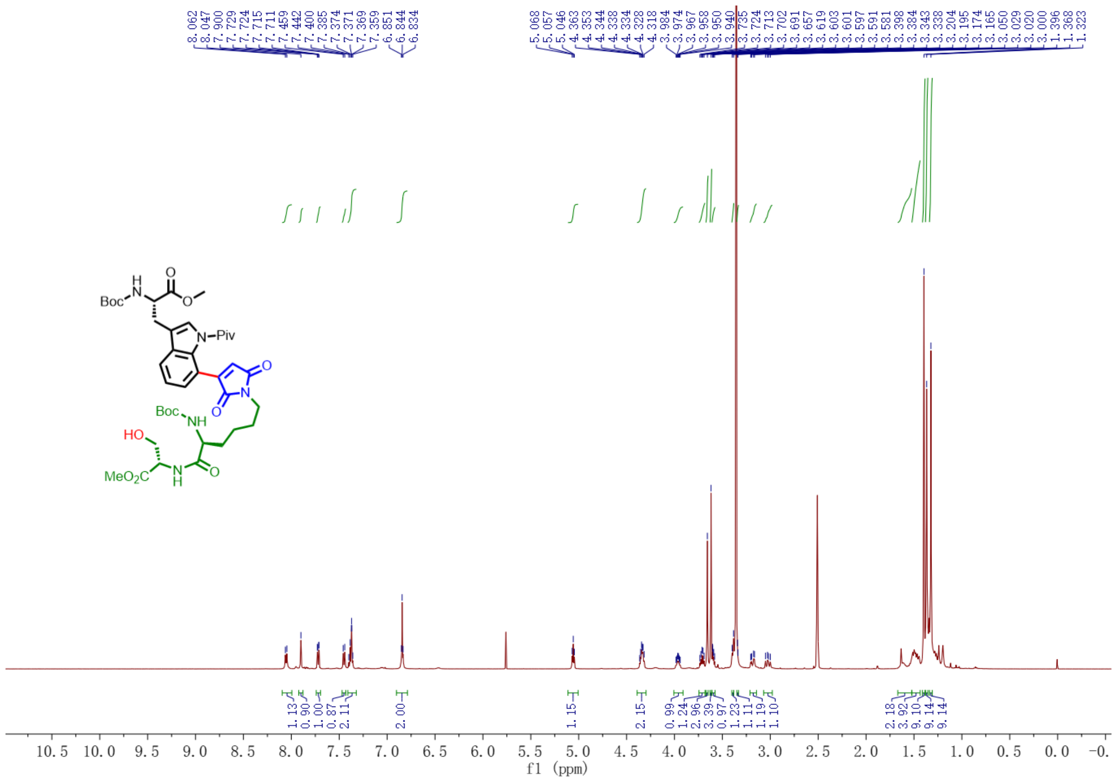

^{1}H NMR (500 MHz, DMSO) spectrum of **7b**

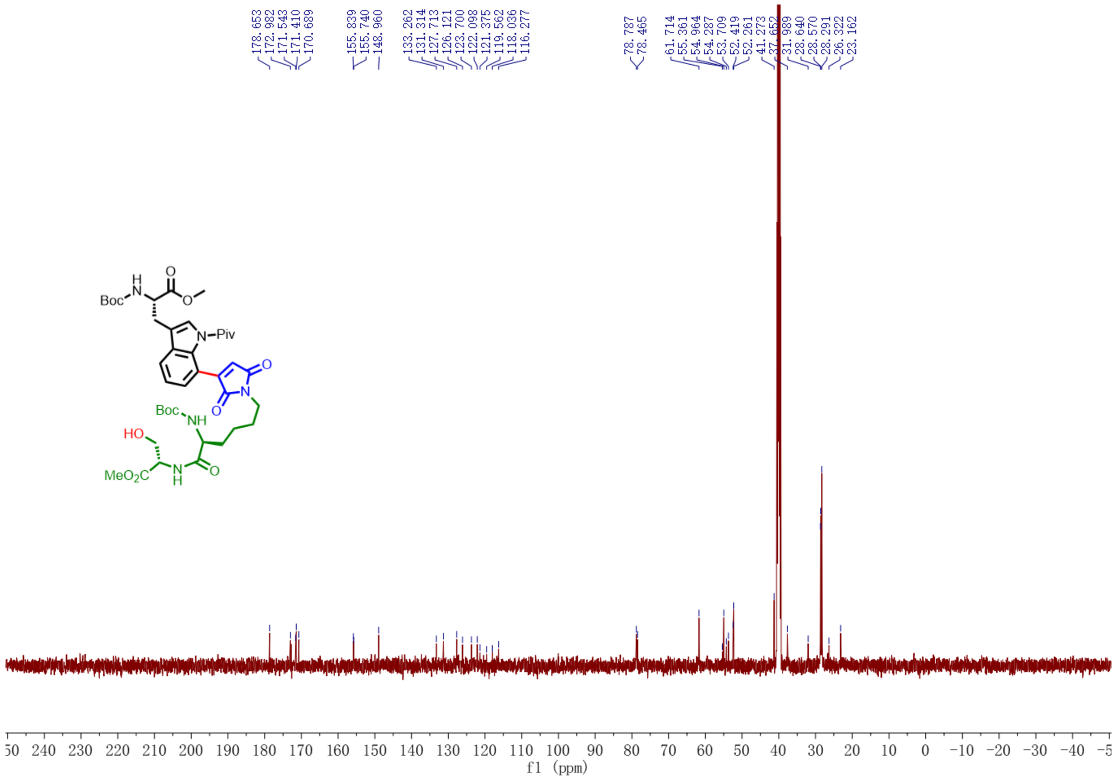

^{1}C NMR (126 MHz, DMSO) spectrum of **7b**

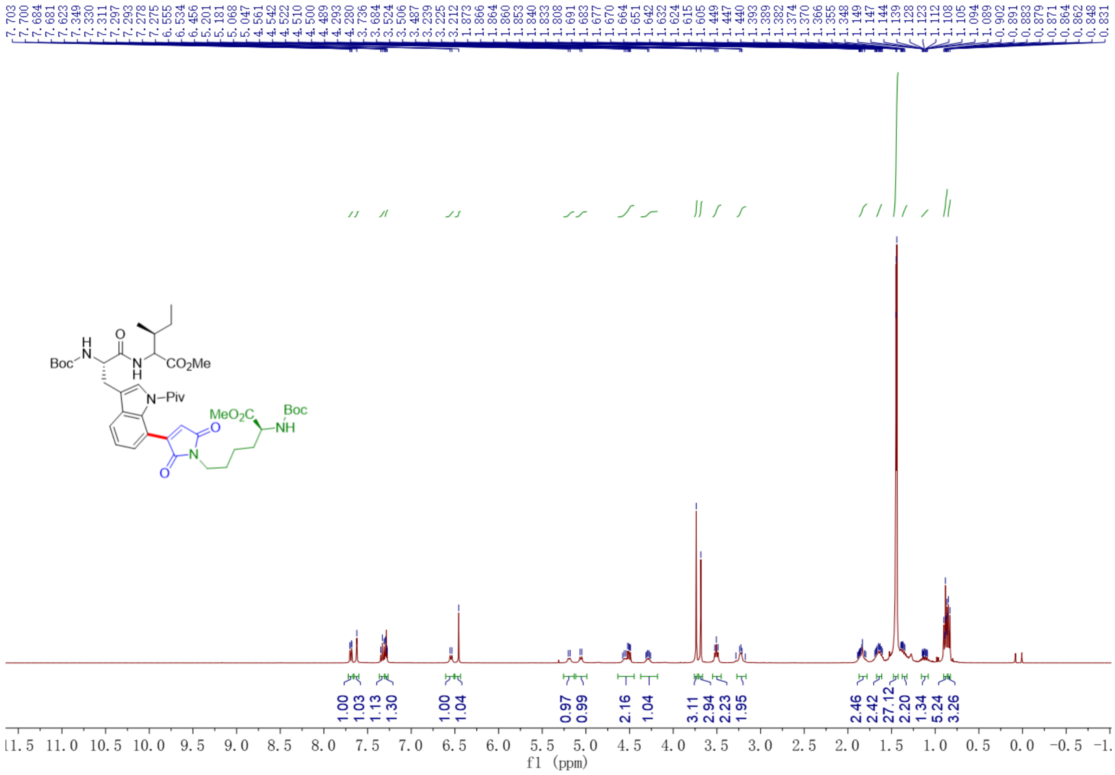

^{1}H NMR (400 MHz, $CDCl_3$) spectrum of **7c**

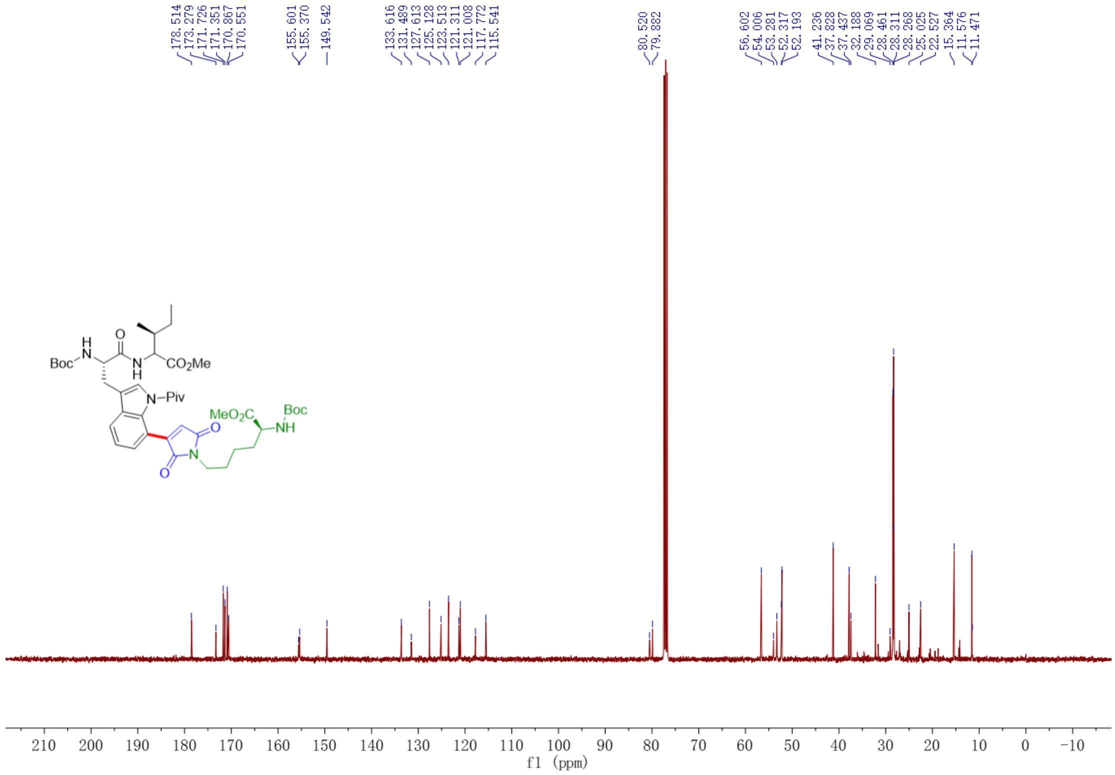

^{1}C NMR (101 MHz, $CDCl_3$) spectrum of **7c**

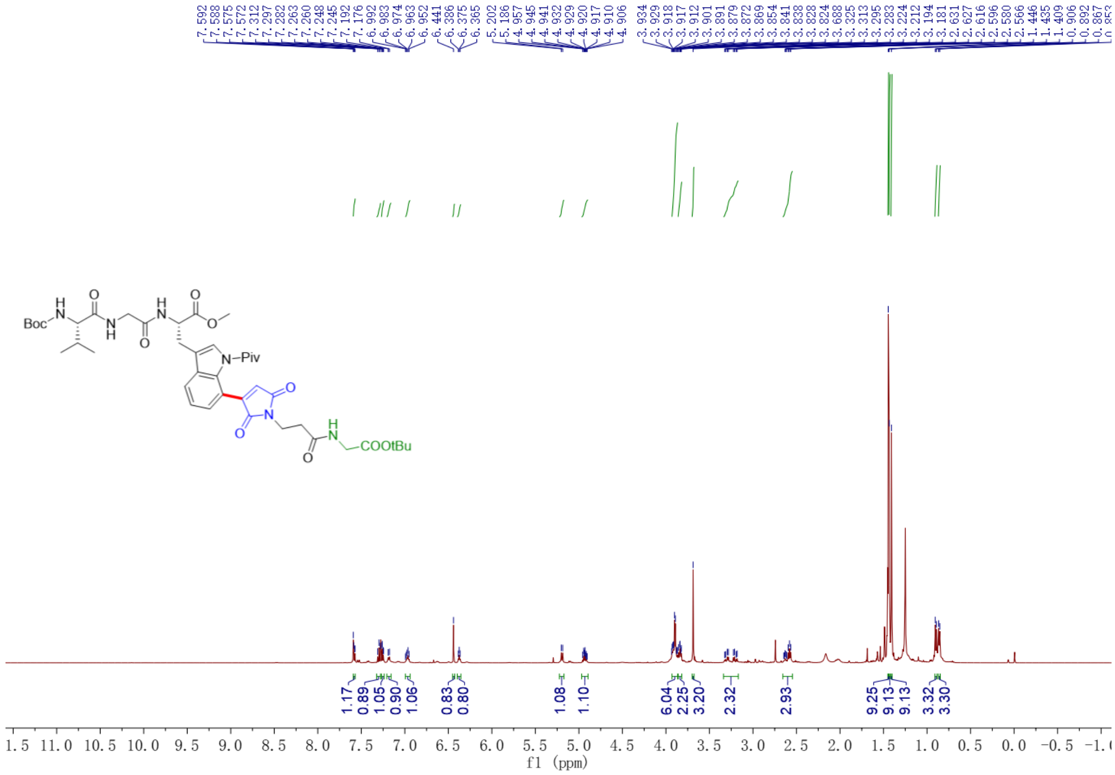

^{1}H NMR (500 MHz, $CDCl_3$) spectrum of **7d**

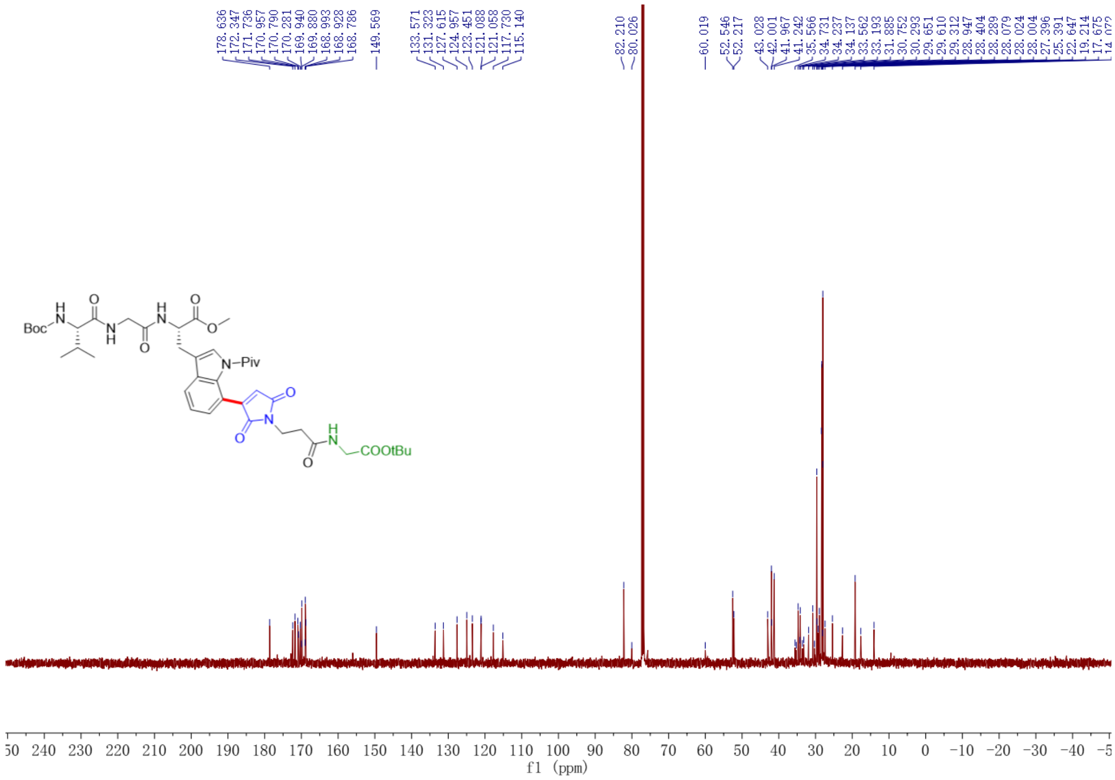

[1]C NMR (126 MHz, $CDCl_3$) spectrum of **7d**

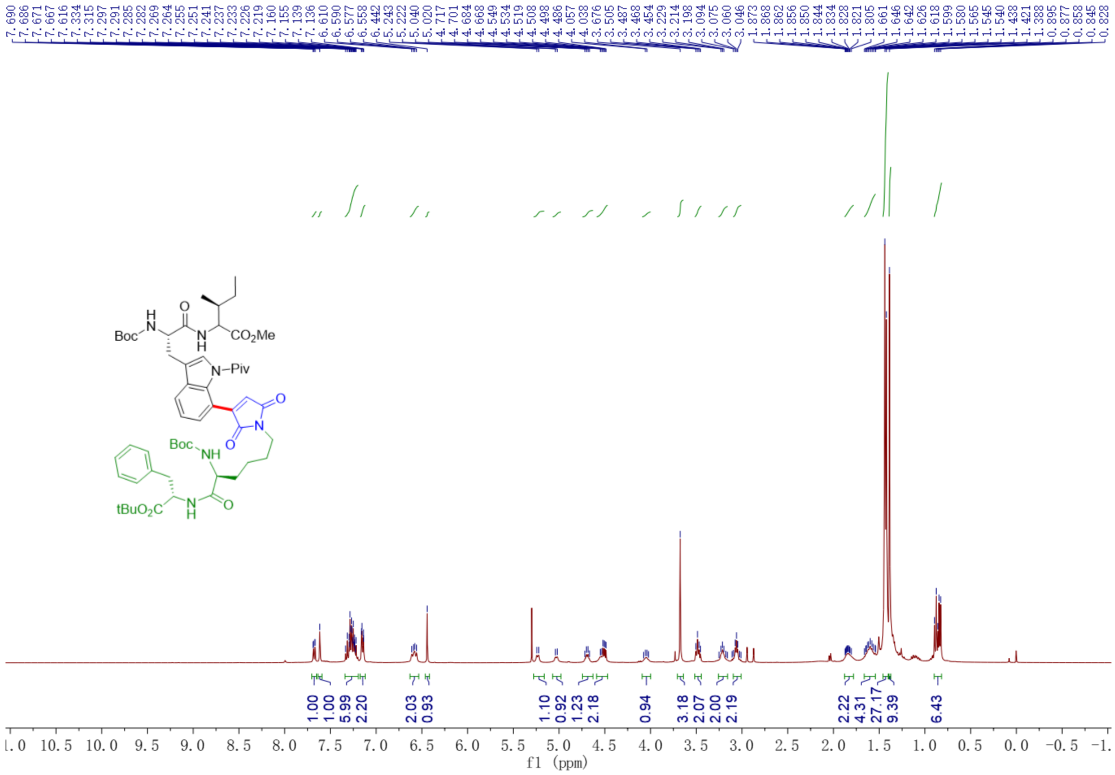

^{1}H NMR (400 MHz, $CDCl_3$) spectrum of **7e**

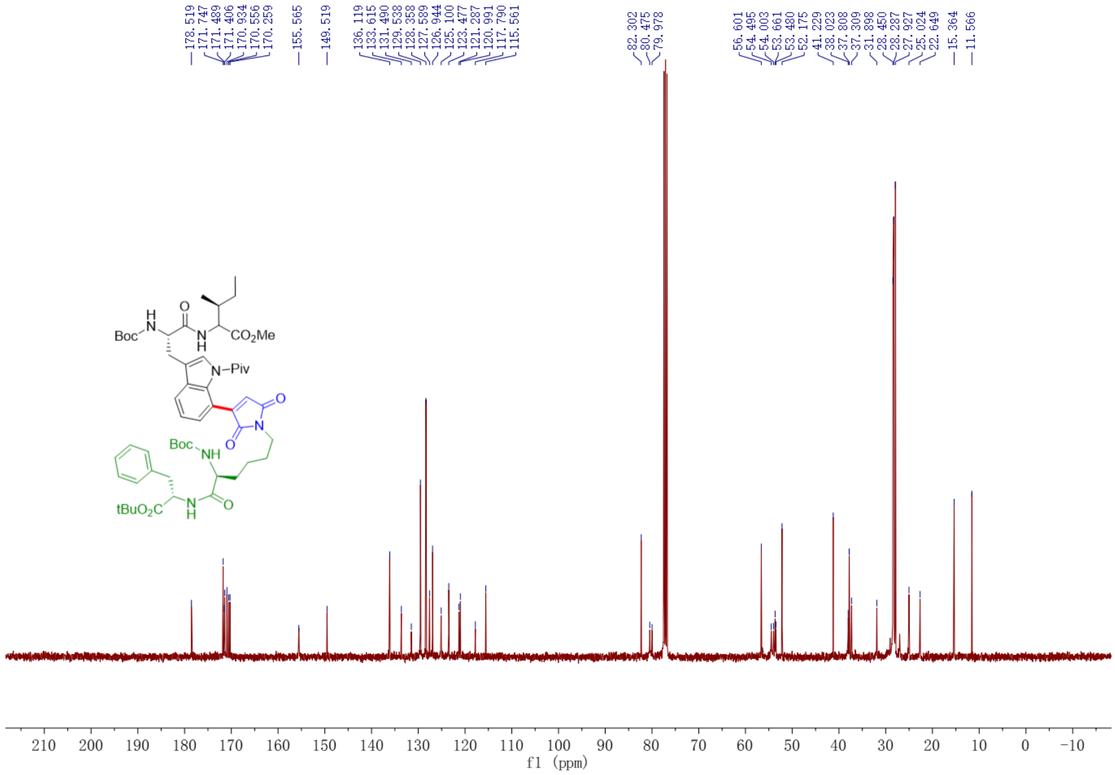

^{1}C NMR (101 MHz, $CDCl_3$) spectrum of **7e**

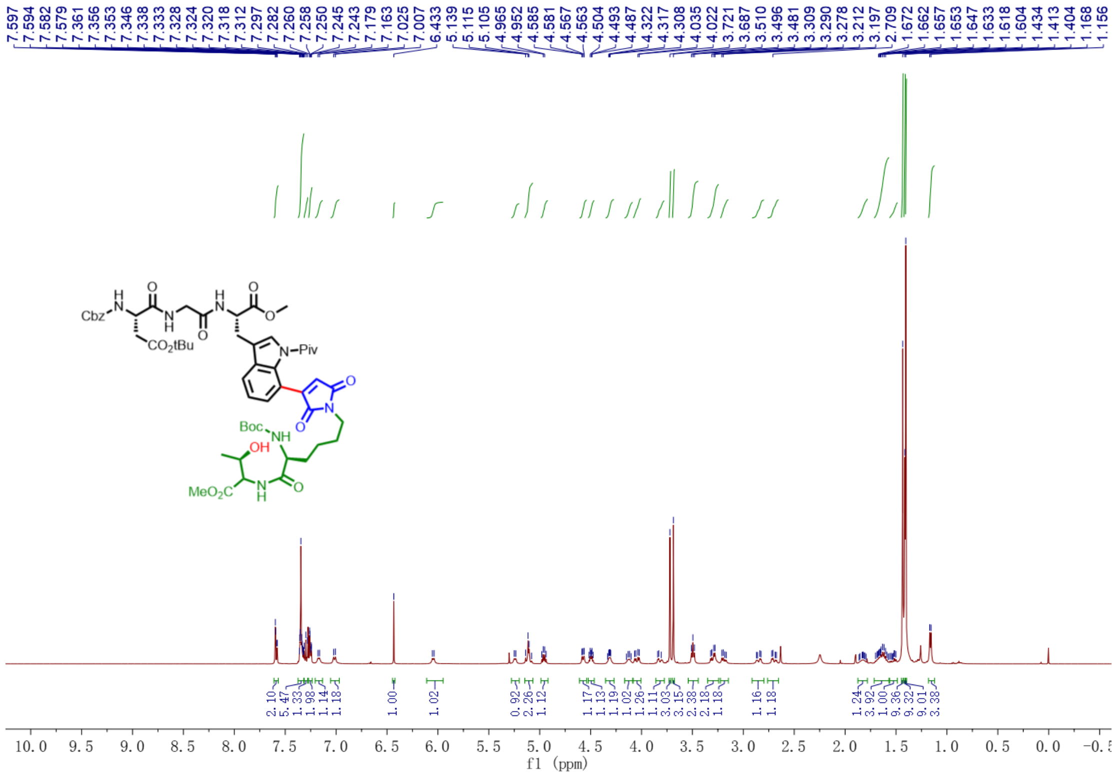

^{1}H NMR (400 MHz, $CDCl_3$) spectrum of **7f**

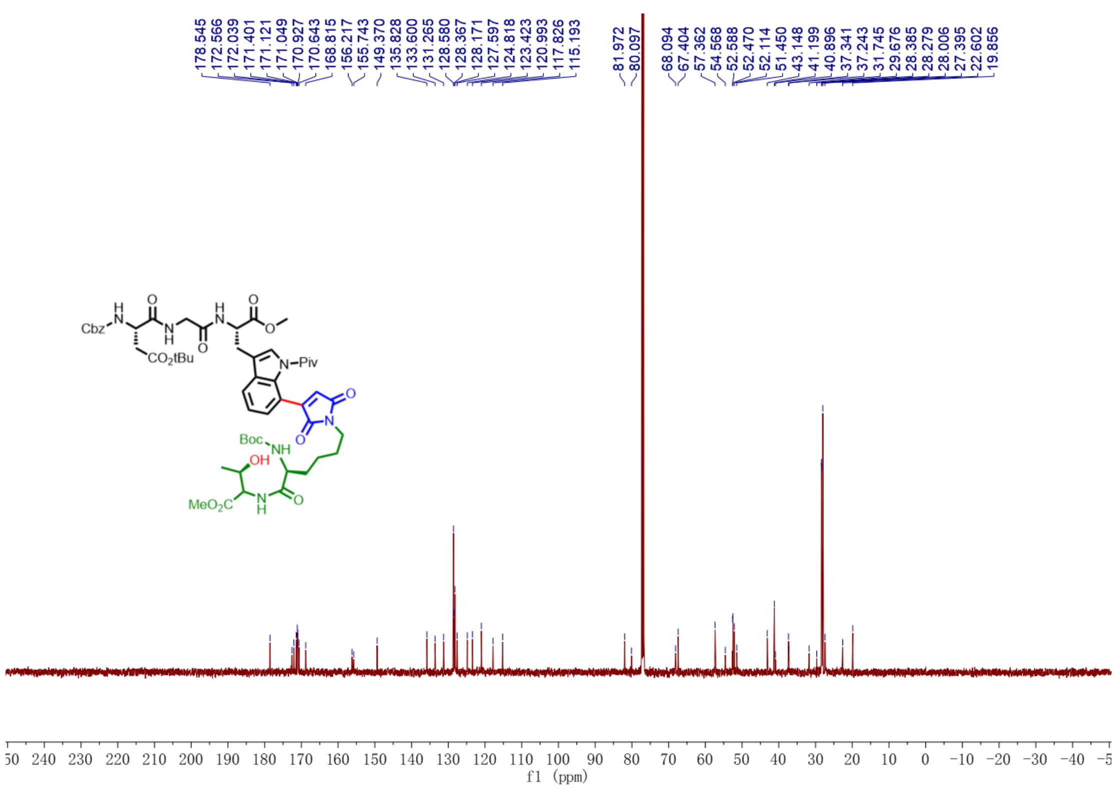

^{1}C NMR (101 MHz, $CDCl_3$) spectrum of **7f**

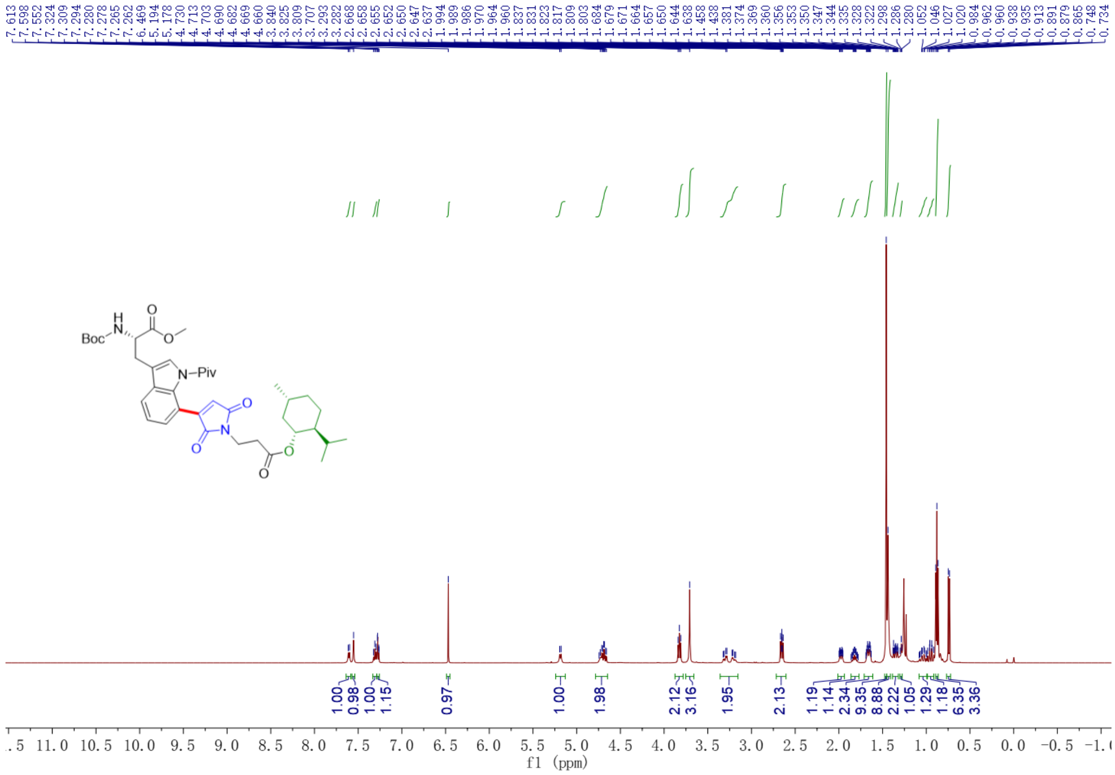

^{1}H NMR (500 MHz, $CDCl_3$) spectrum of **7g**

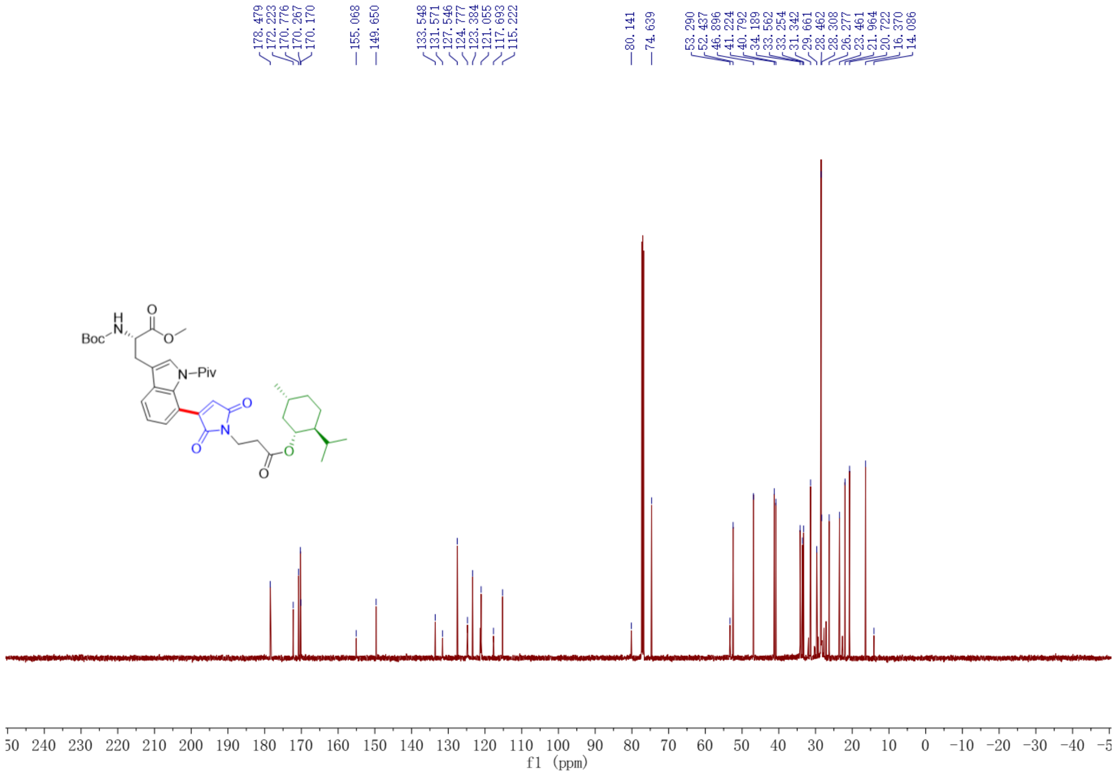

[1]C NMR (126 MHz, $CDCl_3$) spectrum of **7g**

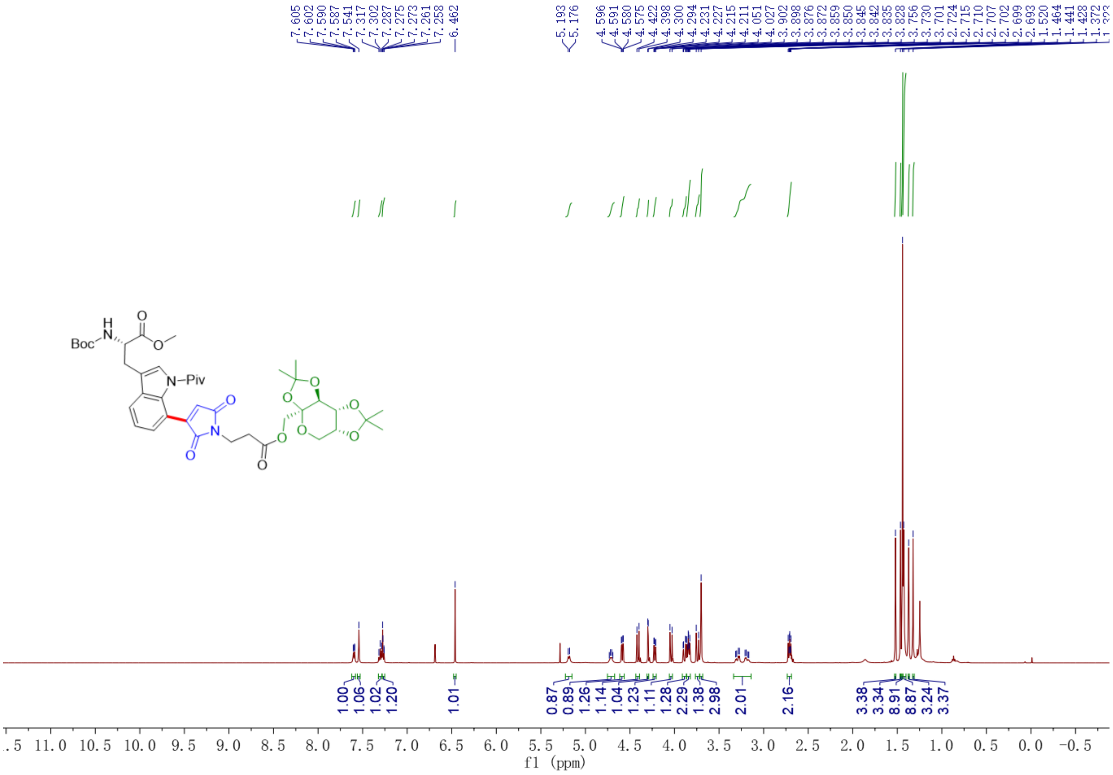

^{1}H NMR (500 MHz, CDCl$_3$) spectrum of **7h**

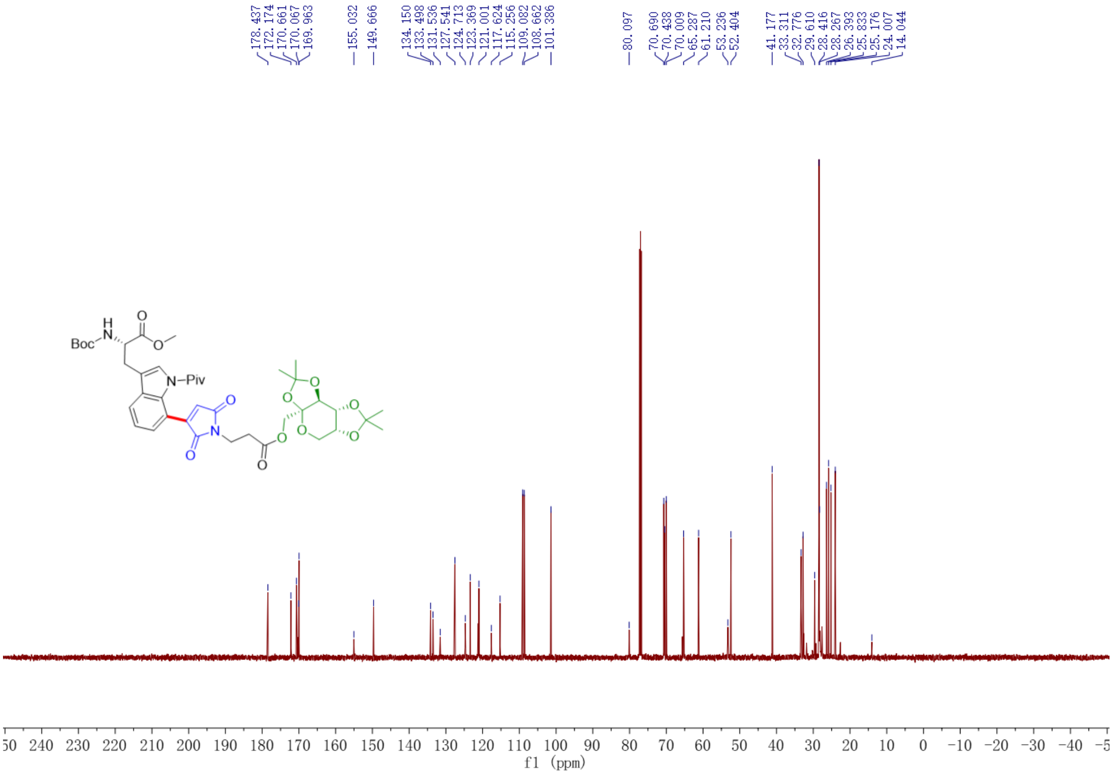

^{1}C NMR (126 MHz, CDCl$_3$) spectrum of **7h**

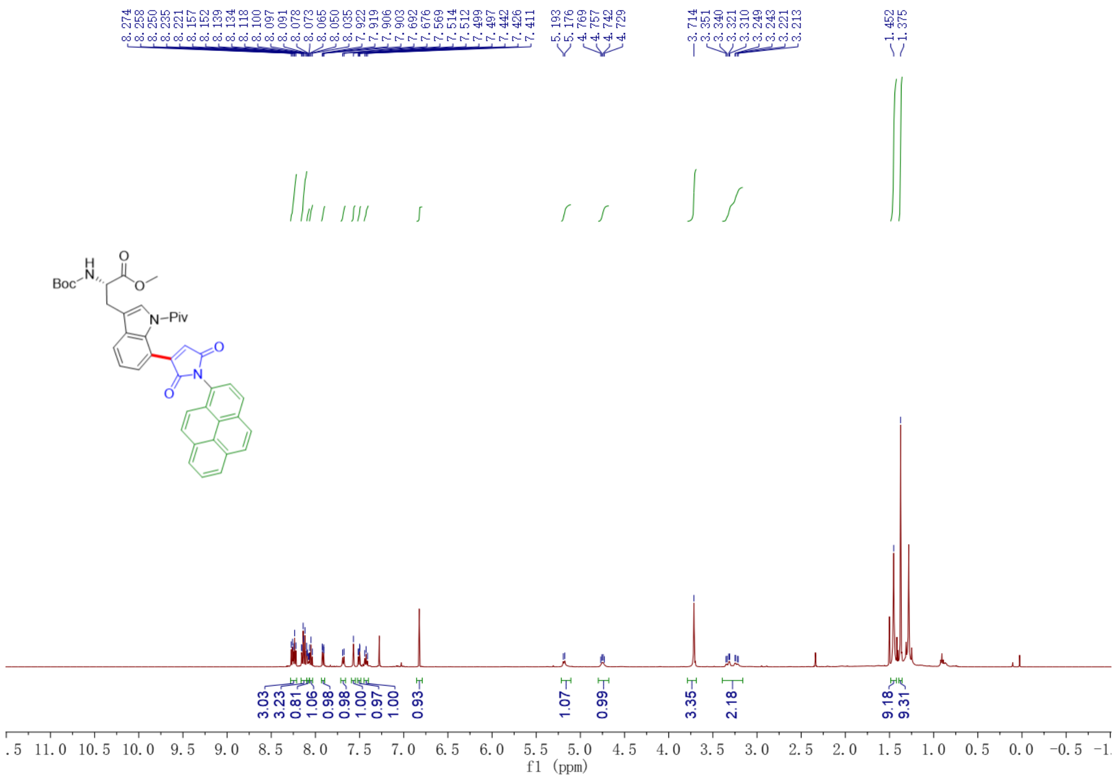

^{1}H NMR (500 MHz, $CDCl_3$) spectrum of **7i**

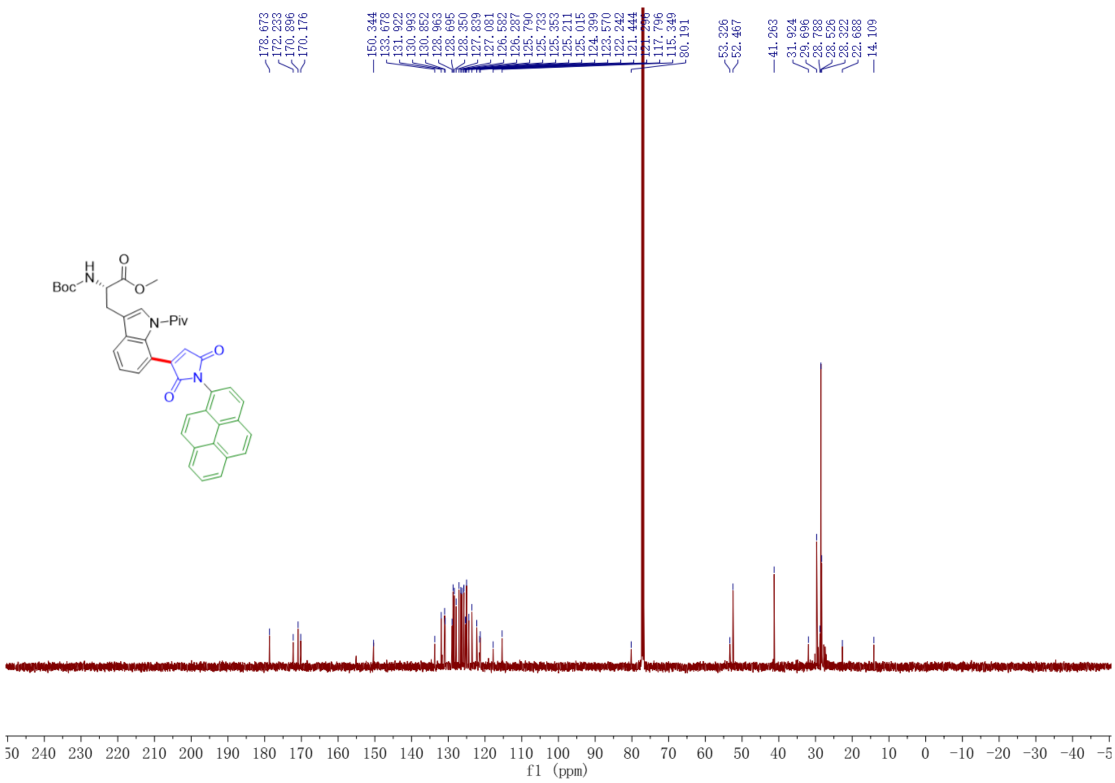

^{1}C NMR (126 MHz, $CDCl_3$) spectrum of **7i**

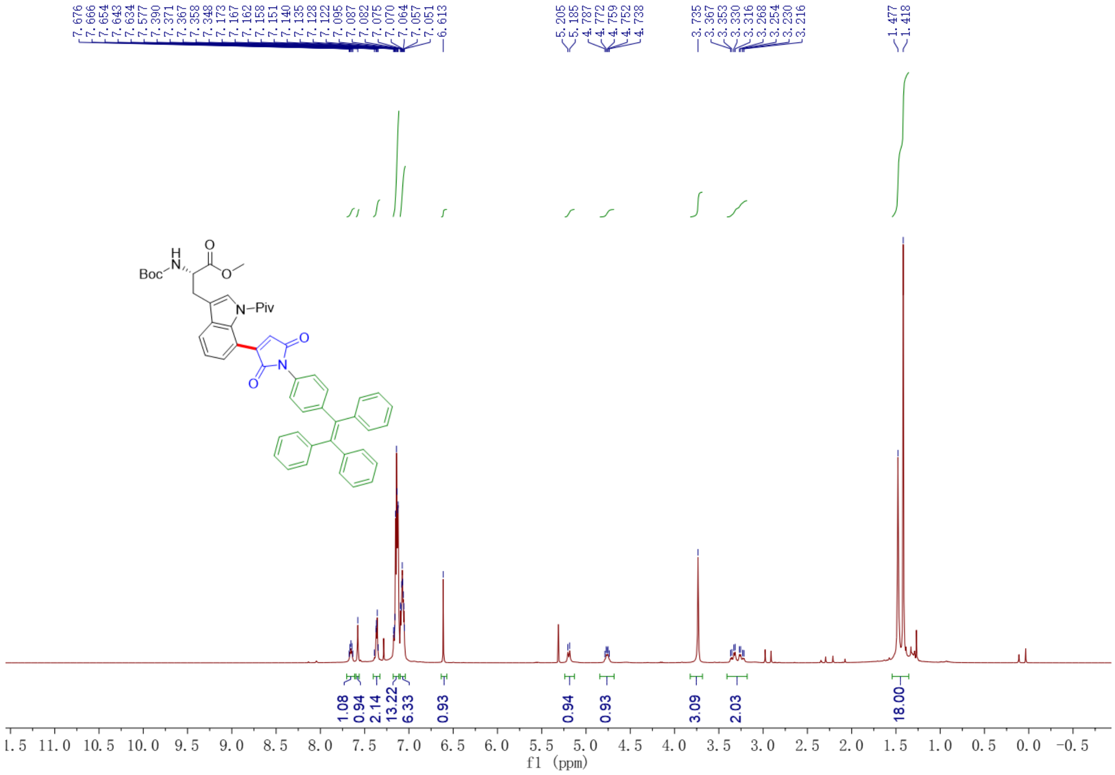

^{1}H NMR (400 MHz, $CDCl_3$) spectrum of **7j**

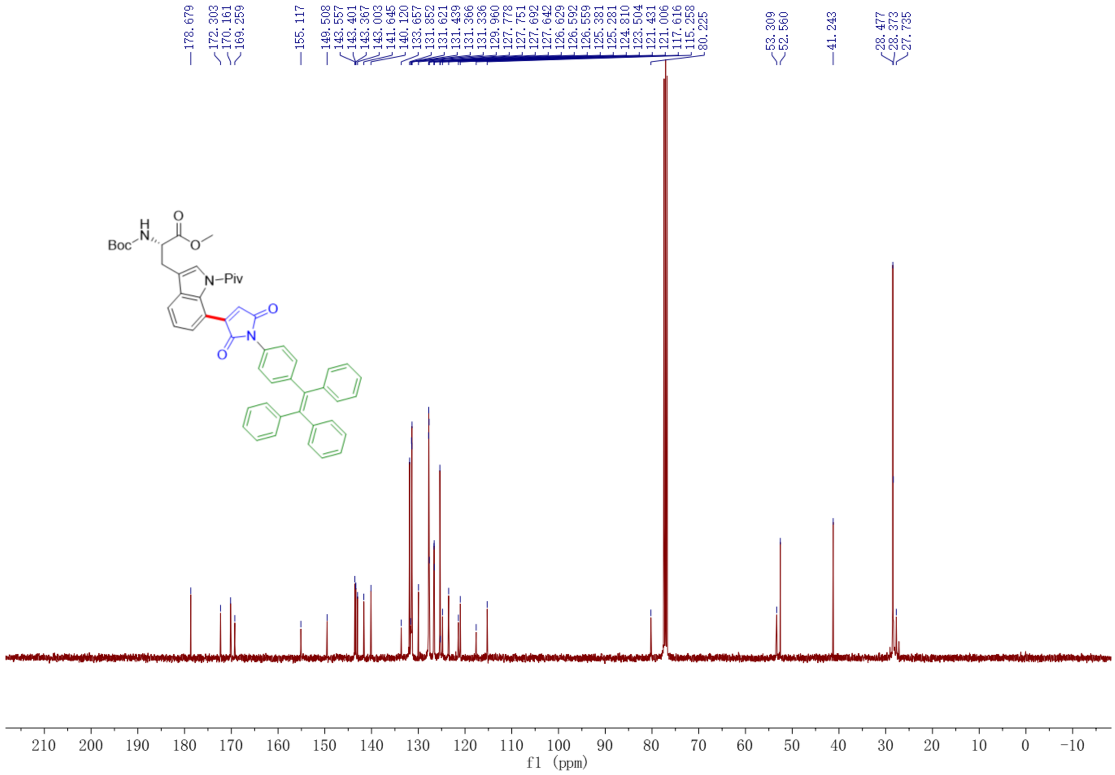

^{1}C NMR (101 MHz, $CDCl_3$) spectrum of **7j**

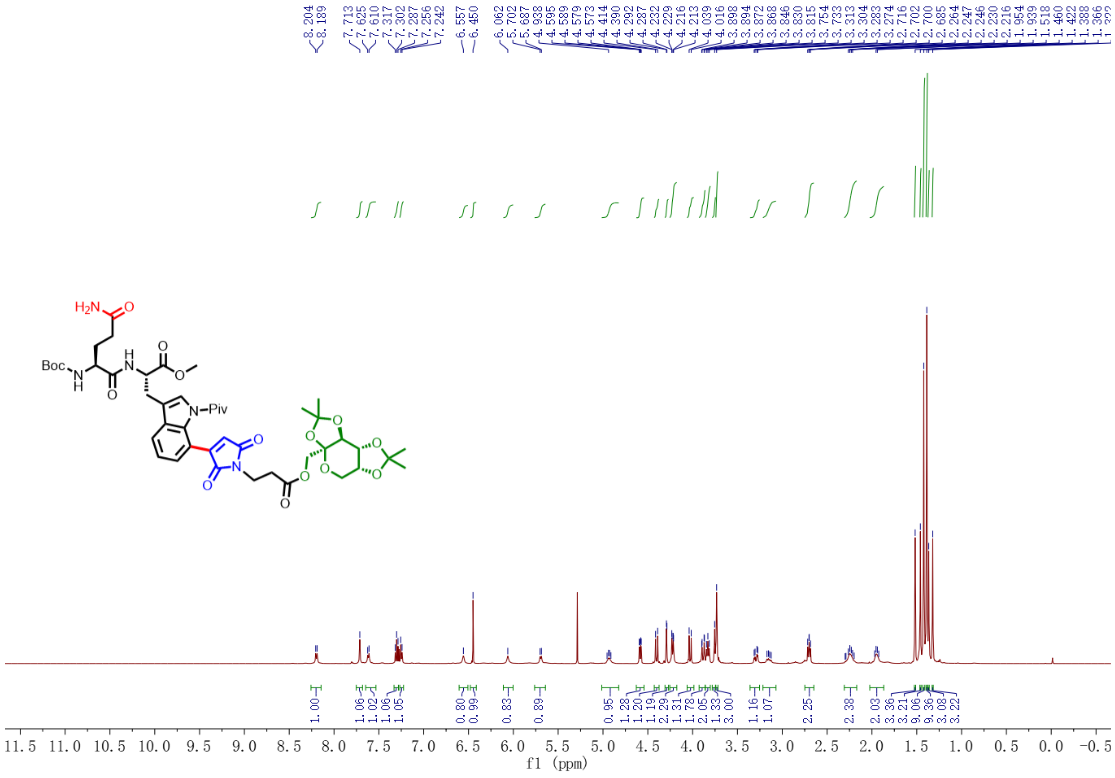

^{1}H NMR (500 MHz, $CDCl_3$) spectrum of **7k**

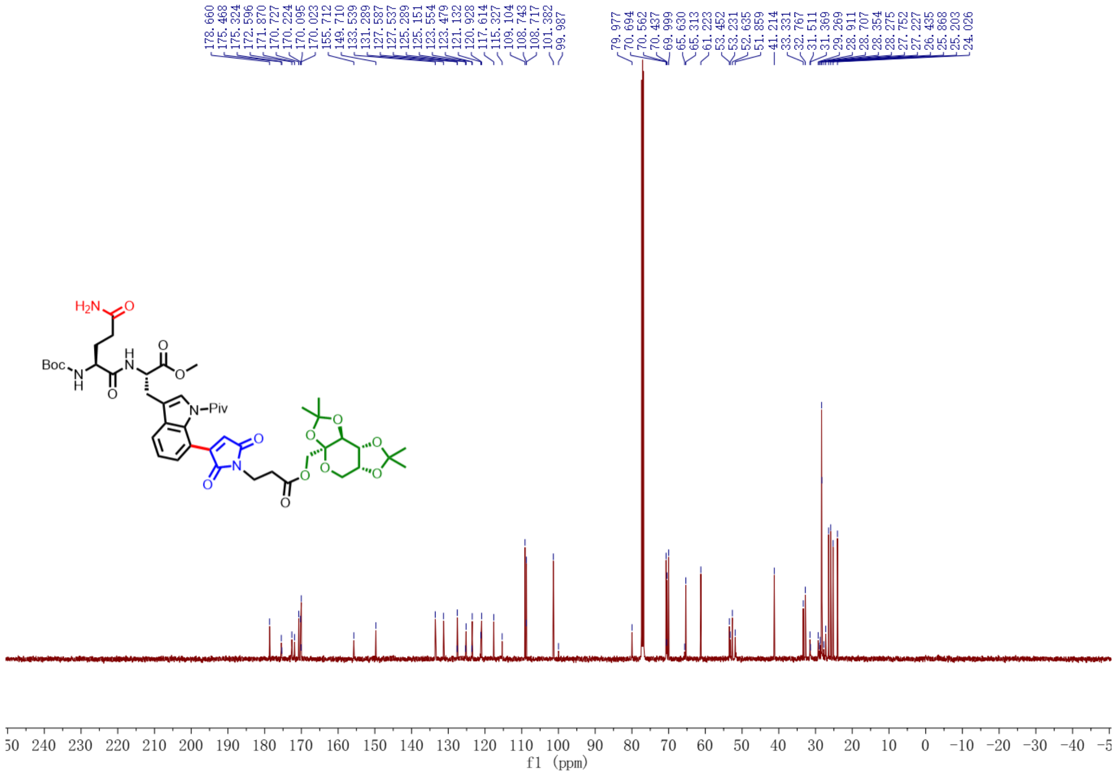

[1]C NMR (126 MHz, $CDCl_3$) spectrum of **7k**

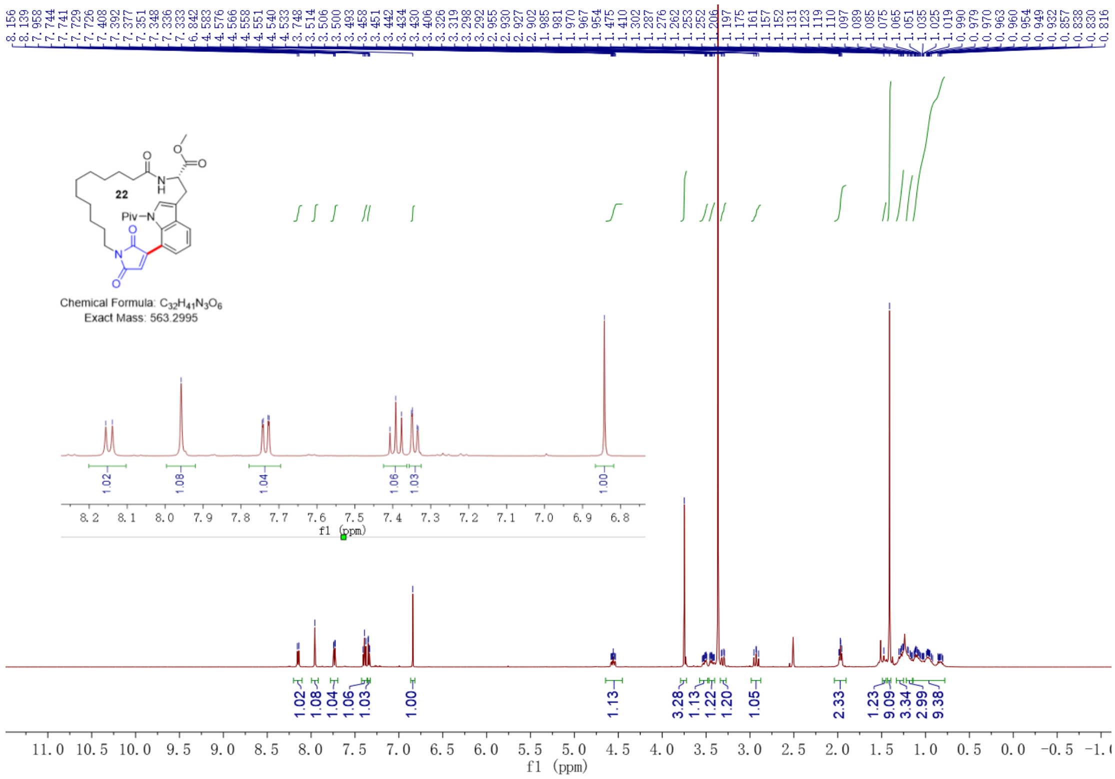

^{1}H NMR (500 MHz, DMSO) spectrum of **9a**

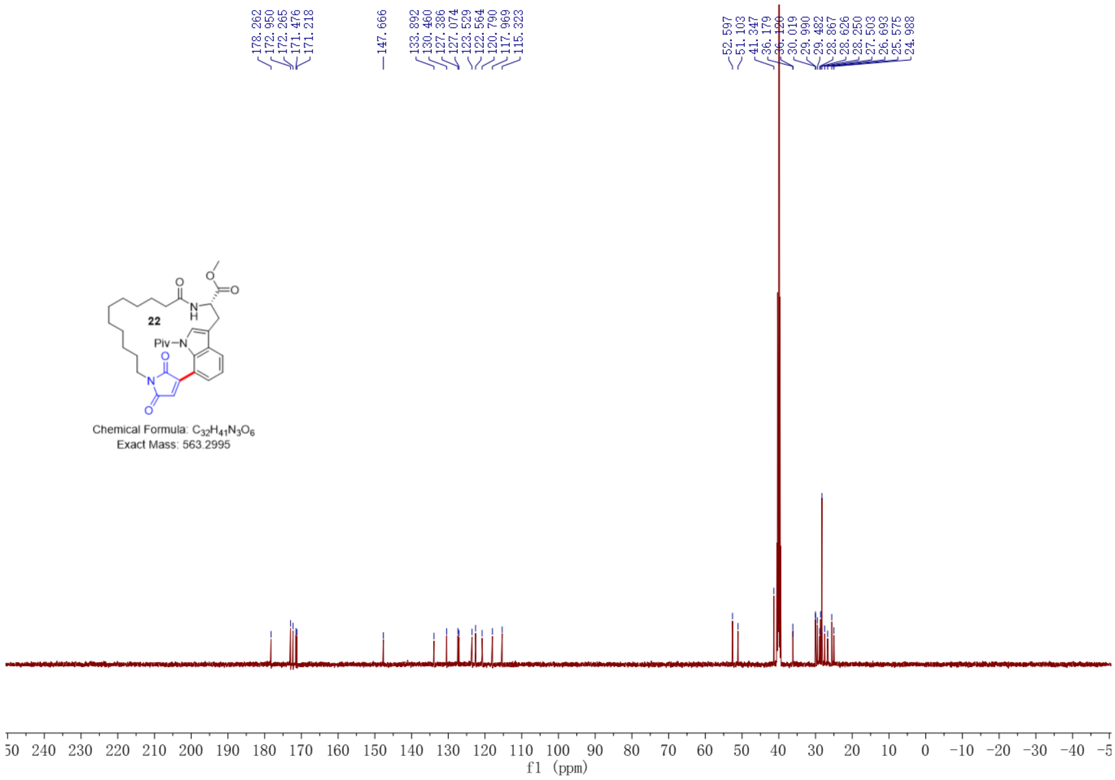

^{1}C NMR (126 MHz, DMSO) spectrum of **9a**

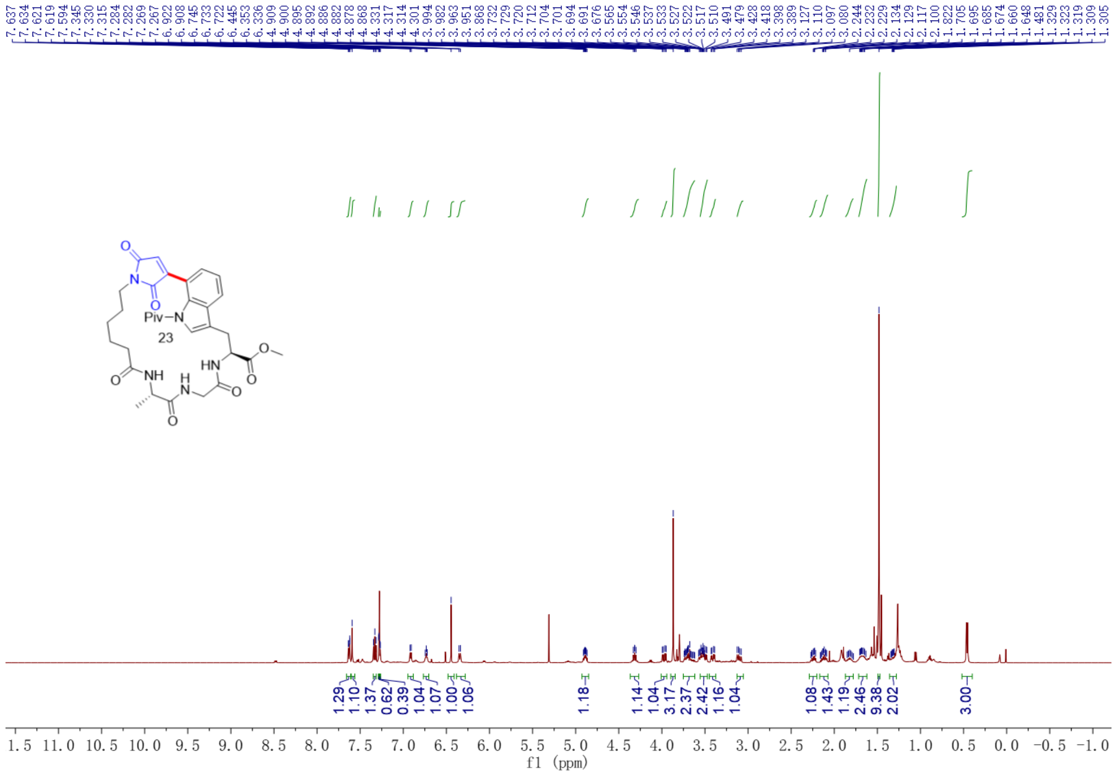

^{1}H NMR (500 MHz, $CDCl_3$) spectrum of **9b**

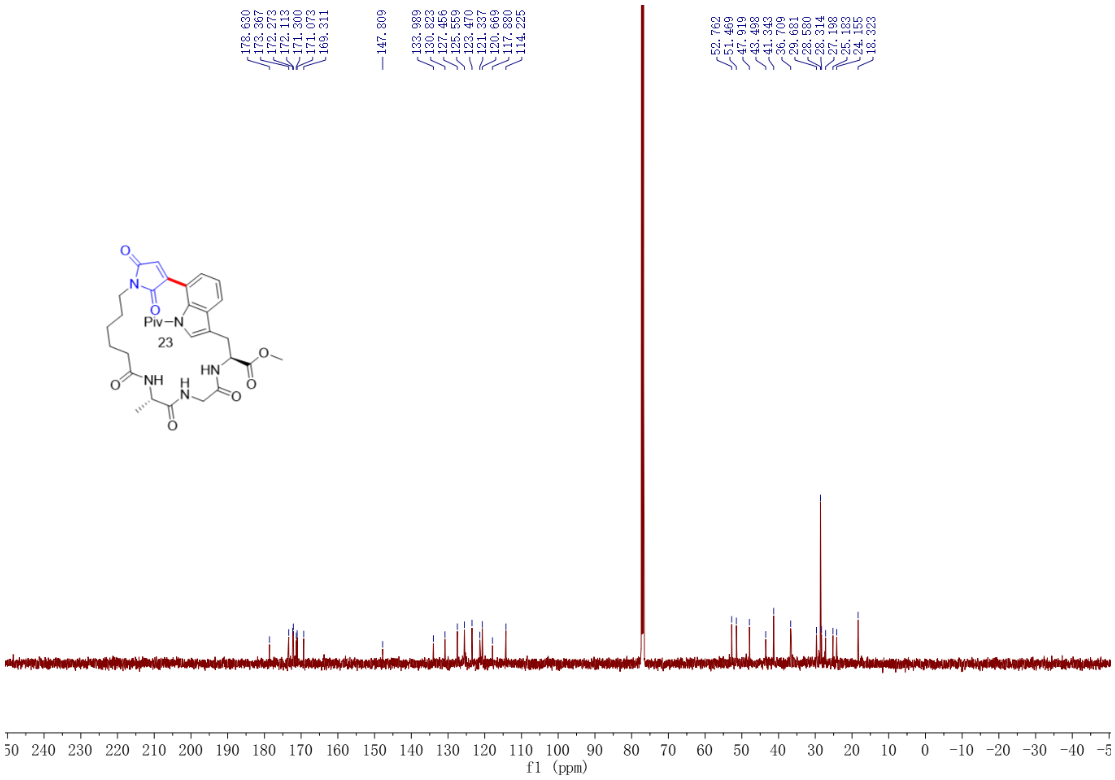

^{1}C NMR (126 MHz, $CDCl_3$) spectrum of **9b**

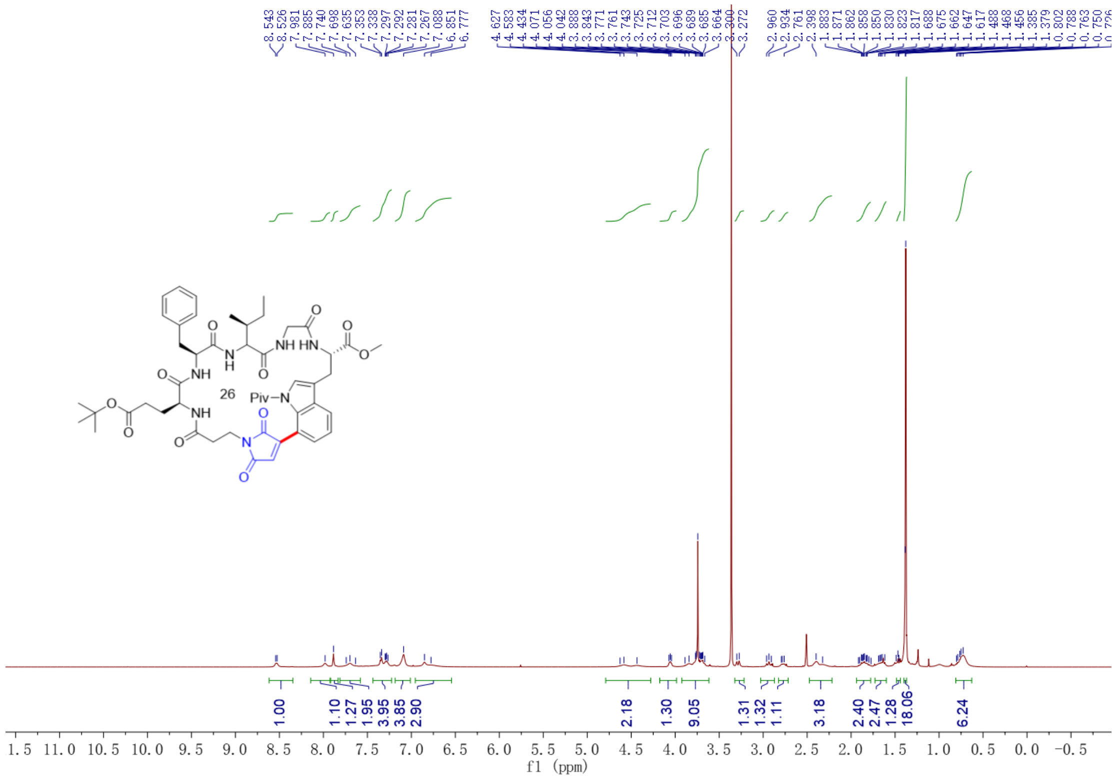

^{1}H NMR (500 MHz, DMSO) spectrum of **9c**

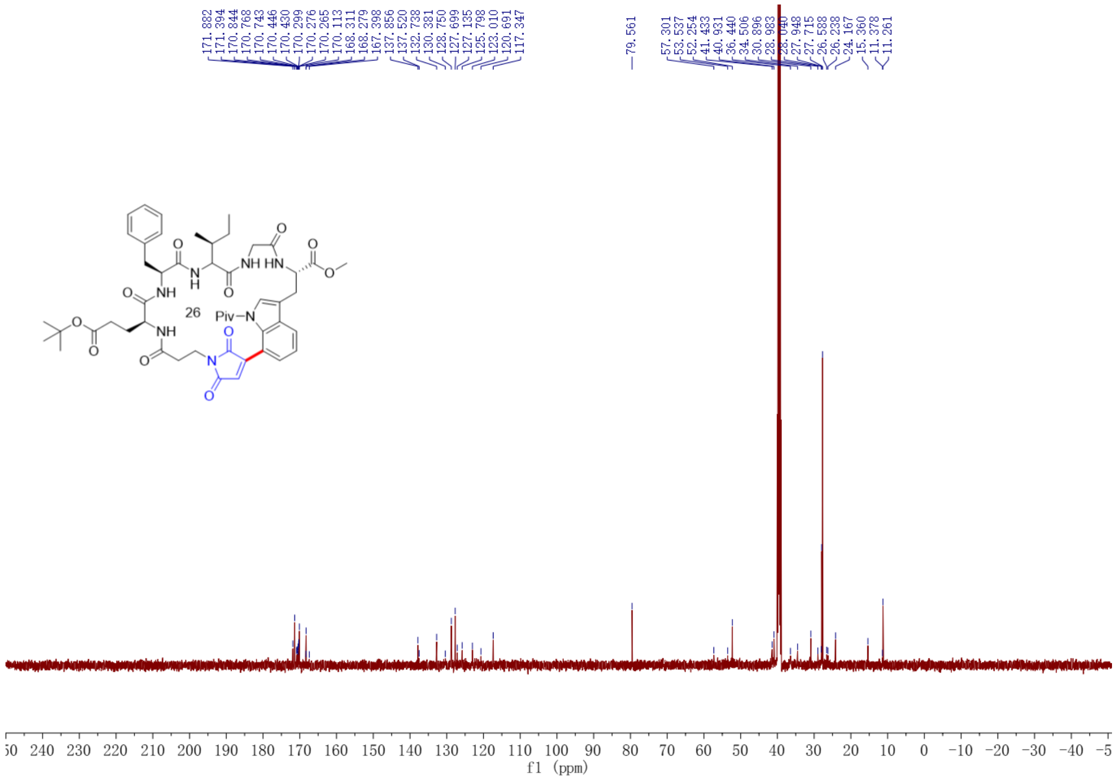

^{1}C NMR (126 MHz, DMSO) spectrum of **9c**

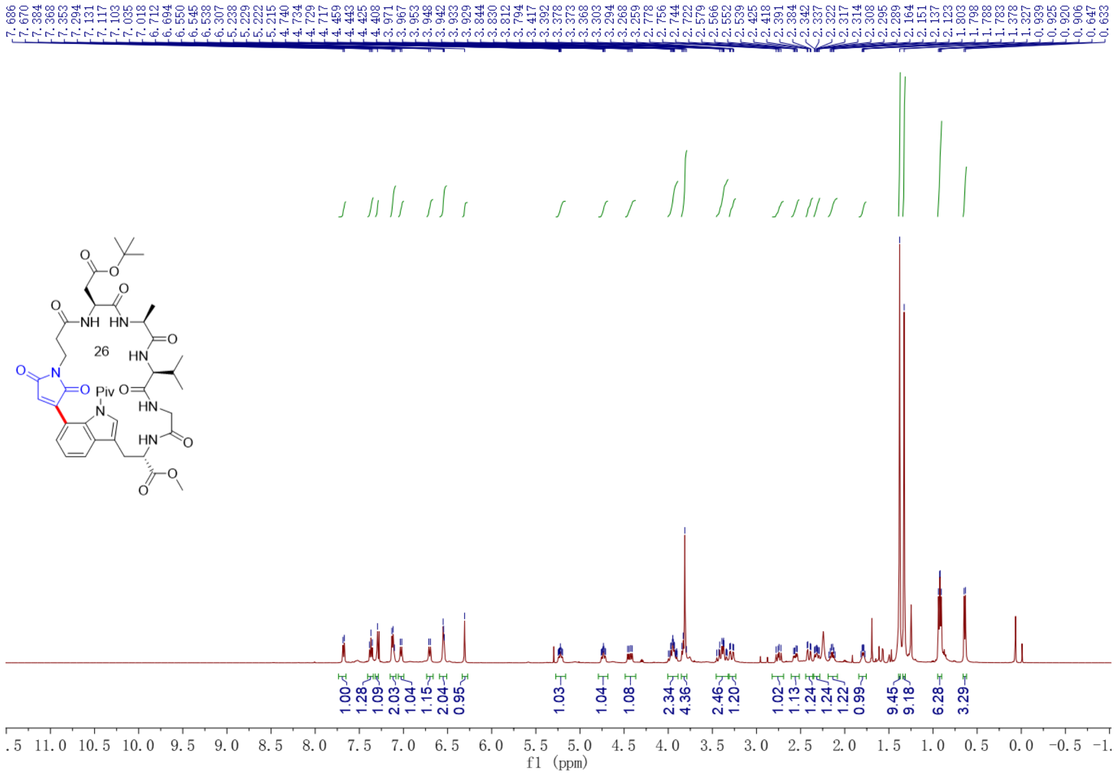

^{1}H NMR (500 MHz, $CDCl_3$) spectrum of **9d**

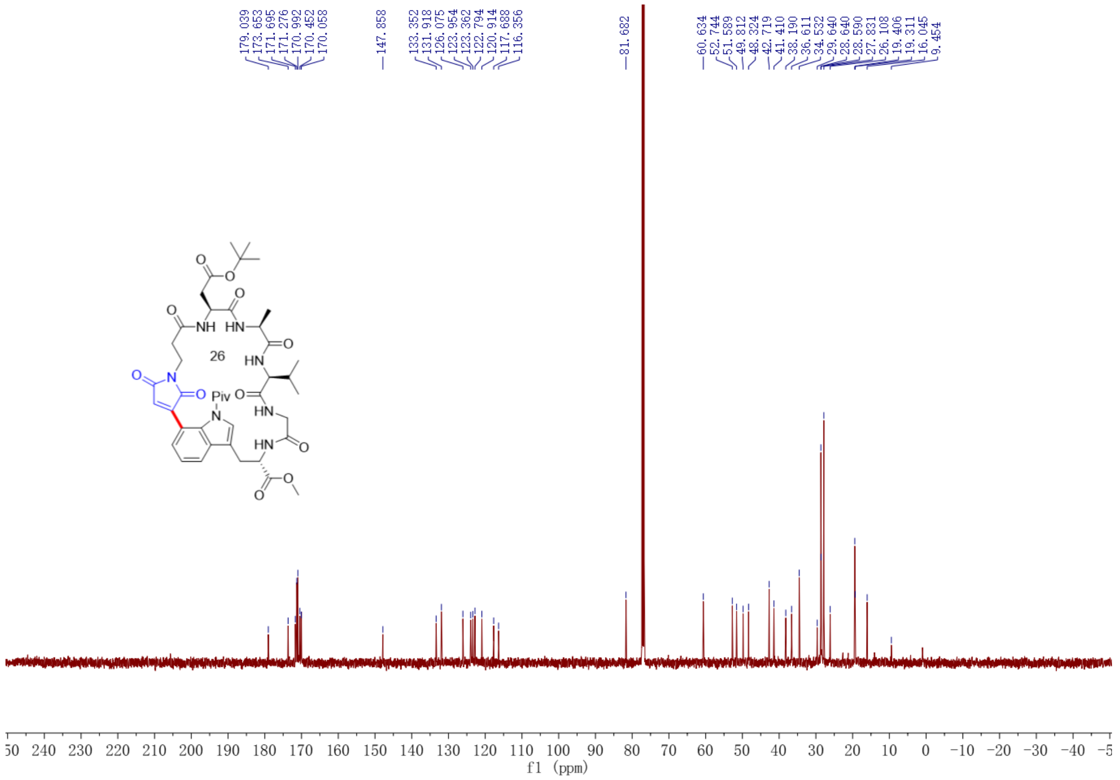

^{1}C NMR (126 MHz, $CDCl_3$) spectrum of **9d**

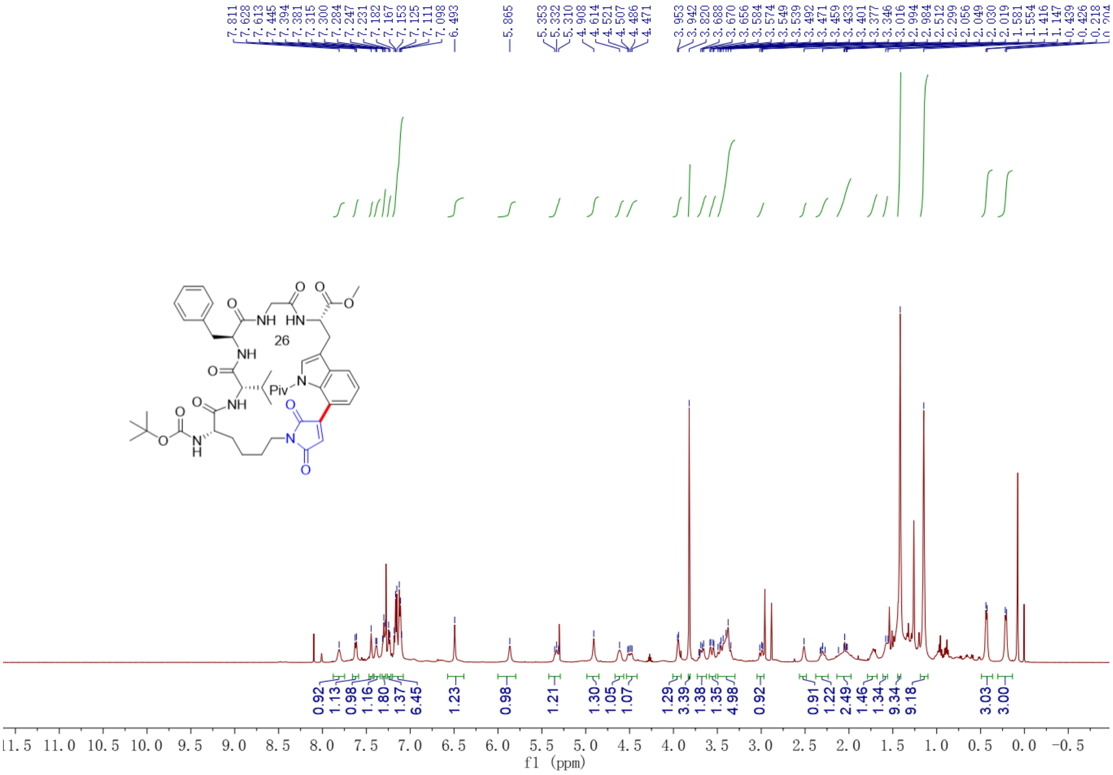

^{1}H NMR (500 MHz, CDCl$_3$) spectrum of **9e**

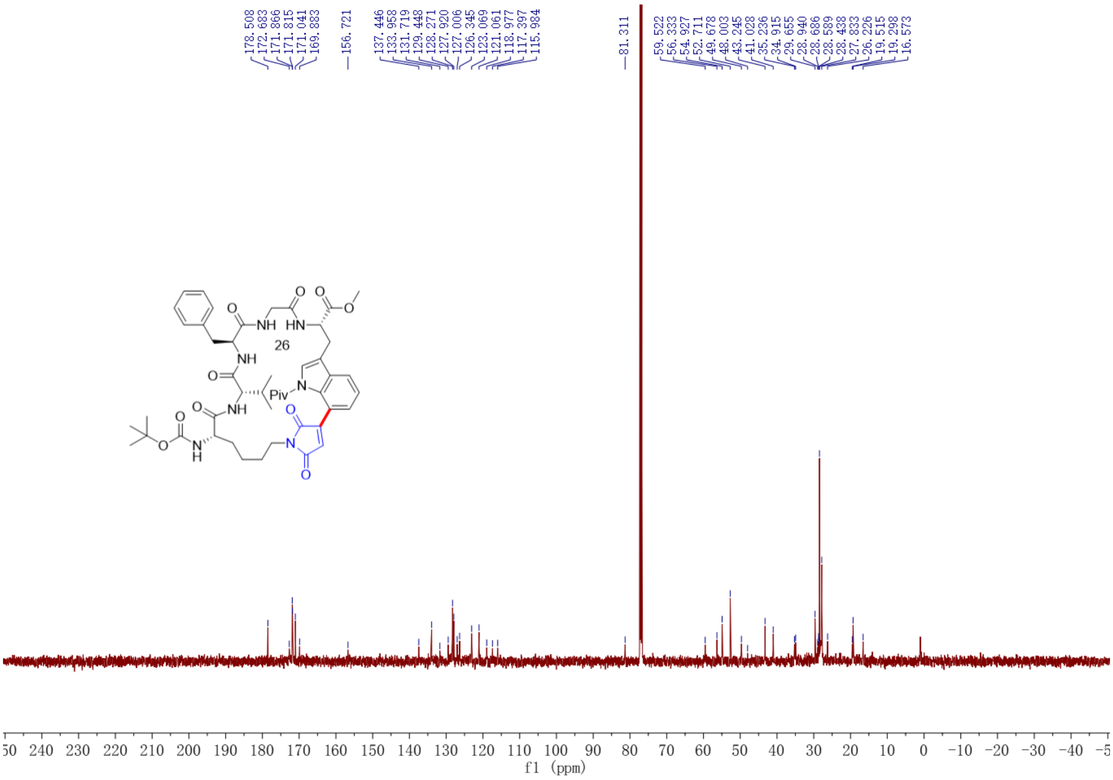

^{1}C NMR (126 MHz, CDCl$_3$) spectrum of **9e**

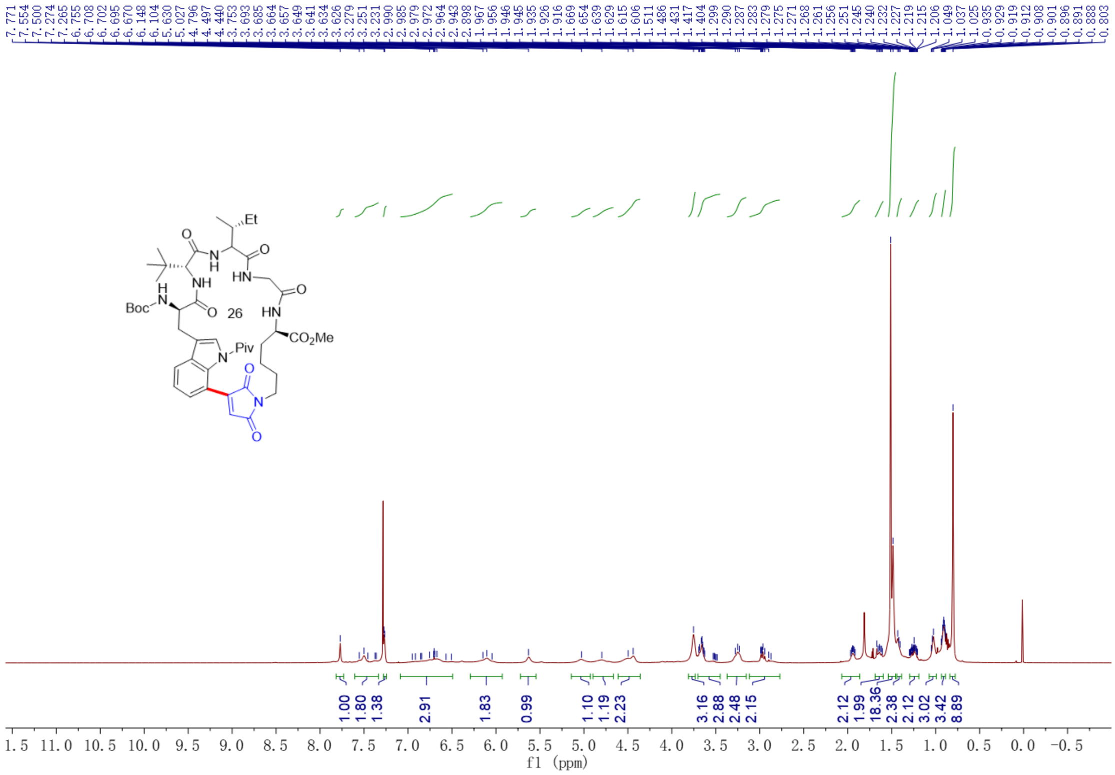

^{1}H NMR (600 MHz, $CDCl_3$) spectrum of **9f**

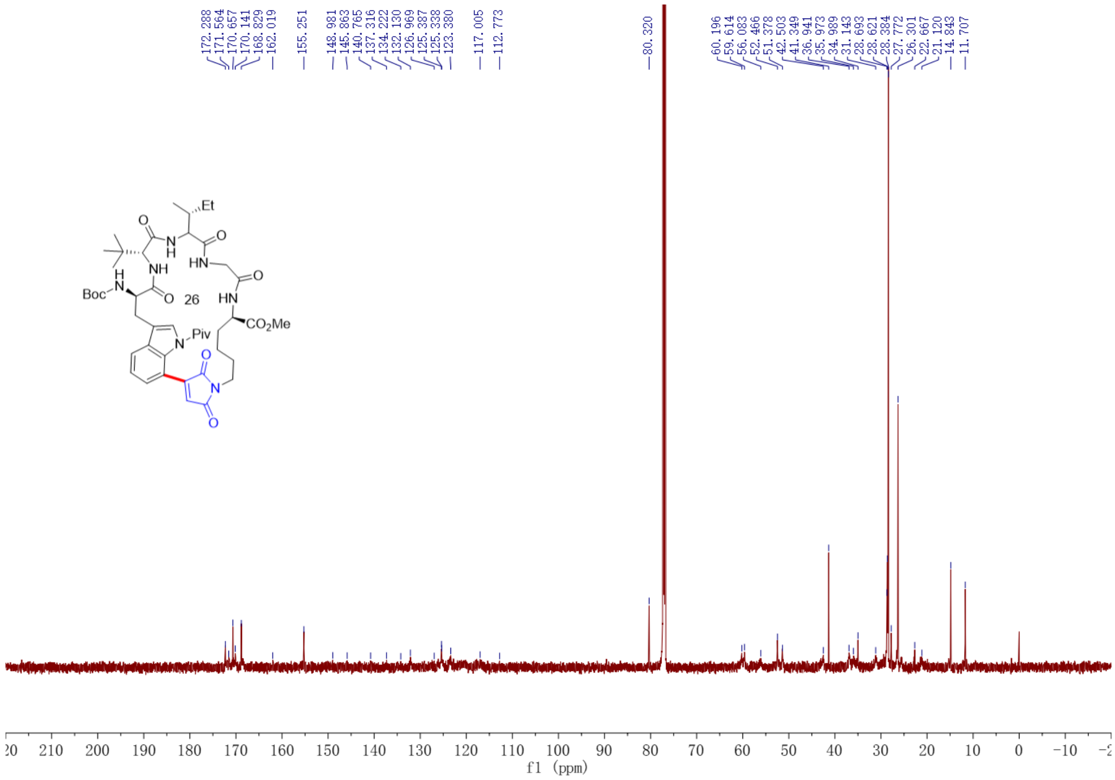

^{1}C NMR (151 MHz, $CDCl_3$) spectrum of **9f**

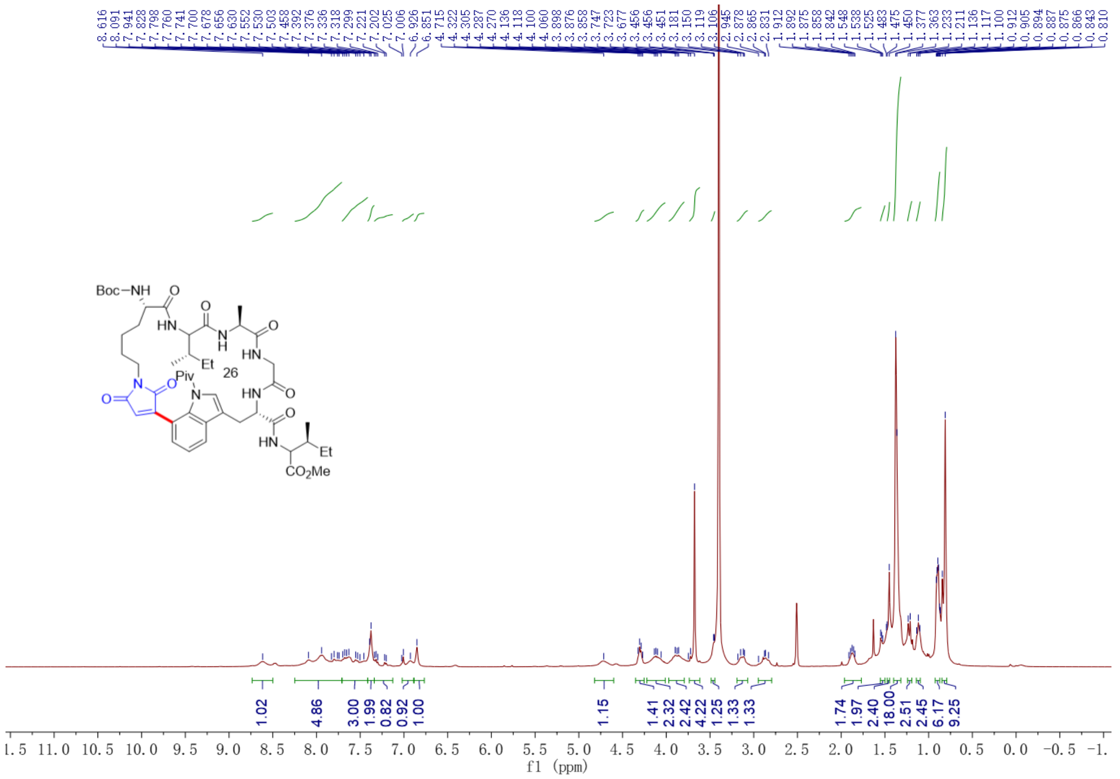

^1H NMR (400 MHz, DMSO) spectrum of **9g**

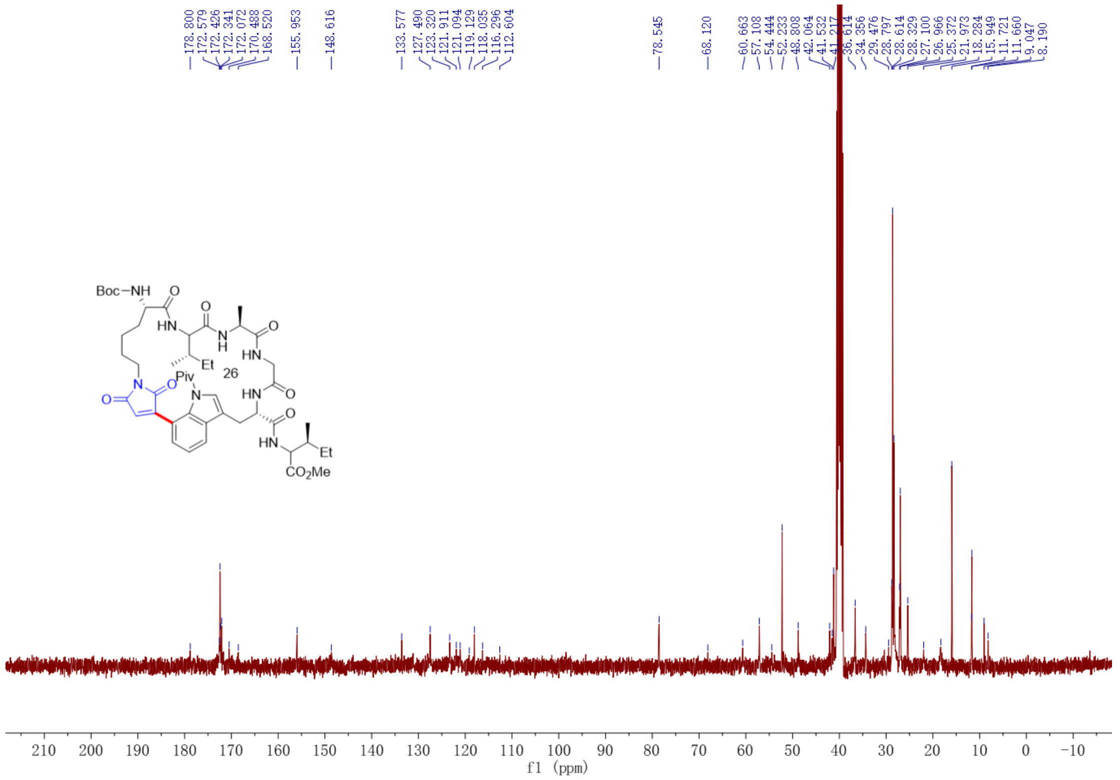

^1C NMR (101 MHz, DMSO) spectrum of **9g**

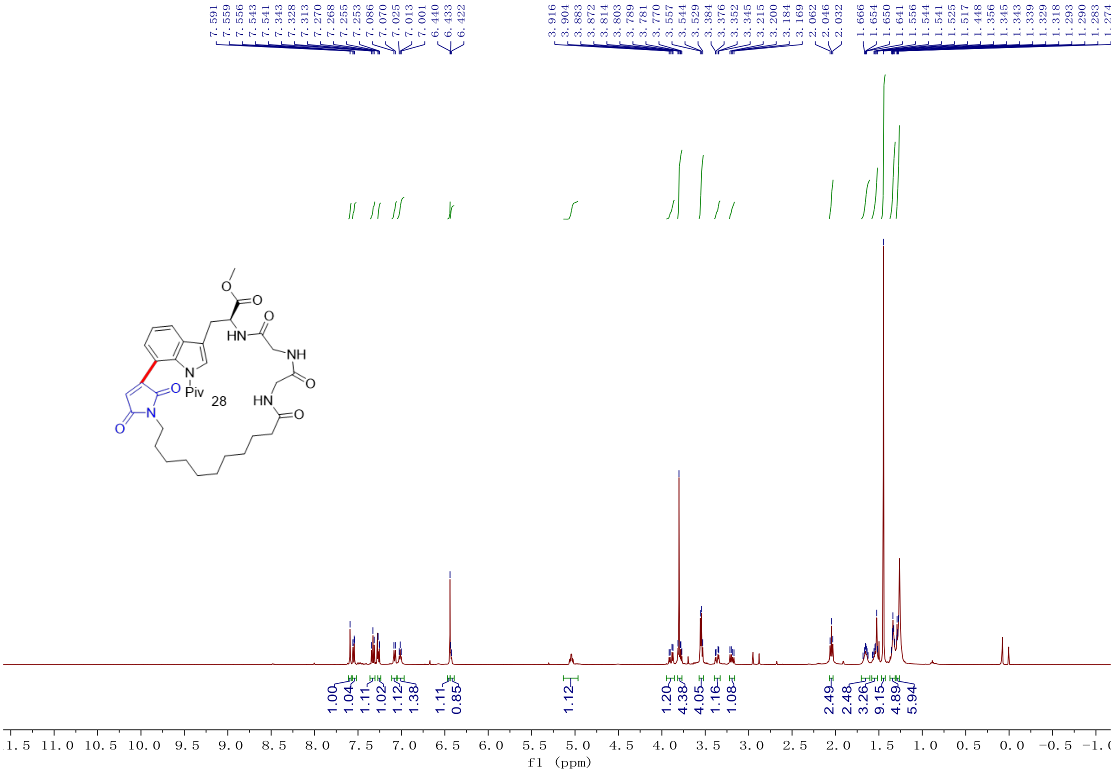

^{1}H NMR (500 MHz, CDCl$_3$) spectrum of **9h**

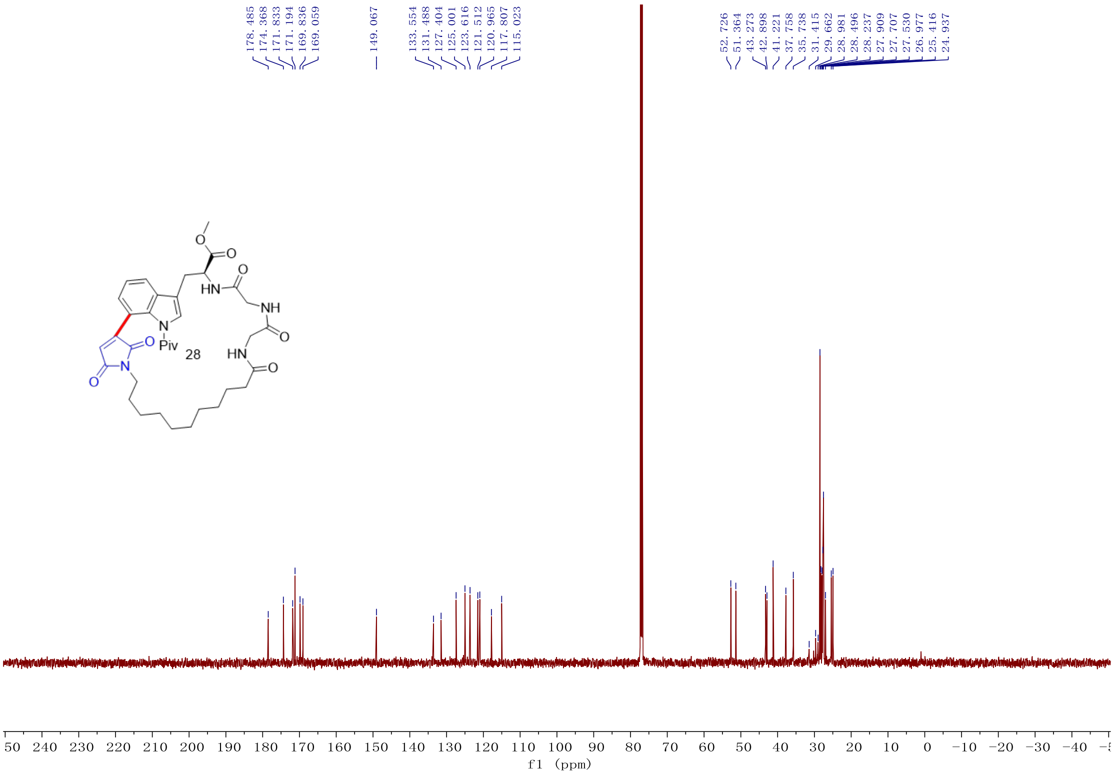

^{1}C NMR (126 MHz, CDCl$_3$) spectrum of **9h**

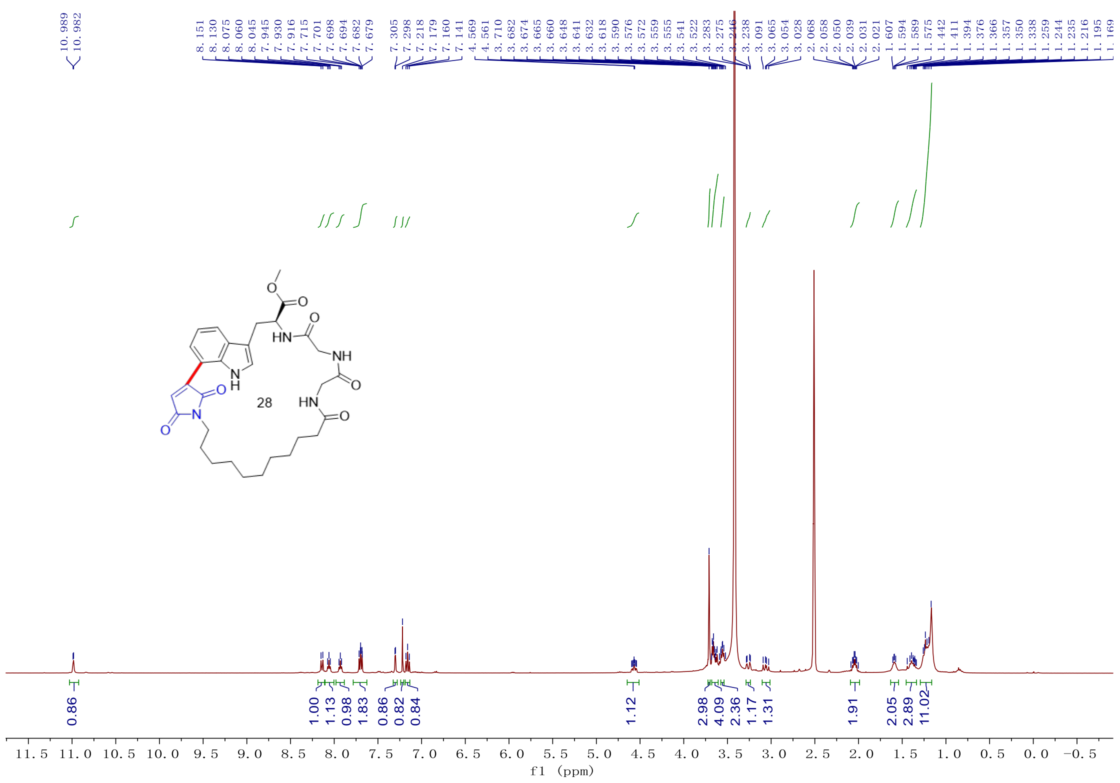

^{1}H NMR (400 MHz, DMSO) spectrum of **9ha**

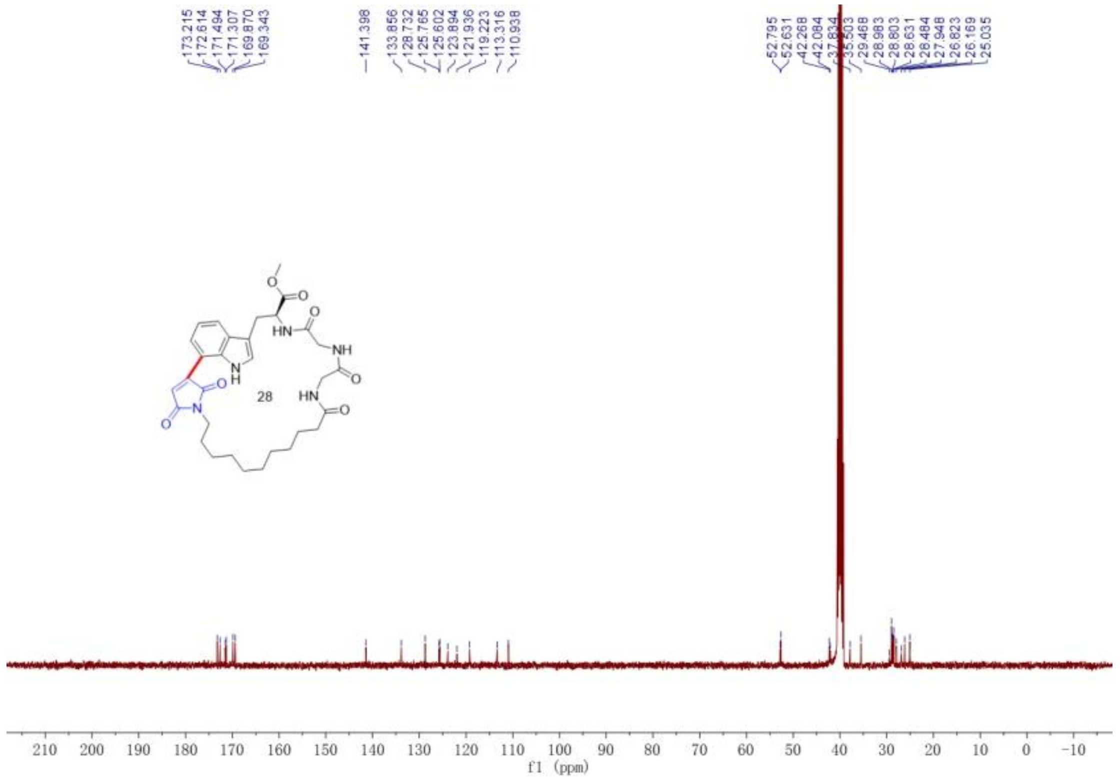

^{1}C NMR (101 MHz, DMSO) spectrum of **9ha**

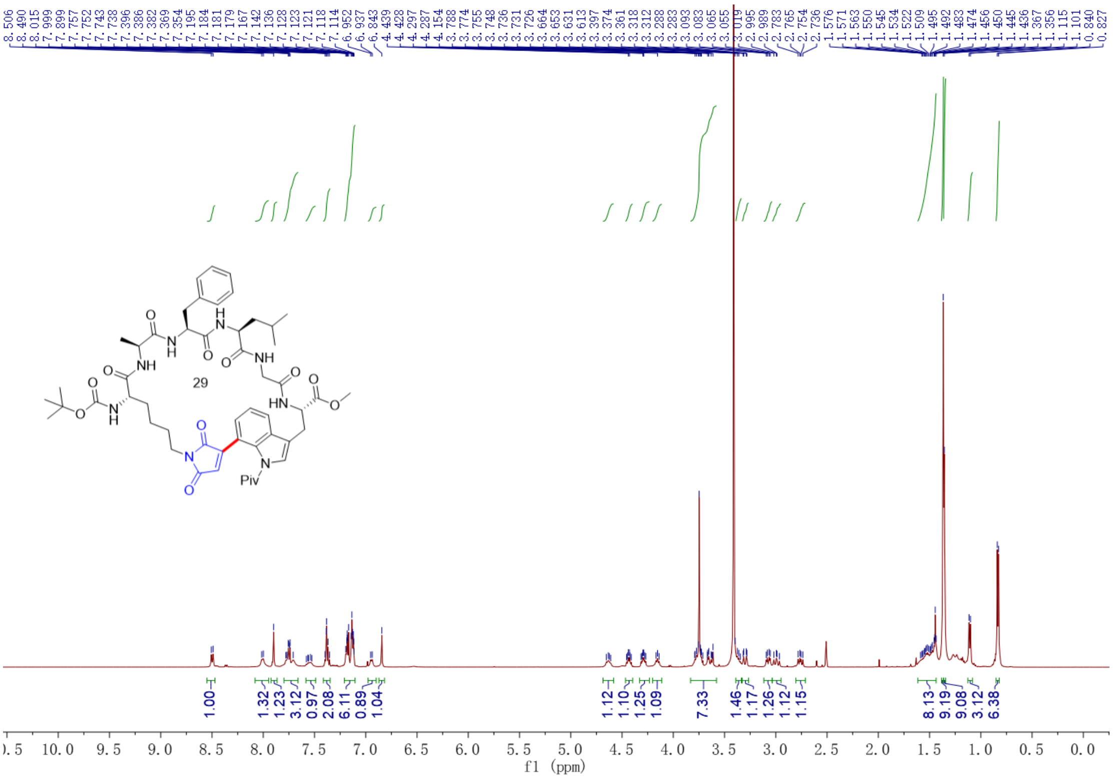

^{1}H NMR (500 MHz, DMSO) spectrum of **9i**

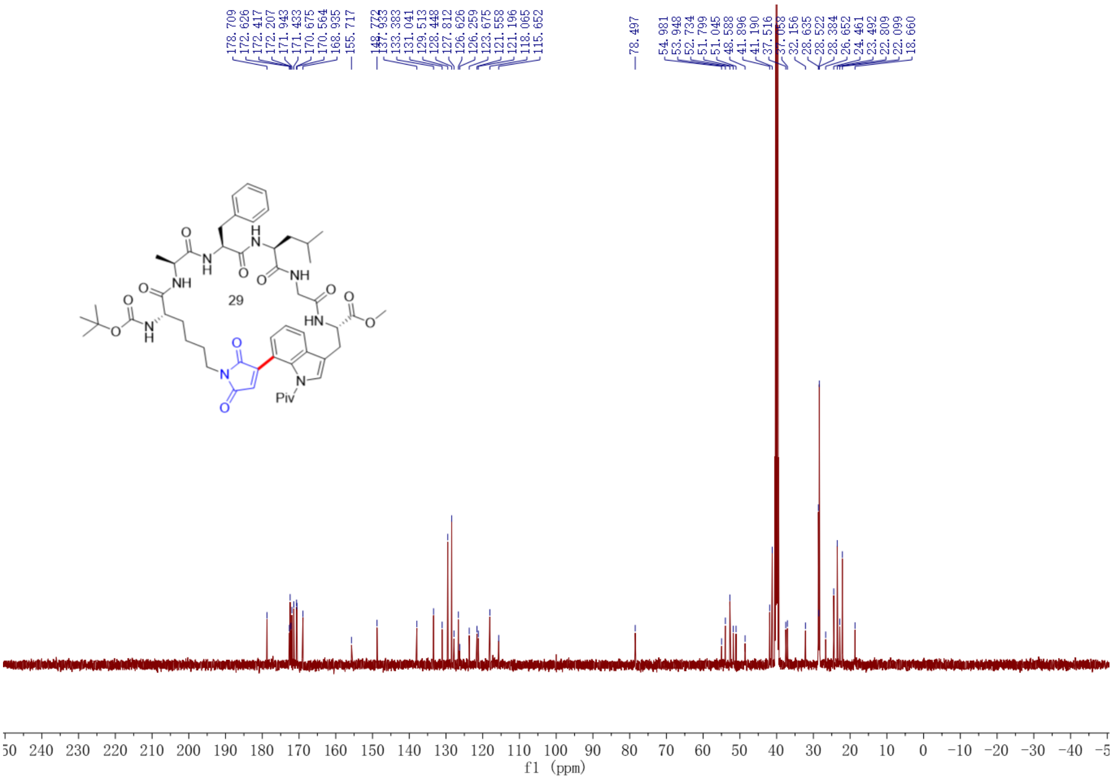

^{1}C NMR (126 MHz, DMSO) spectrum of **9i**

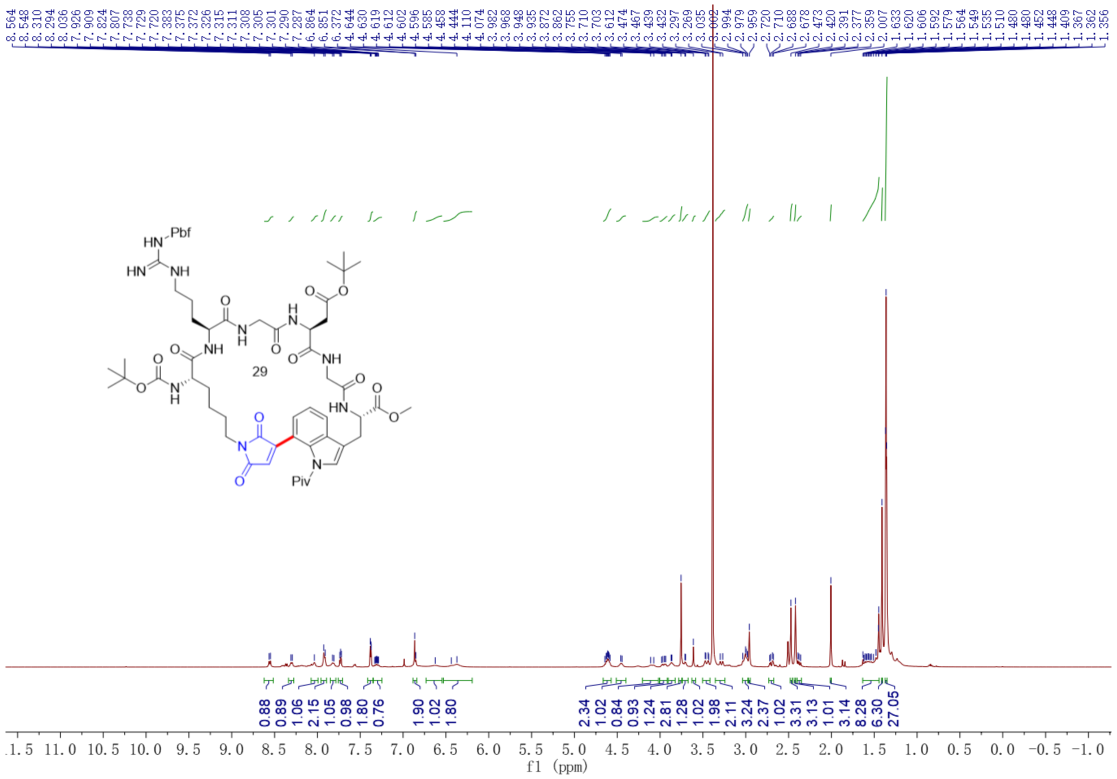

^{1}H NMR (500 MHz, DMSO) spectrum of **9j**

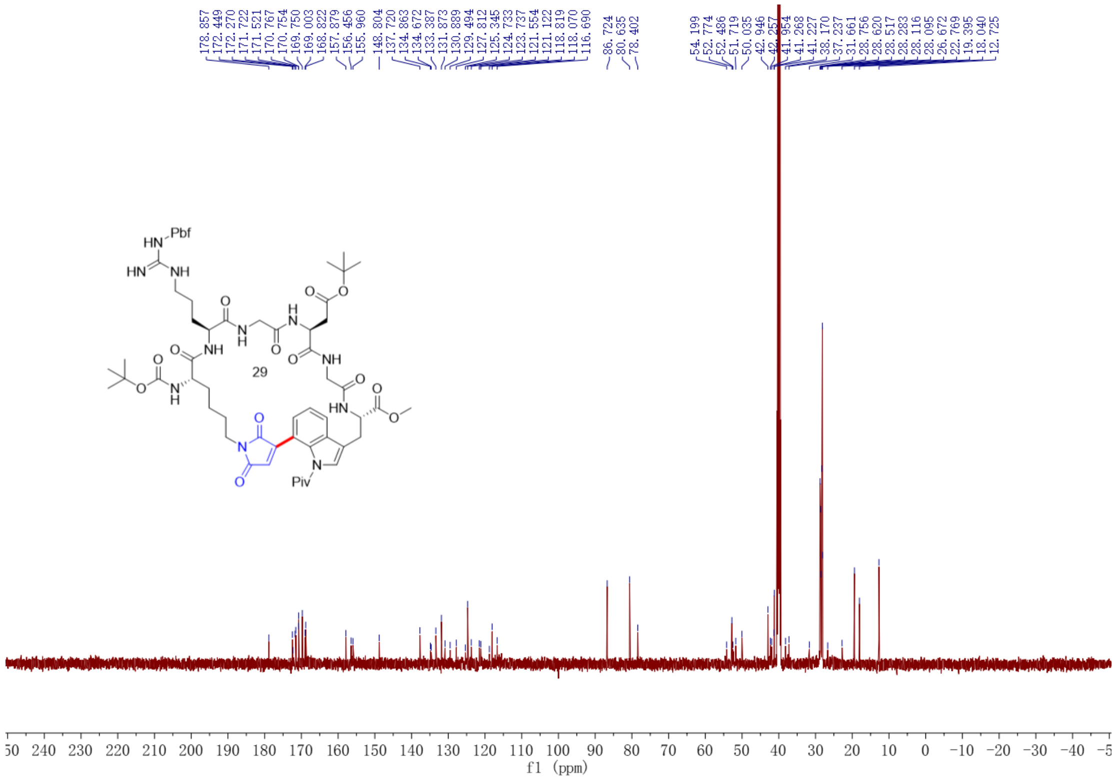

[1]C NMR (126 MHz, DMSO) spectrum of **9j**

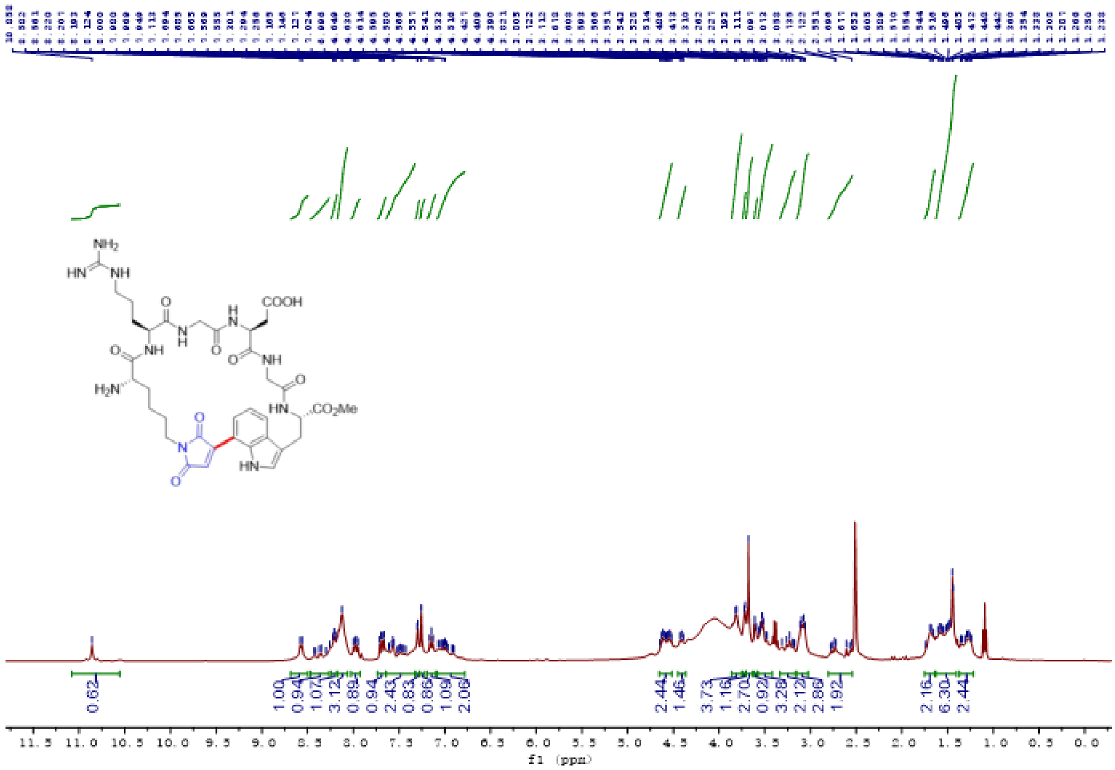

^{1}H NMR (400 MHz, DMSO) spectrum of **10a**

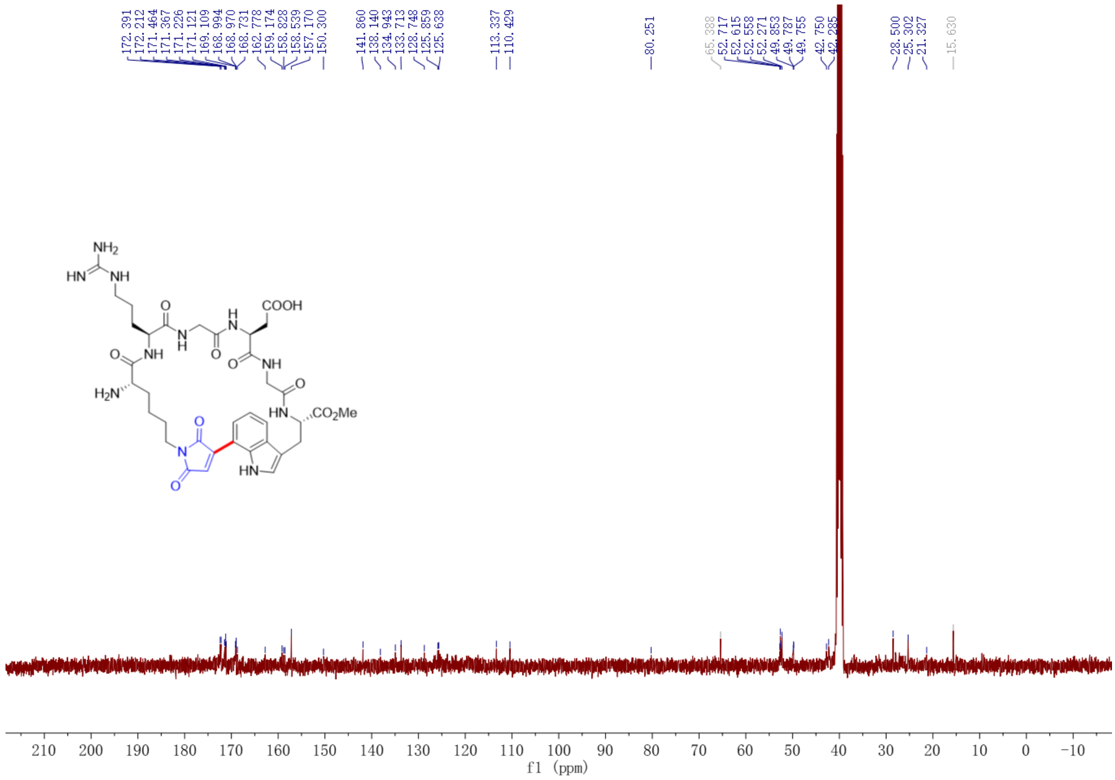

^{1}C NMR (101 MHz, DMSO) spectrum of **10a**

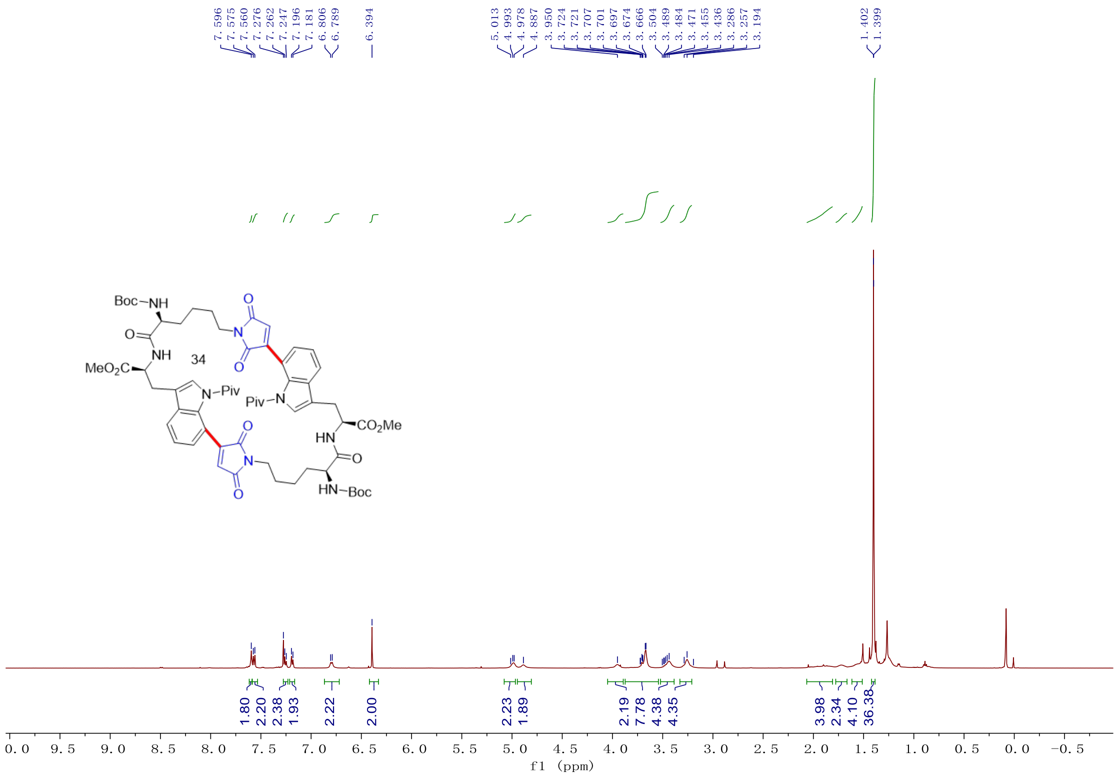

^{1}H NMR (500 MHz, $CDCl_3$) spectrum of **9k**

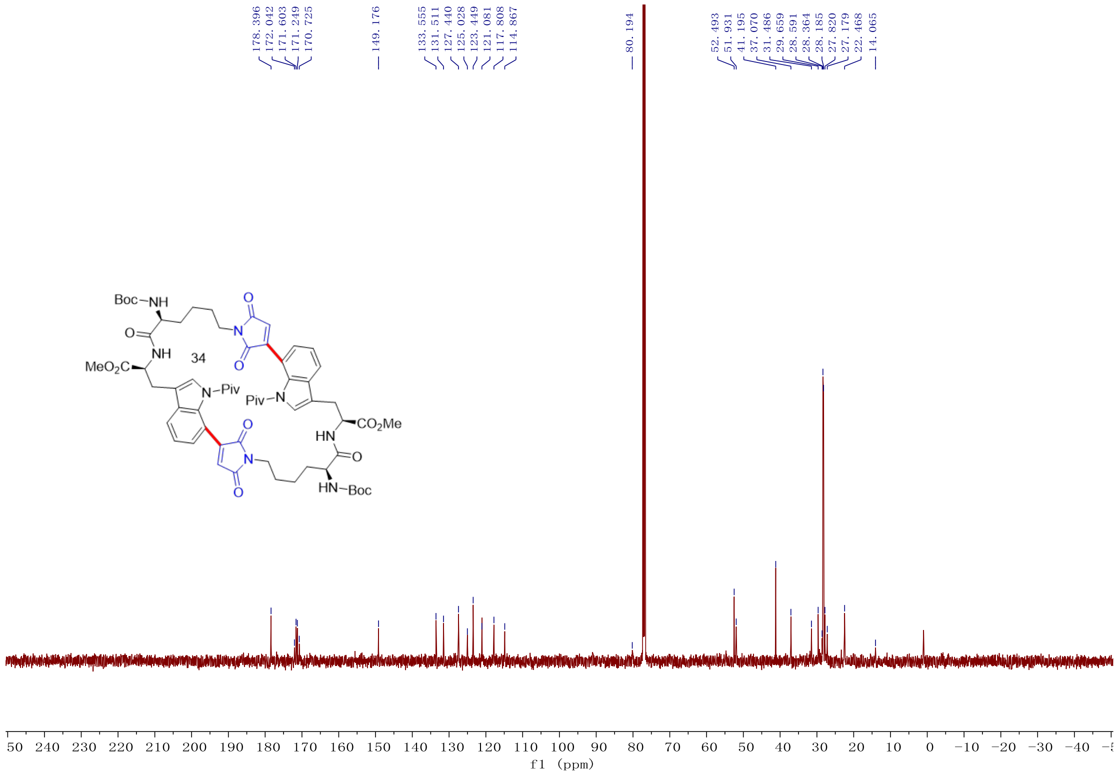

^{1}C NMR (126 MHz, $CDCl_3$) spectrum of **9k**

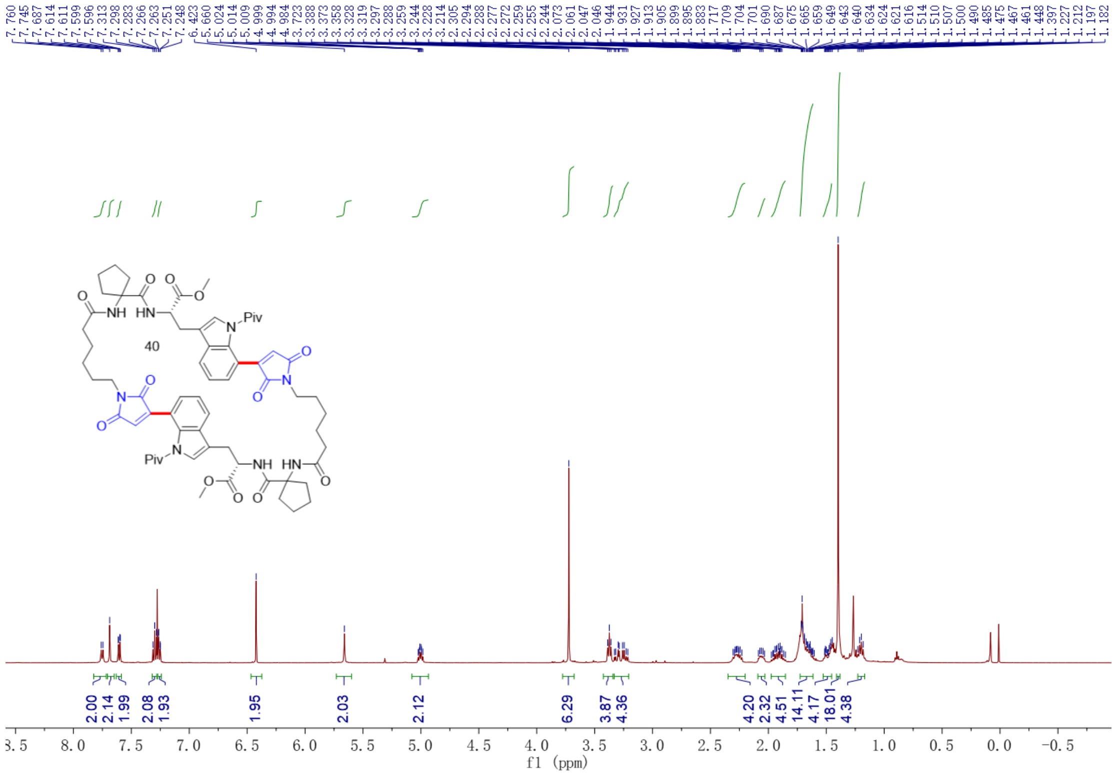

^{1}H NMR (500 MHz, $CDCl_3$) spectrum of **9l**

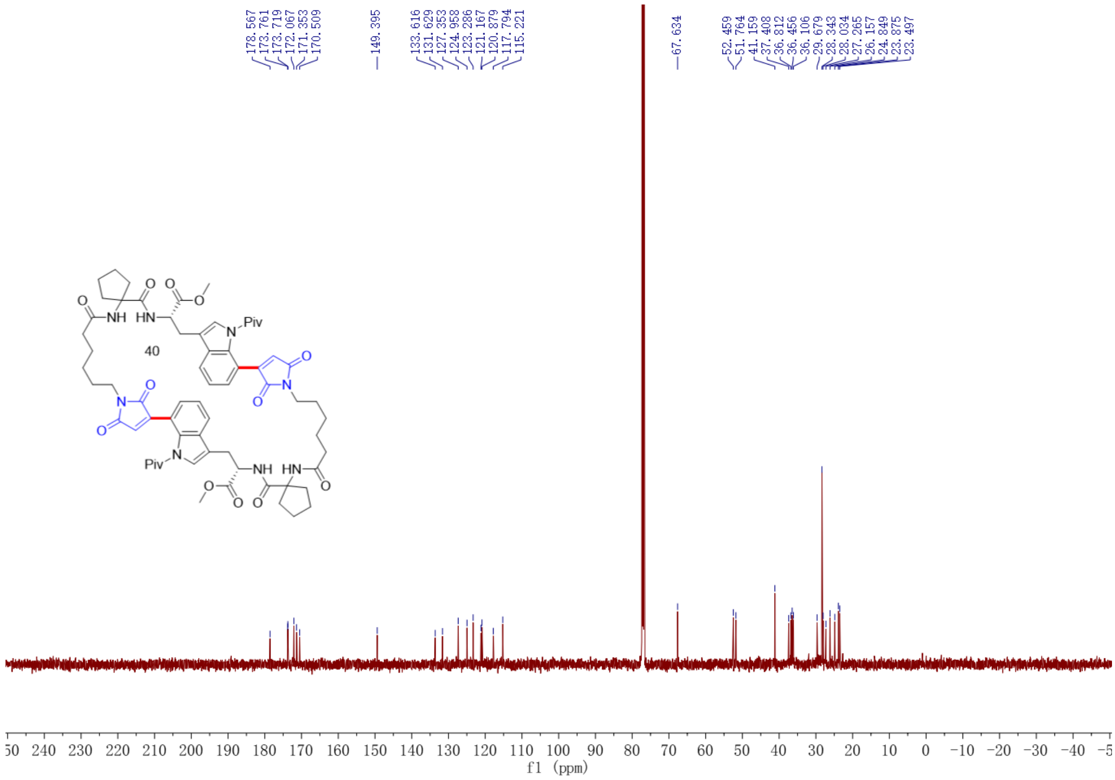

^{1}C NMR (126 MHz, $CDCl_3$) spectrum of **9l**

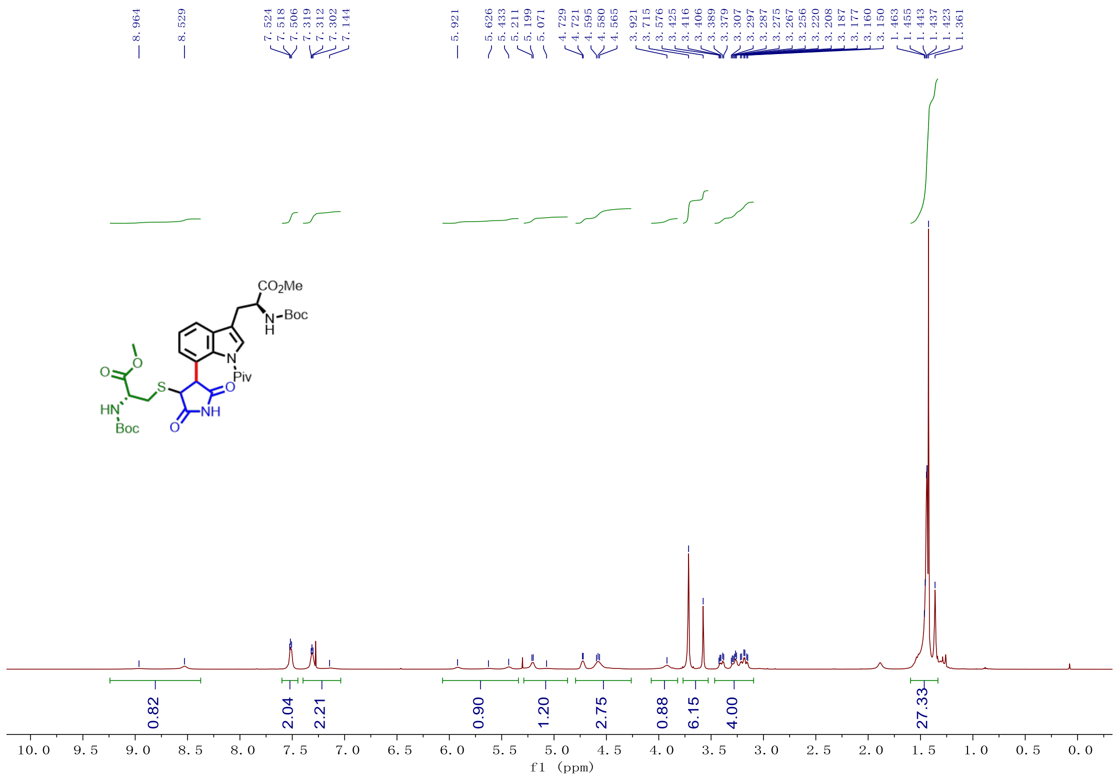

^{1}H NMR (500 MHz, $CDCl_3$) spectrum of **12a**

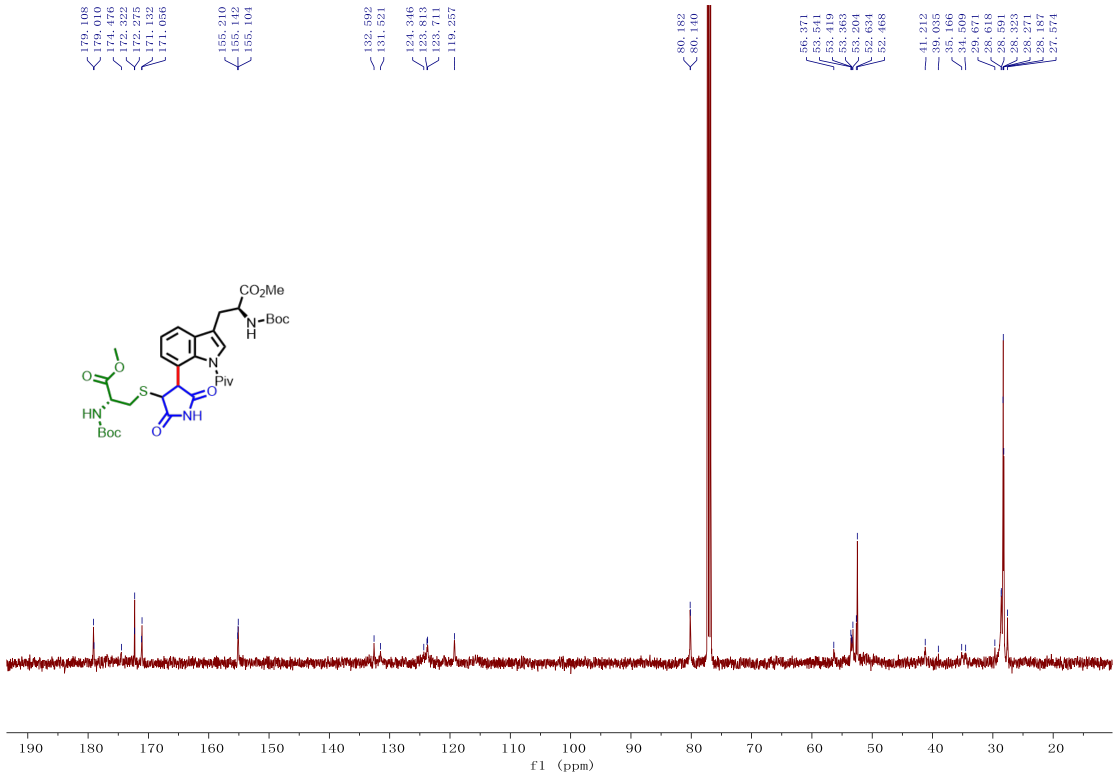

^{1}C NMR (126 MHz, $CDCl_3$) spectrum of **12a**

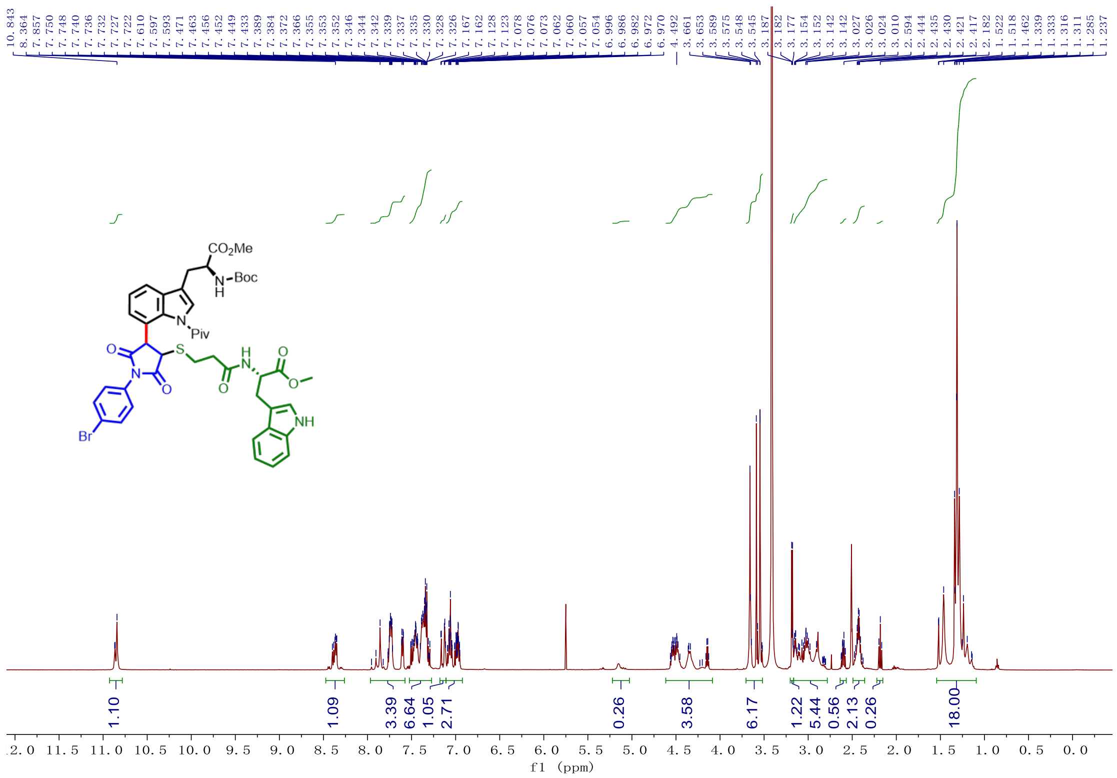

^{1}H NMR (500 MHz, DMSO) spectrum of **12b**

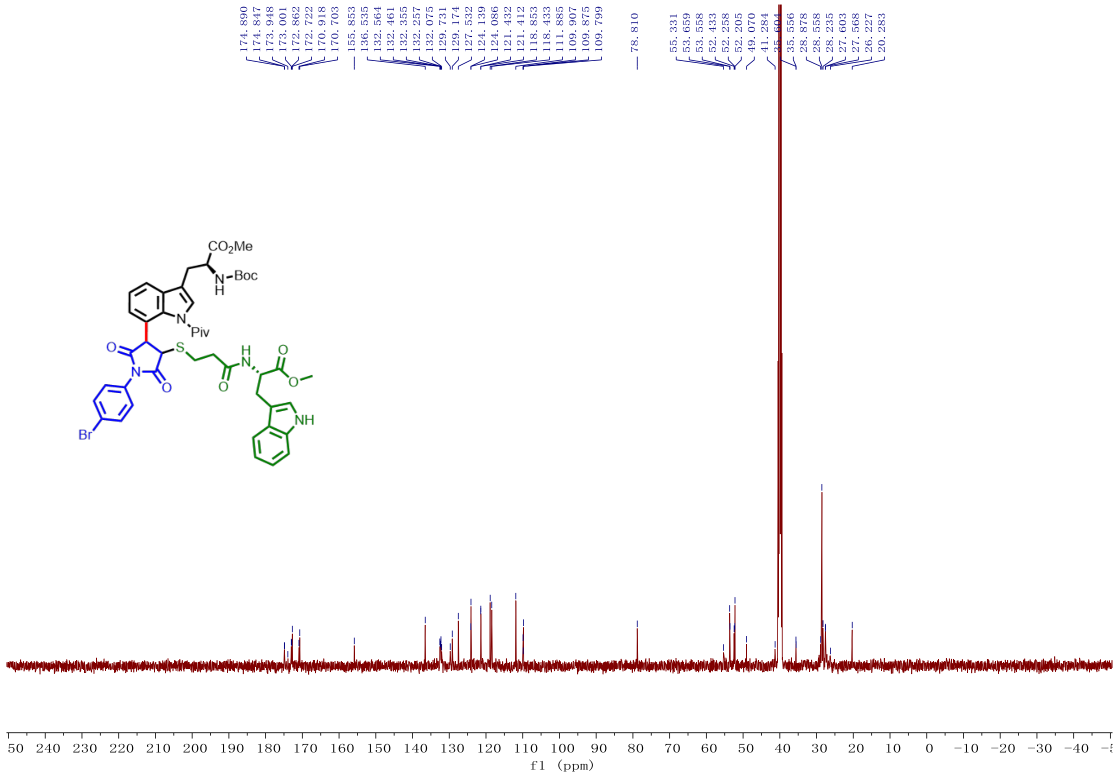

^{1}C NMR (126 MHz, DMSO) spectrum of **12b**

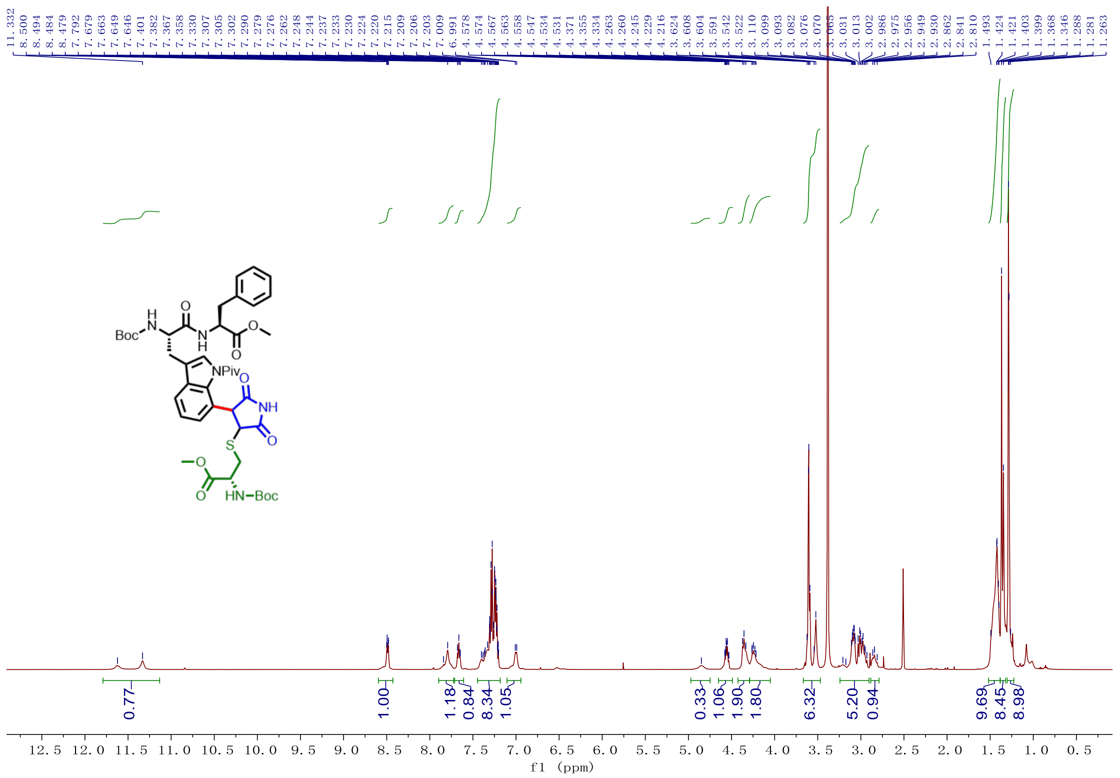

^{1}H NMR (500 MHz, DMSO) spectrum of **12c**

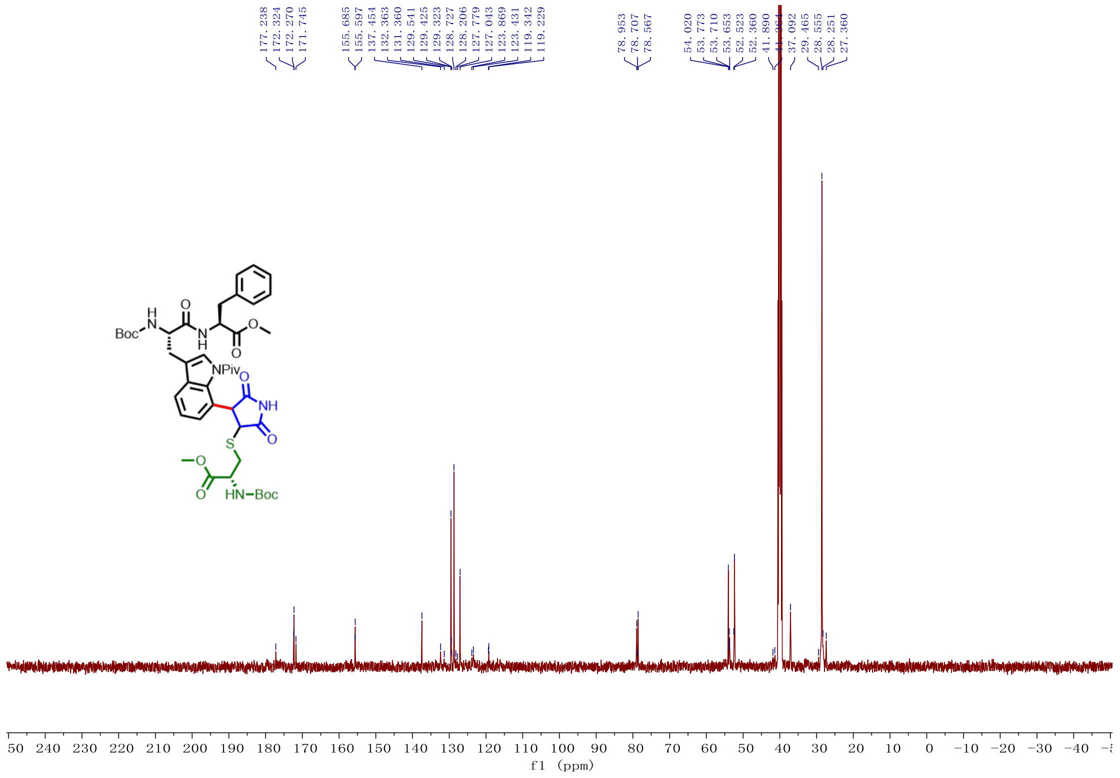

[1]C NMR (126 MHz, DMSO) spectrum of **12c**

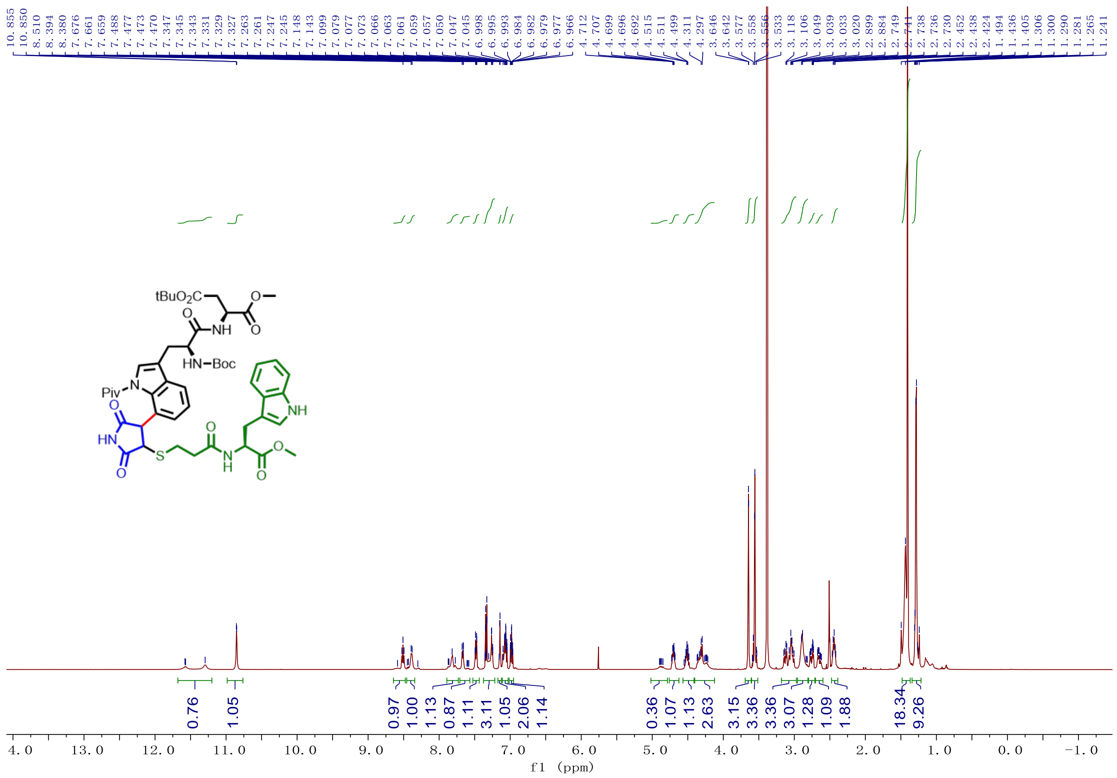

^{1}H NMR (500 MHz, DMSO) spectrum of **12d**

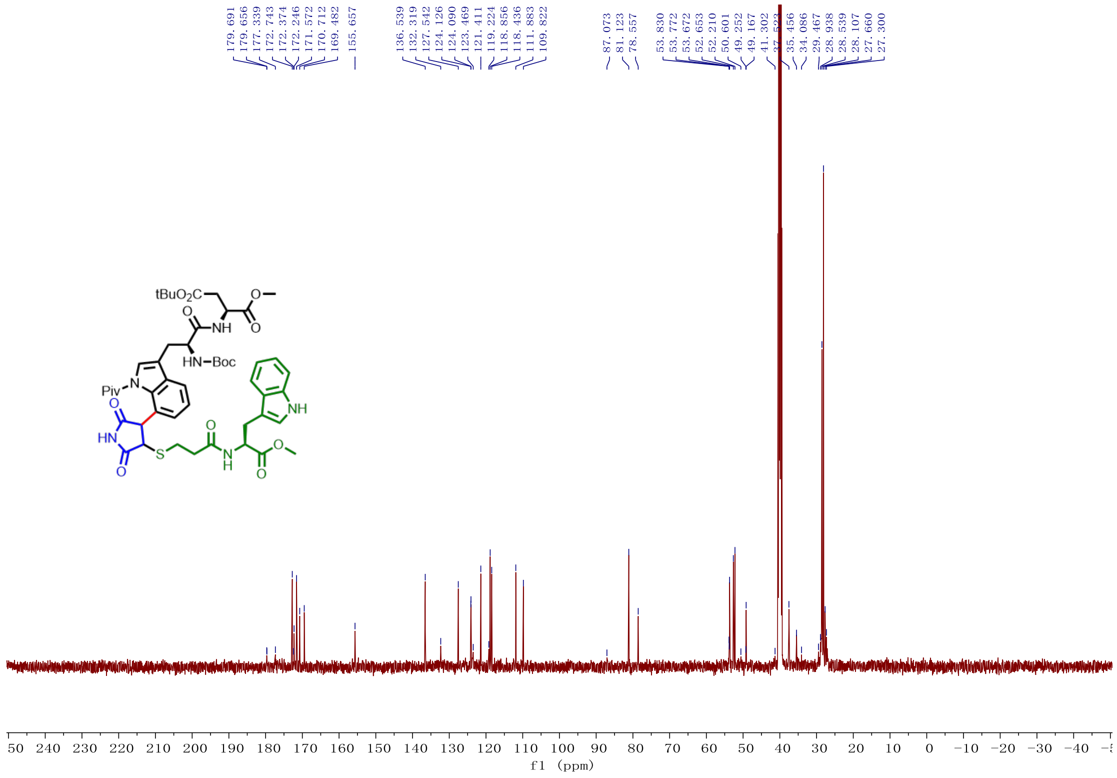

[1]C NMR (126 MHz, DMSO) spectrum of **12d**

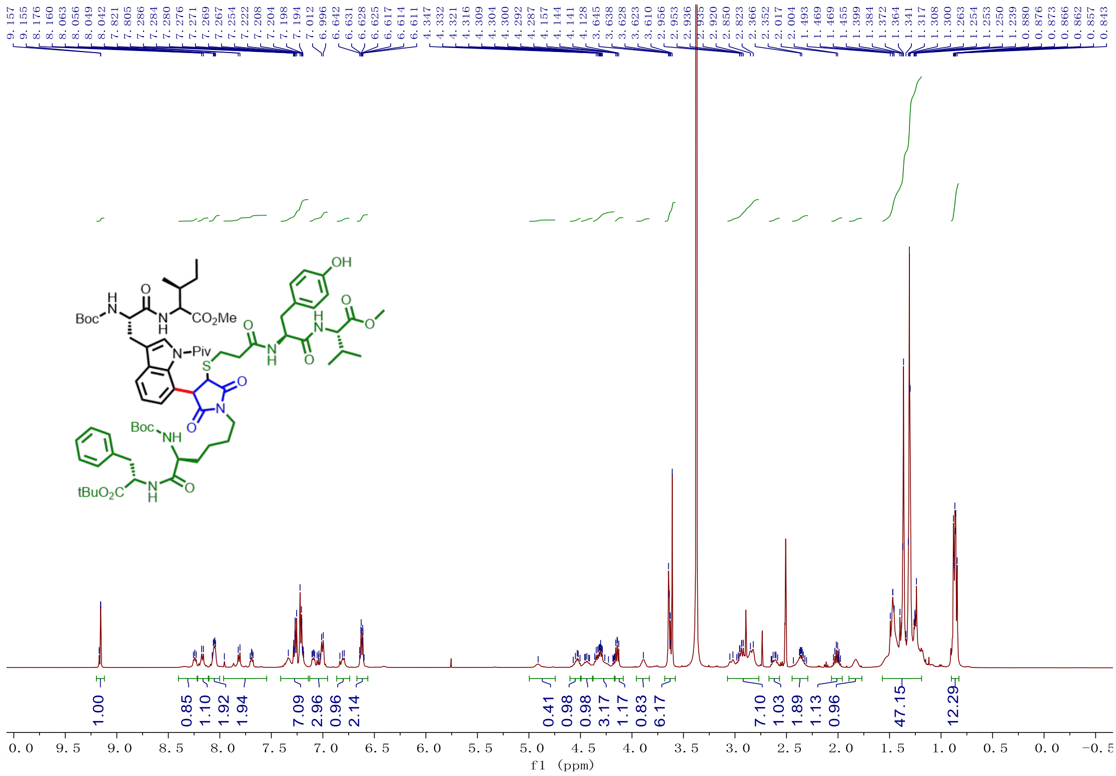

^{1}H NMR (500 MHz, DMSO) spectrum of **12e**

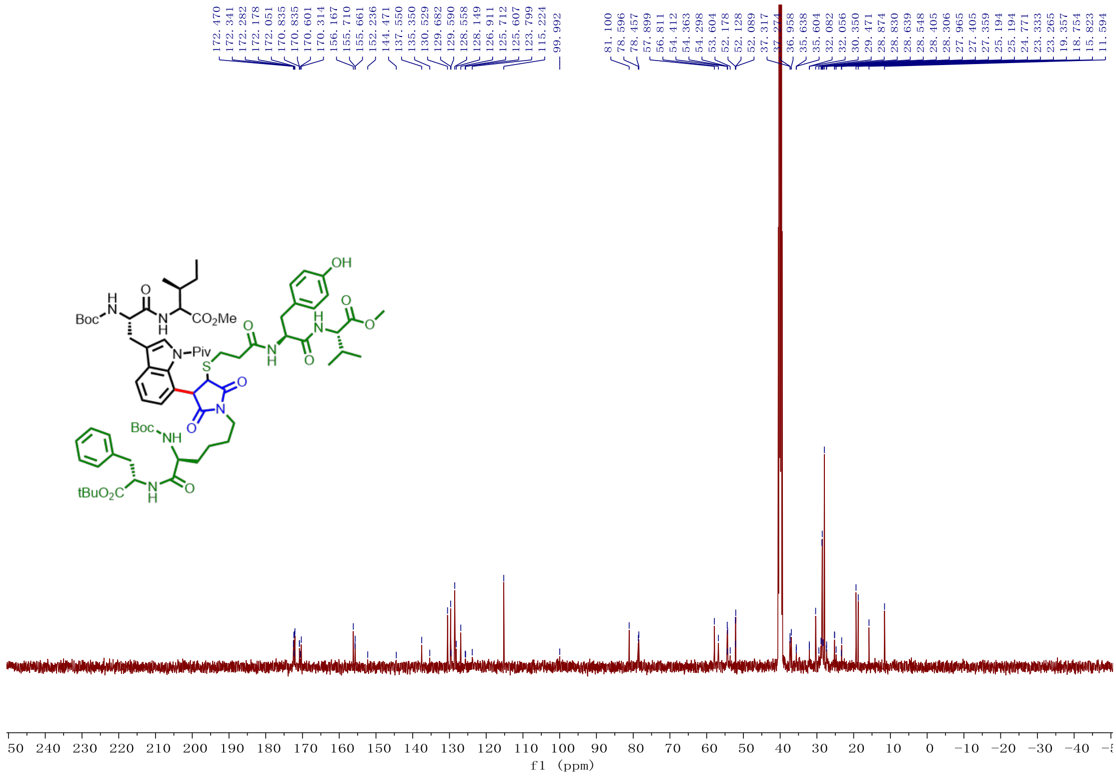

^{1}C NMR (126 MHz, DMSO) spectrum of **12e**

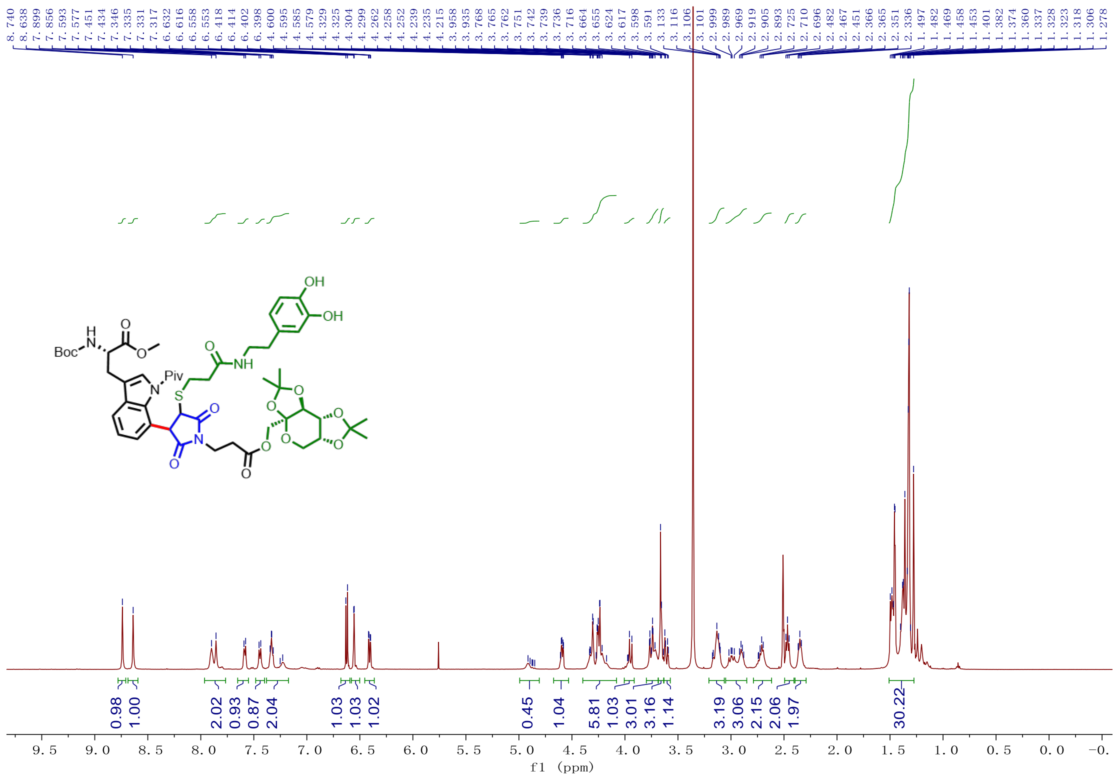

^{1}H NMR (500 MHz, DMSO) spectrum of **12f**

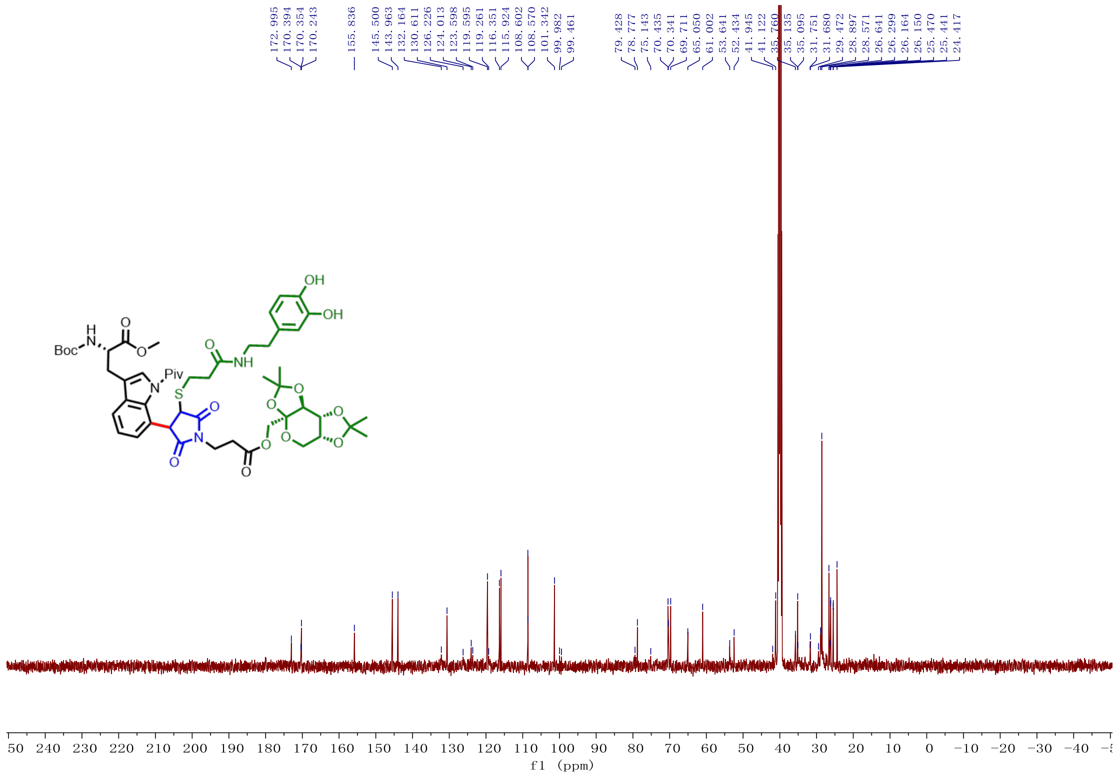

[1]C NMR (126 MHz, DMSO) spectrum of **12f**

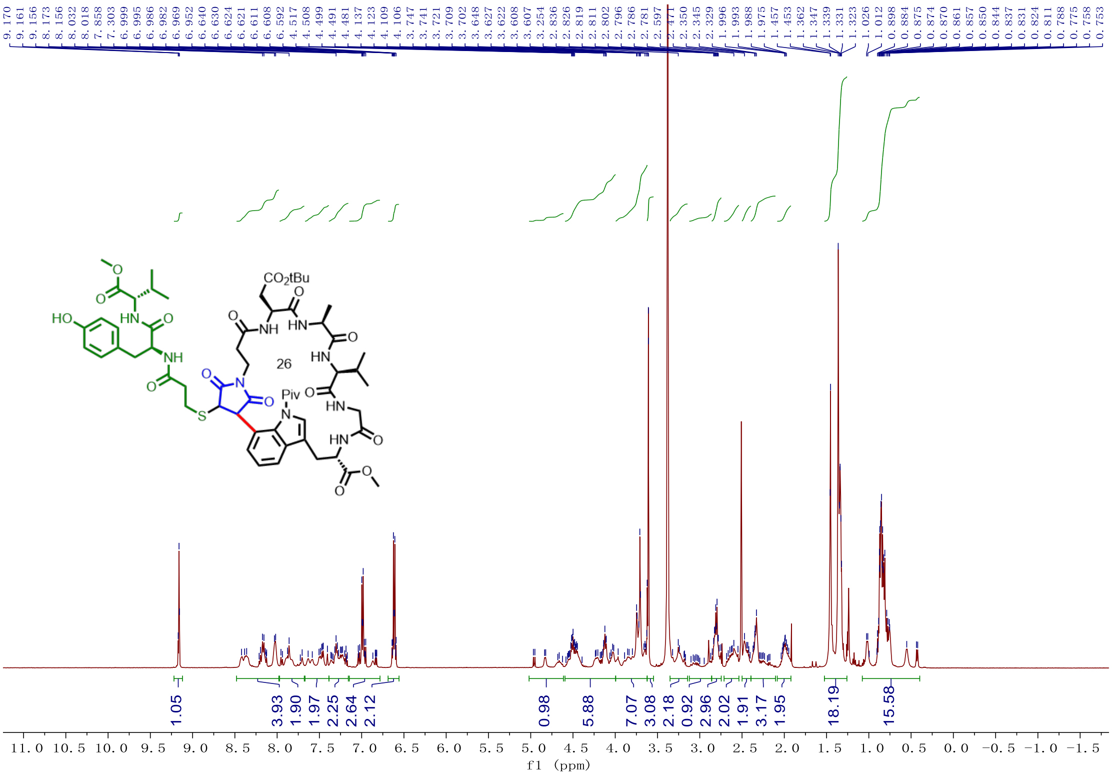

^{1}H NMR (500 MHz, DMSO) spectrum of **12g**

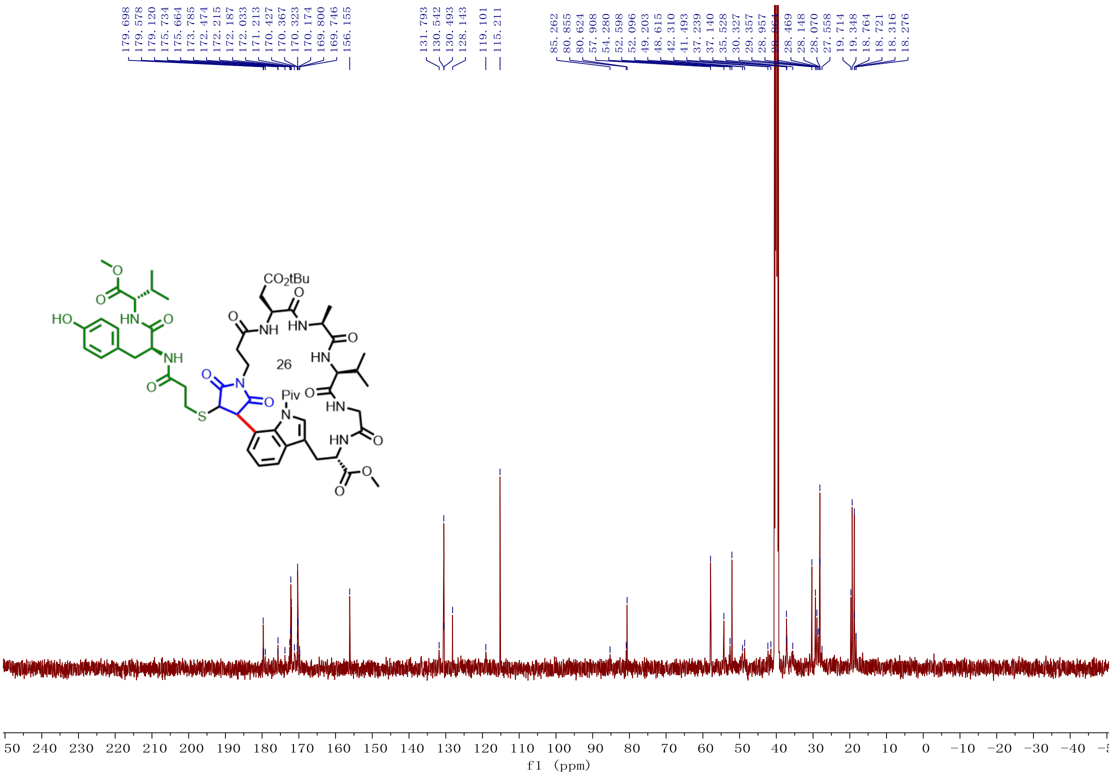

^{1}C NMR (126 MHz, DMSO) spectrum of **12g**

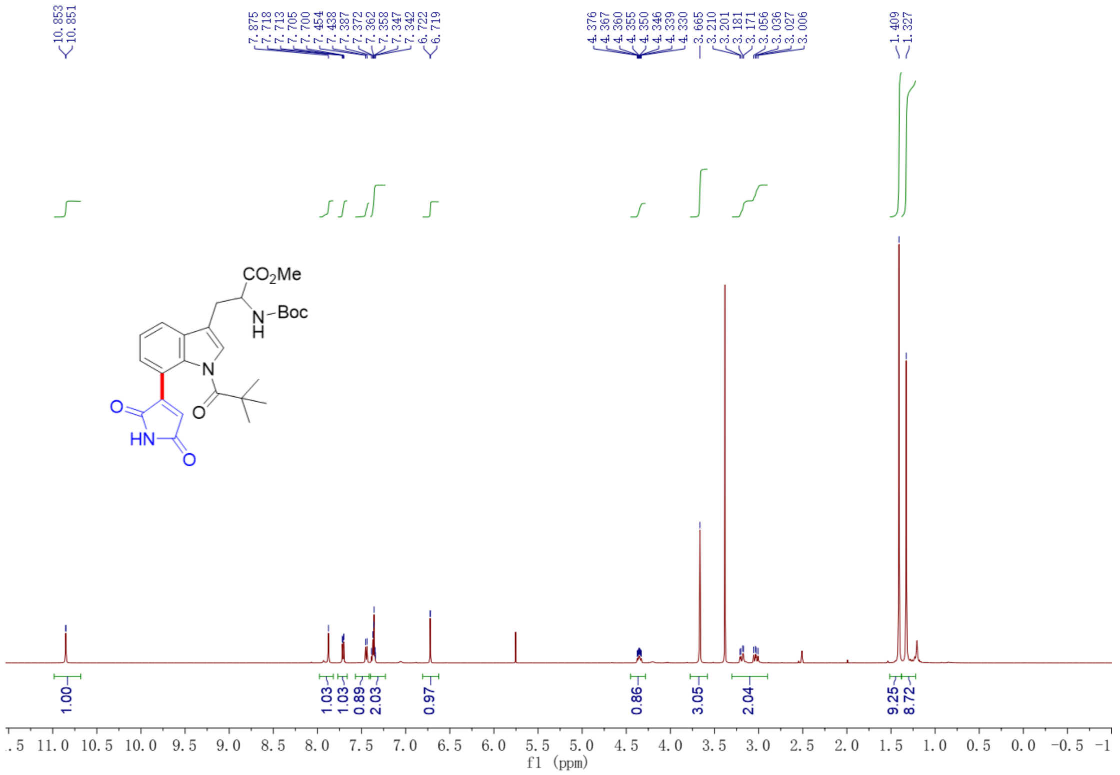

^{1}H NMR (500 MHz, DMSO) spectrum of ***rac*-3a**

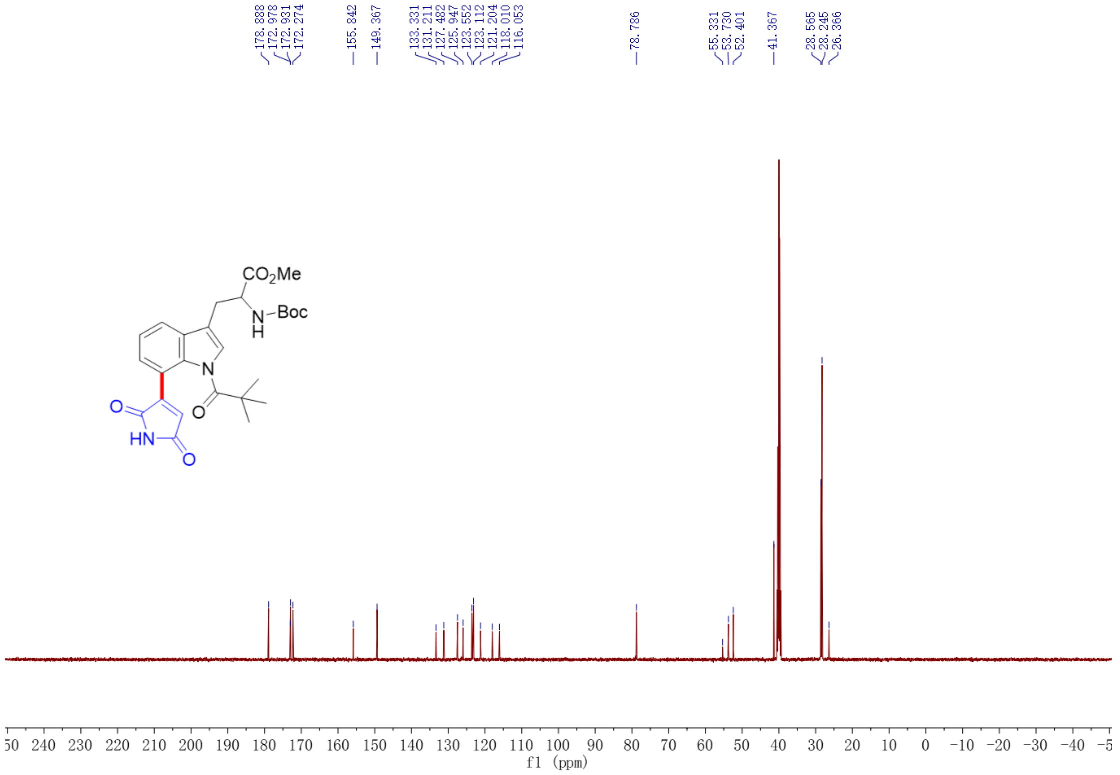

^{1}C NMR (126 MHz, DMSO) spectrum of ***rac*-3a**

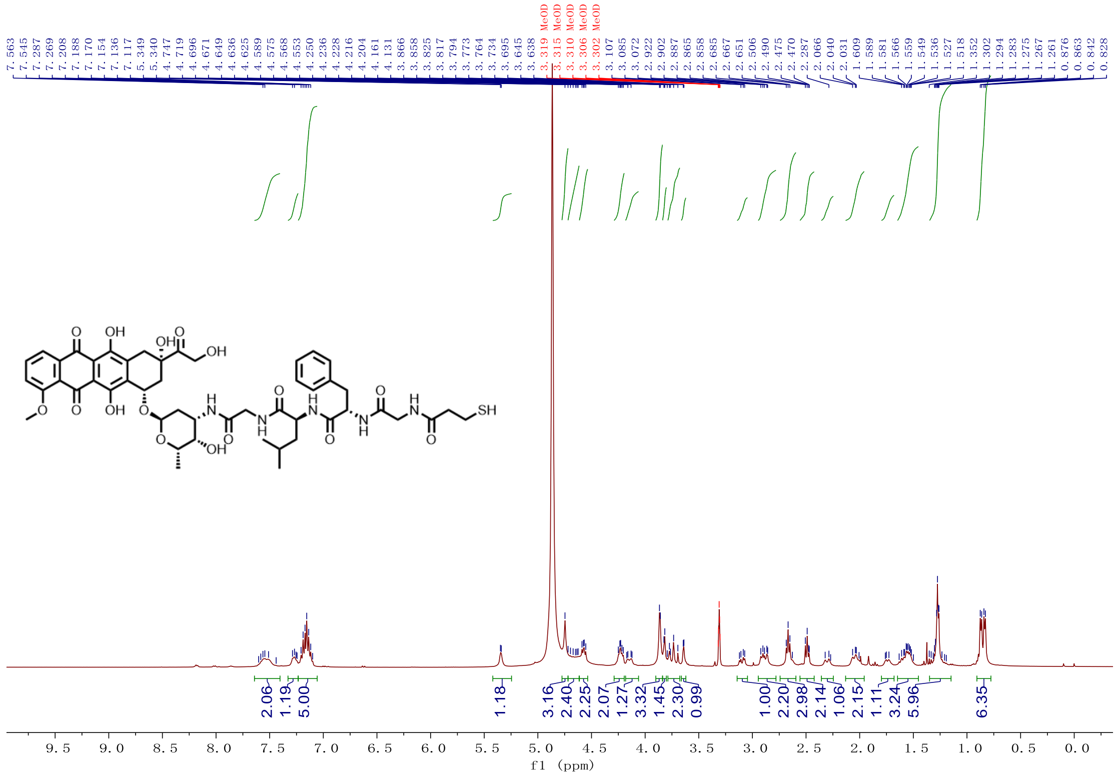

¹H NMR (400 MHz, MeOD) spectrum of **15**

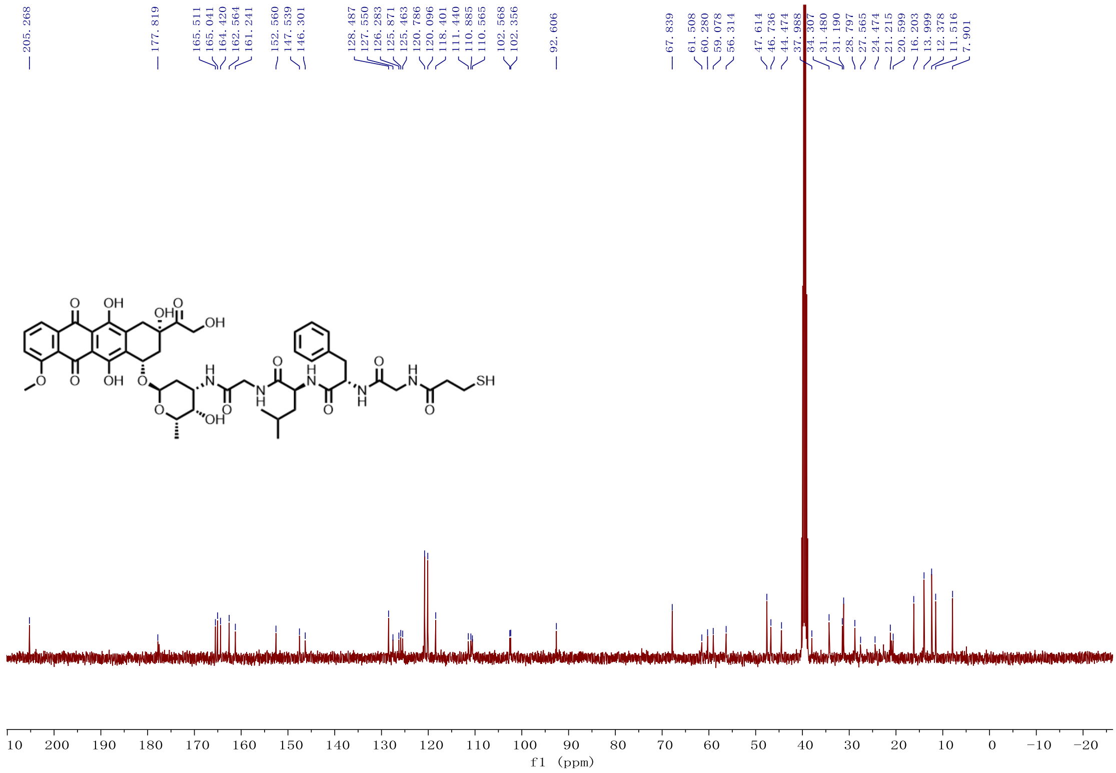

¹C NMR (101 MHz, MeOD) spectrum of **15**

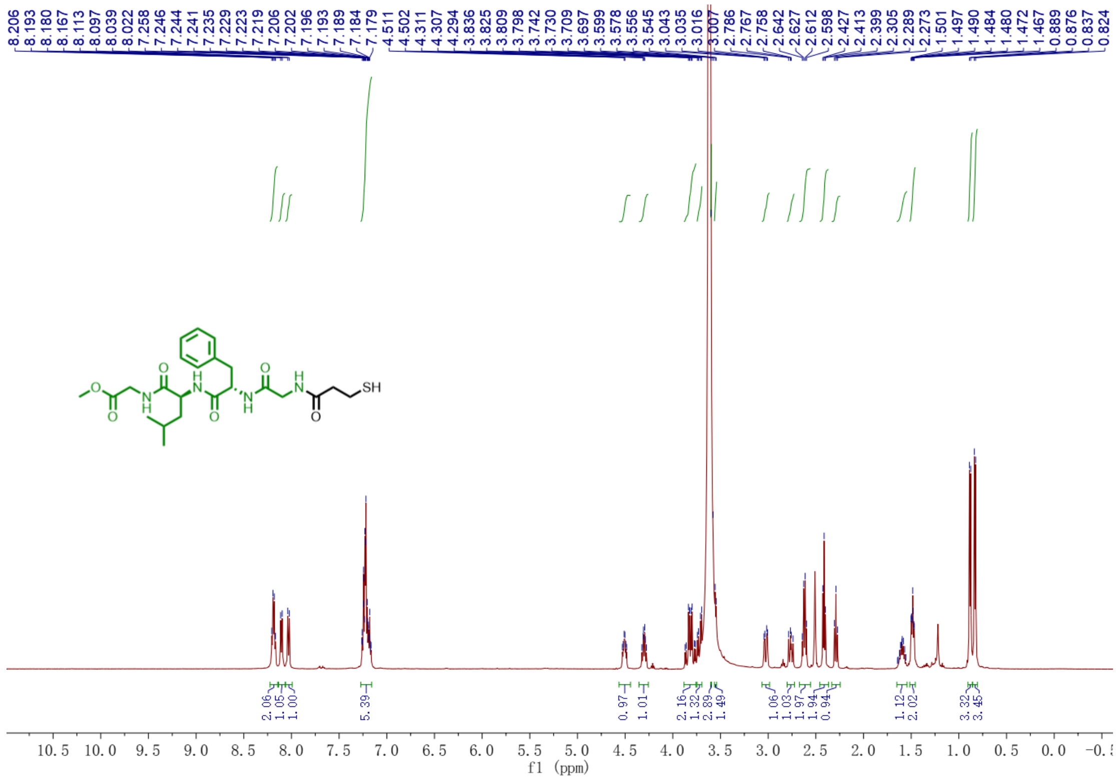

^{1}H NMR (500 MHz, DMSO) spectrum of **17**

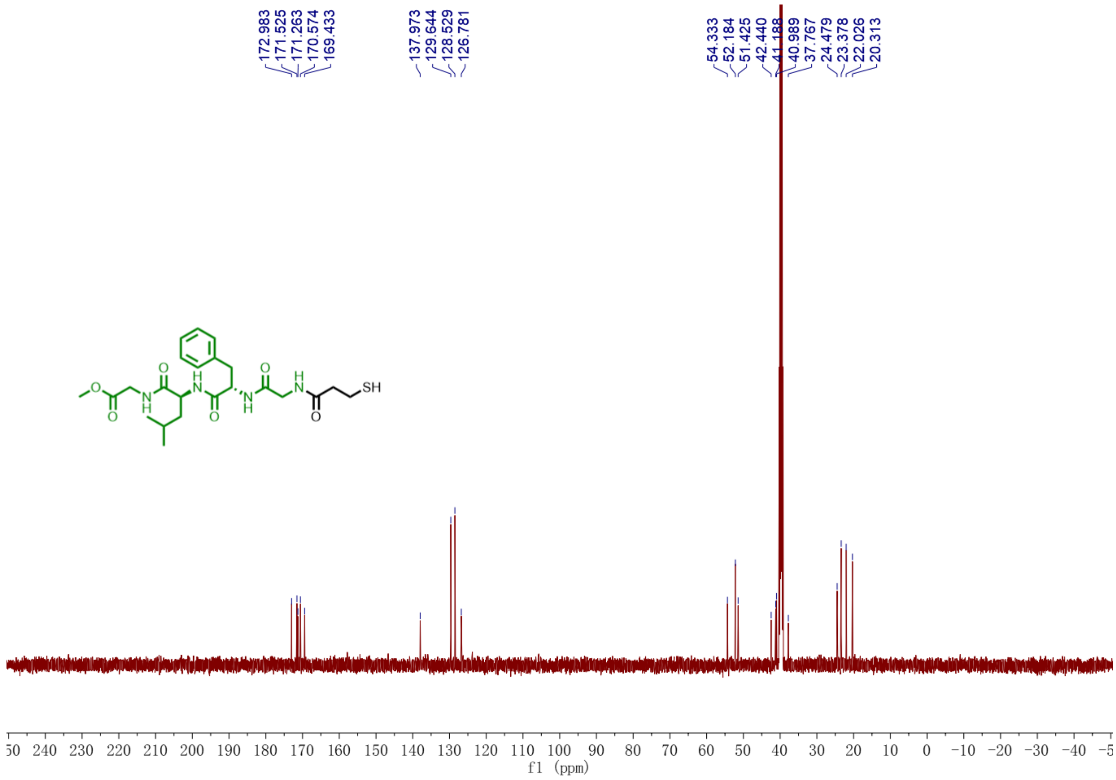

^{1}C NMR (126 MHz, DMSO) spectrum of **17**
